# Prospective Validation of Facial Nerve Monitoring to Prevent Nerve Damage During Robotic Drilling

**DOI:** 10.3389/fsurg.2019.00058

**Published:** 2019-10-01

**Authors:** Juan Ansó, Cilgia Dür, Mareike Apelt, Frederic Venail, Olivier Scheidegger, Kathleen Seidel, Helene Rohrbach, Franck Forterre, Matthias S. Dettmer, Inti Zlobec, Klaus Weber, Marco Matulic, Masoud Zoka-Assadi, Markus Huth, Marco Caversaccio, Stefan Weber

**Affiliations:** ^1^ARTORG Center for Biomedical Engineering, University of Bern, Bern, Switzerland; ^2^Department of Head and Neck Surgery, Inselspital, University of Bern, Bern, Switzerland; ^3^Department of Otolaryngology-Head and Neck Surgery, University Hospital of Montpellier, Montpellier, France; ^4^Department of Neurology, Inselspital, University of Bern, Bern, Switzerland; ^5^Department of Neurosurgery, Inselspital, University of Bern, Bern, Switzerland; ^6^Vetsuisse Faculty, Veterinary Hospital, University of Bern, Bern, Switzerland; ^7^Institute of Pathology, University of Bern, Bern, Switzerland; ^8^AnaPath Services, Liestal, Switzerland; ^9^CAScination, Bern, Switzerland; ^10^MED-EL Medical Electronics, Innsbruck, Austria

**Keywords:** robotic surgery, robotic cochlear implantation, neurophysiology monitoring, monopolar and bipolar, nerve stimulation electrode

## Abstract

Facial nerve damage has a detrimental effect on a patient's life, therefore safety mechanisms to ensure its preservation are essential during lateral skull base surgery. During robotic cochlear implantation a trajectory passing the facial nerve at <0.5 mm is needed. Recently a stimulation probe and nerve monitoring approach were developed and introduced clinically, however for patient safety no trajectory was drilled closer than 0.4 mm. Here we assess the performance of the nerve monitoring system at closer distances. In a sheep model eight trajectories were drilled to test the setup followed by 12 trajectories during which the ENT surgeon relied solely on the nerve monitoring system and aborted the robotic drilling process if intraoperative nerve monitoring alerted of a distance <0.1 mm. Microcomputed tomography images and histopathology showed prospective use of the technology prevented facial nerve damage. Facial nerve monitoring integrated in a robotic system supports the surgeon's ability to proactively avoid damage to the facial nerve during robotic drilling in the mastoid.

## Introduction

Cochlear implants allow to treat severe sensorineural deafness and so far more than 324,200 (by the end of 2012) have been implanted worldwide (1). If a patient's residual hearing can be preserved during surgery, speech recognition is improved (2). Key-hole approaches to the middle and inner ear for cochlear implantation can automate the drilling process and electrode insertion can be more consistent with hypothesized better auditory outcomes (3, 4). The conventional mastoidectomy and posterior tympanotomy is replaced by a linear tunnel reaching from the mastoid surface to the insertion site on the cochlea, passing between the facial nerve and the chorda tympani at distances of <1 mm (5, 6). Optimal electrode insertion angles even advocate a margin of <0.5 mm (7). Facial nerve damage has detrimental effects on a patient's life (8). Therefore, in the conventional approach, the facial nerve is surgically exposed (skeletonized) and risk of facial nerve palsy is minimal (<0.8 %) (9), in most cases only a transitory problem. However, during a keyhole approach sufficient clearance cannot be confirmed visually, hence accurate stereotactic image guidance technology is required, backed up by redundancy to provide for fail safe operation.

**Figure 1:**
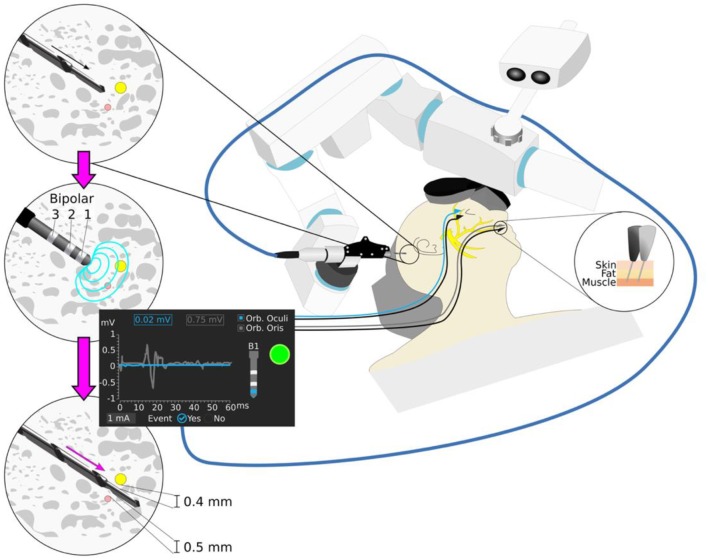
Robotic cochlear implantation supported by facial nerve monitoring. Robotic drilling is performed from the surface of the mastoid to a first safety point 3 mm before the facial nerve (yellow) and the chorda tympani nerve (pink). The robot drill is retracted and the stimulating probe with monopolar and bipolar configurations (Bipolar 1,2,3) is inserted to the end of the trajectory, following application of a stimulation intensity ramp (0.2–2 mA, 4 Hz, 250 μs). The minimum intensity that elicits a muscle evoked potential above threshold is registered for each configuration. Different stimulus thresholds are expected to provide different prediction of distance to the facial nerve. A stimulation intensity above 0.35 mA (bipolar configuration) suggest sufficient facial nerve distance to ensure structural preservation of the facial nerve (e.g., 1 mA stimulus threshold bipolar B1).

Clinically, key-hole cochlear implantation using patient-specific stereotactic frames (10) and our previously reported task specific robot system (11–13) have been demonstrated. We consider surgical robotic technology to be superior over stereotactic frames, as it can control forces, irrigation and feed forward rates accurately during drilling processes and provide coherent integration of necessary safety mechanisms (14). Among other safety elements, we had integrated facial nerve monitoring (FNM) to trace the functional integrity of nerves during the first attempts of robotic keyhole surgery (3, 4) (Figure 1).

Intraoperative nerve monitoring was first proposed for use in lateral skull base surgery (15) and was subsequently introduced to many other surgical disciplines such as Ear-nose-throat (ENT) (8, 16), spinal (17, 18), and cranial neurosurgery (19, 20). While facial nerve monitoring has become a well-accepted clinical utility during certain otologic surgeries (21, 22), our understanding of its performance during key-tunneling approaches is still in its infancy. We previously demonstrated, that conventional monopolar facial nerve monitoring is of only limited use during robotic middle ear access, because it does not give conclusive feedback of nerve proximity at close range (<1 mm) (23).

Hence, we proposed a nerve monitoring approach with variable monopolar and bipolar stimulation intensity and where muscle response are analyzed to determine whether a surgical drill is too close to the facial nerve (24) (Figure 1). As the drill passes the facial nerve, stimulus thresholds (minimum stimulation intensity evoking a muscle action potential response) are sampled at pre-defined locations. The bipolar configurations have a localized electrical field, allowing to distinguish nerve proximity at closer distances than the monopolar configuration (19). In an initial pre-clinical *in vivo* experience, the system was able to determine potential collisions (distance <0.1 mm, stimulation threshold <0.35 mA) with the facial nerve (24). Different stimulation channels provide different penetration depths of the electrical current, thus higher sensitivity is associated to a monopolar configuration and higher specificity to a bipolar. From four stimulation channels studied (bipolar 1, 2, 3, and monopolar), it was demonstrated that bipolar stimulation (distance cathode-anode of 2 mm) is the most reliable to determine a potential collision with the facial nerve (<0.1 mm). The system was then introduced clinically (25) in six patients, and its predicted facial nerve clearance correlated with the distances measured in the intraoperative CT scans (12). For patient safety, trajectories were drilled with a safety margin to the facial nerve of 0.4 mm.

Here, we aim to validate the performance of the system in distance ranges between potential collision and sufficient clearance to the facial nerve (0.0 < distance <0.4 mm). We hypothesize that the surgeon is able to abort the robot assisted procedure based on the facial nerve monitoring feedback, without support on systems navigation, before structurally damaging the facial nerve. We carried out an *in vivo* study (sheep model), where trajectories were planned and drilled in the mastoid at pre-defined low distances from the facial nerve. The ENT surgeon stopped the drill process if the system suggested a collision with the facial nerve. Post-mortem micro-computed tomography images and histopathology were used to evaluate the nerve monitoring system's ability to support the surgeon in avoiding facial nerve structural damage.

## Materials and Methods

### *In-vivo* Experiment

#### Study Design

The study was approved by the Bernese cantonal animal commission (license number BE56/12). The study was designed as prospective and observer-blinded. In total, a number of 24 drilling attempts (eight trajectories in three animals) with varying distances (0 to 1.5 mm) to the facial nerve were carried out. Following a redesign of a FNM stimulation probe as part of a commercial development and in order to confirm expected system performance (24, 25), the first animal served for training (training trajectories) and the last two for prospective assessment of the nerve monitoring approach. In the training trajectories, the surgeon is asked to continue drilling even if the system suggests of a collision with the facial nerve. In the prospective trajectories the surgeon will stop robotic drilling in case the system suggests collision with the facial nerve. A sheep model was chosen, because it provides similar mastoid and facial nerve anatomy to human (26), whilst the non-existence of air cells (27) is considered to be of no relevance.

Per drill attempt, the trajectory was planned using pre-operative computed tomography image data and drilled by a surgical robotic system for cochlear implantation. Nerve monitoring was applied at pre-defined positions near the facial nerve. The system was operated by an ENT surgeon who was not aware of the specifics of the drill trajectory relative to the facial nerve (observer role). After each nerve monitoring measurement, the ENT surgeon was presented with the findings of the measurement and asked to terminate or continue the drilling process based on stimulation threshold of 0.35 mA derived from a previous study (25). After completion of all trajectories, the subject was sacrificed by 0.5 ml/kg pentobarbital 40%. The mastoid bone region was extracted for post-operative micrometer resolution computed tomography. Potential structural damage of the facial nerve in the proximity of the drilled trajectories was studied via histopathological analysis of each mastoid.

#### Anesthesia Protocol

Animals were pre-medicated with 0.1 mg/kg diazepam and 0.01 mg/kg fentanyl administered intravenously. General anesthesia was induced with an intravenous injection of propofol 1% to effect. Endotracheal intubation was performed, whereby the anesthetic state was maintained by isoflurane in 100% oxygen and fentanyl 0.01 mg/kg/h. Ringers lactate solution was administered at a rate of 5 ml/kg/h. Neuromuscular blockade was avoided to ensure normal facial nerve activity.

#### Site Preparation, Imaging, and Planning

**Figure 2:**
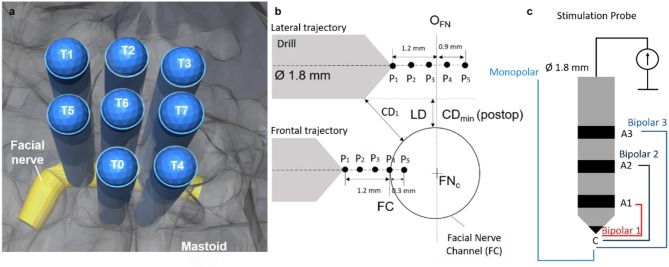
**(a)** Trajectories (*n* = 8) plan relative to the facial nerve. **(b)** Schematic representation: five measurement points planned relative to the origin of the facial nerve in the drill axis (O_FN_) for the lateral trajectories. Five measurement points planned relative to the facial nerve canal (FC) in the frontal trajectories. Lateral distance (LD) planned between 0.00 mm and 0.5 mm. Drill trajectory to nerve closest distance defined as CD. **(c)** Stimulation probe, same diameter as the drill, four different stimulation configurations, bipolar 1 (red), bipolar 2 (dark blue), bipolar 3 (blue) and monopolar (light blue). The monopolar configuration is measured with needle anode near the sternum.

The temporalis muscle was excised and four titanium reference screws (2.2 mm diameter × 5 mm length, Medartis, Switzerland) were implanted near the external auditory canal for subject-to-image registration (28). A CT image (0.4 mm space between slices, 0.8 mm slice thickness) of the animal's head was acquired (Brilliance, Philips AG). A modified version of an otologic surgical planning software (OTOPLAN™) (CAScination AG, Switzerland) (29) was used to segment the facial nerve and to plan the drilling trajectories (1.8 mm Ø) relative to the segmented facial nerve. In subject 1 (training trajectories) an intraoperative O-arm system (Medtronic) was used for pre-operative imaging due to a technical problem with the CT system (0.415 mm pixel spacing, 0.8 mm slice thickness). In each animal eight drilling trajectories were planned relative to the segmented facial nerve (Figure 2a). Five trajectories were defined with closest lateral distances (LD) of 0.5 mm (T0), 0.3 mm (T2, T4), and 0.0 mm (T1, T3). Three trajectories were planned to frontally intersect with the facial nerve (T5, T6, T7). On each trajectory five measurement points (*P*_*N*_, *N* = *5)* were defined (Figure 2b). For the lateral trajectories the first point was defined at an axial distance of 1.2 mm before the facial nerve center and the last point 0.9 mm after (axial increments of 0.54 mm) (25). In frontal trajectories, the first measurement point was 1.2 mm before the facial nerve canal and the last point 0.3 mm inside (axial increments 0.375 mm). Trajectories are herein after referred to by two attributed numbers (x.y): the subject number (1–3) and the trajectory number (0–7), e.g., subject 1 trajectory 6 is labeled as 1.6.

#### Electromyography Setup

Two pairs of subdermal electromyography (EMG) needles (SDN Trigon, Inomed, Germany) were inserted into the facial muscles, orbicularis oculi, and oris; a ground and a stimulation-return needle were placed central to the nose bridge. To verify correct placement of the measuring needles, a positive control method previously describe in Ansó et al. (25) was used. In case one or both channels would depict no compound muscle action potential (CMAP) above threshold (100 μV), the corresponding measuring needle was repositioned in the facial muscle.

#### Drilling and Nerve Monitoring System

The HEARO™ system (CAScination AG, Switzerland) is a neurotological surgical robotic system. It integrates a 5DoF surgical robotic manipulator, an optical tracking camera and a task-specific facial nerve monitoring system. To assess the performance of the nerve monitoring system all integrated safety mechanisms were disabled. The nerve monitoring system consists of a navigated multipolar stimulating probe, stimulation, and monitoring hardware and dedicated graphical user interface functionality to carry out all necessary measurements.

The stimulating probe (Figure 2c) is based on a previously reported design (24), to enable bipolar (i) and monopolar (ii) stimulation with a cathode electrode at the tip and four anode configurations: (i) three concentric anodes rings (configurations B_1, 2, 3_) distally distributed behind the tip of the probe (distances *d* = 2, 4, and 7 mm); (ii) a needle anode electrode placed in a far-field location relative to the stimulating tip acts as anode of the monopolar stimulation. The stimulating probe is navigated via tracking markers integrated in the housing to ensure its complete insertion into the drilled tunnel during facial nerve stimulation.

#### Robotic Guided Nerve Monitoring

Lateral drilling trajectories were randomly selected and loaded to the system, to ensure the ENT surgeon was blinded. Drilling commenced with pre-defined drilling parameters (drilling speed: 1,000 RPM, feed forward rate: 0.5 mms^−1^) and irrigation with saline solution (NaCl 0.9%, room temperature). Upon reaching the first measurement point (Figure 2, P1), the drill was removed, and the drilled tunnel flushed (NaCl 0.9%) for consistency in electrode-tissue contact properties. The FNM probe was inserted to the end of the tunnel and an electrical impedance check of the probe electrodes was done prior to application of the stimulating protocol. Then a ramp of 11 stimulation intensities (0.2, 0.25, 0.3, 0.35, 0.4, 0.5, 0.75, 1.0, 1.25, 1.5, 2.0 mA) was applied to each of the channels of the probe with duration of 250 μs (see Supplemental Digital Content 1 (Supplementary Video 1), which shows how the robotic system drills to the next measurement point, the insertion of the FNM probe and the stimulation protocol).

**Figure 3:**
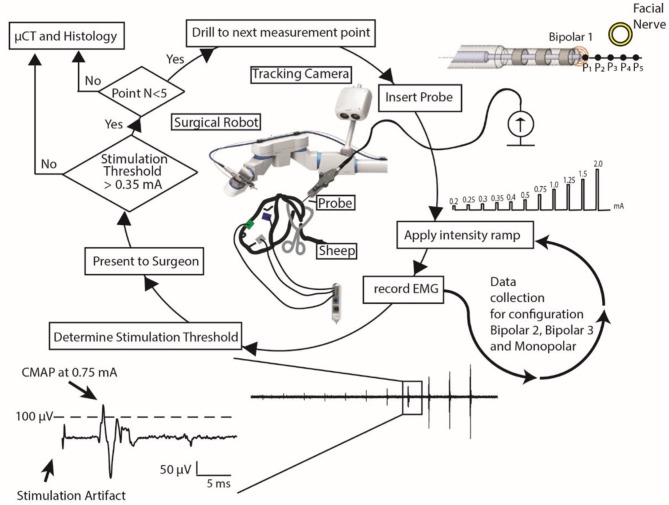
Nerve monitoring workflow: using a surgical robot and a stimulation probe, the surgeon follows this workflow to determine if it is safe to continue to drill or if she should stop. EMG data of only one EMG channel and only for stimulation electrode configuration Bipolar 1 is represented.

Recorded EMG responses were examined in a 50 ms search window after a 3 ms rejection period following the stimulation pulse. The minimum current intensity that produced an CMAP response above threshold (100 μV) was determined and the result for each channel presented to the ENT surgeon (Figure 3). If the stimulation intensity of electrode configuration Bipolar 1 was above 0.35 mA, the ENT surgeon continued drilling to the next measurement point, otherwise the ENT surgeon stopped. Upon completion of all drillings and FNM measurements, subjects were euthanized, and the mastoid removed and preserved in formalin for post-mortem analysis.

### Post-mortem Assessment

#### Drill Trajectory to Facial Nerve Distance Assessment

Mastoids were μCT imaged (isotropic 18 μm, Scanco μCT 40, Scanco Medical, Switzerland). Drilled trajectories and the facial nerve channel were manually segmented (Amira, FEI, United States). Spatial positions of the measurement points (*P*_*i*_*)* were determined along the segmented trajectory axes and the distance between the trajectory and the facial nerve were measured (Matlab, The Mathworks, 2016a). The minimum closest distance measured between each drilled tunnel and the FN was defined as CD_min_ (Figure 2). The trajectories were classified in two groups: (i) lateral and (ii) frontal, and lateral trajectories were subcategorized as facial nerve (CD_min_) above or below 0.1 mm. Each trajectory was labeled as X.Y with X being defined as the subject number (1–3) and Y as the trajectory number within the subject (0–7) (e.g., trajectory 1.0 stands for subject1-trajectory0).

#### Histopathology to Determine Structural Nerve Damage

Histopathologic inspection was used to study structural nerve integrity and potential damage in all subjects. Mastoid bones were first stored in EDTA for initial decalcification, and then, bones were gently decalcified using a 10% formic acid base (Anapath GmbH) until they became suitably soft to be sliced. Sections were taken at an approximate thickness of 4 μm, and at steps of 60 μm. The resulting sections (50–60 per subject) were stained by hematoxylin and eosin (H&E), before undergoing histological evaluation. Pictures were taken by an UC30 camera.

#### FNM Performance to Determine Critical Nerve Proximity

For the prospective assessment of FNM (Subjects 2–3), each measurement point was classified as “outside” or “inside” the nerve canal based on visual inspection in the histology slices. Sensitivity and specificity were calculated comparing the histology assessment with the intraoperative FNM “unsafe” vs. “safe” assessment. For comparison of these findings with the previous retrospective pre-clinical study (25, 30), we replaced not drilled/measured points by a best/worst case scenario. To calculate confidence intervals, the efficient score method corrected for continuity was used (31). All measurement points (S1–S3) were retrospectively classified based on the measured distance to the facial nerve in the μCT images. Positive and negative predictive values were determined (PPV and NPV, respectively) for all stimulation intensities and distances for which PPV and NPV were above 95%. Youden's *J*-test (32) was used to derive the highest sensitivity and specificity result as a function of stimulation intensity.

## Results

A total of 24 trajectories were drilled in three subjects, each tunnel consisting of up to five measurement points at varying distances from the facial nerve canal. During the experiment, breakage of the bony walls during insertion of the probe (trajectory 3.6) lead to exclusion of one trajectory. Difficulties to determine the location of the facial nerve in the post-mortem imagery lead to exclusion of three more trajectories (exclusion 4/24 trajectories; inclusion 20/24 trajectories). After μCT, we classified the trajectories in three types based on facial nerve distance: (a) lateral and FN distance > 0.1 mm (*n* = 6), (b) lateral and FN distance <0.1 mm (*n* = 6), and (c) frontal (*n* = 8). In total, 96 FNM points were assessed, 40 FNM points in subject 1 (training trajectories) and 56 points in subjects 2, 3 (prospective trajectories). Facial nerve distances ranged from 0.0 to 1.5 mm, with 35 points (prospective, 35/56 = 62.5%) below 0.4 mm (range of interest of this study). An overview of the recorded CMAP data (from monopolar stimulation) can be found on the Supplemental Digital Content 2 (Supplementary Data Sheet 1), which shows the CMAP responses to stimulation for each measurement point.

### Stimulation Thresholds vs. Potential Facial Nerve Damage

#### Training Trajectories

**Figure 4:**
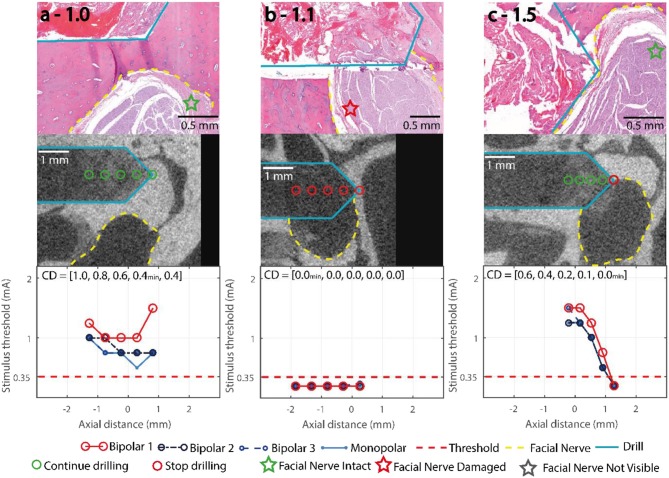
Representative trajectories of the training subject, stimulation thresholds of the FNM (bottom row), with the decision thresholds and the decisive electrode configuration marked in red, and electrode configurations Monopolar, Bipolar 2 and Bipolar 3 (blue). μCT (middle row), drill outlined in turquoise, measurement points marked red, facial nerve outlined in yellow. Histology (top row) with H&E staining, facial nerve assessment marked with a star. **(a)** 1.0: lateral distance > 0.4 mm, no warning from system. **(b)** 1.1: lateral distance < 0.0 mm, stimulus threshold below 0.35 mA indicates an emitted system warning at the first FNM point. **(c)** 1.5: frontal trajectory with final nerve distance < 0.1 mm, the system warned at the deepest FNM point.

All trajectories were drilled to the deepest measurement point. Figure 4 shows three representative trajectories of the training subject. Nerve monitoring suggested trajectories 1.0 (Figure 4a) and 1.4 with distances to the facial nerve above 0.1 mm (Bipolar 1 > 0.35 mA), confirmed in μCT (CD_min_ > 0.2 mm). In lateral trajectories 1.1 (Figure 4b), 1.2 and 1.3 the FNM system suggested critical FN proximity below 0.1 mm and subsequently the system warns the ENT surgeon (Bipolar 1 ≤ 0.35 mA), confirmed in μCT (CD_min_ < 0.0 mm). In the frontal trajectories 1.5 (Figure 4c), 1.6, and 1.7, the FNM determined critical nerve distance at the deepest measurement point (P_5_), confirmed in μCT (CD_min_ > 0.2 mm). Histopathology determined structural damage to the facial nerve in trajectory 1.1 (Figure 4b).

#### Prospective Trajectories

**Figure 5:**
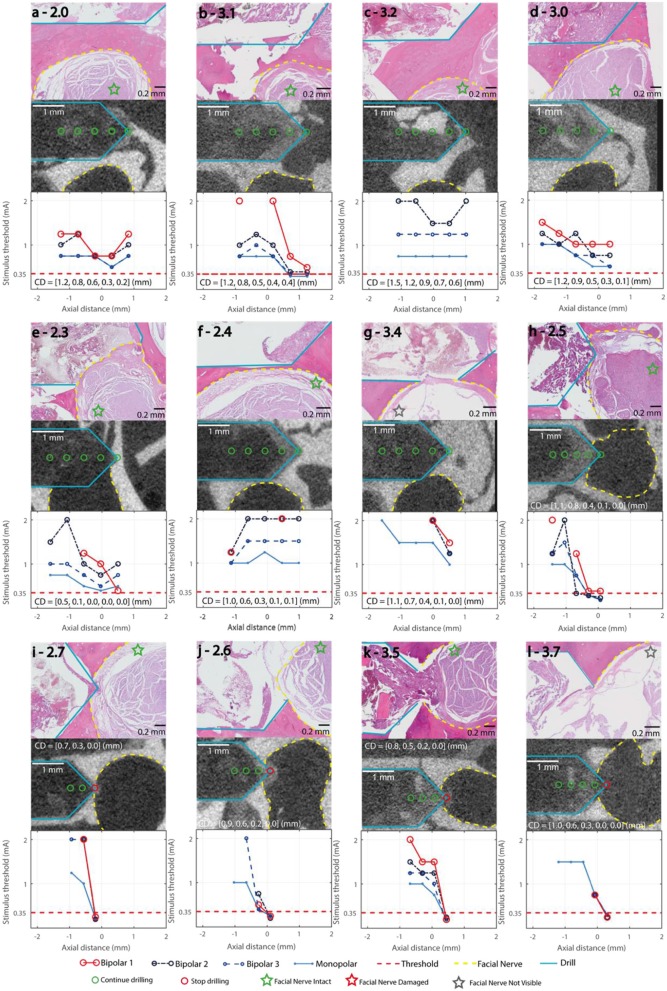
Stimulation Thresholds of the FNM (bottom row), with the decision thresholds and the decisive electrode configuration marked in red, bipolar 2,3 and monopolar (blue). μCT (middle row), drill (turquois), measurement points marked green/red (go/stop) depending on the FNM assessment, facial nerve (yellow). CD: closest distance between drill and facial nerve in mm in each measurement point. Histology (top row) with H&E staining, Facial nerve assessment marked with a star. **(a–c)** Lateral trajectories with FN distance ≥ 0.2 mm; **(d–g)** Lateral trajectories with FN distance ≤ 0.1 mm; **(h–l)** Frontal trajectories with FN distance ≤ 0.1 mm.

In 49/56 points, the system correctly concluded it was uncritical to continue drilling. From the remaining seven points, four were correctly identified to be closer to the nerve than 0.1 mm (Figures 5i–l) and in three points the drill contacted the fallopian canal with no prior warning from the FNM (Figures 5e,g,h). All point along the three lateral trajectories with distances > 0.1 mm (Figures 5a–c) were correctly classified. Of the four lateral trajectories with distances < 0.1 mm to the facial nerve (Figures 5d–g), the FNM system missed to identify two critical trajectories (Figures 5e,g). Five trajectories approached the facial nerve frontally (Figures 5h–l). In three frontal trajectories the system recommended aborting the drill process before reaching the last point (Figures 5i–k). In one frontal trajectory the FNM detected critical nerve distance in the last measurement point (Figure 5l). In one frontal trajectory FNM suggested FN proximity < 0.1 mm based on B_2_ (intensity of 0.35 mA at P_3_) but missed to indicate it based on Bipolar 1.

### No Structural Damage to the Facial Nerve in Prospective Trajectories

**Figure 6:**
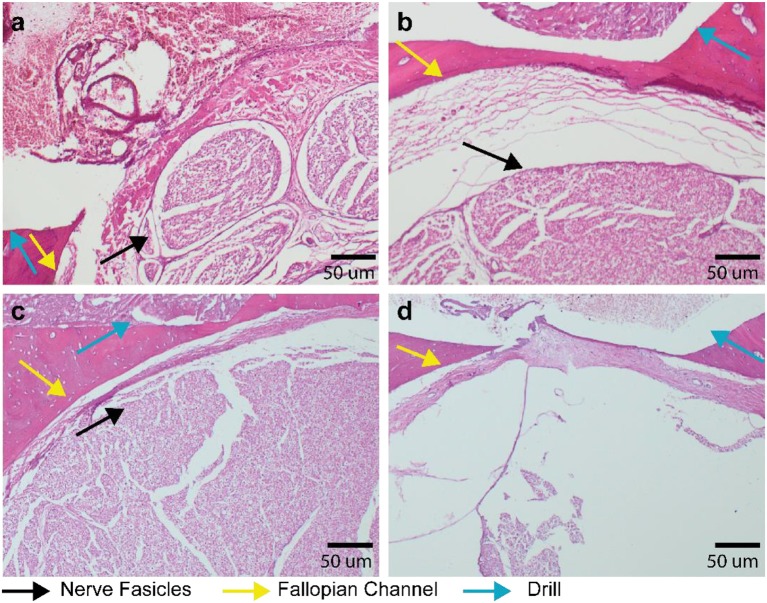
Histopathology of lateral trajectories at critically low distance to the fallopian channel (facial nerve canal). Decalcified mastoid bone slices with hematoxylin and eosin staining. **(a)** Trajectory 2.3–Facial nerve in close proximity to the drilled hole but without visible destruction of the nerve fascicles. **(b)** Trajectory 2.4 with 6 μm distance (measured in μCT) between drill and fallopian canal, the black arrow indicating intact facial nerve fascicles. **(c)** Trajectory 3.0 with 10 μm distance between drill and fallopian channel and with intact facial nerve fascicles. **(d)** Trajectory 3.4 with facial nerve not visible (due to a technical artefact) and an intact epineurium.

In the prospective trajectories, no structural damage to the facial nerve was observed in any of the histology slices for any of the drilled trajectories. Trajectories 2.5 and 3.5 (Figures 5h,k) were identified close to the facial nerve (distance < 0.1 mm) with hemorrhage and tissue debris in the trajectories. Contact to the epineurium surrounding the facial nerve was determined in trajectories 2.3, 2.5, 3.4, 3.5 (Figures 5e,g,h,k). No structural damage was observed to any of the nerve fascicles of the four lateral trajectories with distances < 0.1 mm (Figure 6). The complete histopathology analysis is in Supplemental Digital Content 3 (Supplementary Table 1), with each trajectory relative to the facial nerve and a region of interest in the facial nerve at a magnified scale.

### Stimulation Threshold as Function of Facial Nerve Distance

**Figure 7:**
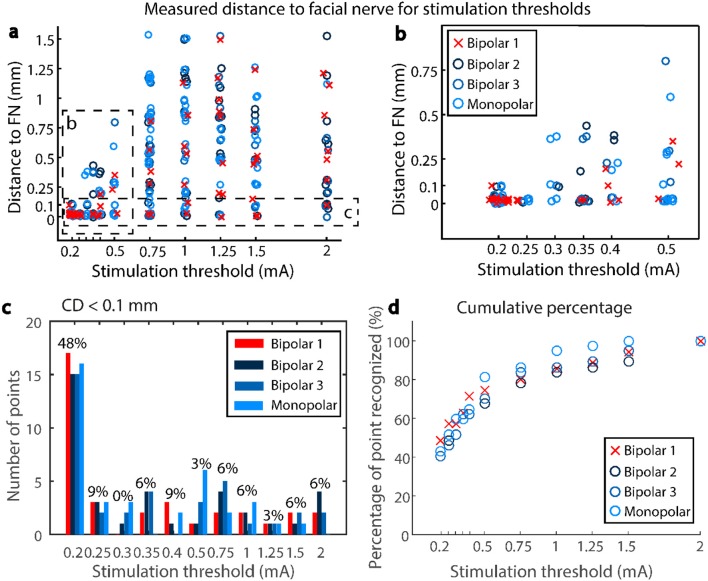
**(a)** Scatter plot of the stimulation thresholds and the corresponding measured distances to the facial nerve, for subjects 1,2, and 3. **(b)** Zoom into the lower stimulation thresholds, **(c)** number of points closer to the facial nerve than 0.1 mm which generate a CMAP at a given stimulus threshold, percentage only shown for Bipolar 1 **(d)** Cumulative percentage—percentage of points closer to the facial nerve than 0.1 mm recognized by a given stimulus threshold.

The measured distance to the facial nerve in function of the stimulation intensity (0.2–2 mA) showed a wide spread (0–1.5 mm; Figure 7a). In the lower stimulation intensities (0.2–0.5 mA), monopolar, bipolar 3, and bipolar 2 showed a wider spread of distances (up to 0.9 mm) compared to Bipolar 1 (up to 0.4 mm; Figure 7b). From the measurement points at facial nerve distances below 0.1 mm (37 points in all three subjects), 75% of the times the stimulus threshold was below 0.5 mA (configuration B1, Figure 7c). For the pre-defined threshold of 0.35 mA, 63% of the points were recognized by the system as distances below 0.1 mm. To reach 95% true positive detections at facial nerve distance < 0.1 mm, a stimulation intensity of 1.5 mA was needed (Figure 7d), but this would have resulted in a large amount of false positive detections (0.1 mm < FN distance < 1.5 mm, Figure 7a).

### FNM Performance to Determine Critical FN Proximity

Evaluation of the prospective use of the FNM system based on the histopathology resulted in a sensitivity of 0.57 (95% confidence interval (CI), [0.2, 0.88]) and a specificity of 1 (95% CI, [0.91, 1]). Providing the four measurement points which were not drilled (Figures 5i–k) would have been classified as critical distance (expected scenario), the sensitivity was 0.73 (95% CI, [0.39, 0.93]) and specificity 1 (95% CI, [0.91, 1]). From the retrospective assessment (Subject 1, 2, and 3), the sensitivity resulted in 0.68 (95% CI, [0.49, 0.84]) and specificity 0.97 (95% CI, [0.89, 0.99]) (*J* = 0.65). The positive predictive value was 0.91 reached at distances < 0.08 mm (95% CI, [0.69, 0.98]); and the negative predicted value was 0.88 at distances > 0.0 mm (95% CI, [0.78, 0.94]).

**Figure 8:**
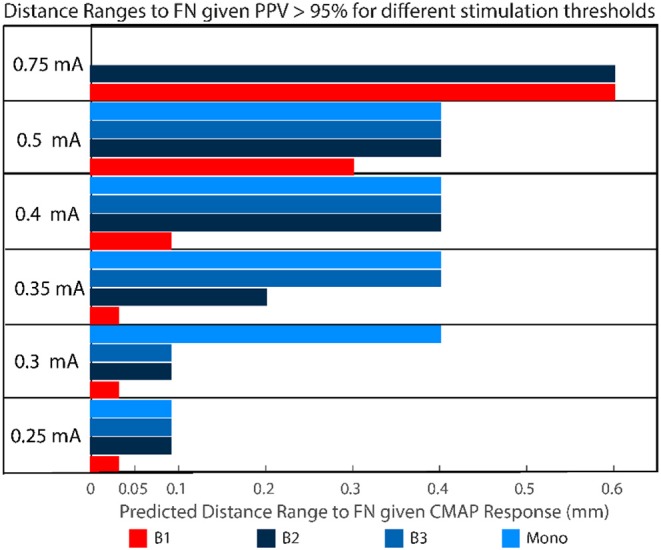
For different stimulation threshold levels, given a CMAP response >100 μV with a positive predictive value PPV > 95%, the trajectory will be found in the distance range depicted in colors. For example, with a positive response measured in B1 at 0.5 mA, the distance between the trajectory and the facial nerve is <0.3 mm.

Depending on the stimulation threshold, the system is able to discriminate different distance ranges to the facial nerve with a positive predictive value > 95% (Figure 8). Lower stimulation intensities resulted in narrow distance ranges, for example, in B1 configuration at 0.35 mA the drilled trajectories were within 0.03 mm from the facial nerve. Increasing stimulation intensities widened the distance ranges, for example at 0.5 mA (B1) the trajectories were within 0.3 mm from the facial nerve. Stimulation amplitude increased with distance for Bipolar configuration B1, whereas with bipolar B3 and monopolar almost no differences were found in the distance range < 0.6 mm.

## Discussion

In this study, we present a prospective validation of a previously proposed nerve monitoring approach to detect facial nerve proximity during robotic cochlear implantation procedure. Experimental results demonstrated that facial nerve monitoring can correctly warn the surgeon to stop robotic operation when the drill trajectory contacts the fallopian canal (facial nerve channel). Although the drill trajectory may have contacted the channel before the system has warned, structural preservation of the facial nerve is still guaranteed with the proposed nerve monitoring settings. Functional preservation of the facial nerve at the critical distance range <0.1 mm could not be assessed and remains unknown.

Histological assessment showed that in all prospective trajectories the system warned and stopped before the facial nerve was damaged. All measurement points which were outside the fallopian canal were correctly identified. In subject 1 (training trajectories), the nerve monitoring assessment correctly identified lateral trajectories that intersected and damaged the facial nerve (Figure 4b). In the prospective trajectories, in three measurement points the drill trajectory terminated inside the fallopian canal without previous warning of the FNM system (false negatives). While the system works well in most trajectories, there are borderline trajectories with distance below 0.1 mm when the drill approaches laterally the facial nerve channel which the system cannot yet correctly identify (Figure 6). Due to the prospective study set-up there was a limited number of measurement points classified as inside the nerve, which biases (underestimates) the sensitivity of the system compared to specificity (sensitivity 0.73, specificity 1).

The system had previously been used in a clinical trial (25) to prevent mechanical destruction of the facial nerve. In patients, a safety margin > 0.4 mm had been implemented (13, 25). Here distances between 0 and 0.4 mm were further studied to potentially decrease the safety margin defined in future clinical studies. In a previous pre-clinical *in vivo* study (24), we tested an initial version of the system and explored stimulation settings (intensities) and probe configurations (bipolar and monopolar). We found that 0.3 mA and bipolar stimulation (B_1_) could detect a transition into the nerve channel. Now, with a newly manufactured stimulation probe system, we found that below 0.35 mA (configuration B1) avoided structural damage of the facial nerve, but an intensity of 0.4 mA would potentially increase the sensitivity to detect critical proximity below 0.1 mm and is suggested for future studies (Figure 8).

The previous clinical study (25) suggested that the nerve monitoring information from configurations B2 and B3 may be redundant at distances > 0.4 mm. Here, we further suggest that even at the distance range < 0.4 mm, the configurations B2, B3, and monopolar suggest similar correlates of nerve distance for stimulation intensities below 0.5 mA (Figure 8). A probe-based measuring system allows to implement a large amount of stimulation settings, however only a limited number of points can be measured (due to time restrictions). Therefor we argue in the future the number of electrode configurations could be reduced from four to two, e.g., B1 and B2 or B1 and monopolar. Additionally, integrating the electrodes in the surgical drill bit will increase spatial resolution, save time and reduce the complexity for the surgeon.

Although the approach was able to ensure structural (anatomical) preservation of the facial nerve, here we did not evaluate a correlation between structural damage and functional damage. Thus, one practical limitation of our study is that only structural damage could be assessed with microcomputed tomography or histopathology. Standard assessment of facial nerve function in an animal model may result cumbersome and ethically inappropriate. We have applied functional testing during our first clinical study in patients and it requires awake state and collaboration of the patients before and after the operation (30).

An alternative to assess functional nerve damage in an animal model is recording a positive control stimulation channel at the brainstem level before the facial nerve mastoid region. Changes in amplitude of the evoked muscle action potentials at supramaximal stimulation intensity is correlated to intraoperative iatrogenic nerve damage (e.g., <50%) (33). The viability of this method to test nerve integrity in the mastoid region has not yet been studied, and its efficacy in our animal model set-up is still unknown. Due to the “somatotopic” distribution of the facial nerve fibers (34), in future studies we suggest using a larger number of EMG needles to be able to precisely assess minimal changes in integrity of the nerve.

The criteria for stopping the robotic drilling is based on a pre-defined stimulation intensity and EMG-triggered response, but the surgeon still needs to decide. This is especially relevant in border-line cases, e.g., when the EMG signal is contaminated with ambient noise or interference, or when the peak EMG value is close but not yet above threshold level. In the operating room, the final decision of the surgeon is based on a combination of nerve monitoring with the primary layers of safety i.e., accuracy of the registration process and direction of drilling error relative to the plan based on intraoperative imaging before the facial nerve (12).

The nerve monitoring measuring set-up uses a simplified model with two variables (stimulation intensity and muscle evoke potentials amplitude) to derive facial nerve distance. This simplified model ignores anatomical and bone quality properties which do influence the physical path of the electrical current (volume conductor). We assume the unexpected high stimulation intensities in some of the measured points (e.g., Figures 5f,g) are due to such anatomical and structural differences. In the future, tissue impedance and computational modeling from pre-operative computed tomography images (30, 35) could be investigated to calibrate intensity thresholds at the facial nerve canal, specific to each patient.

To conclude, facial nerve monitoring integrated in a surgical robotic system supported the surgeon to enable facial nerve structural preservation prospectively. Although structural preservation is a primary requirement during robotic drilling in the mastoid, it remains unknown if functional nerve integrity is ensured within distances of 0.1 mm or below. The system and approach are specific to not give false alarms in case of trajectories being drilled within the planned safety distance margin (>0.4 mm); and the system proved to correctly warn before the drill could structurally damage the facial nerve in frontal trajectories. Lateral distances below 0.1 mm cannot always be discriminated and should be avoided.

## Data Availability Statement

Data sets of this study are made available by the authors (see Supplementary Materials: Data Sheet 1, Table 1). Requests to access additional datasets should be directed to juan.anso.research@gmail.com.

## Ethics Statement

Full name of the ethics committee that approved the study: Bernese cantonal animal commission. https://www.vol.be.ch/vol/de/index/direktion/organisation/lanat/organigramm/kommission_fuer_tierversuche.html.

## Author Contributions

JA contributed in study design, experiments, data analysis, statistics, and manuscript writing. CD contributed in study design, experiments, data analysis, manuscript writing, and reviewing. MA contributed in data analysis, statistics, manuscript writing, and reviewing. FV contributed in study design and experiments. OS, KS, HR, and FF contributed in study design, experiments, and manuscript reviewing. MD, IZ, and KW contributed in histopathology analysis. MM and MZ-A contributed in study design, system implementation, and manuscript reviewing. MH and MC contributed in manuscript reviewing. SW contributed in study design, manuscript writing, and reviewing.

### Conflict of Interest

JA and SW were inventors on the related patents PCT/EP2018/067648 and PCT/IB2017/055312. SW was cofounder, shareholder, and advisor to the board. MM was chief technical officer and shareholder of CAScination AG (Bern, Switzerland), a company that is developing the robotic cochlear implantation technology presented herein. The remaining authors declare that the research was conducted in the absence of any commercial or financial relationships that could be construed as a potential conflict of interest.

## Supplementary Material

Supplementary Data Sheet 1 Overview of recorded electromyography data showing CMAP responses to the stimulation intensity ramp at each measurement point for the monopolar stimulation. A graph with maximum CMAP responses of monopolar stimulation for each trajectory is depicted. A Summary report (Subject 1, 2, 3.docx) of CMAP responses (for monopolar stimulation) in trajectories with potential FN damage are presented. Data sets of bipolar stimulation can be shared if the reader is interested (see Data Availability Statement).

Supplementary Table 1 Complete histopathology analysis with each trajectory relative to the facial nerve and a region of interest in the facial nerve at a magnified scale.

Supplementary Video 1 Experimental robotic drilling and FNM set-up. The robotic system drills to next measurement point, the otologic surgeon (user) inserts the FNM probe. The stimulation ramp is applied and CMAP responses of the subjects facial muscles are recorded. This protocol is reaped for each FNM measurement point in the trajectory. If FNM system warns of a distance to the FN below 0.1 mm, drilling of subsequent points of the trajectory is aborted.
