# Supplementary material for: Prospective Validation of Facial Nerve Monitoring to Prevent Nerve Damage During Robotic Drilling
Source: Front Surg. 2019 Oct 1;6:58. doi: 10.3389/fsurg.2019.00058 (PMC6781655; doi:10.3389/fsurg.2019.00058)
Supplement: Supplementary Data Sheet 1 — Overview of recorded electromyography data showing CMAP responses to the stimulation intensity ramp at each measurement point for the monopolar stimulation. A graph with maximum CMAP responses of monopolar stimulation for each trajectory is depicted. A Summary report (Subject 1, 2, 3.docx) of CMAP responses (for monopolar stimulation) in trajectories with potential FN damage are presented. Data sets of bipolar stimulation can be shared if the reader is interested (see Data Availability Statement). [file Data_Sheet_1.ZIP › Analysis_EMG_Amplitude_Changes/Subject 1.docx]

**Trajectory 1.0**

| 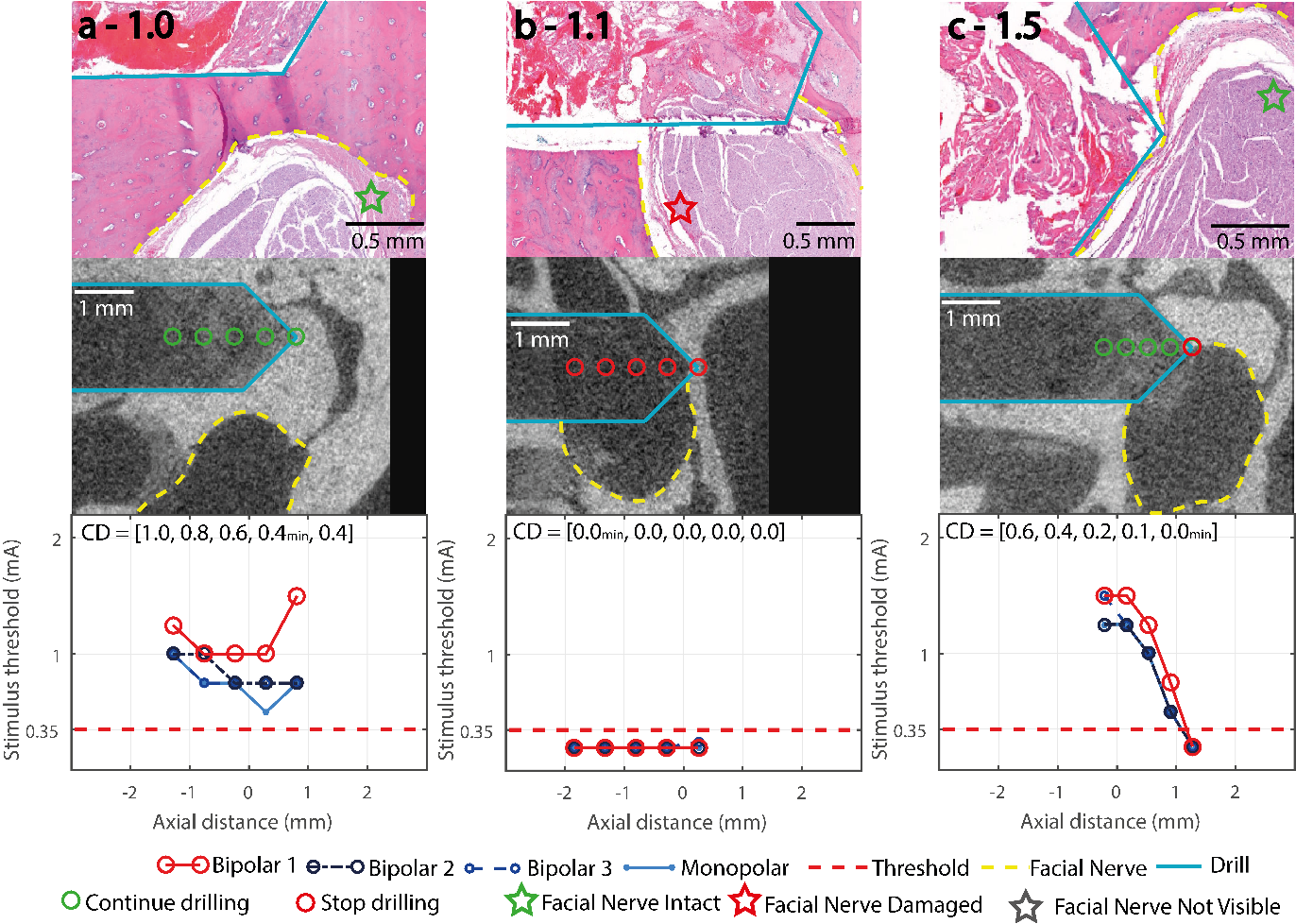 | | | | 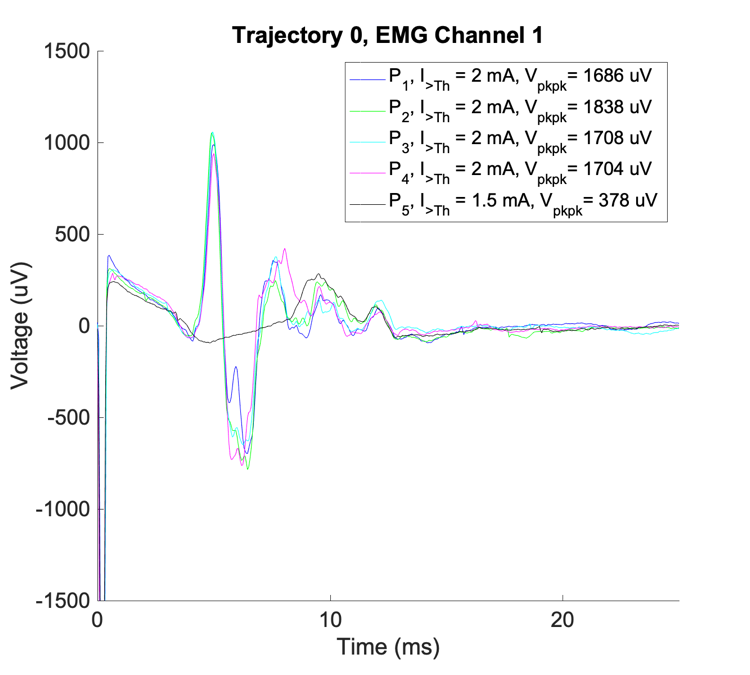  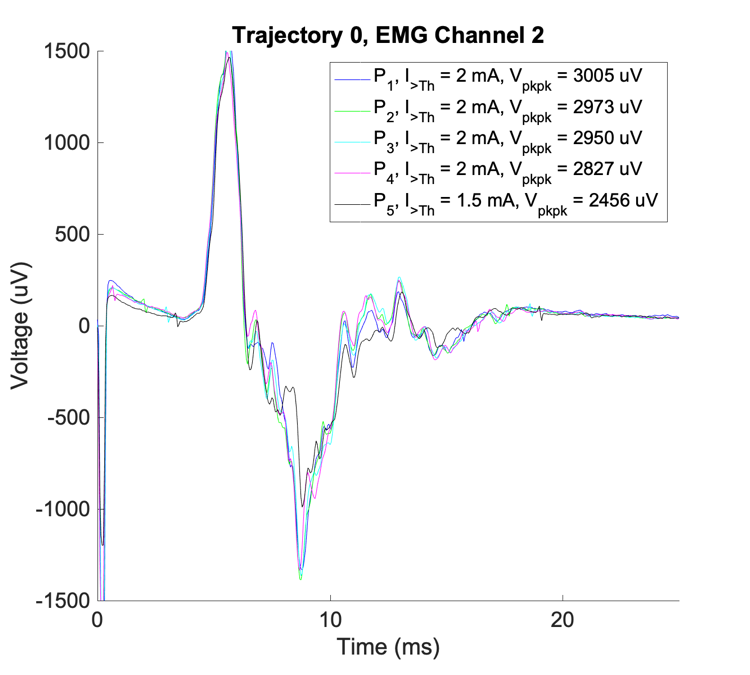 | | |
| --- | --- | --- | --- | --- | --- | --- |
| **Comments**  It’s not expected damage to the facial nerve in this trajectory. EMG amplitude decreased over 50% in the last measuring point (EMG Ch1). Our hypothesis here is that this change is not indicating nerve functional damage. It only happens in Ch1. This may indicate a large distance from the stimulating tip to the nerve fibers innervating Ch1. These fibers may be located at the other side of the nerve’s cross section (Trajectories 1,2,3). | | | | 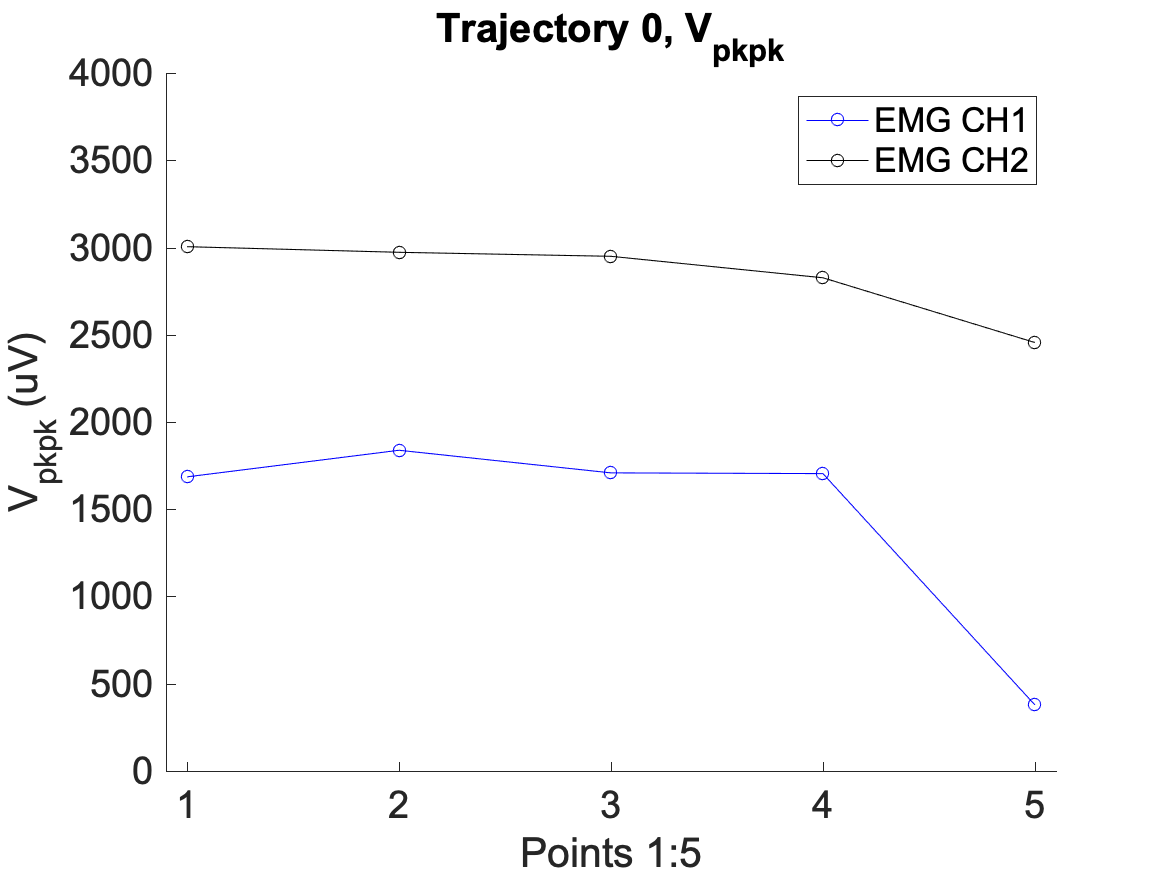 | | |
| % EMG change | P1 | P2 | P3 | | P4 | P5 |
| EMG Ch1 | +0% | +9% | -7% | | -0.2% | -77%* (no supramaximal) |
| EMG Ch2 | +0% | -1% | +0.7% | | -4% | -13% |

**Trajectory 1.1**

| 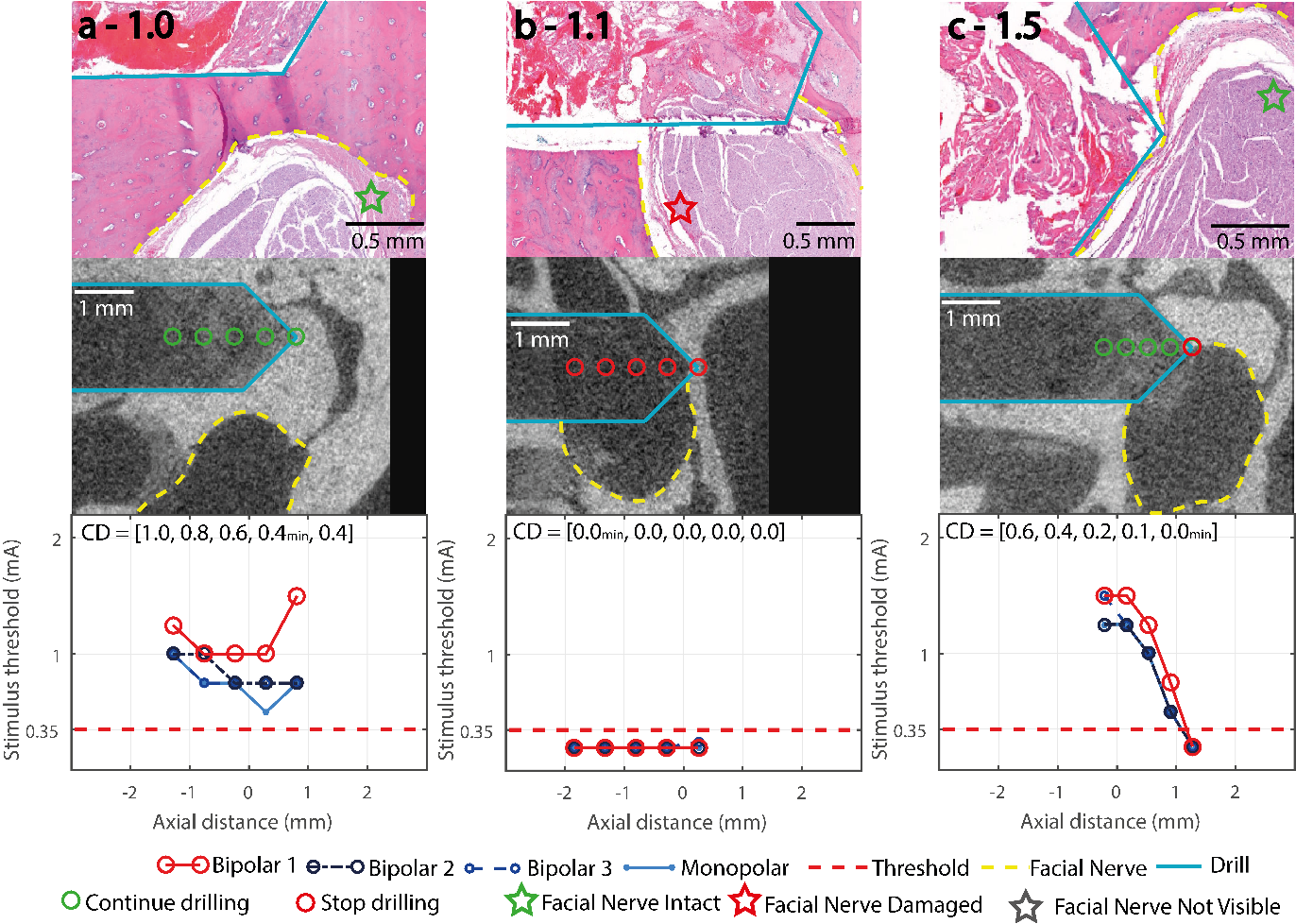 | | | 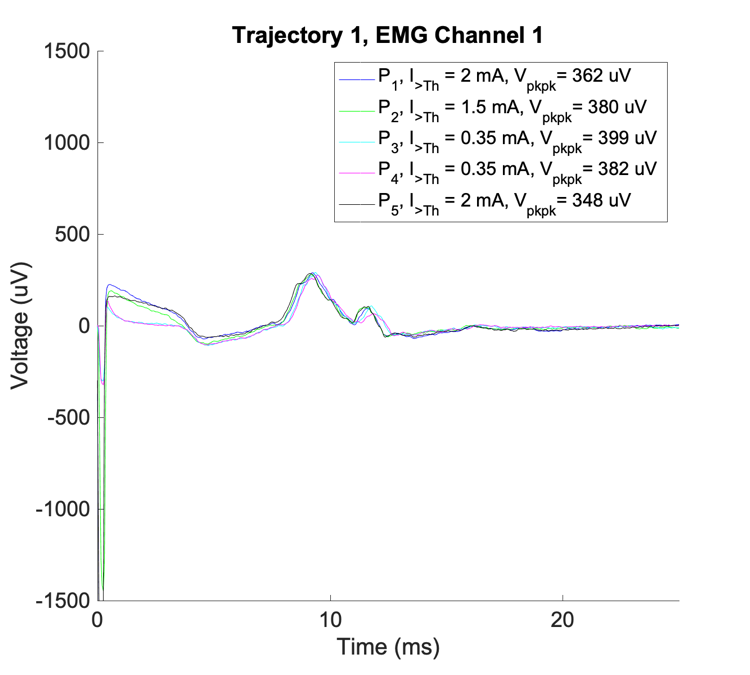  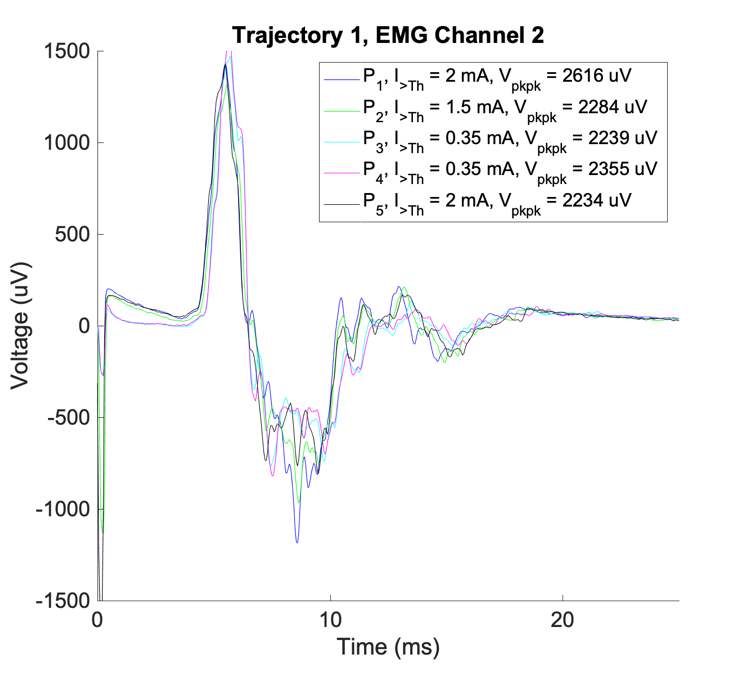 | | | |
| --- | --- | --- | --- | --- | --- | --- |
| **Comments**  It’s expected functional damage to the nerve in this trajectory.  EMG in channel 1 decreased (<50%) with respect to EMG amplitude of CH1 of trajectory 1.0 (see above).  This change could indicate facial nerve integrity change compared to the start of the procedure.  There is no relative change in amplitude as the drill further enters the facial nerve. | | | 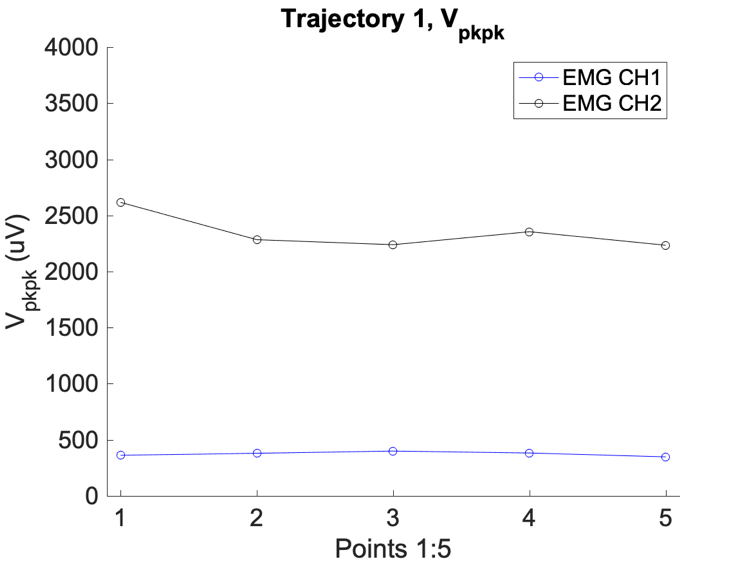 | | | |
| % EMG change | P1 | P2 | | P3 | P4 | P5 |
| EMG Ch1 | +0% | +0% | | +5% | -4% | **-**1% |
| EMG Ch2 | +0% | -11% | | +6% | +5% | -8% |
