# Supplementary material for: Prospective Validation of Facial Nerve Monitoring to Prevent Nerve Damage During Robotic Drilling
Source: Front Surg. 2019 Oct 1;6:58. doi: 10.3389/fsurg.2019.00058 (PMC6781655; doi:10.3389/fsurg.2019.00058)
Supplement: Supplementary Data Sheet 1 — Overview of recorded electromyography data showing CMAP responses to the stimulation intensity ramp at each measurement point for the monopolar stimulation. A graph with maximum CMAP responses of monopolar stimulation for each trajectory is depicted. A Summary report (Subject 1, 2, 3.docx) of CMAP responses (for monopolar stimulation) in trajectories with potential FN damage are presented. Data sets of bipolar stimulation can be shared if the reader is interested (see Data Availability Statement). [file Data_Sheet_1.ZIP › Analysis_EMG_Amplitude_Changes/Subject 3.docx]

**Trajectory 3.1**

| 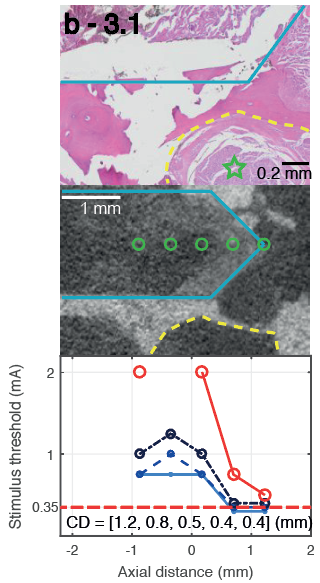 | | | **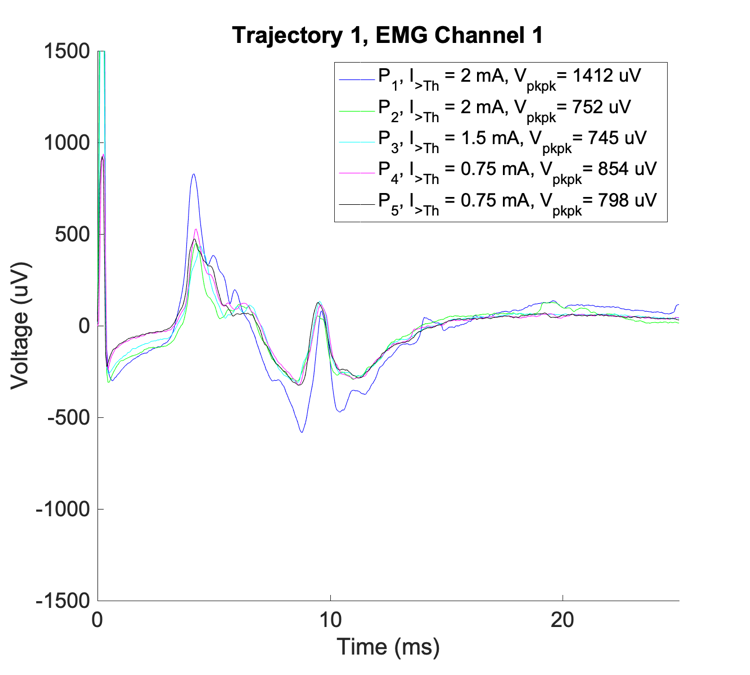**  **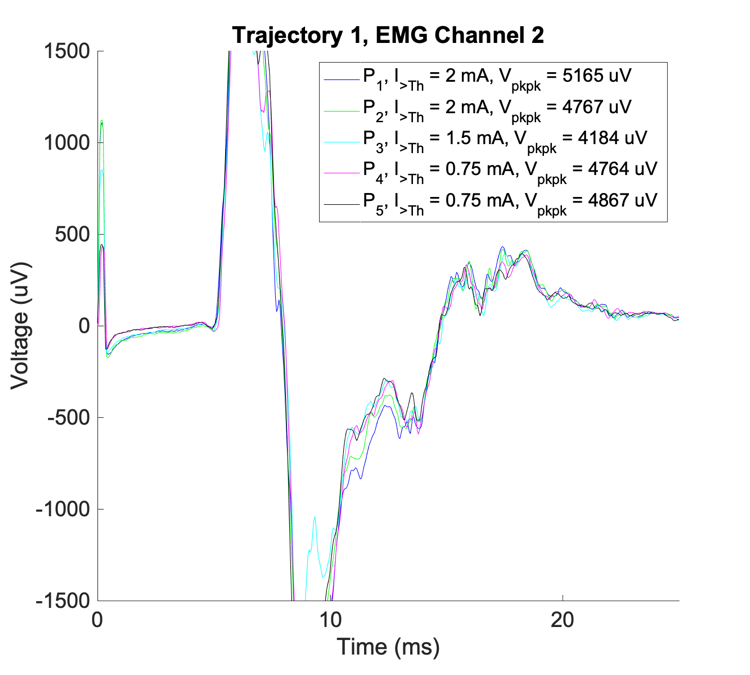** | | | |
| --- | --- | --- | --- | --- | --- | --- |
| **Comments**  This trajectory, although no structural damage is observed.  EMG amplitude of Ch2 decreases to 30% in the last measuring point P5.  At the last measuring point the stimulation threshold in Bipolar 1 dropped from 1 to 0.4 mA, indicating close nerve distance.  We don’t know if integrity of the nerve may have been changed given the dropped to -29% pk-pk amplitude. | | | 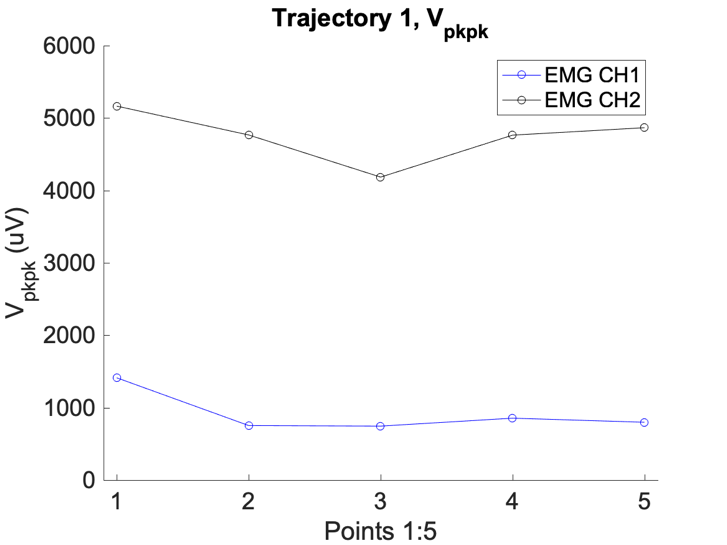 | | | |
| % EMG change | P1 | P2 | | P3 | P4 | P5 |
| EMG Ch1 | +0% | -47% | | -1% | +15% | -7% |
| EMG Ch2 | +0% | -8% | | -12% | +14% | 2% |

**Trajectory 3.0**

| 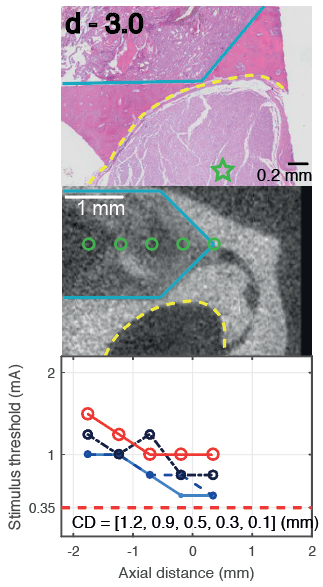 | | | **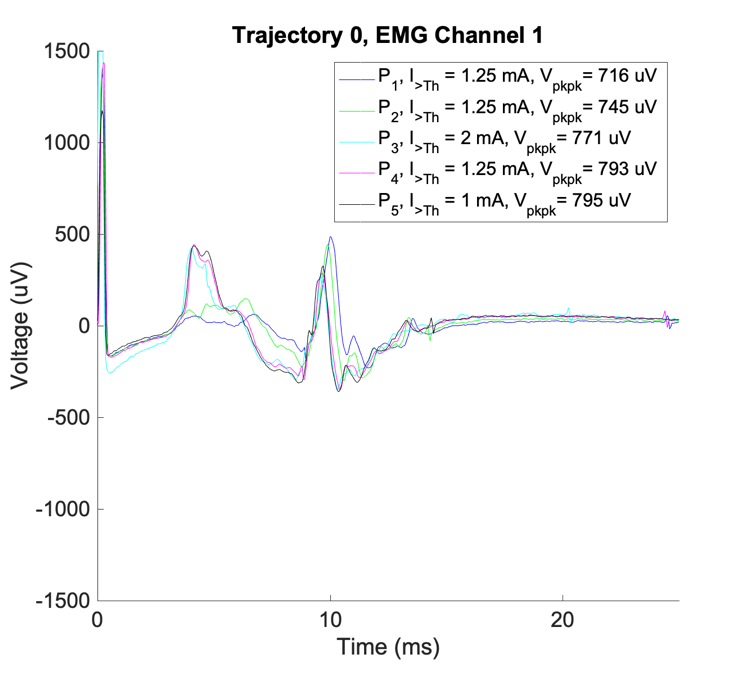**  **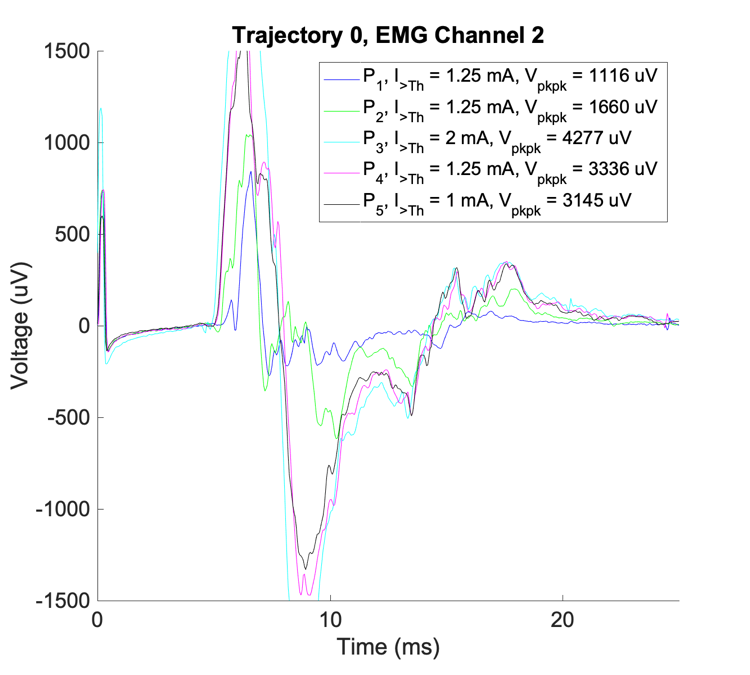** | | | |
| --- | --- | --- | --- | --- | --- | --- |
| **Comments**  This lateral trajectory finishes at the minimum distance plane, at a distance of 0.1 mm to the facial nerve channel.  EMG amplitude in CH2 presented first a large increase of +158% at P3. Then, a decrease of **-22%** and -8% at P4, P5.  P4, P5 corresponded to a minimum distance of (P4) 0.3 mm, (P5) 0.1 mm. | | | **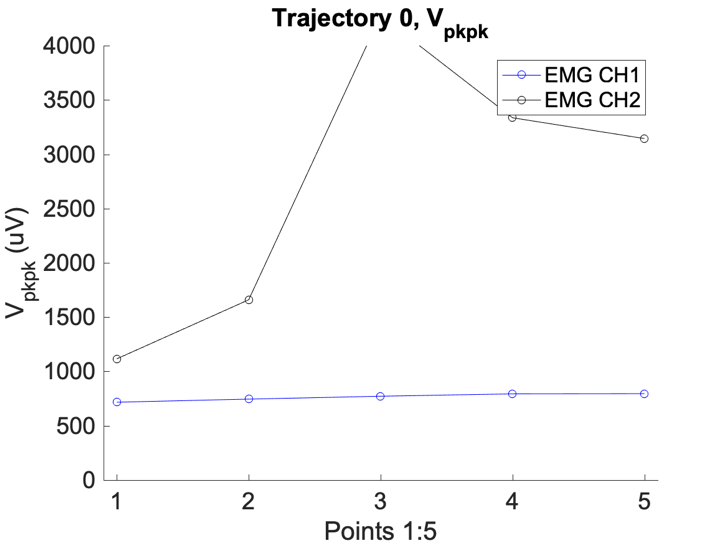** | | | |
| % EMG change | P1 | P2 | | P3 | P4 | P5 |
| EMG Ch1 | +0% | -4% | | +4% | +3% | +0% |
| EMG Ch2 | +0% | +49% | | +158% | **-22%** | -6% |

**Trajectory 3.4**

| 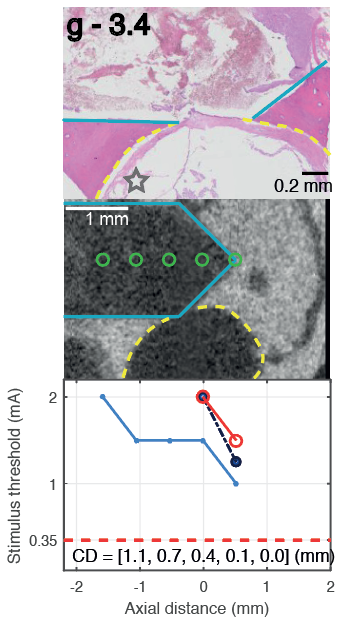 | | | 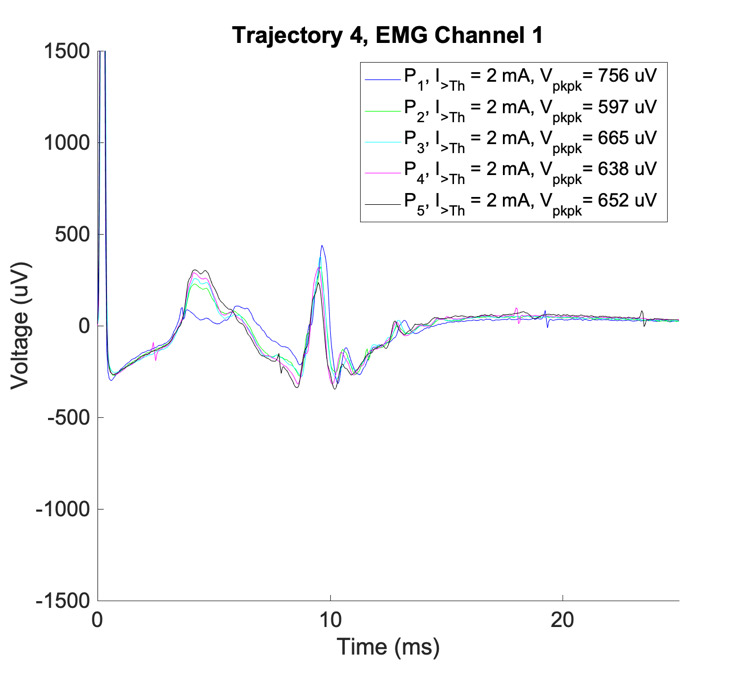  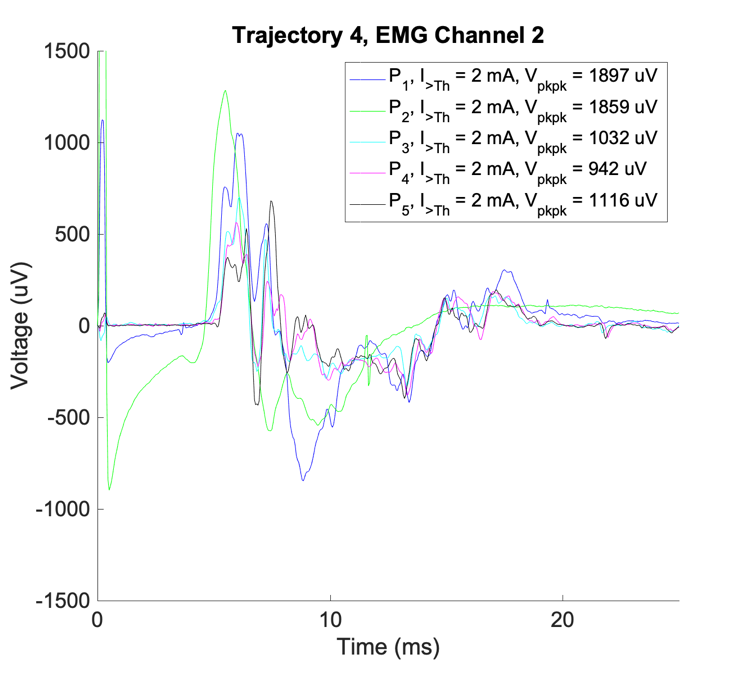 | | | |
| --- | --- | --- | --- | --- | --- | --- |
| **Comments**  This trajectory contacted the facial nerve channel (0.0 mm) at the last point P5. EMG amplitude in CH2 decreases -44% at the transition from P2 to P3. These points correspond to a distance transition from 0.7 to 0.4 mm.  In the next transition, from P3 to P4, the distance is reduced from 0.4 to 0.1 mm: additional EMG decrease of -9%. | | | 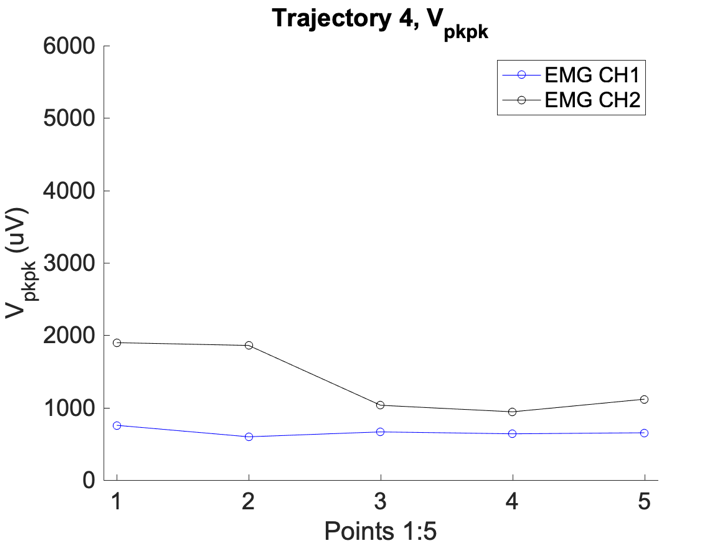 | | | |
| % EMG change | P1 | P2 | | P3 | P4 | P5 |
| EMG Ch1 | +0% | -21% | | +11% | -4% | +2% |
| EMG Ch2 | +0% | -2% | | -44% | -9% | +18% |

**Trajectory 3.2**

| 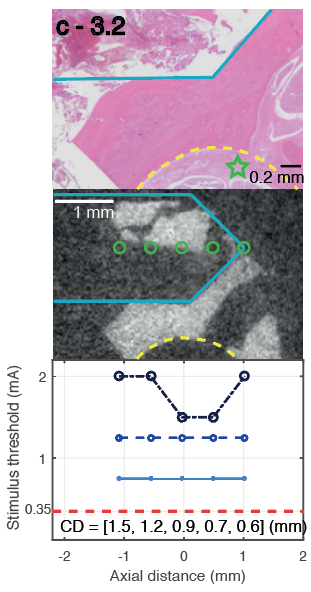 | | | 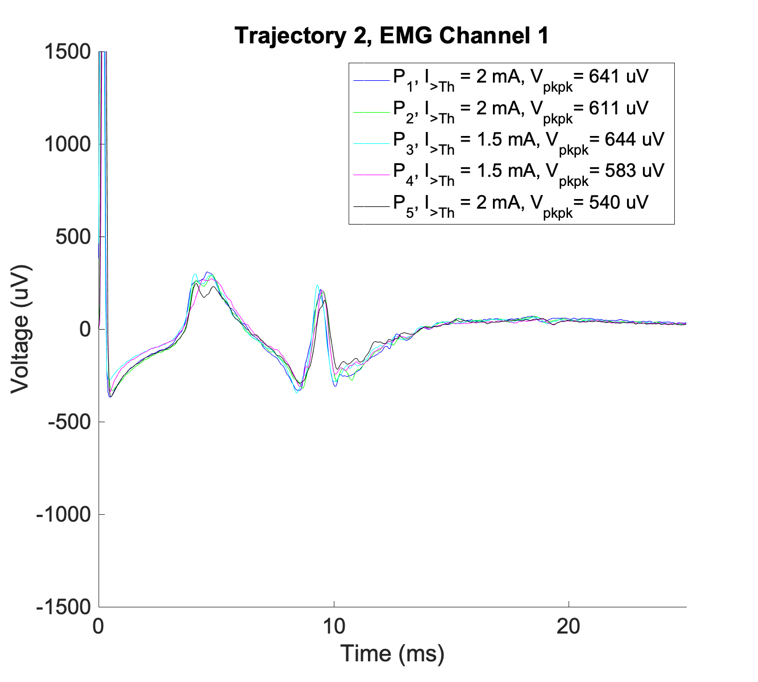  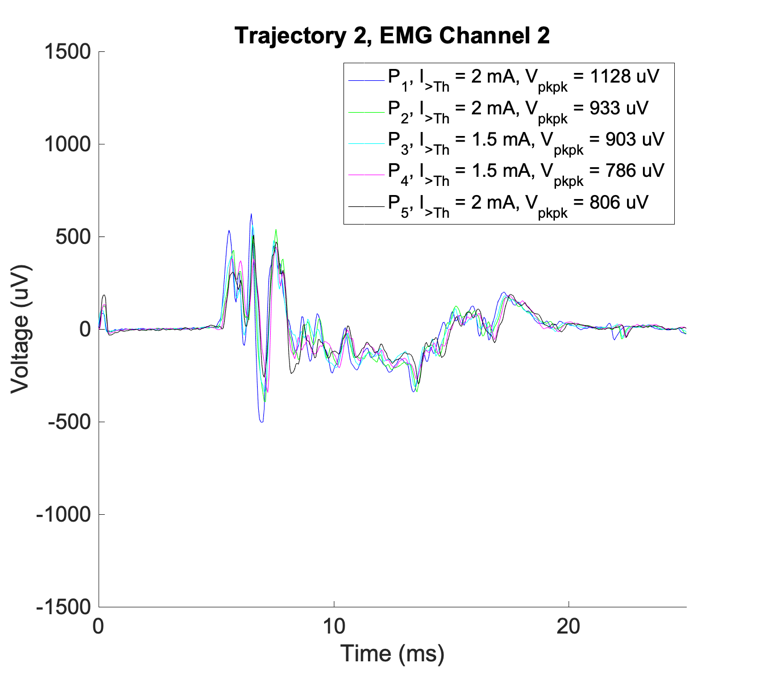 | | | |
| --- | --- | --- | --- | --- | --- | --- |
| **Comments**  This trajectory passes within 0.6 mm to the facial nerve channel.  EMG amplitude remains within 20% changes, no large fluctuation. | | | 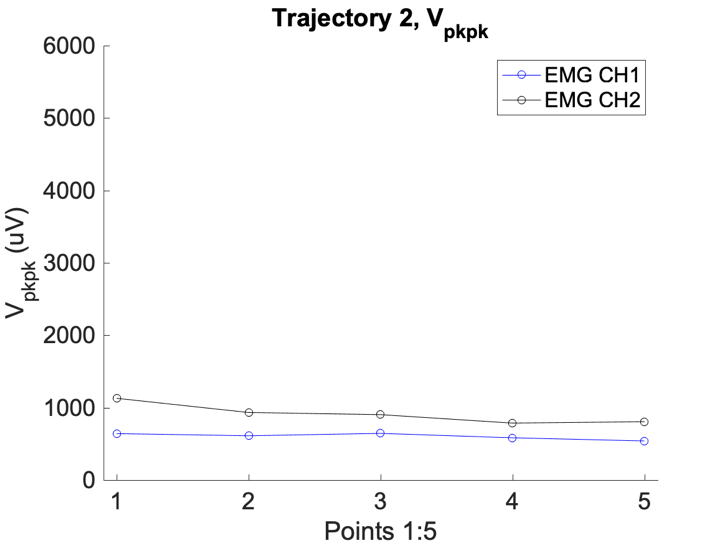 | | | |
| % EMG change | P1 | P2 | | P3 | P4 | P5 |
| EMG Ch1 | +0% | -5% | | +5% | -9% | -7% |
| EMG Ch2 | +0% | -17% | | -3% | -13% | +2% |
