# Supplementary material for: Prospective Validation of Facial Nerve Monitoring to Prevent Nerve Damage During Robotic Drilling
Source: Front Surg. 2019 Oct 1;6:58. doi: 10.3389/fsurg.2019.00058 (PMC6781655; doi:10.3389/fsurg.2019.00058)
Supplement: Supplementary Data Sheet 1 — Overview of recorded electromyography data showing CMAP responses to the stimulation intensity ramp at each measurement point for the monopolar stimulation. A graph with maximum CMAP responses of monopolar stimulation for each trajectory is depicted. A Summary report (Subject 1, 2, 3.docx) of CMAP responses (for monopolar stimulation) in trajectories with potential FN damage are presented. Data sets of bipolar stimulation can be shared if the reader is interested (see Data Availability Statement). [file Data_Sheet_1.ZIP › Analysis_EMG_Amplitude_Changes/Subject 2.docx]

**Trajectory 2.3**

| 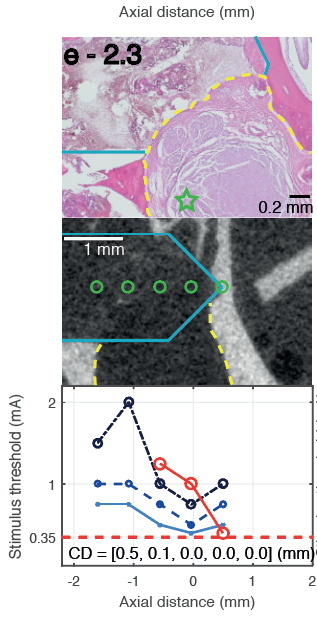 | | | **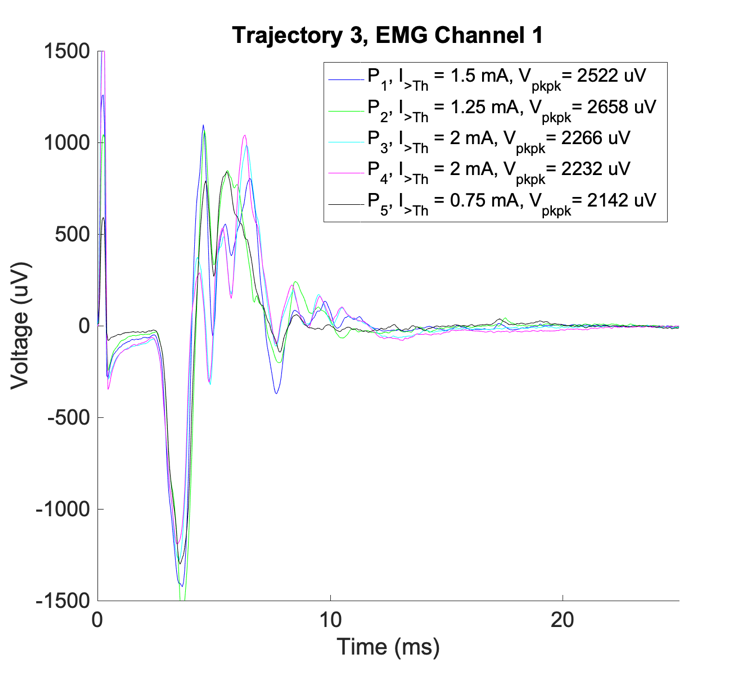**  **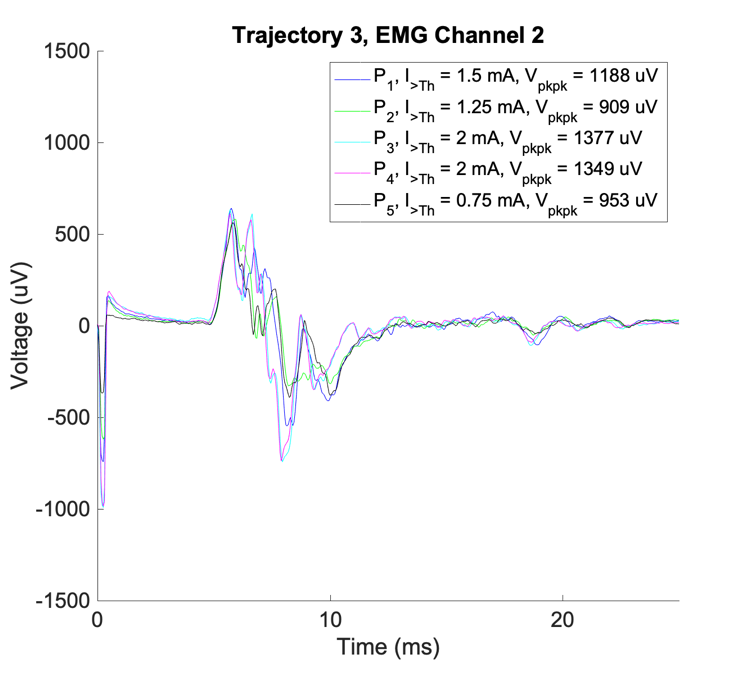** | | | |
| --- | --- | --- | --- | --- | --- | --- |
| **Comments**  This trajectory, although no structural damage is observed.  EMG amplitude of Ch2 decreases to 30% in the last measuring point P5.  At the last measuring point the stimulation threshold in Bipolar 1 dropped from 1 to 0.4 mA, indicating close nerve distance.  We don’t know if integrity of the nerve may have been changed given the dropped to -29% pk-pk amplitude. | | | **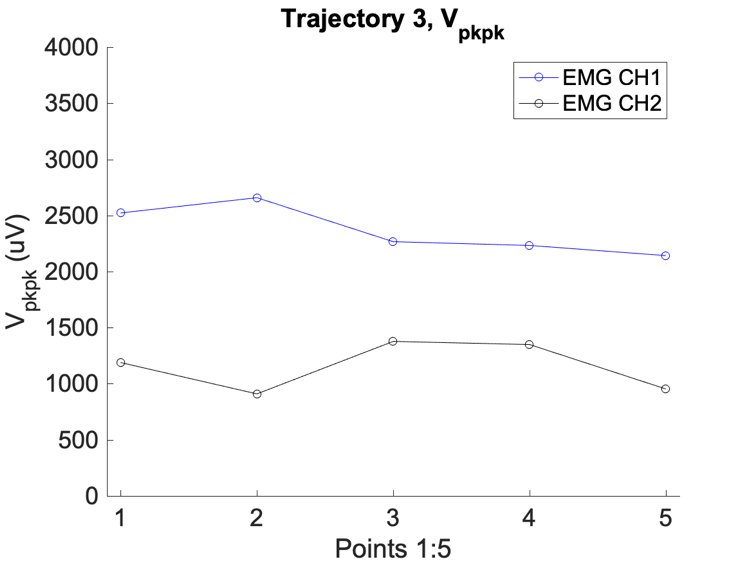** | | | |
| % EMG change | P1 | P2 | | P3 | P4 | P5 |
| EMG Ch1 | +0% | +5% | | -15% | -1% | -4% |
| EMG Ch2 | +0% | -24% | | +51% | -2% | -29% |

**Trajectory 2.4**

| 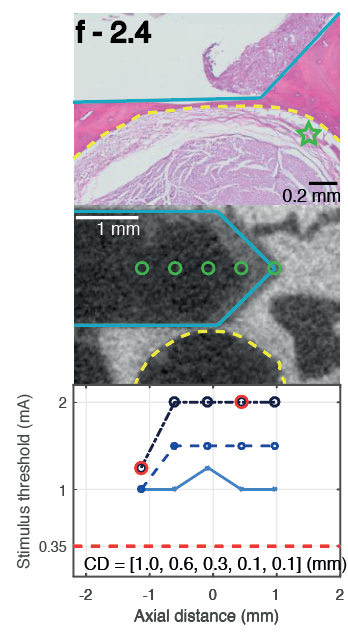 | | | 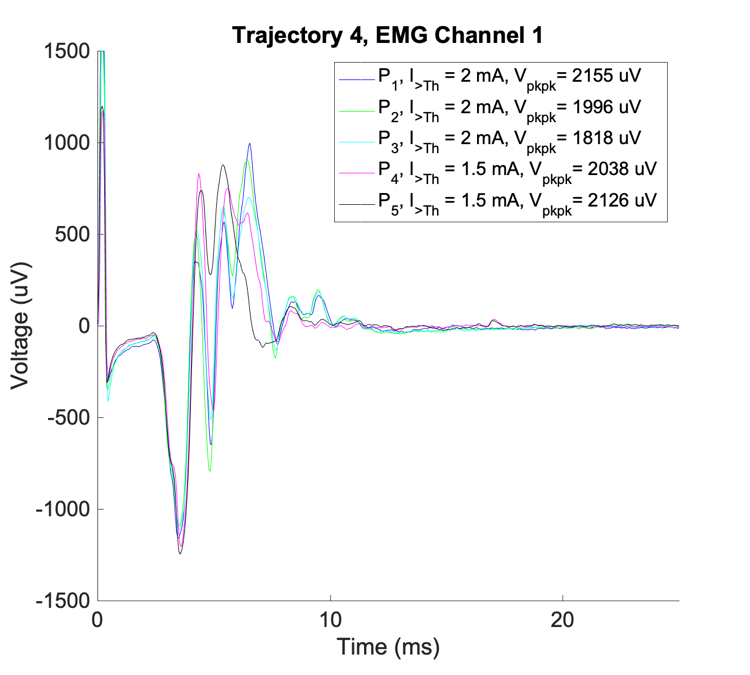  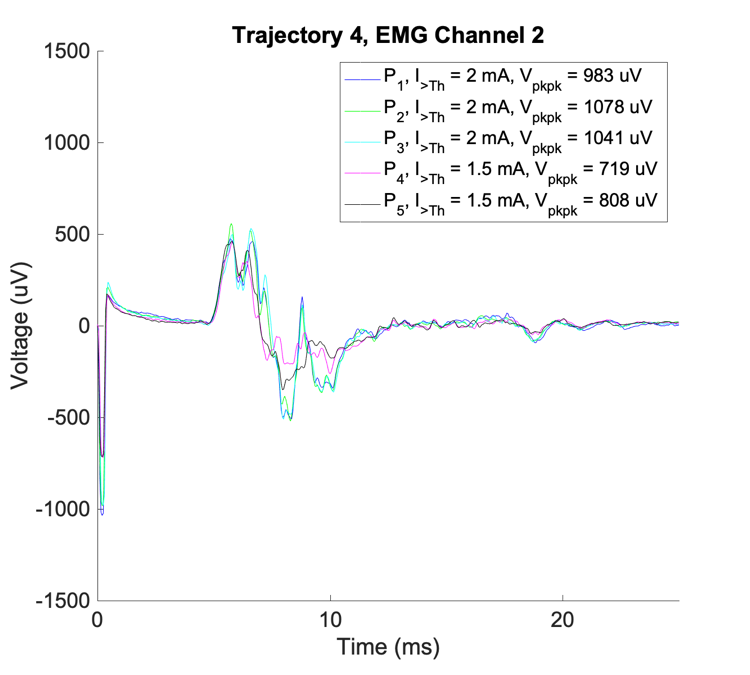 | | | |
| --- | --- | --- | --- | --- | --- | --- |
| **Comments**  This trajectory passes within 0.1 mm to the facial nerve channel.  EMG amplitude in CH2 decreases 31% between Points 3 and 4. These points correspond to the transition to minimum lateral distance of 0.1 mm.  In the last measuring point (P5), the relative change to the previous point is positive +12%. | | | 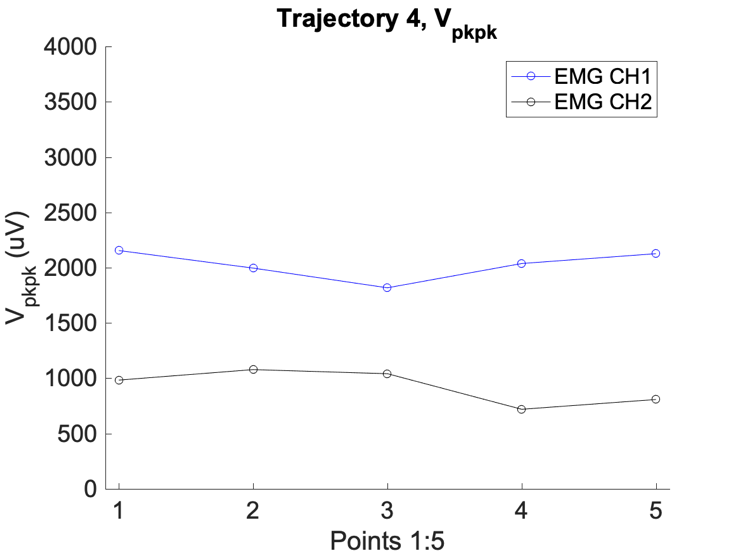 | | | |
| % EMG change | P1 | P2 | | P3 | P4 | P5 |
| EMG Ch1 | +0% | -7% | | -9% | +12% | +4% |
| EMG Ch2 | +0% | +10% | | -3% | -31% | +12% |

**Trajectory 2.4**

| 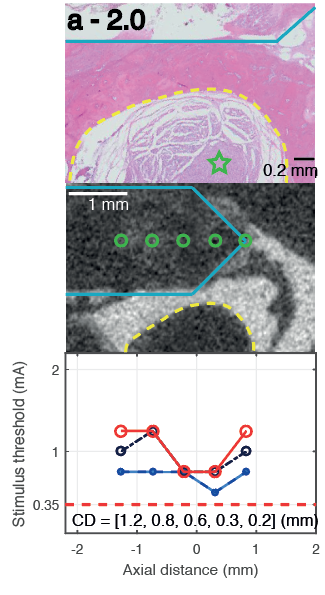 | | | 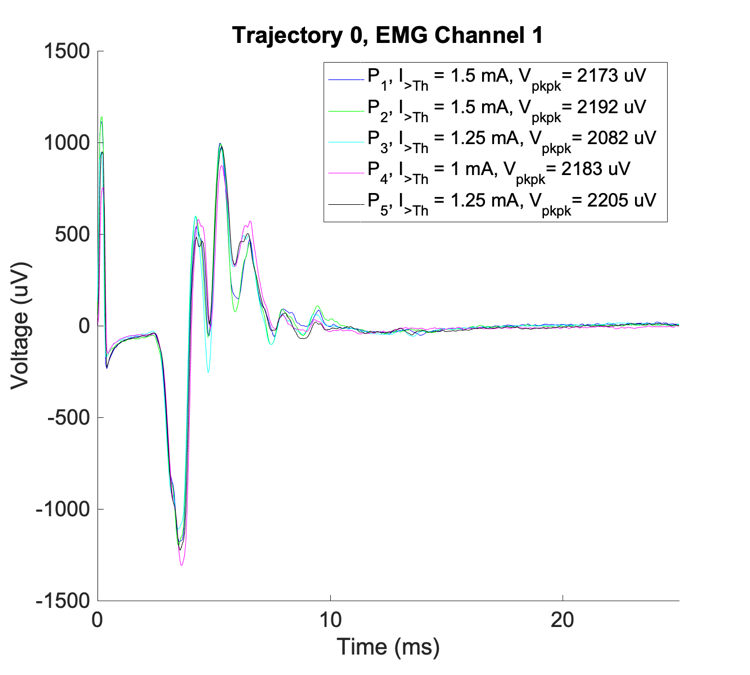  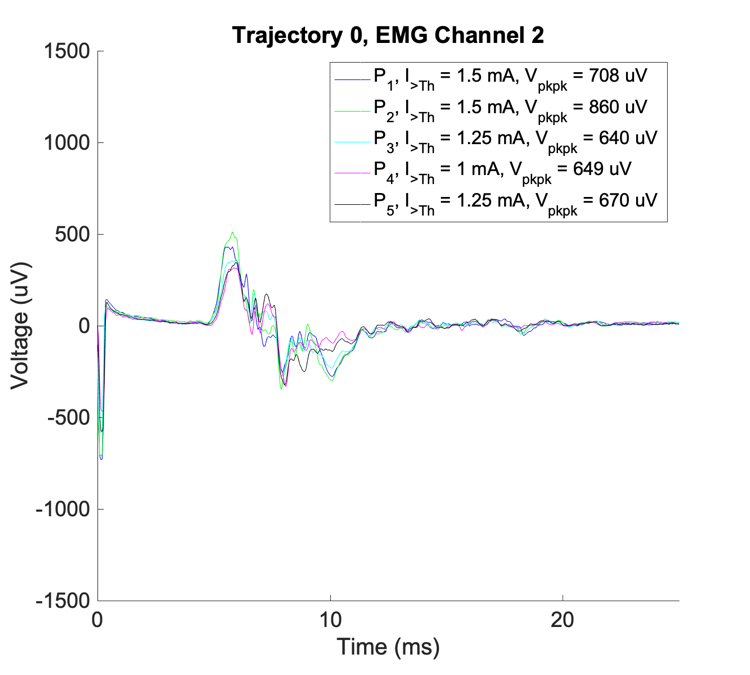 | | | |
| --- | --- | --- | --- | --- | --- | --- |
| **Comments**  This trajectory passes within 0.1 mm to the facial nerve channel.  EMG amplitude in CH2 decreases 31% between Points 3 and 4. These points correspond to the transition to minimum lateral distance of 0.1 mm.  In the last measuring point (P5), the relative change to the previous point is positive +12%. | | | 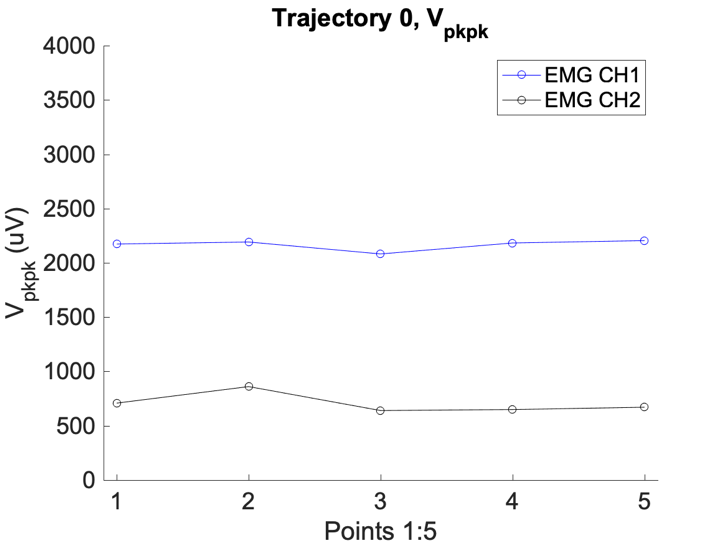 | | | |
| % EMG change | P1 | P2 | | P3 | P4 | P5 |
| EMG Ch1 | +0% | +1% | | -5% | +5% | +1% |
| EMG Ch2 | +0% | +21% | | -26% | +1% | +3% |
