# Supplementary figures and images for: Prospective Validation of Facial Nerve Monitoring to Prevent Nerve Damage During Robotic Drilling (part 1 of 2)
Source: Front Surg. 2019 Oct 1;6:58. doi: 10.3389/fsurg.2019.00058 (PMC6781655; doi:10.3389/fsurg.2019.00058)

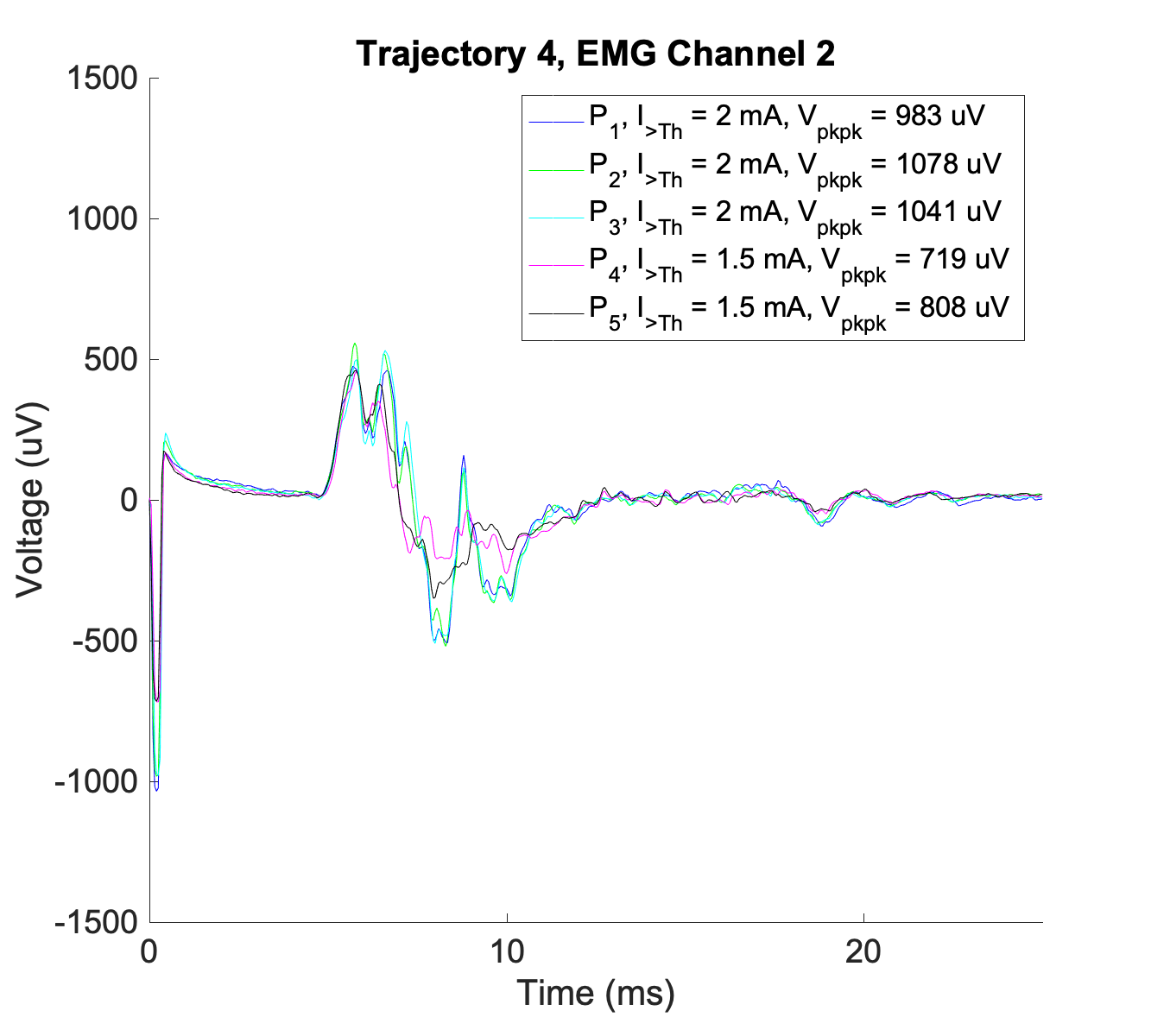

Supplement: Supplementary Data Sheet 1 — Overview of recorded electromyography data showing CMAP responses to the stimulation intensity ramp at each measurement point for the monopolar stimulation. A graph with maximum CMAP responses of monopolar stimulation for each trajectory is depicted. A Summary report (Subject 1, 2, 3.docx) of CMAP responses (for monopolar stimulation) in trajectories with potential FN damage are presented. Data sets of bipolar stimulation can be shared if the reader is interested (see Data Availability Statement). [file Data_Sheet_1.ZIP › Analysis_EMG_Amplitude_Changes/EMGAmp_OutputData/Subject2/Subject2_Traj4_AllPoints_EMG_CH2.png]

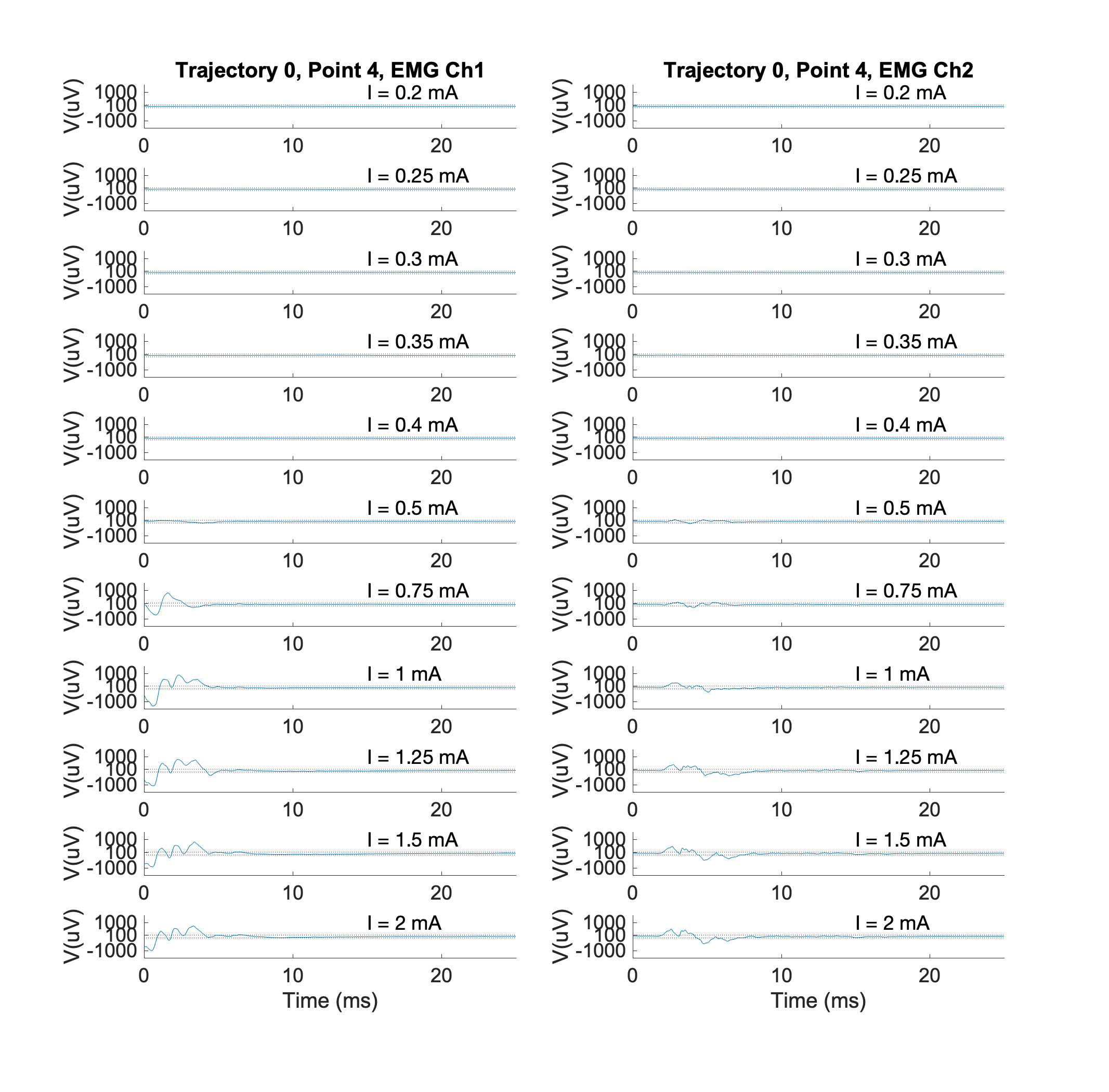

Supplement: Supplementary Data Sheet 1 — Overview of recorded electromyography data showing CMAP responses to the stimulation intensity ramp at each measurement point for the monopolar stimulation. A graph with maximum CMAP responses of monopolar stimulation for each trajectory is depicted. A Summary report (Subject 1, 2, 3.docx) of CMAP responses (for monopolar stimulation) in trajectories with potential FN damage are presented. Data sets of bipolar stimulation can be shared if the reader is interested (see Data Availability Statement). [file Data_Sheet_1.ZIP › Analysis_EMG_Amplitude_Changes/EMGAmp_OutputData/Subject2/Subject2_Traj0_Point4_EMGepochs.png]

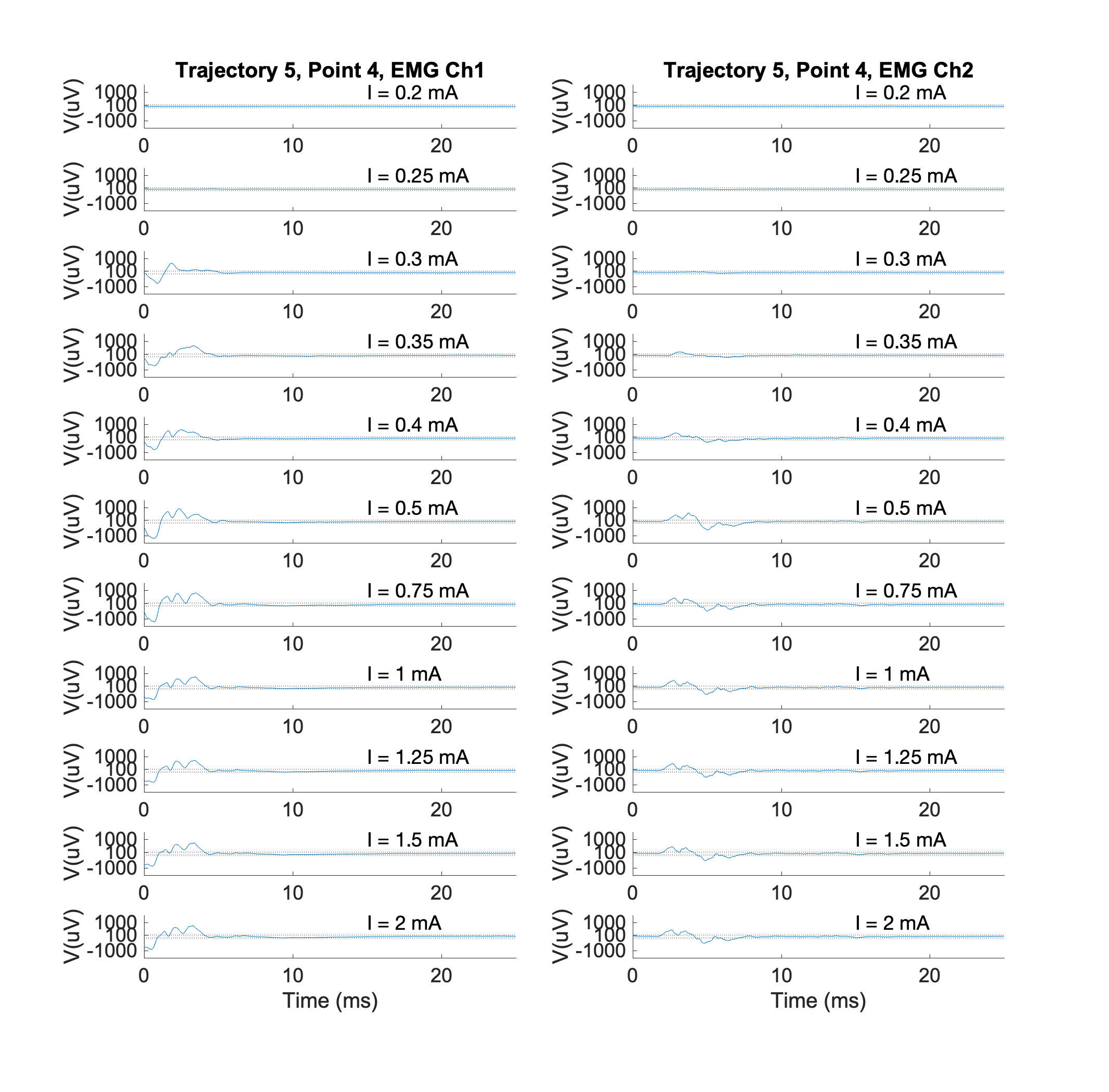

Supplement: Supplementary Data Sheet 1 — Overview of recorded electromyography data showing CMAP responses to the stimulation intensity ramp at each measurement point for the monopolar stimulation. A graph with maximum CMAP responses of monopolar stimulation for each trajectory is depicted. A Summary report (Subject 1, 2, 3.docx) of CMAP responses (for monopolar stimulation) in trajectories with potential FN damage are presented. Data sets of bipolar stimulation can be shared if the reader is interested (see Data Availability Statement). [file Data_Sheet_1.ZIP › Analysis_EMG_Amplitude_Changes/EMGAmp_OutputData/Subject2/Subject2_Traj5_Point4_EMGepochs.png]

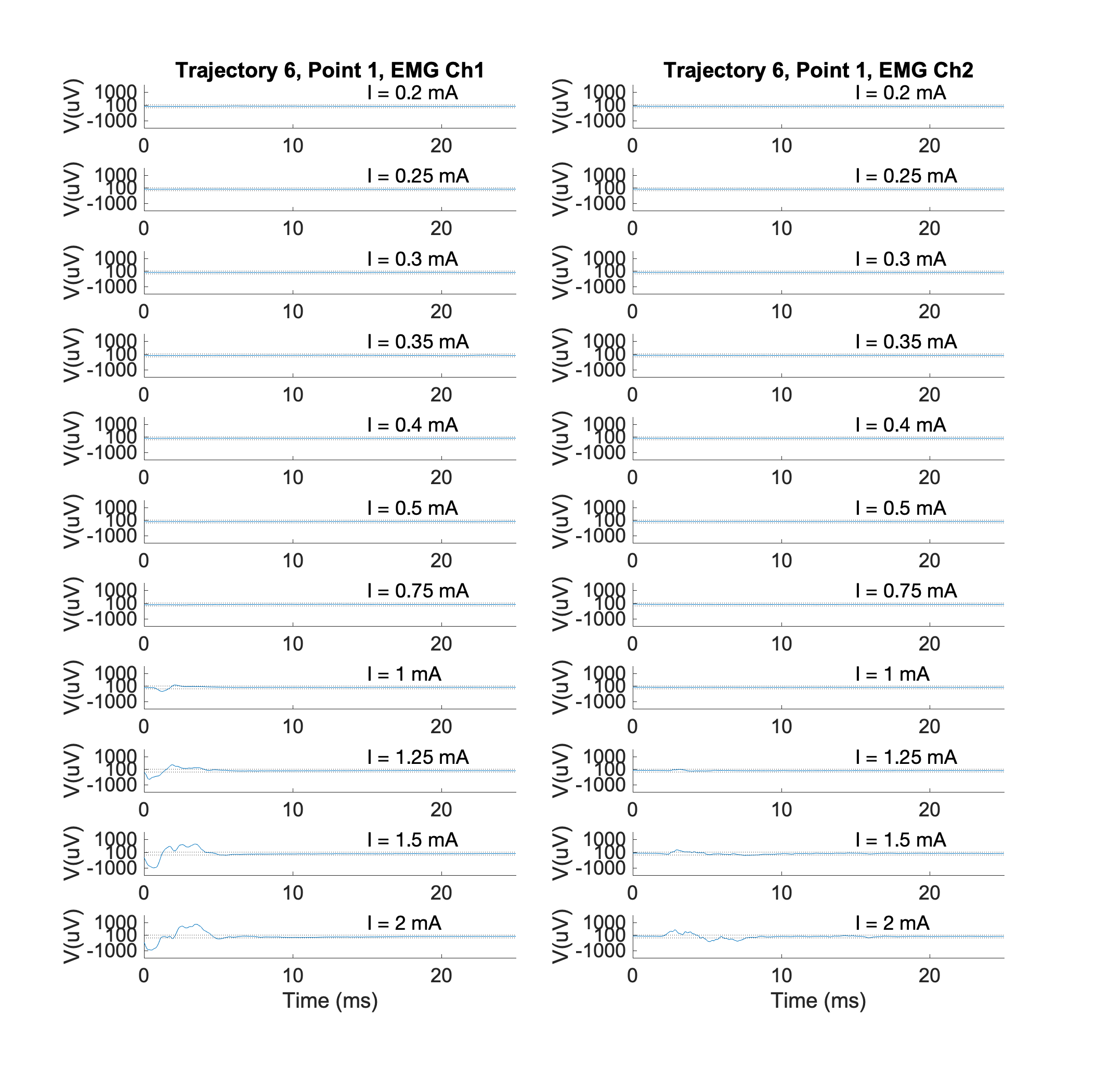

Supplement: Supplementary Data Sheet 1 — Overview of recorded electromyography data showing CMAP responses to the stimulation intensity ramp at each measurement point for the monopolar stimulation. A graph with maximum CMAP responses of monopolar stimulation for each trajectory is depicted. A Summary report (Subject 1, 2, 3.docx) of CMAP responses (for monopolar stimulation) in trajectories with potential FN damage are presented. Data sets of bipolar stimulation can be shared if the reader is interested (see Data Availability Statement). [file Data_Sheet_1.ZIP › Analysis_EMG_Amplitude_Changes/EMGAmp_OutputData/Subject2/Subject2_Traj6_Point1_EMGepochs.png]

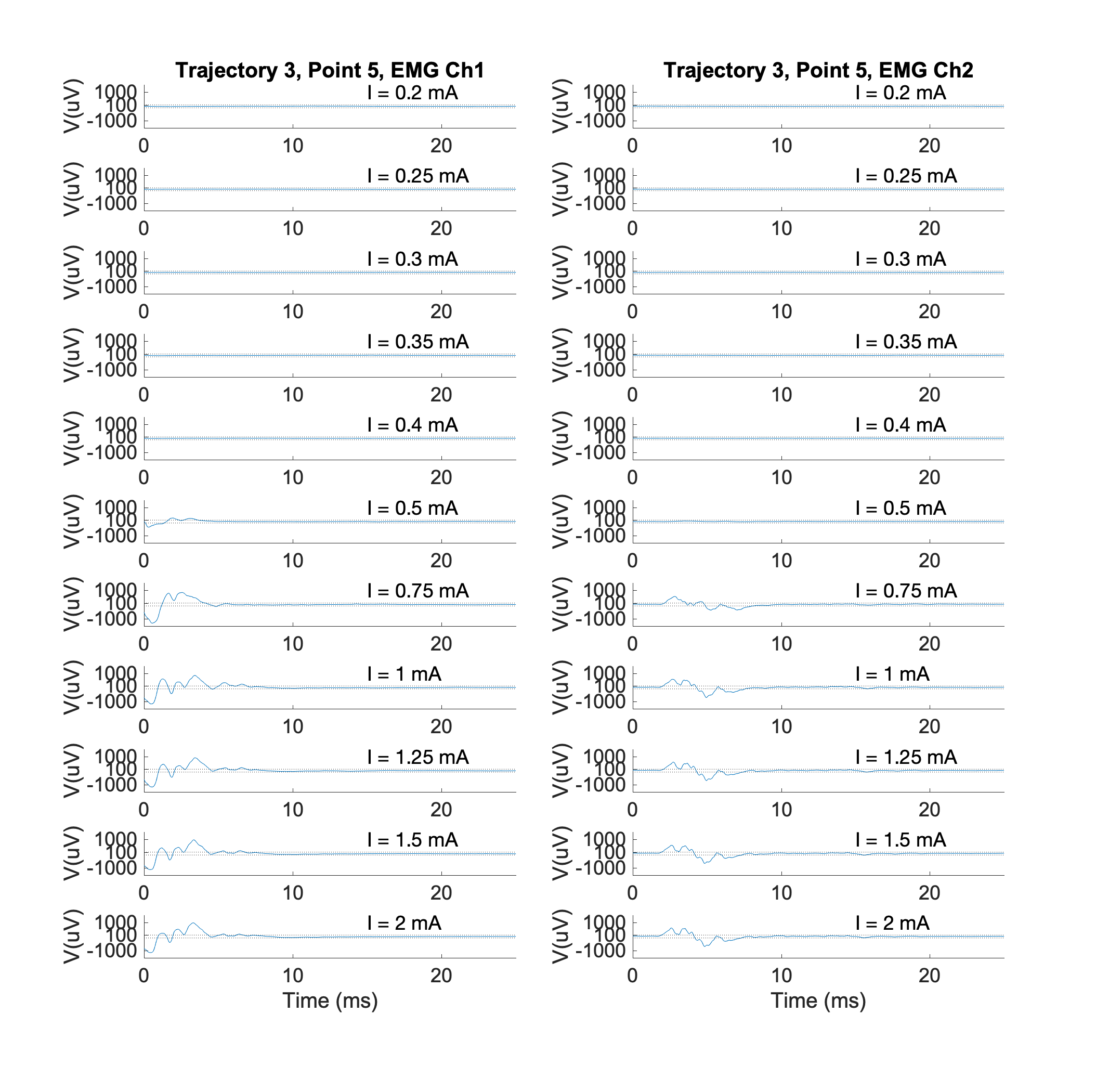

Supplement: Supplementary Data Sheet 1 — Overview of recorded electromyography data showing CMAP responses to the stimulation intensity ramp at each measurement point for the monopolar stimulation. A graph with maximum CMAP responses of monopolar stimulation for each trajectory is depicted. A Summary report (Subject 1, 2, 3.docx) of CMAP responses (for monopolar stimulation) in trajectories with potential FN damage are presented. Data sets of bipolar stimulation can be shared if the reader is interested (see Data Availability Statement). [file Data_Sheet_1.ZIP › Analysis_EMG_Amplitude_Changes/EMGAmp_OutputData/Subject2/Subject2_Traj3_Point5_EMGepochs.png]

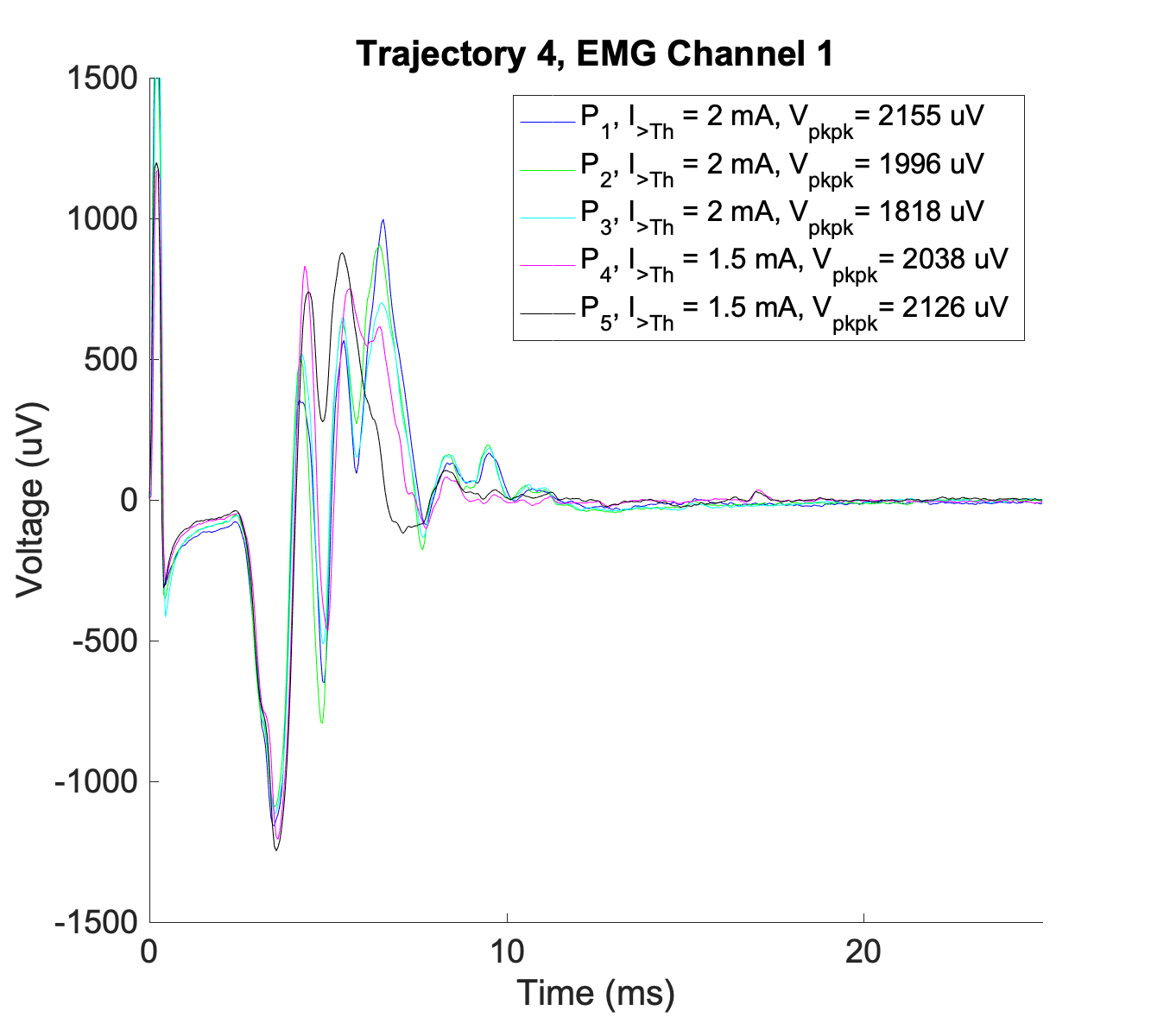

Supplement: Supplementary Data Sheet 1 — Overview of recorded electromyography data showing CMAP responses to the stimulation intensity ramp at each measurement point for the monopolar stimulation. A graph with maximum CMAP responses of monopolar stimulation for each trajectory is depicted. A Summary report (Subject 1, 2, 3.docx) of CMAP responses (for monopolar stimulation) in trajectories with potential FN damage are presented. Data sets of bipolar stimulation can be shared if the reader is interested (see Data Availability Statement). [file Data_Sheet_1.ZIP › Analysis_EMG_Amplitude_Changes/EMGAmp_OutputData/Subject2/Subject2_Traj4_AllPoints_EMG_CH1.png]

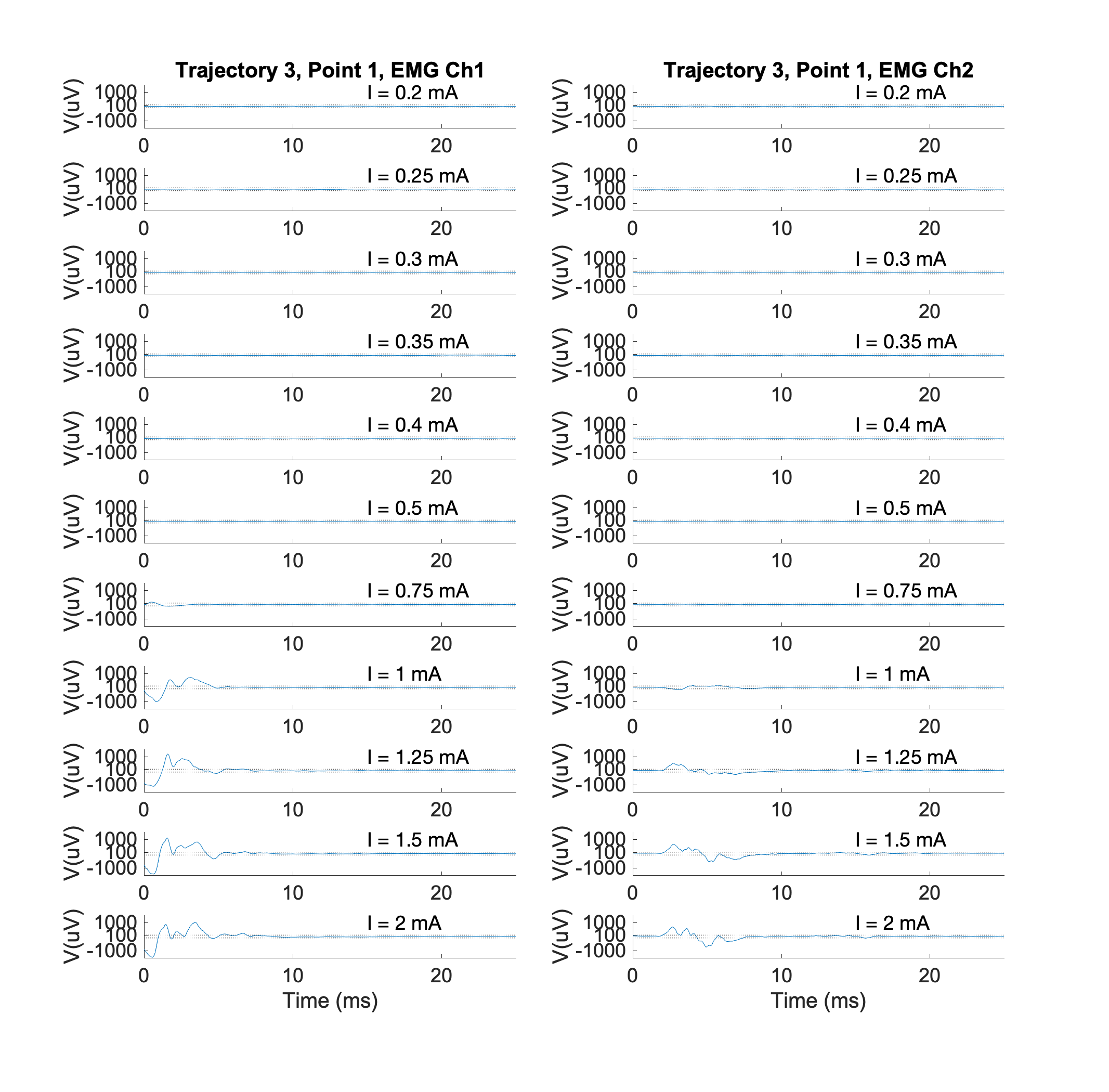

Supplement: Supplementary Data Sheet 1 — Overview of recorded electromyography data showing CMAP responses to the stimulation intensity ramp at each measurement point for the monopolar stimulation. A graph with maximum CMAP responses of monopolar stimulation for each trajectory is depicted. A Summary report (Subject 1, 2, 3.docx) of CMAP responses (for monopolar stimulation) in trajectories with potential FN damage are presented. Data sets of bipolar stimulation can be shared if the reader is interested (see Data Availability Statement). [file Data_Sheet_1.ZIP › Analysis_EMG_Amplitude_Changes/EMGAmp_OutputData/Subject2/Subject2_Traj3_Point1_EMGepochs.png]

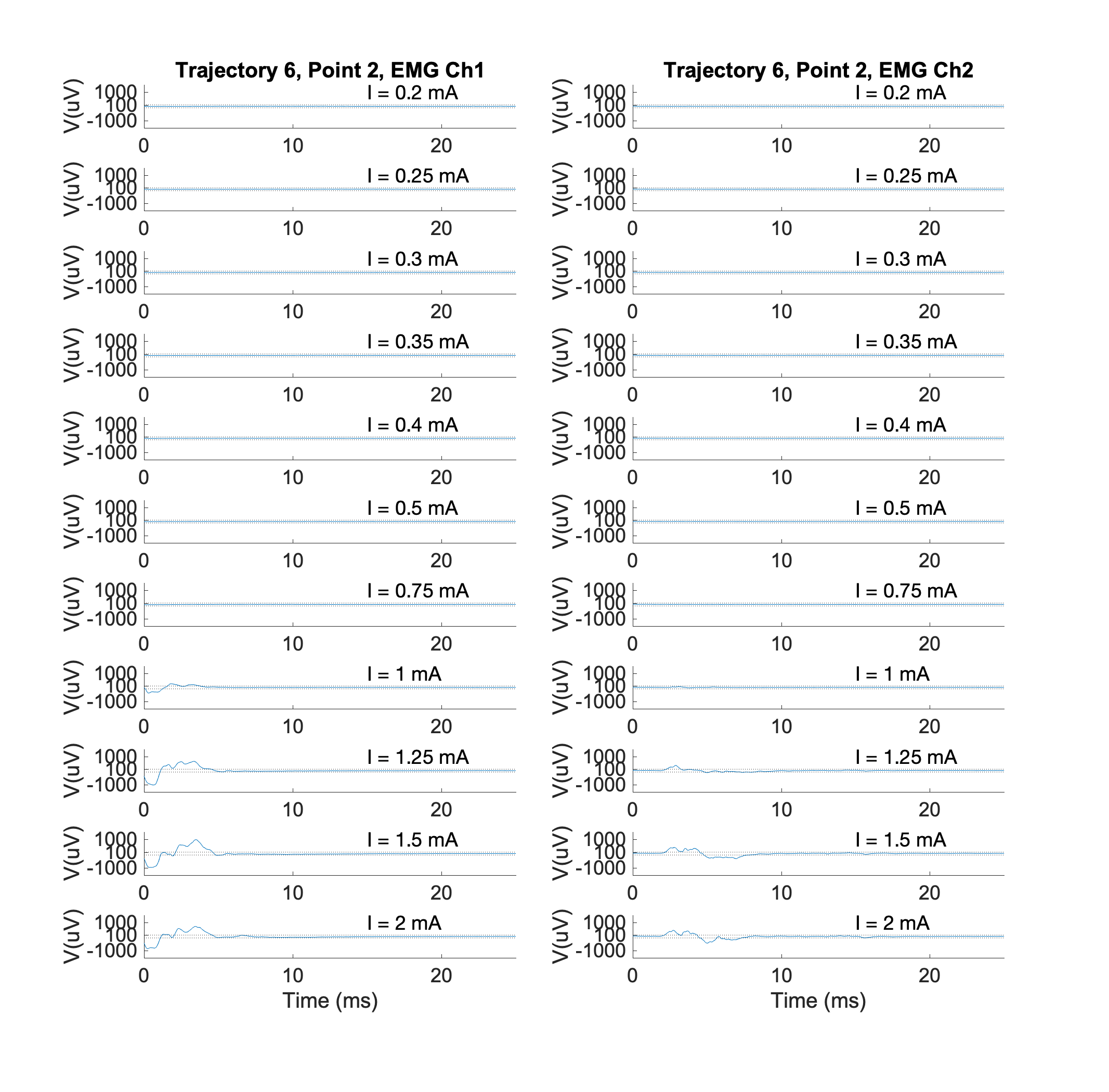

Supplement: Supplementary Data Sheet 1 — Overview of recorded electromyography data showing CMAP responses to the stimulation intensity ramp at each measurement point for the monopolar stimulation. A graph with maximum CMAP responses of monopolar stimulation for each trajectory is depicted. A Summary report (Subject 1, 2, 3.docx) of CMAP responses (for monopolar stimulation) in trajectories with potential FN damage are presented. Data sets of bipolar stimulation can be shared if the reader is interested (see Data Availability Statement). [file Data_Sheet_1.ZIP › Analysis_EMG_Amplitude_Changes/EMGAmp_OutputData/Subject2/Subject2_Traj6_Point2_EMGepochs.png]

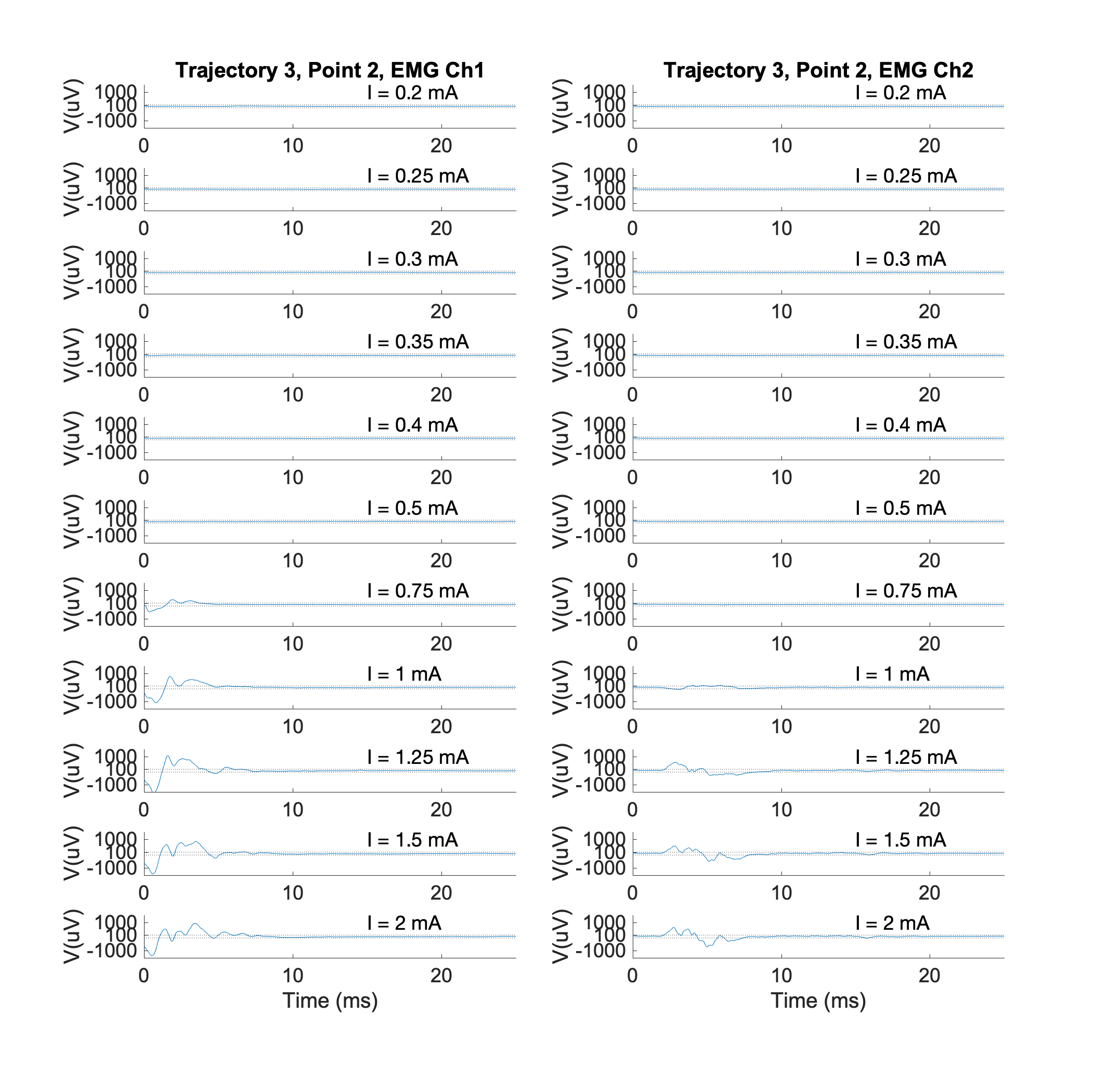

Supplement: Supplementary Data Sheet 1 — Overview of recorded electromyography data showing CMAP responses to the stimulation intensity ramp at each measurement point for the monopolar stimulation. A graph with maximum CMAP responses of monopolar stimulation for each trajectory is depicted. A Summary report (Subject 1, 2, 3.docx) of CMAP responses (for monopolar stimulation) in trajectories with potential FN damage are presented. Data sets of bipolar stimulation can be shared if the reader is interested (see Data Availability Statement). [file Data_Sheet_1.ZIP › Analysis_EMG_Amplitude_Changes/EMGAmp_OutputData/Subject2/Subject2_Traj3_Point2_EMGepochs.png]

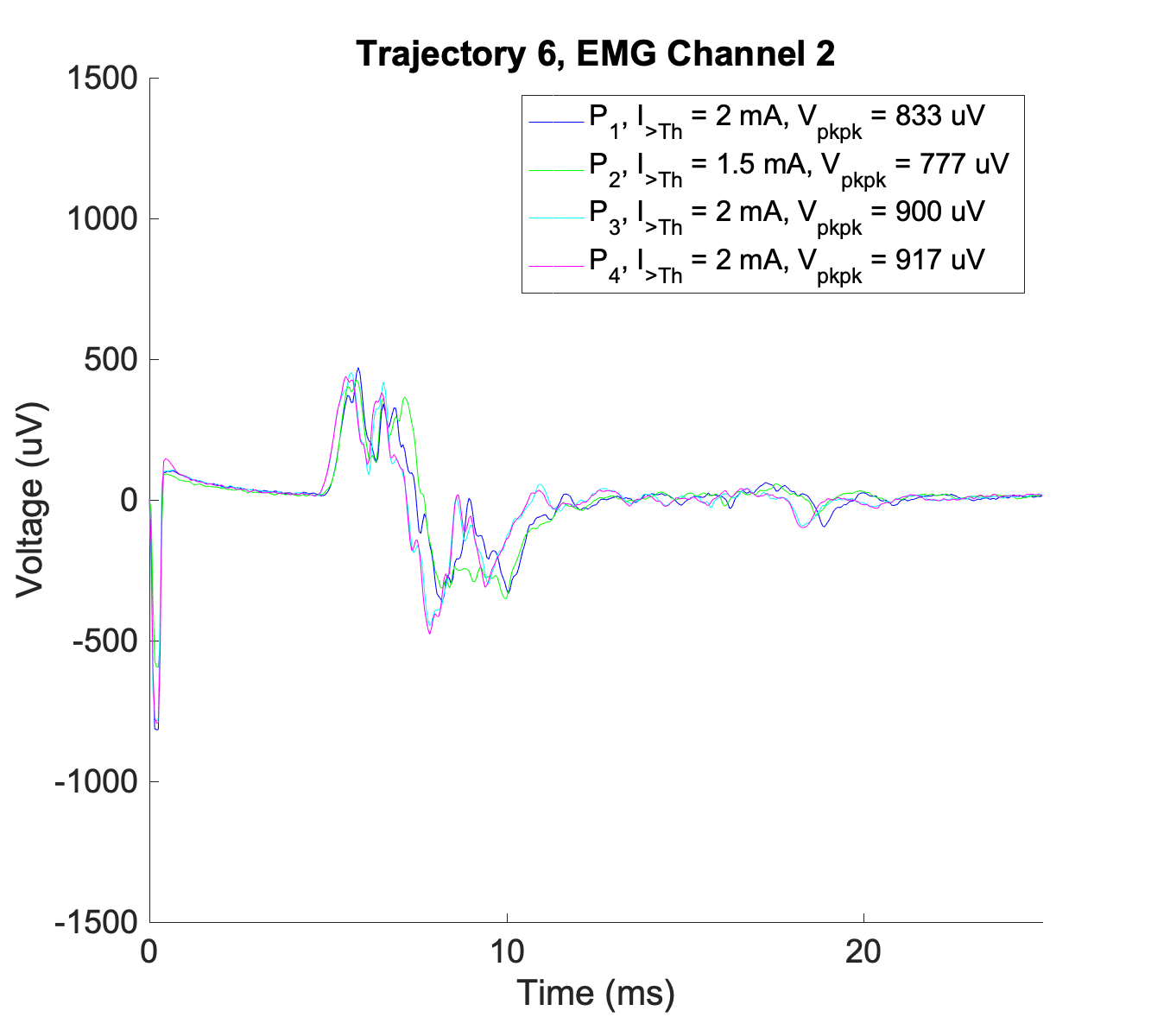

Supplement: Supplementary Data Sheet 1 — Overview of recorded electromyography data showing CMAP responses to the stimulation intensity ramp at each measurement point for the monopolar stimulation. A graph with maximum CMAP responses of monopolar stimulation for each trajectory is depicted. A Summary report (Subject 1, 2, 3.docx) of CMAP responses (for monopolar stimulation) in trajectories with potential FN damage are presented. Data sets of bipolar stimulation can be shared if the reader is interested (see Data Availability Statement). [file Data_Sheet_1.ZIP › Analysis_EMG_Amplitude_Changes/EMGAmp_OutputData/Subject2/Subject2_Traj6_AllPoints_EMG_CH2.png]

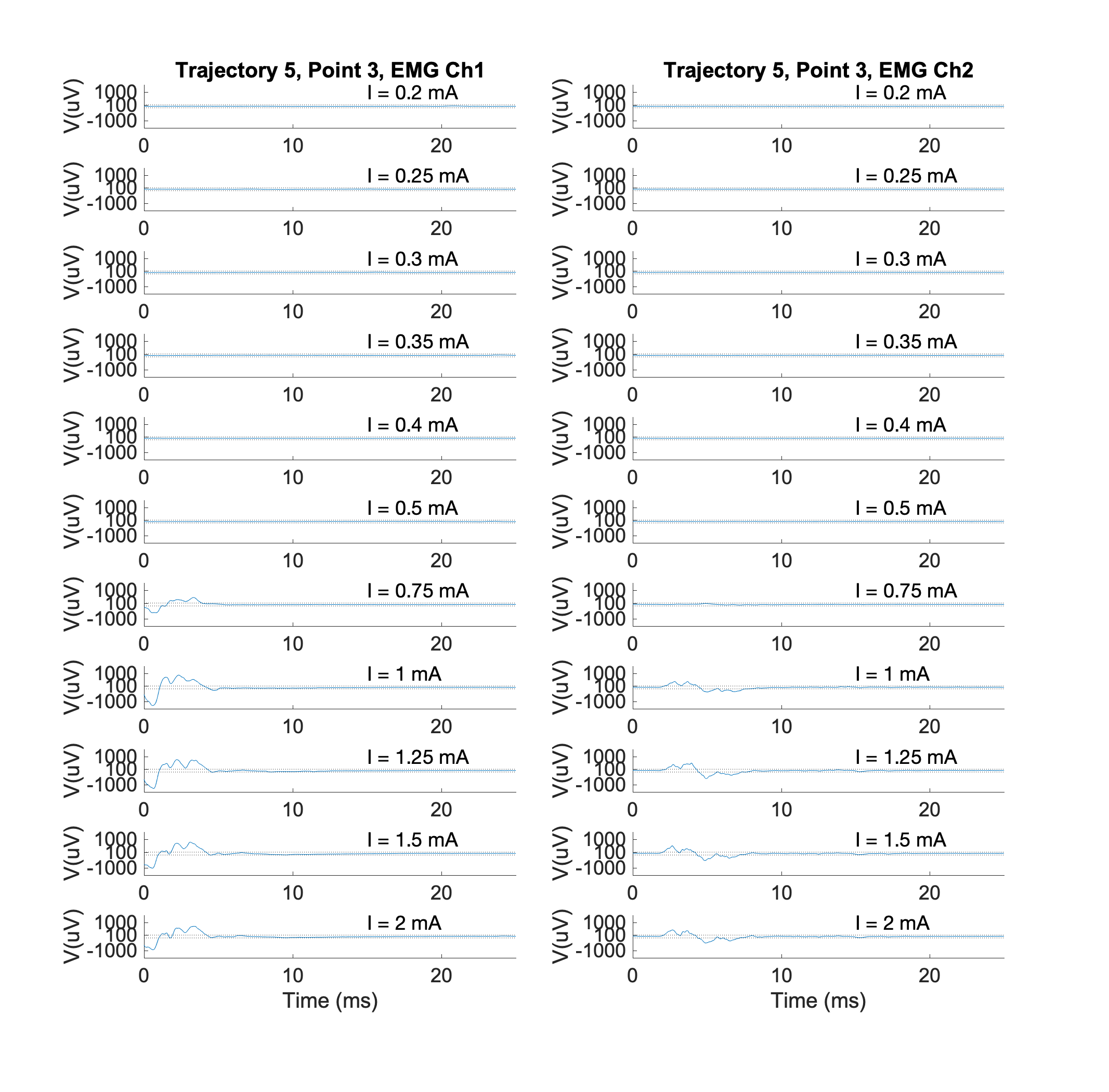

Supplement: Supplementary Data Sheet 1 — Overview of recorded electromyography data showing CMAP responses to the stimulation intensity ramp at each measurement point for the monopolar stimulation. A graph with maximum CMAP responses of monopolar stimulation for each trajectory is depicted. A Summary report (Subject 1, 2, 3.docx) of CMAP responses (for monopolar stimulation) in trajectories with potential FN damage are presented. Data sets of bipolar stimulation can be shared if the reader is interested (see Data Availability Statement). [file Data_Sheet_1.ZIP › Analysis_EMG_Amplitude_Changes/EMGAmp_OutputData/Subject2/Subject2_Traj5_Point3_EMGepochs.png]

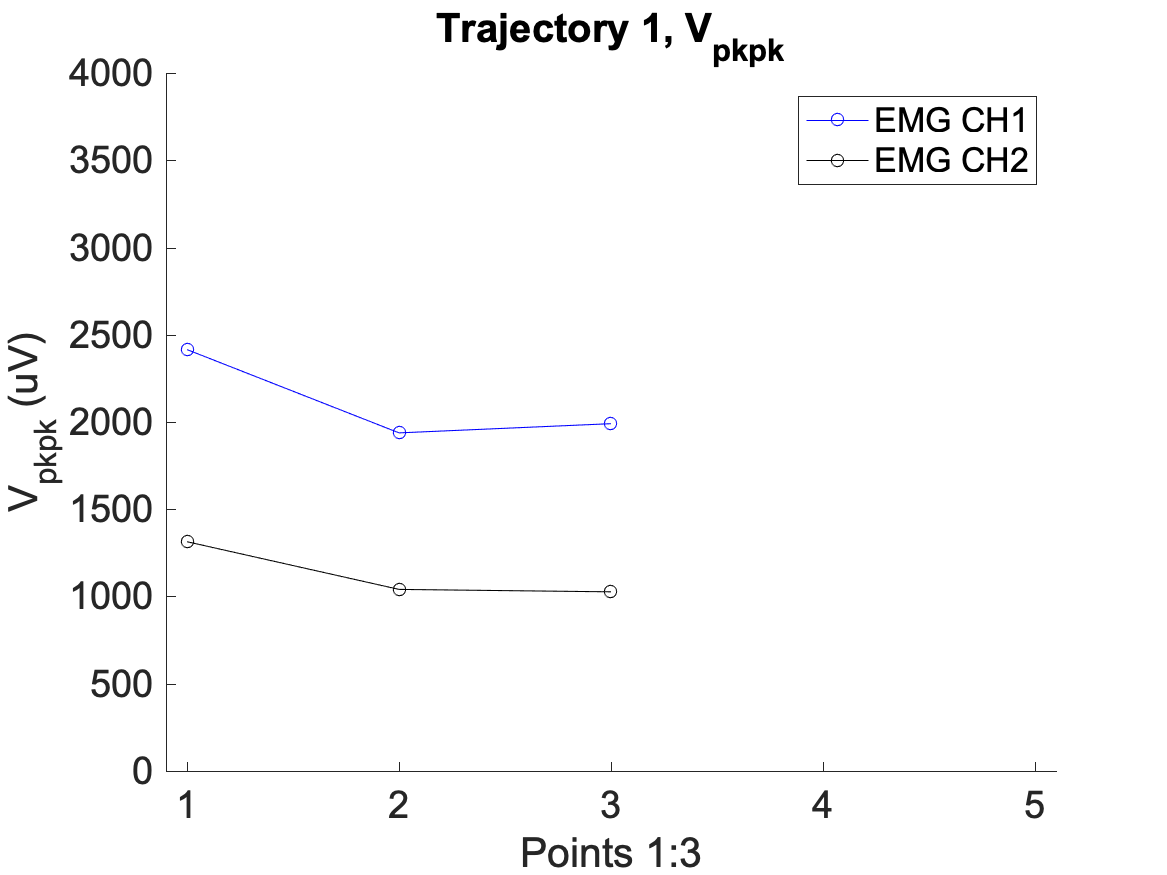

Supplement: Supplementary Data Sheet 1 — Overview of recorded electromyography data showing CMAP responses to the stimulation intensity ramp at each measurement point for the monopolar stimulation. A graph with maximum CMAP responses of monopolar stimulation for each trajectory is depicted. A Summary report (Subject 1, 2, 3.docx) of CMAP responses (for monopolar stimulation) in trajectories with potential FN damage are presented. Data sets of bipolar stimulation can be shared if the reader is interested (see Data Availability Statement). [file Data_Sheet_1.ZIP › Analysis_EMG_Amplitude_Changes/EMGAmp_OutputData/Subject2/Subject2_Traj1_Vpkk.png]

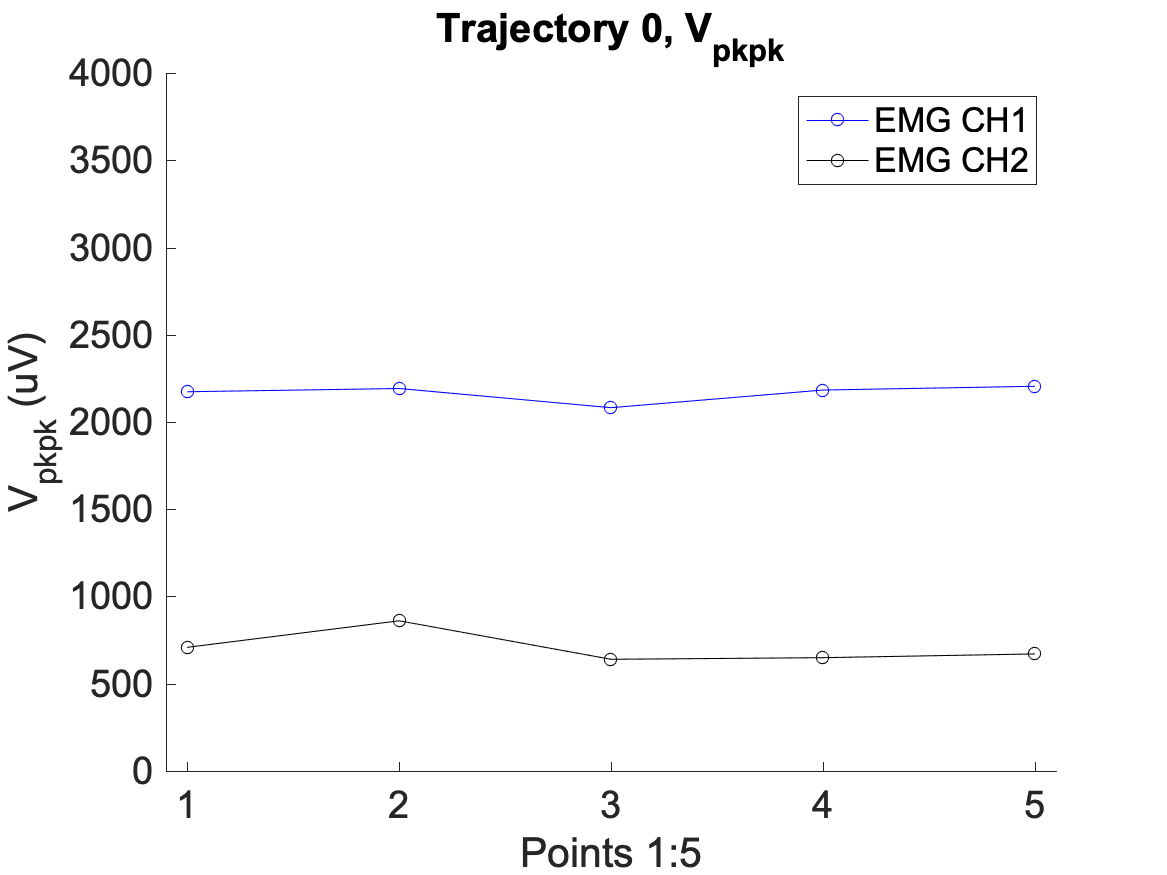

Supplement: Supplementary Data Sheet 1 — Overview of recorded electromyography data showing CMAP responses to the stimulation intensity ramp at each measurement point for the monopolar stimulation. A graph with maximum CMAP responses of monopolar stimulation for each trajectory is depicted. A Summary report (Subject 1, 2, 3.docx) of CMAP responses (for monopolar stimulation) in trajectories with potential FN damage are presented. Data sets of bipolar stimulation can be shared if the reader is interested (see Data Availability Statement). [file Data_Sheet_1.ZIP › Analysis_EMG_Amplitude_Changes/EMGAmp_OutputData/Subject2/Subject2_Traj0_Vpkk.png]

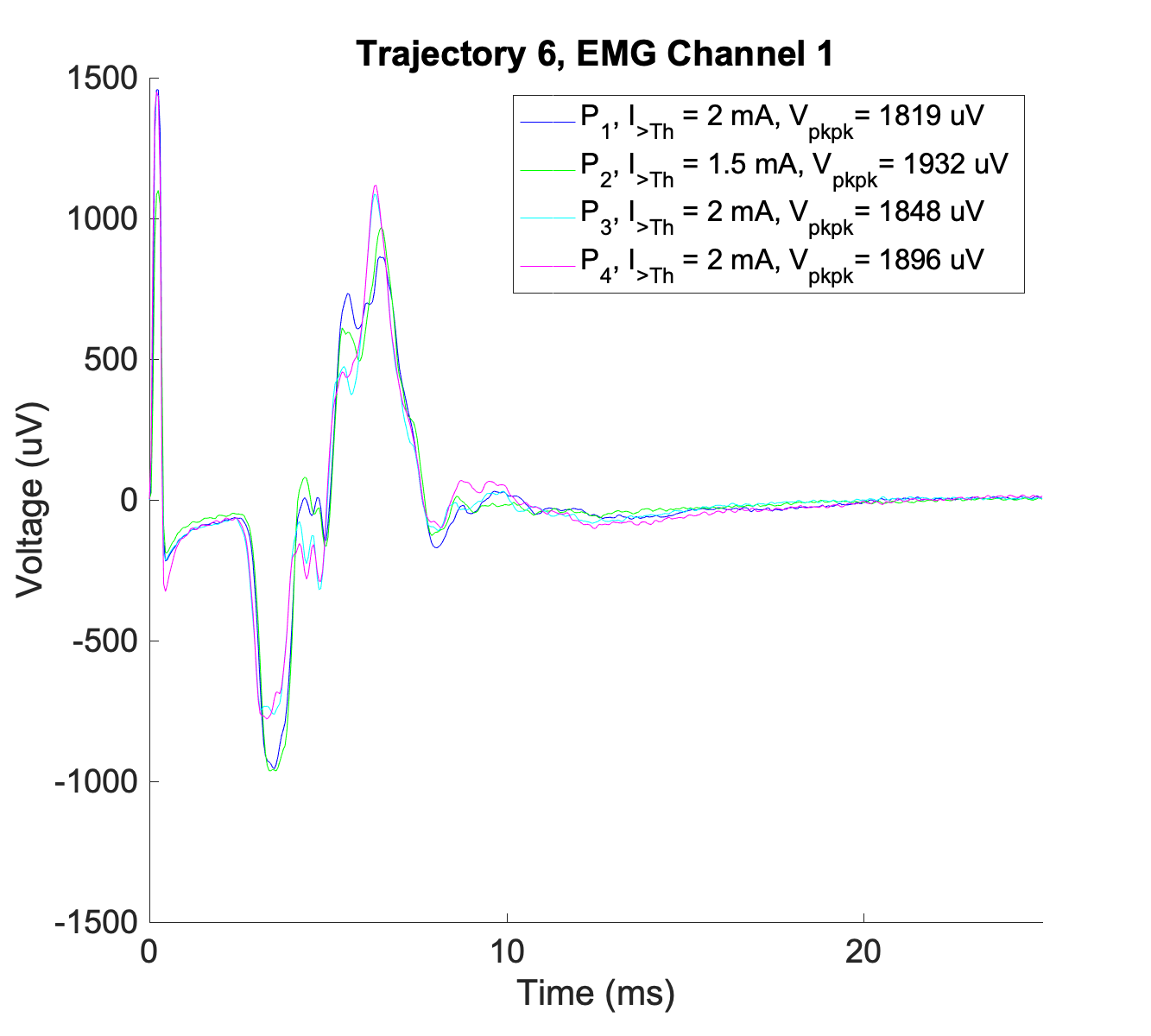

Supplement: Supplementary Data Sheet 1 — Overview of recorded electromyography data showing CMAP responses to the stimulation intensity ramp at each measurement point for the monopolar stimulation. A graph with maximum CMAP responses of monopolar stimulation for each trajectory is depicted. A Summary report (Subject 1, 2, 3.docx) of CMAP responses (for monopolar stimulation) in trajectories with potential FN damage are presented. Data sets of bipolar stimulation can be shared if the reader is interested (see Data Availability Statement). [file Data_Sheet_1.ZIP › Analysis_EMG_Amplitude_Changes/EMGAmp_OutputData/Subject2/Subject2_Traj6_AllPoints_EMG_CH1.png]

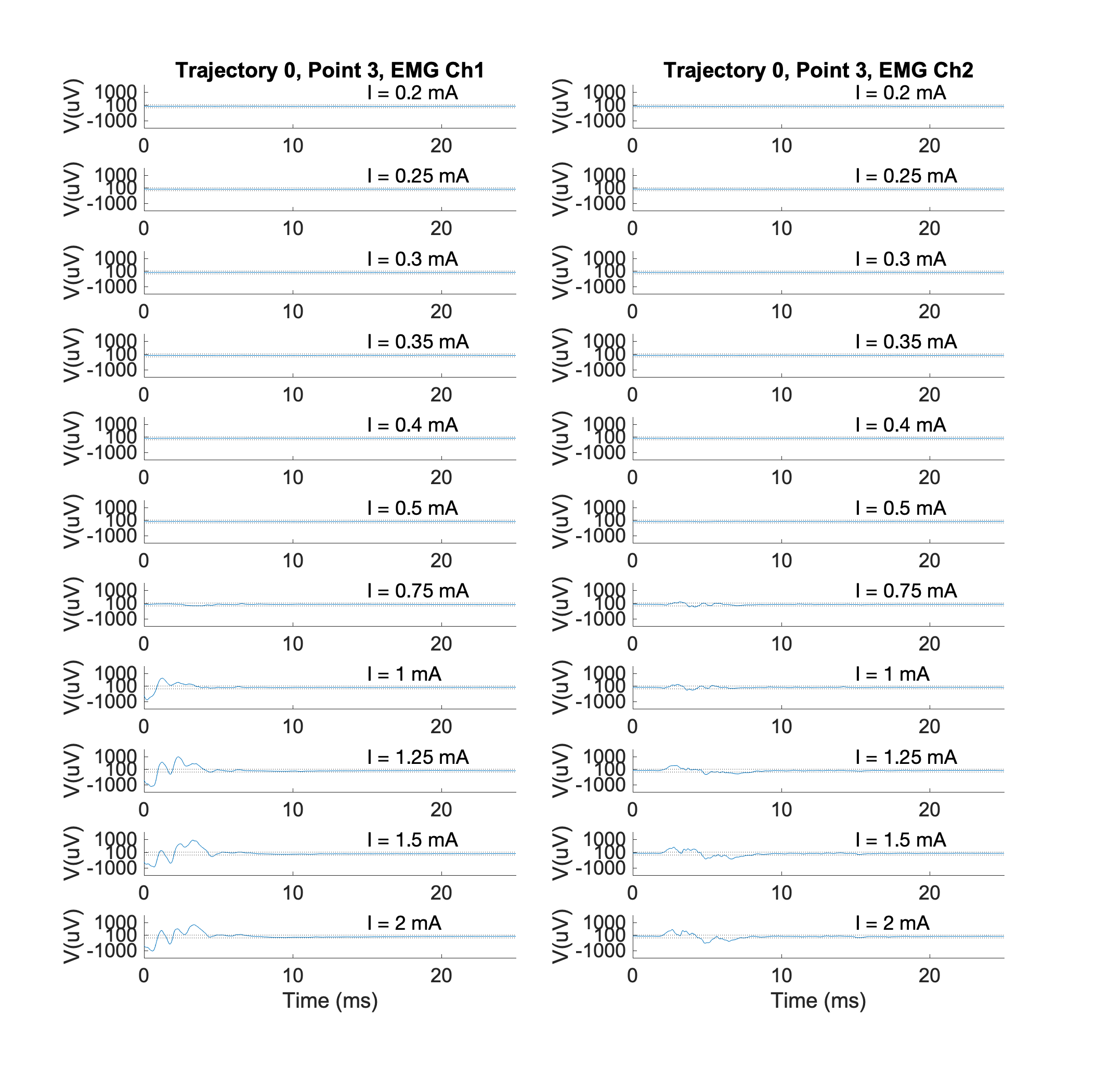

Supplement: Supplementary Data Sheet 1 — Overview of recorded electromyography data showing CMAP responses to the stimulation intensity ramp at each measurement point for the monopolar stimulation. A graph with maximum CMAP responses of monopolar stimulation for each trajectory is depicted. A Summary report (Subject 1, 2, 3.docx) of CMAP responses (for monopolar stimulation) in trajectories with potential FN damage are presented. Data sets of bipolar stimulation can be shared if the reader is interested (see Data Availability Statement). [file Data_Sheet_1.ZIP › Analysis_EMG_Amplitude_Changes/EMGAmp_OutputData/Subject2/Subject2_Traj0_Point3_EMGepochs.png]

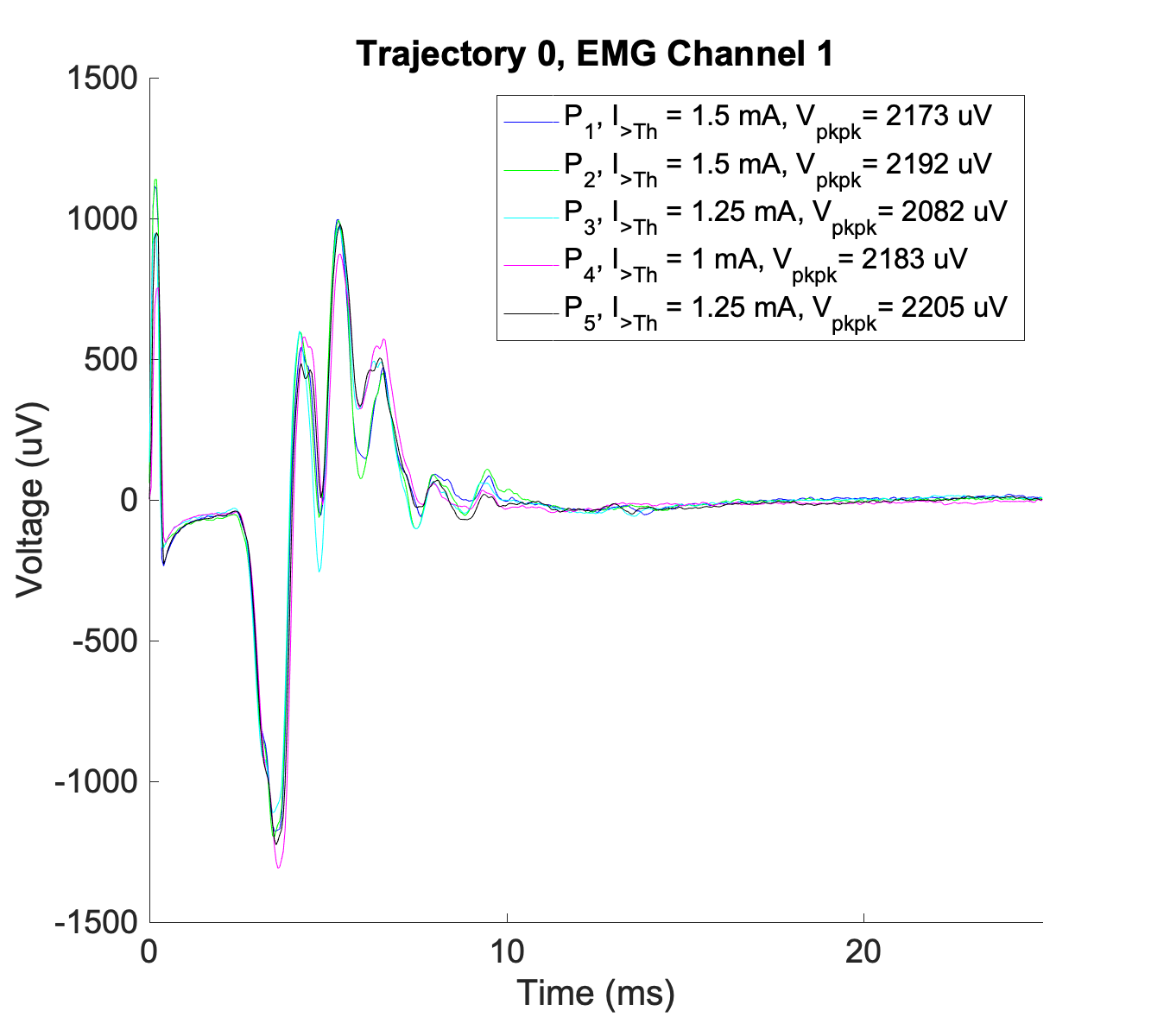

Supplement: Supplementary Data Sheet 1 — Overview of recorded electromyography data showing CMAP responses to the stimulation intensity ramp at each measurement point for the monopolar stimulation. A graph with maximum CMAP responses of monopolar stimulation for each trajectory is depicted. A Summary report (Subject 1, 2, 3.docx) of CMAP responses (for monopolar stimulation) in trajectories with potential FN damage are presented. Data sets of bipolar stimulation can be shared if the reader is interested (see Data Availability Statement). [file Data_Sheet_1.ZIP › Analysis_EMG_Amplitude_Changes/EMGAmp_OutputData/Subject2/Subject2_Traj0_AllPoints_EMG_CH1.png]

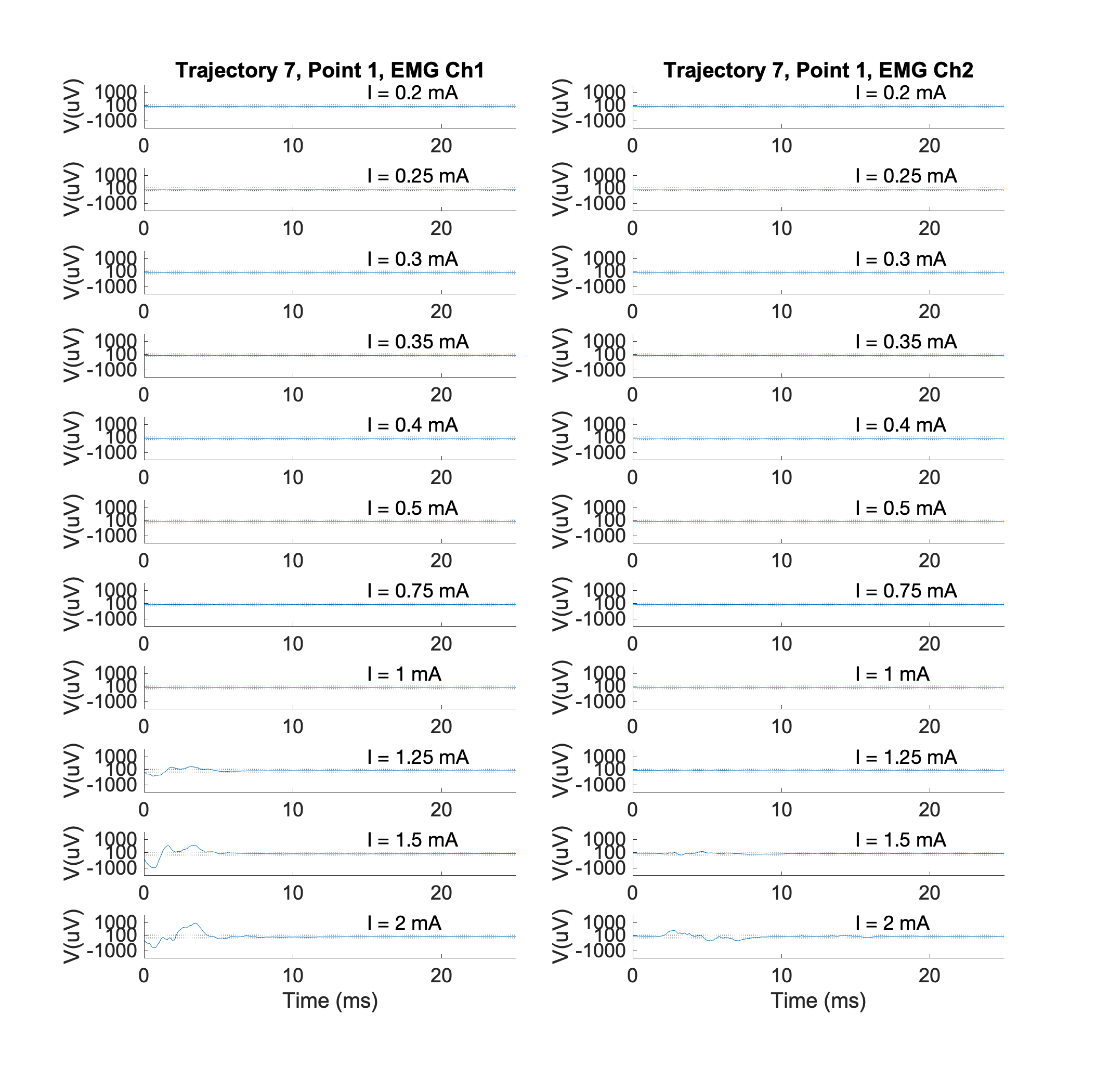

Supplement: Supplementary Data Sheet 1 — Overview of recorded electromyography data showing CMAP responses to the stimulation intensity ramp at each measurement point for the monopolar stimulation. A graph with maximum CMAP responses of monopolar stimulation for each trajectory is depicted. A Summary report (Subject 1, 2, 3.docx) of CMAP responses (for monopolar stimulation) in trajectories with potential FN damage are presented. Data sets of bipolar stimulation can be shared if the reader is interested (see Data Availability Statement). [file Data_Sheet_1.ZIP › Analysis_EMG_Amplitude_Changes/EMGAmp_OutputData/Subject2/Subject2_Traj7_Point1_EMGepochs.png]

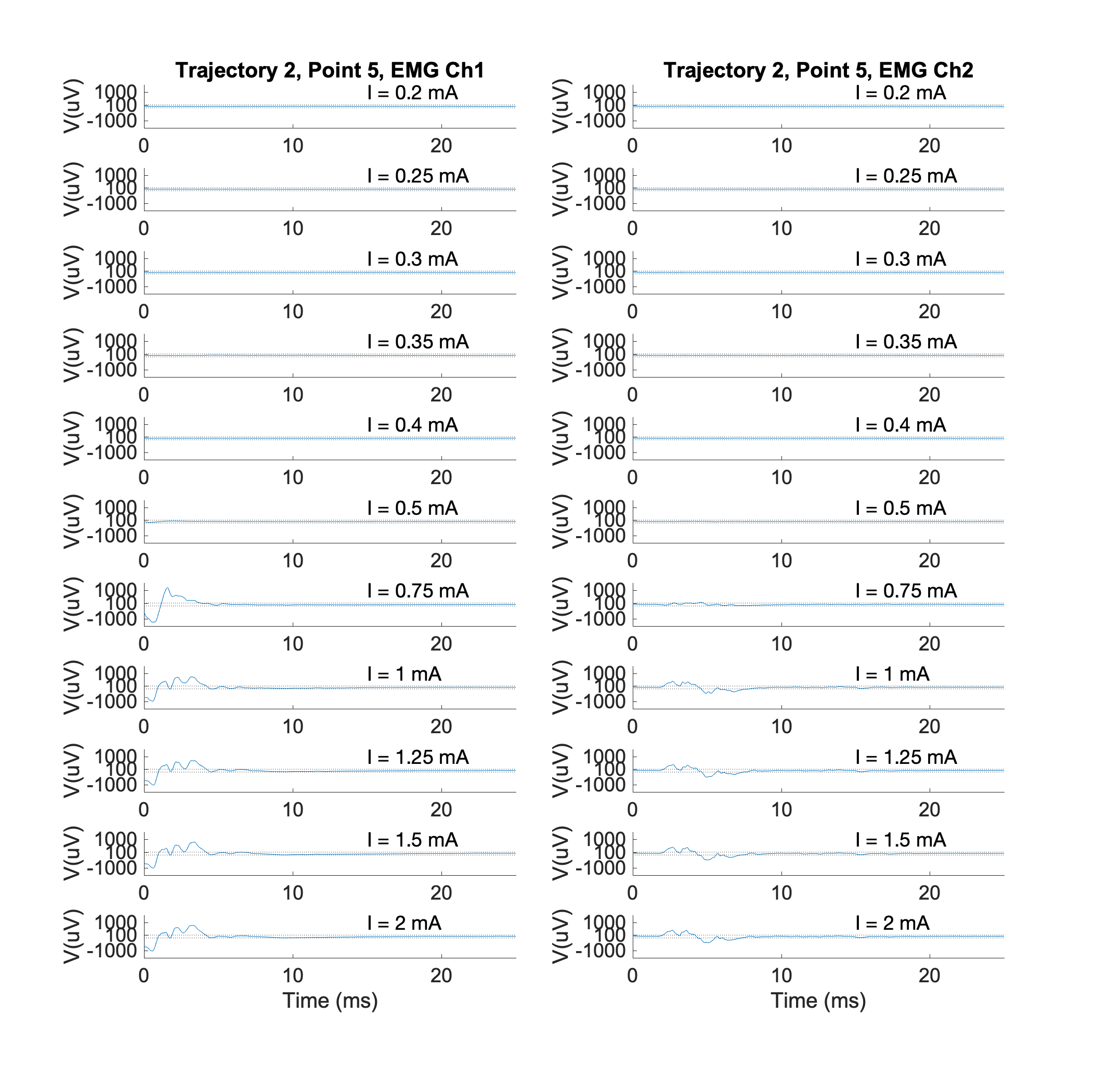

Supplement: Supplementary Data Sheet 1 — Overview of recorded electromyography data showing CMAP responses to the stimulation intensity ramp at each measurement point for the monopolar stimulation. A graph with maximum CMAP responses of monopolar stimulation for each trajectory is depicted. A Summary report (Subject 1, 2, 3.docx) of CMAP responses (for monopolar stimulation) in trajectories with potential FN damage are presented. Data sets of bipolar stimulation can be shared if the reader is interested (see Data Availability Statement). [file Data_Sheet_1.ZIP › Analysis_EMG_Amplitude_Changes/EMGAmp_OutputData/Subject2/Subject2_Traj2_Point5_EMGepochs.png]

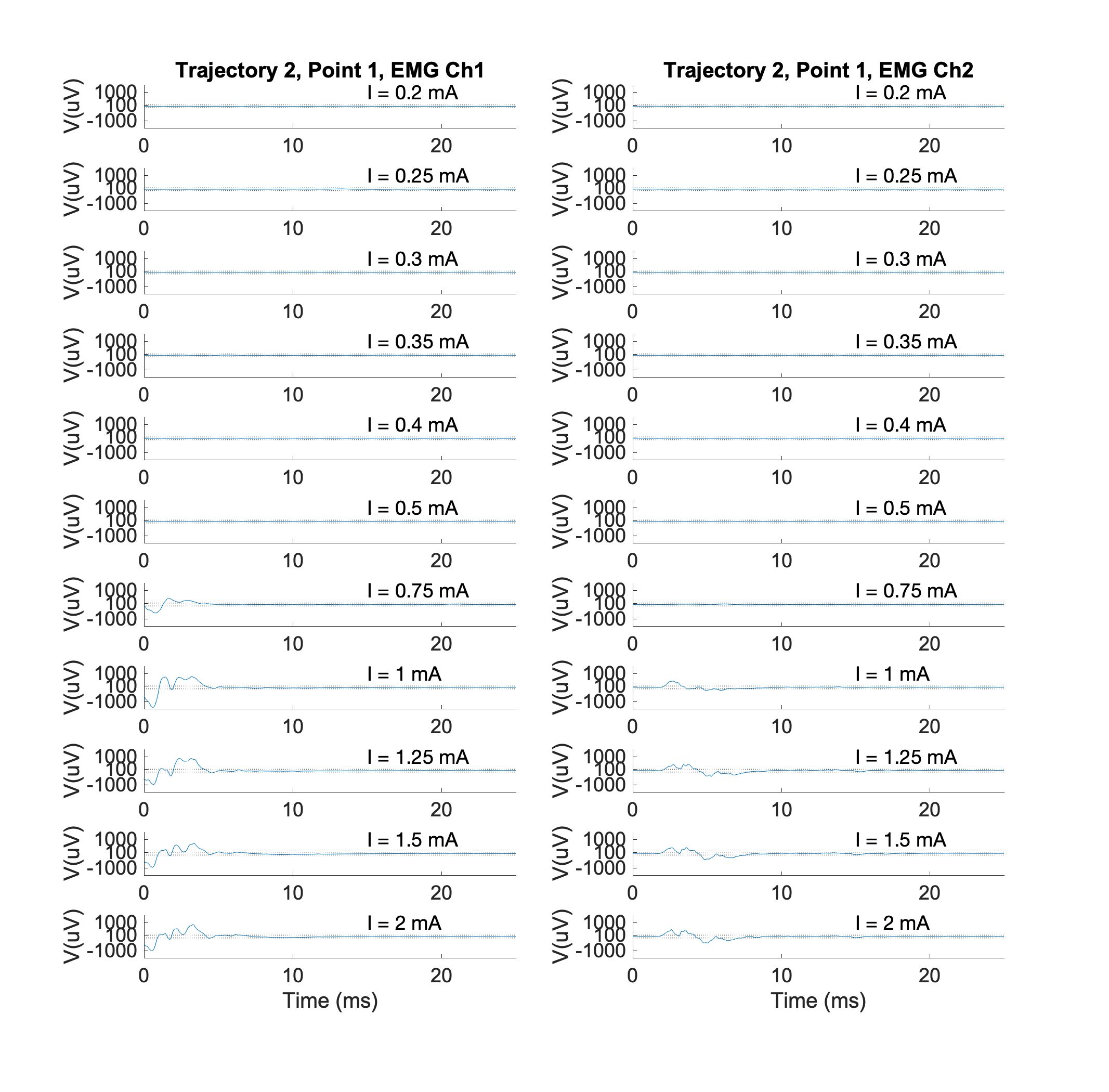

Supplement: Supplementary Data Sheet 1 — Overview of recorded electromyography data showing CMAP responses to the stimulation intensity ramp at each measurement point for the monopolar stimulation. A graph with maximum CMAP responses of monopolar stimulation for each trajectory is depicted. A Summary report (Subject 1, 2, 3.docx) of CMAP responses (for monopolar stimulation) in trajectories with potential FN damage are presented. Data sets of bipolar stimulation can be shared if the reader is interested (see Data Availability Statement). [file Data_Sheet_1.ZIP › Analysis_EMG_Amplitude_Changes/EMGAmp_OutputData/Subject2/Subject2_Traj2_Point1_EMGepochs.png]

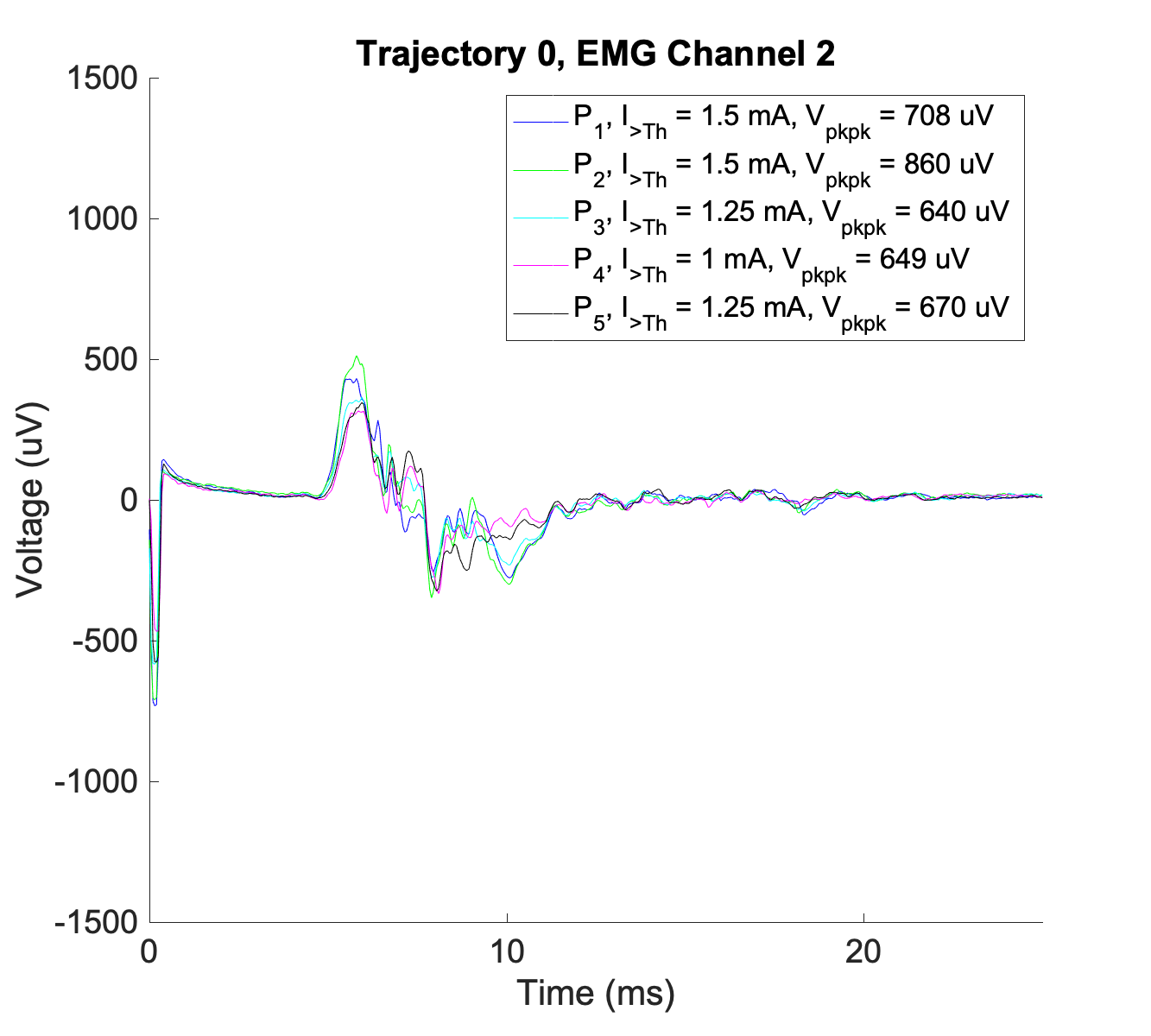

Supplement: Supplementary Data Sheet 1 — Overview of recorded electromyography data showing CMAP responses to the stimulation intensity ramp at each measurement point for the monopolar stimulation. A graph with maximum CMAP responses of monopolar stimulation for each trajectory is depicted. A Summary report (Subject 1, 2, 3.docx) of CMAP responses (for monopolar stimulation) in trajectories with potential FN damage are presented. Data sets of bipolar stimulation can be shared if the reader is interested (see Data Availability Statement). [file Data_Sheet_1.ZIP › Analysis_EMG_Amplitude_Changes/EMGAmp_OutputData/Subject2/Subject2_Traj0_AllPoints_EMG_CH2.png]

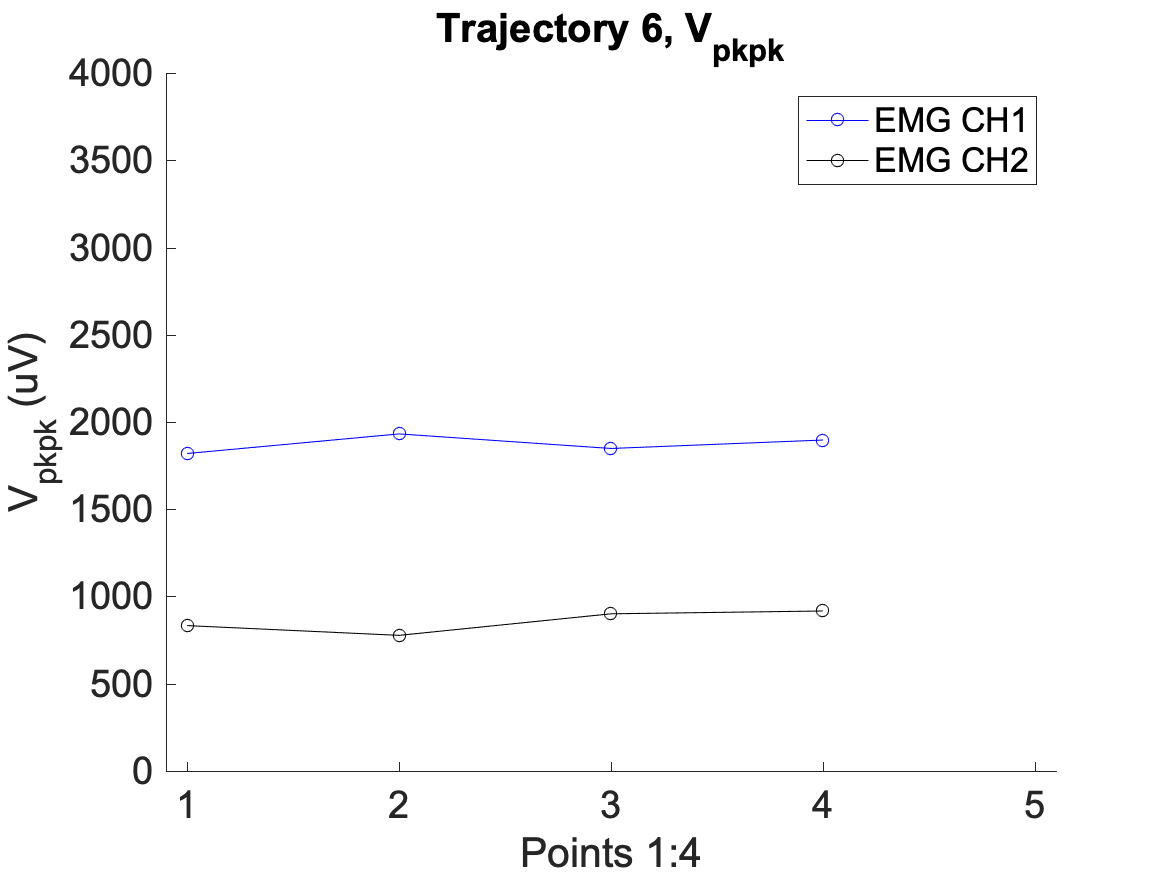

Supplement: Supplementary Data Sheet 1 — Overview of recorded electromyography data showing CMAP responses to the stimulation intensity ramp at each measurement point for the monopolar stimulation. A graph with maximum CMAP responses of monopolar stimulation for each trajectory is depicted. A Summary report (Subject 1, 2, 3.docx) of CMAP responses (for monopolar stimulation) in trajectories with potential FN damage are presented. Data sets of bipolar stimulation can be shared if the reader is interested (see Data Availability Statement). [file Data_Sheet_1.ZIP › Analysis_EMG_Amplitude_Changes/EMGAmp_OutputData/Subject2/Subject2_Traj6_Vpkk.png]

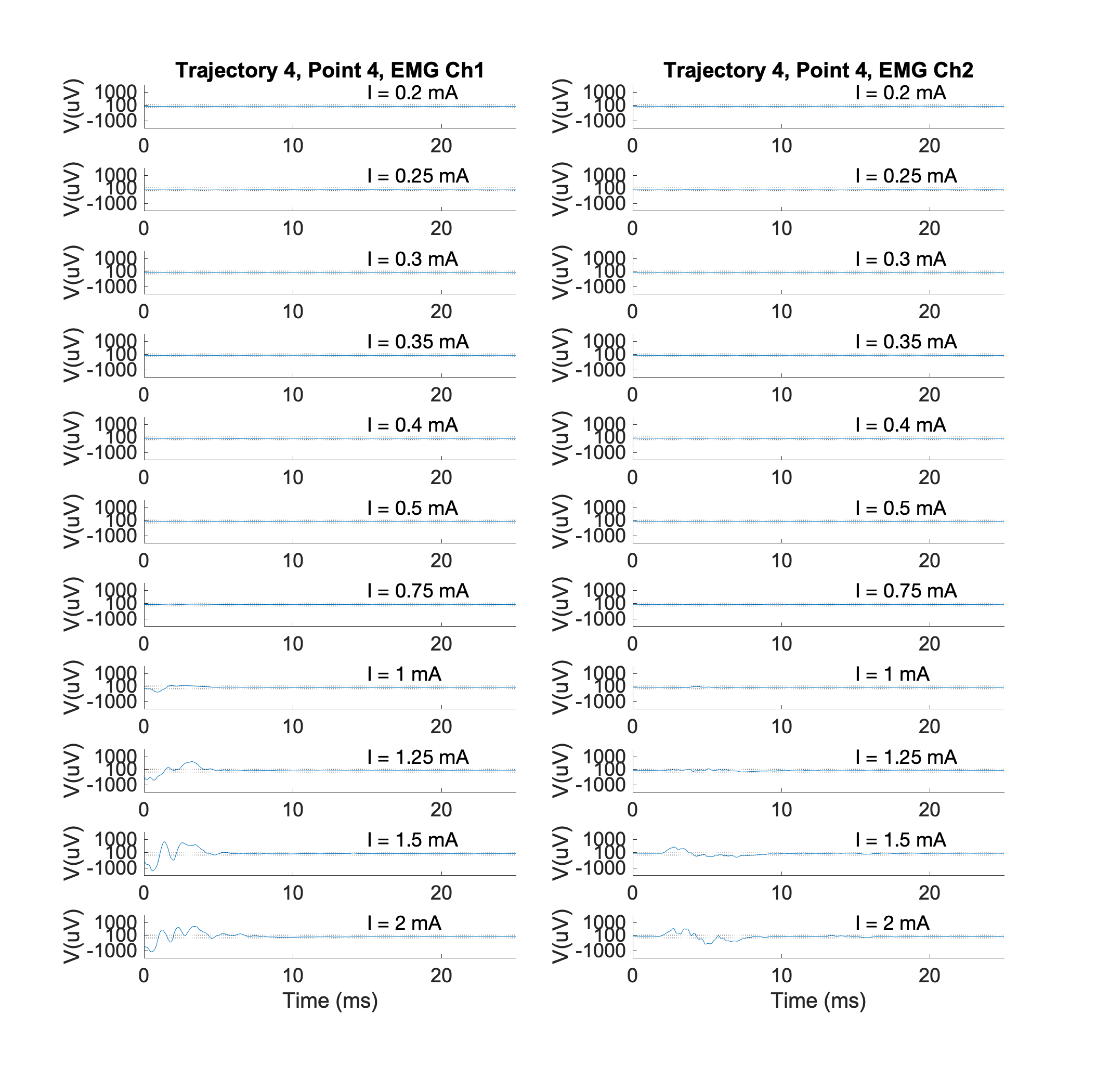

Supplement: Supplementary Data Sheet 1 — Overview of recorded electromyography data showing CMAP responses to the stimulation intensity ramp at each measurement point for the monopolar stimulation. A graph with maximum CMAP responses of monopolar stimulation for each trajectory is depicted. A Summary report (Subject 1, 2, 3.docx) of CMAP responses (for monopolar stimulation) in trajectories with potential FN damage are presented. Data sets of bipolar stimulation can be shared if the reader is interested (see Data Availability Statement). [file Data_Sheet_1.ZIP › Analysis_EMG_Amplitude_Changes/EMGAmp_OutputData/Subject2/Subject2_Traj4_Point4_EMGepochs.png]

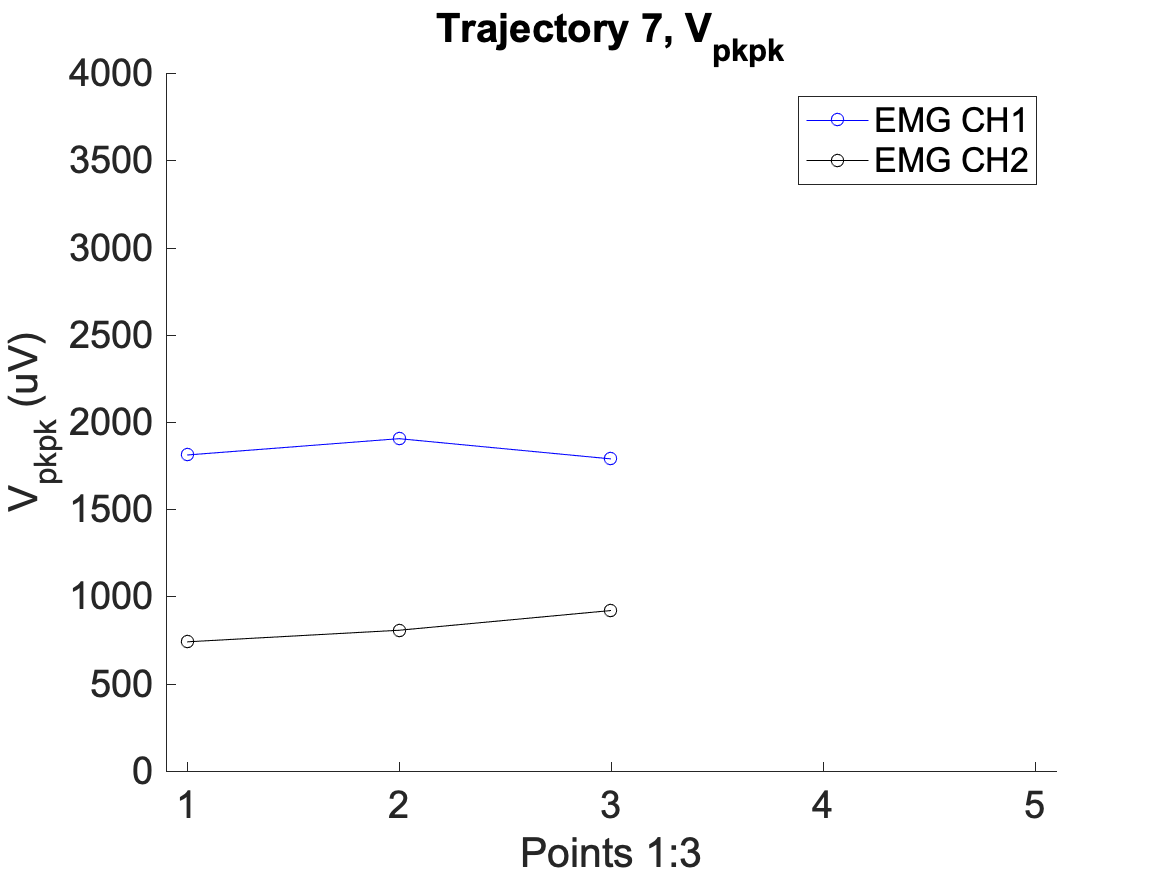

Supplement: Supplementary Data Sheet 1 — Overview of recorded electromyography data showing CMAP responses to the stimulation intensity ramp at each measurement point for the monopolar stimulation. A graph with maximum CMAP responses of monopolar stimulation for each trajectory is depicted. A Summary report (Subject 1, 2, 3.docx) of CMAP responses (for monopolar stimulation) in trajectories with potential FN damage are presented. Data sets of bipolar stimulation can be shared if the reader is interested (see Data Availability Statement). [file Data_Sheet_1.ZIP › Analysis_EMG_Amplitude_Changes/EMGAmp_OutputData/Subject2/Subject2_Traj7_Vpkk.png]

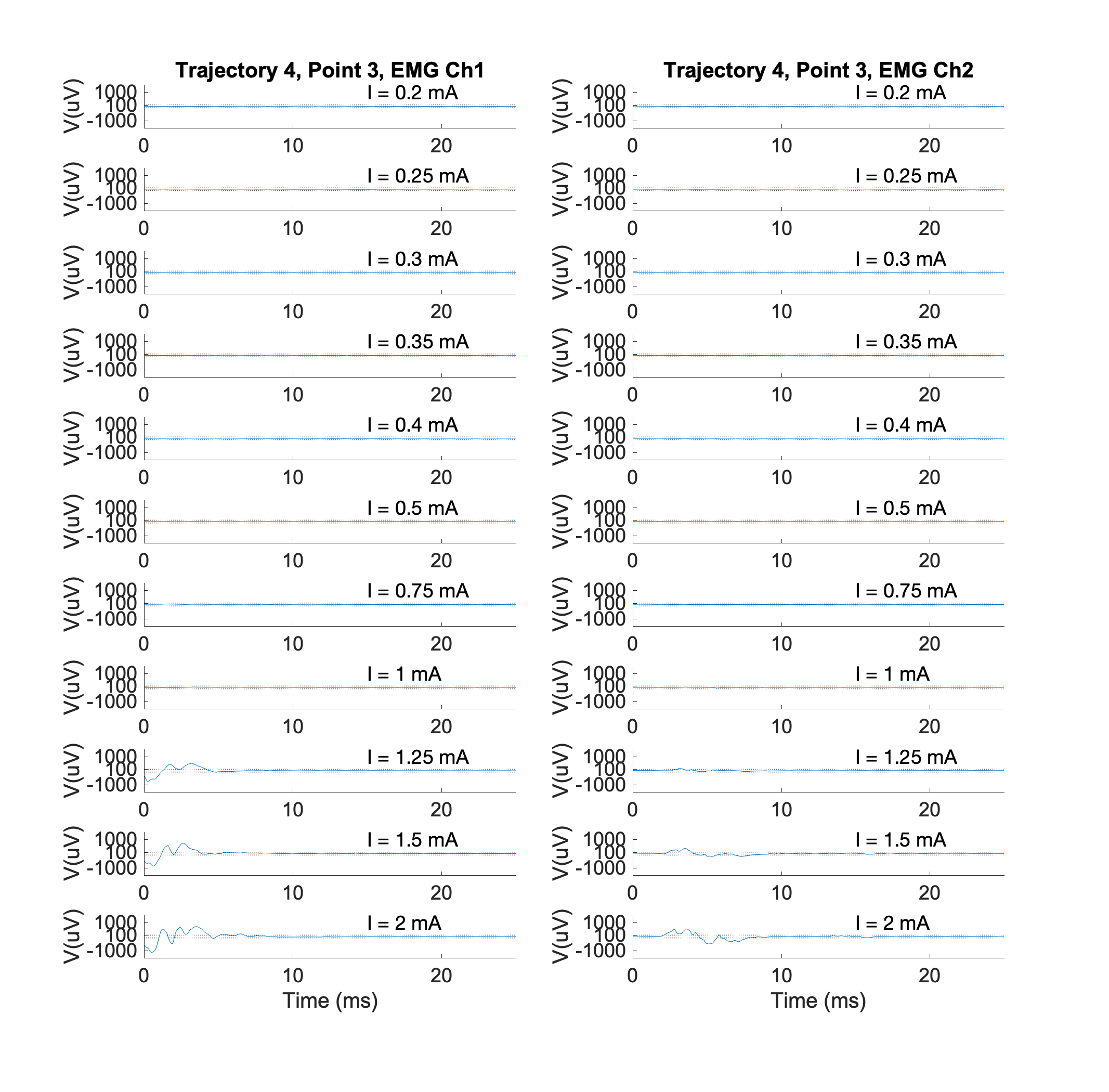

Supplement: Supplementary Data Sheet 1 — Overview of recorded electromyography data showing CMAP responses to the stimulation intensity ramp at each measurement point for the monopolar stimulation. A graph with maximum CMAP responses of monopolar stimulation for each trajectory is depicted. A Summary report (Subject 1, 2, 3.docx) of CMAP responses (for monopolar stimulation) in trajectories with potential FN damage are presented. Data sets of bipolar stimulation can be shared if the reader is interested (see Data Availability Statement). [file Data_Sheet_1.ZIP › Analysis_EMG_Amplitude_Changes/EMGAmp_OutputData/Subject2/Subject2_Traj4_Point3_EMGepochs.png]

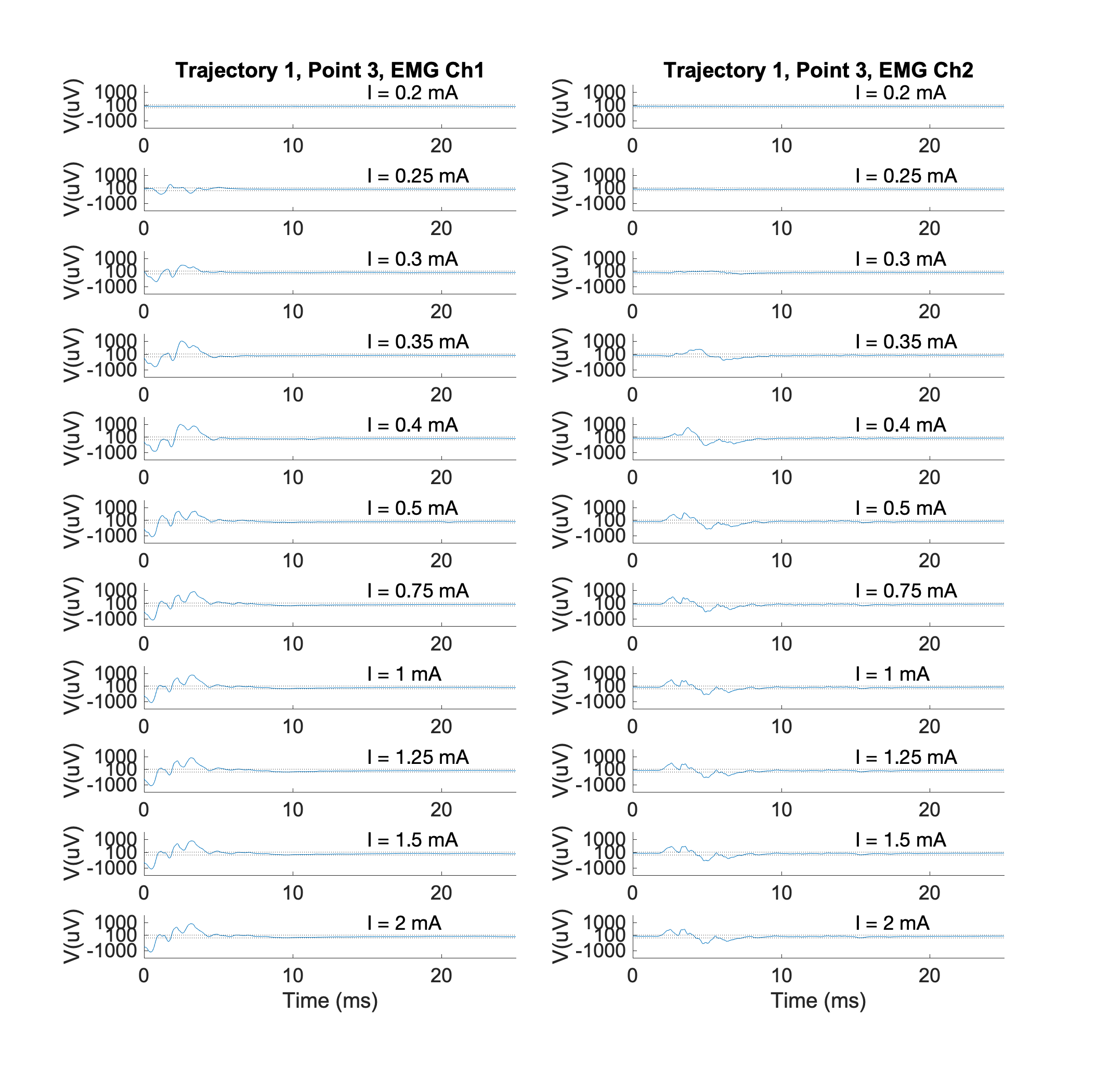

Supplement: Supplementary Data Sheet 1 — Overview of recorded electromyography data showing CMAP responses to the stimulation intensity ramp at each measurement point for the monopolar stimulation. A graph with maximum CMAP responses of monopolar stimulation for each trajectory is depicted. A Summary report (Subject 1, 2, 3.docx) of CMAP responses (for monopolar stimulation) in trajectories with potential FN damage are presented. Data sets of bipolar stimulation can be shared if the reader is interested (see Data Availability Statement). [file Data_Sheet_1.ZIP › Analysis_EMG_Amplitude_Changes/EMGAmp_OutputData/Subject2/Subject2_Traj1_Point3_EMGepochs.png]

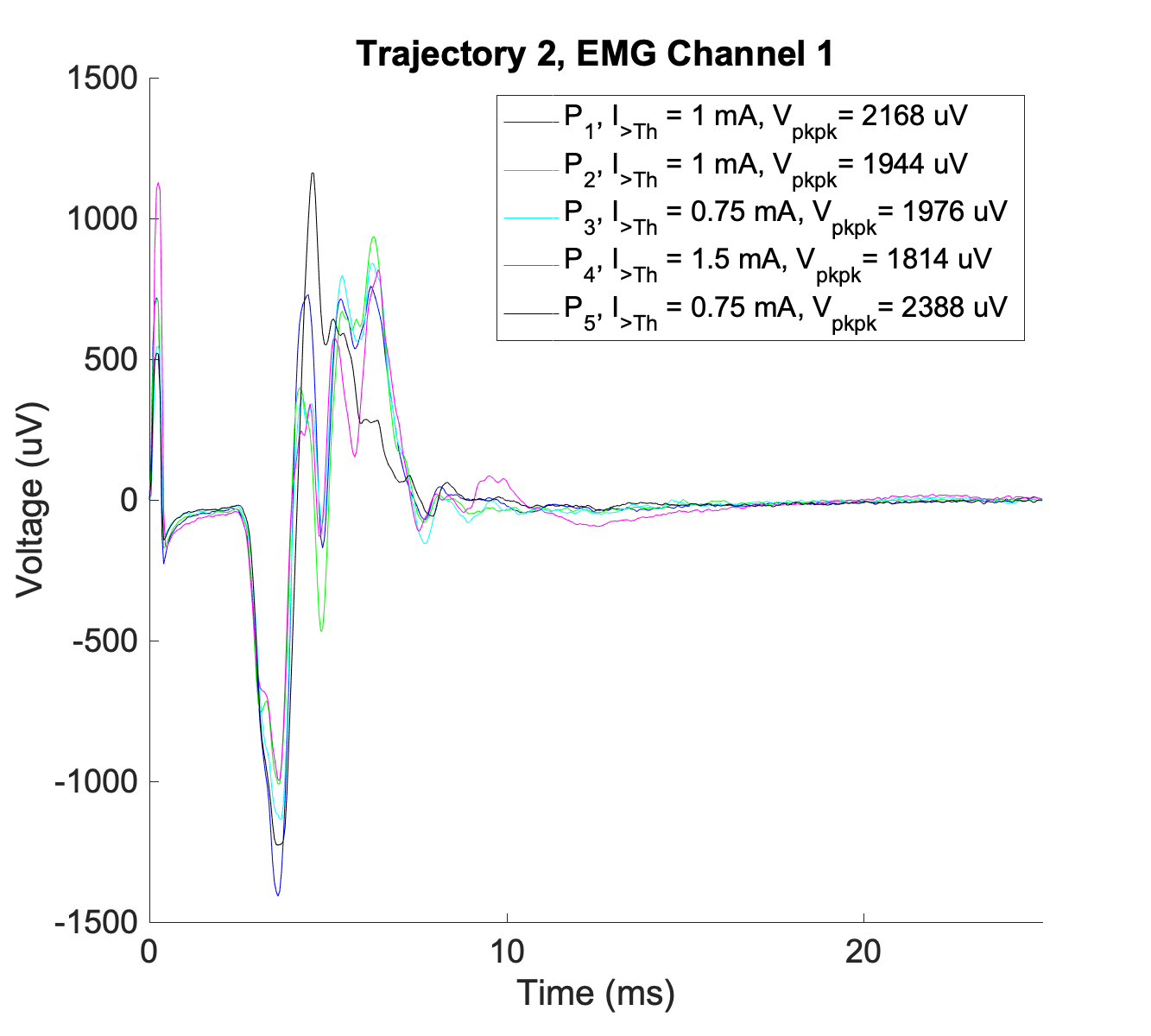

Supplement: Supplementary Data Sheet 1 — Overview of recorded electromyography data showing CMAP responses to the stimulation intensity ramp at each measurement point for the monopolar stimulation. A graph with maximum CMAP responses of monopolar stimulation for each trajectory is depicted. A Summary report (Subject 1, 2, 3.docx) of CMAP responses (for monopolar stimulation) in trajectories with potential FN damage are presented. Data sets of bipolar stimulation can be shared if the reader is interested (see Data Availability Statement). [file Data_Sheet_1.ZIP › Analysis_EMG_Amplitude_Changes/EMGAmp_OutputData/Subject2/Subject2_Traj2_AllPoints_EMG_CH1.png]

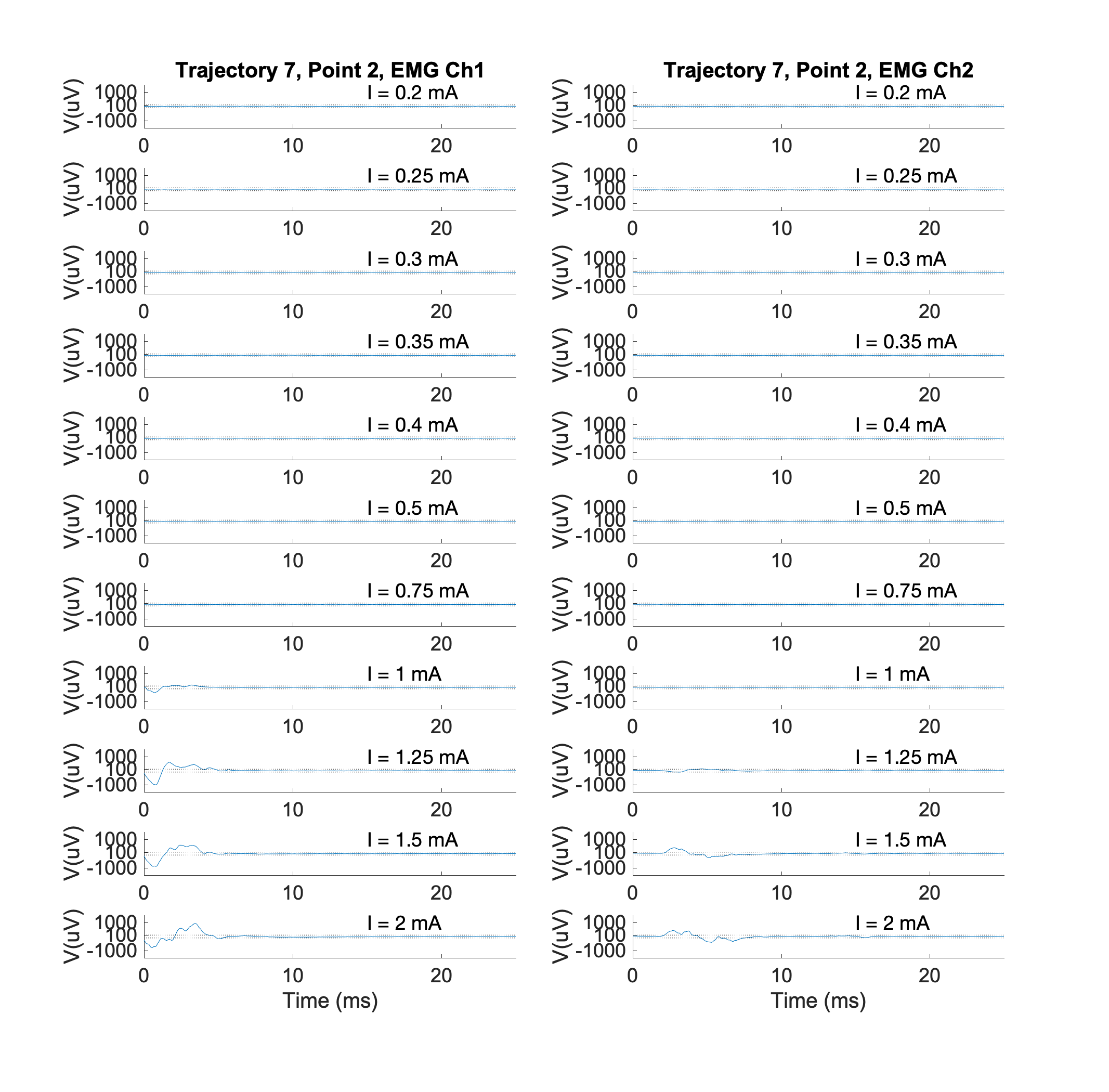

Supplement: Supplementary Data Sheet 1 — Overview of recorded electromyography data showing CMAP responses to the stimulation intensity ramp at each measurement point for the monopolar stimulation. A graph with maximum CMAP responses of monopolar stimulation for each trajectory is depicted. A Summary report (Subject 1, 2, 3.docx) of CMAP responses (for monopolar stimulation) in trajectories with potential FN damage are presented. Data sets of bipolar stimulation can be shared if the reader is interested (see Data Availability Statement). [file Data_Sheet_1.ZIP › Analysis_EMG_Amplitude_Changes/EMGAmp_OutputData/Subject2/Subject2_Traj7_Point2_EMGepochs.png]

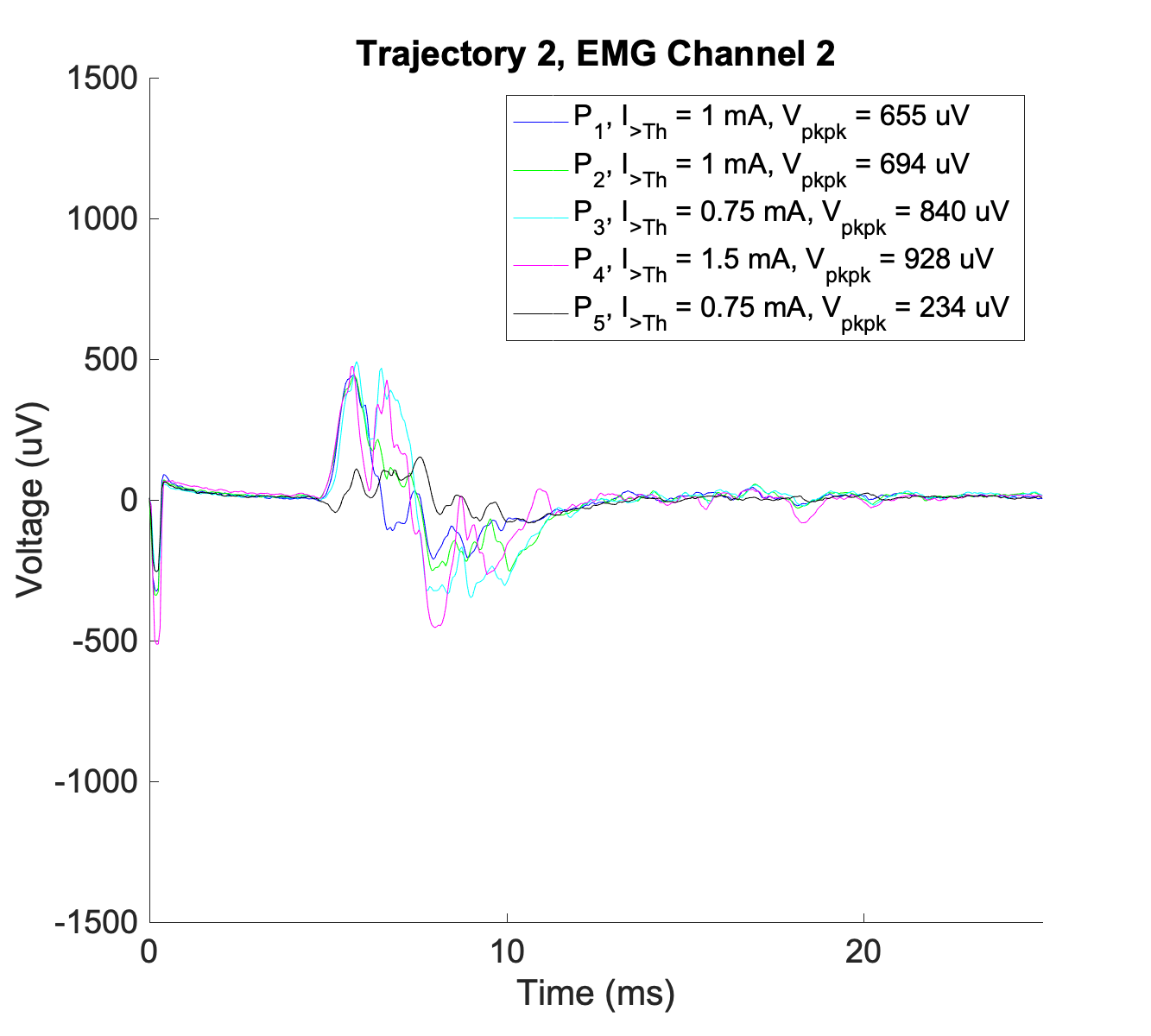

Supplement: Supplementary Data Sheet 1 — Overview of recorded electromyography data showing CMAP responses to the stimulation intensity ramp at each measurement point for the monopolar stimulation. A graph with maximum CMAP responses of monopolar stimulation for each trajectory is depicted. A Summary report (Subject 1, 2, 3.docx) of CMAP responses (for monopolar stimulation) in trajectories with potential FN damage are presented. Data sets of bipolar stimulation can be shared if the reader is interested (see Data Availability Statement). [file Data_Sheet_1.ZIP › Analysis_EMG_Amplitude_Changes/EMGAmp_OutputData/Subject2/Subject2_Traj2_AllPoints_EMG_CH2.png]

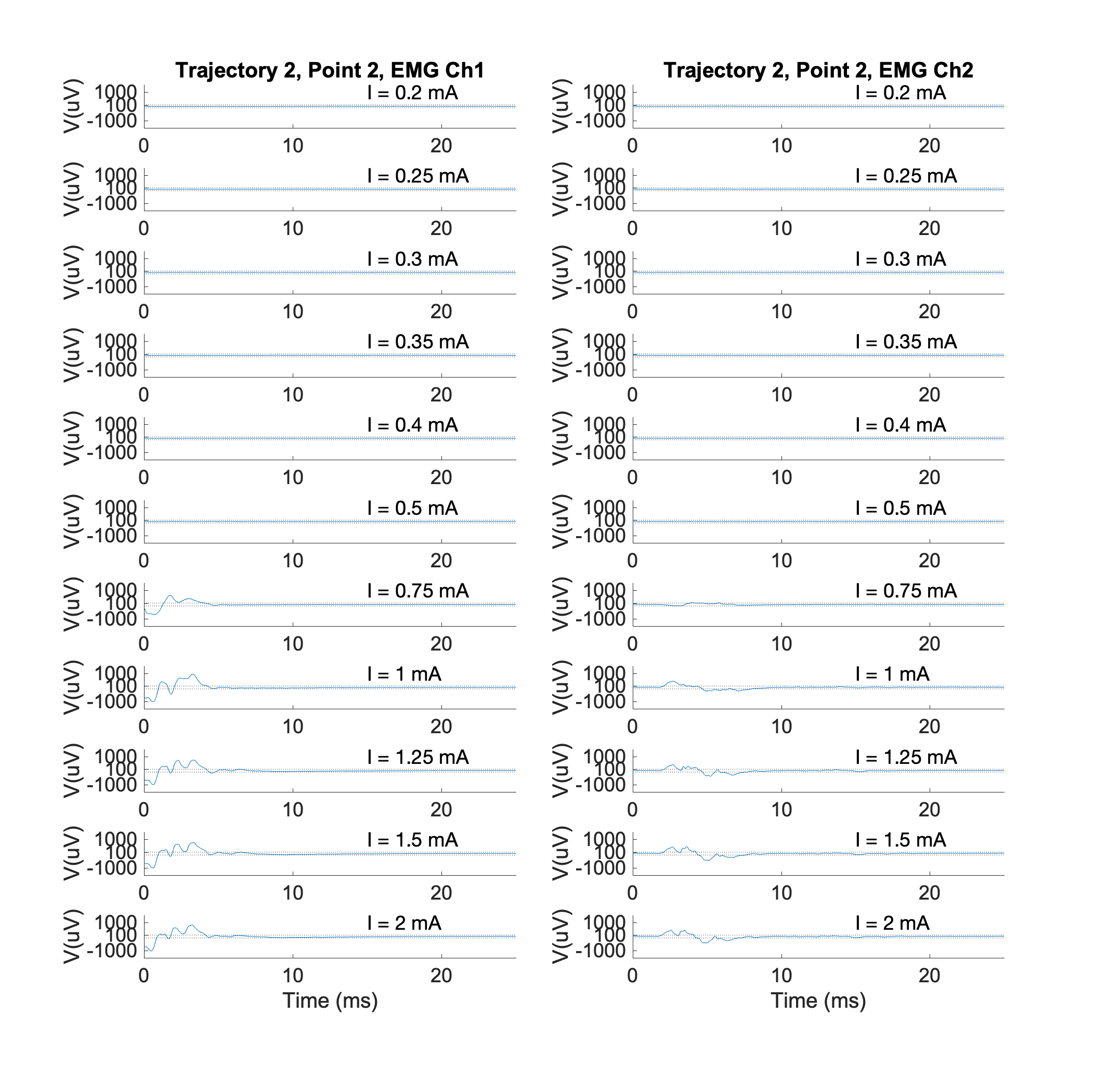

Supplement: Supplementary Data Sheet 1 — Overview of recorded electromyography data showing CMAP responses to the stimulation intensity ramp at each measurement point for the monopolar stimulation. A graph with maximum CMAP responses of monopolar stimulation for each trajectory is depicted. A Summary report (Subject 1, 2, 3.docx) of CMAP responses (for monopolar stimulation) in trajectories with potential FN damage are presented. Data sets of bipolar stimulation can be shared if the reader is interested (see Data Availability Statement). [file Data_Sheet_1.ZIP › Analysis_EMG_Amplitude_Changes/EMGAmp_OutputData/Subject2/Subject2_Traj2_Point2_EMGepochs.png]

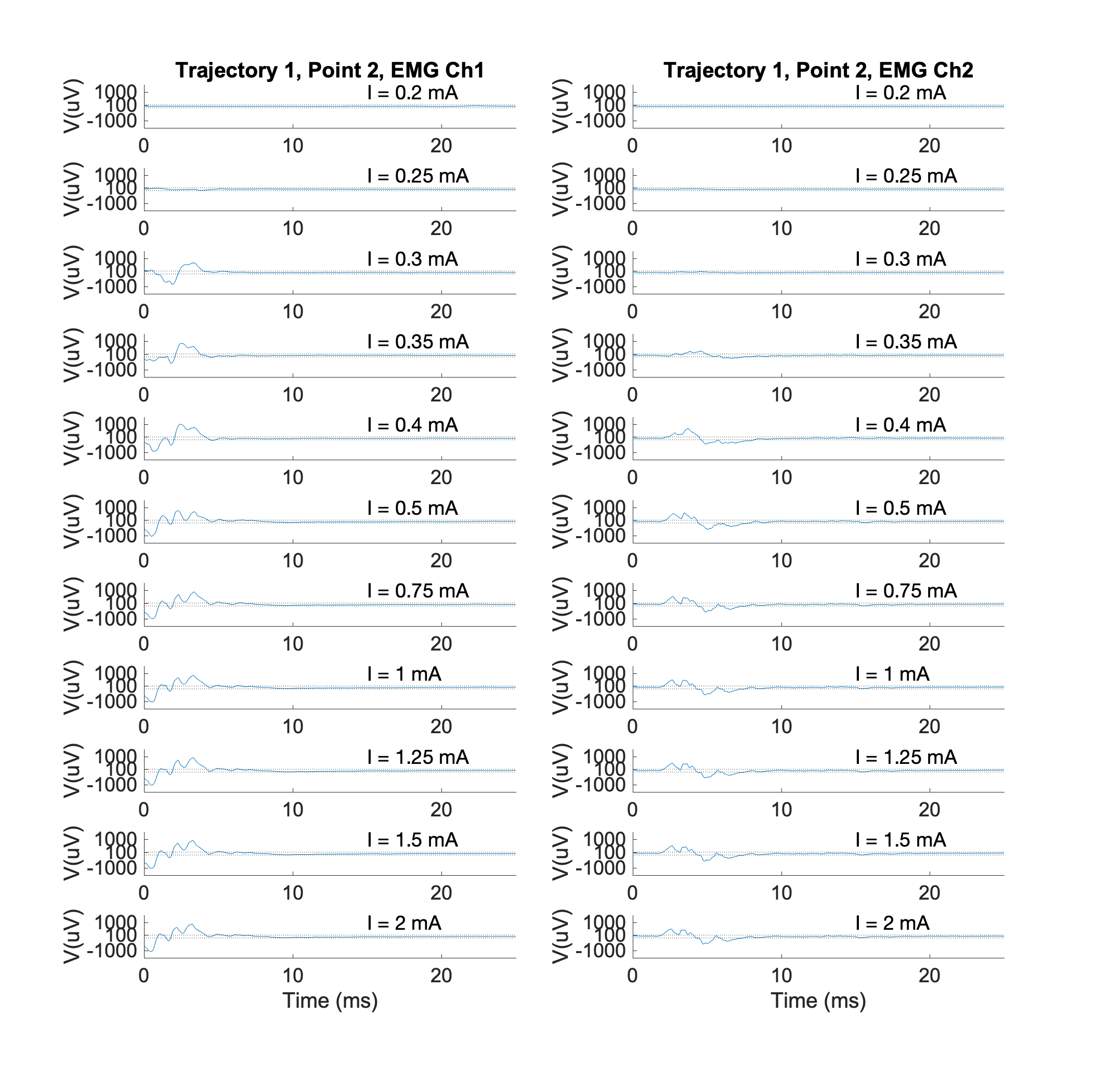

Supplement: Supplementary Data Sheet 1 — Overview of recorded electromyography data showing CMAP responses to the stimulation intensity ramp at each measurement point for the monopolar stimulation. A graph with maximum CMAP responses of monopolar stimulation for each trajectory is depicted. A Summary report (Subject 1, 2, 3.docx) of CMAP responses (for monopolar stimulation) in trajectories with potential FN damage are presented. Data sets of bipolar stimulation can be shared if the reader is interested (see Data Availability Statement). [file Data_Sheet_1.ZIP › Analysis_EMG_Amplitude_Changes/EMGAmp_OutputData/Subject2/Subject2_Traj1_Point2_EMGepochs.png]

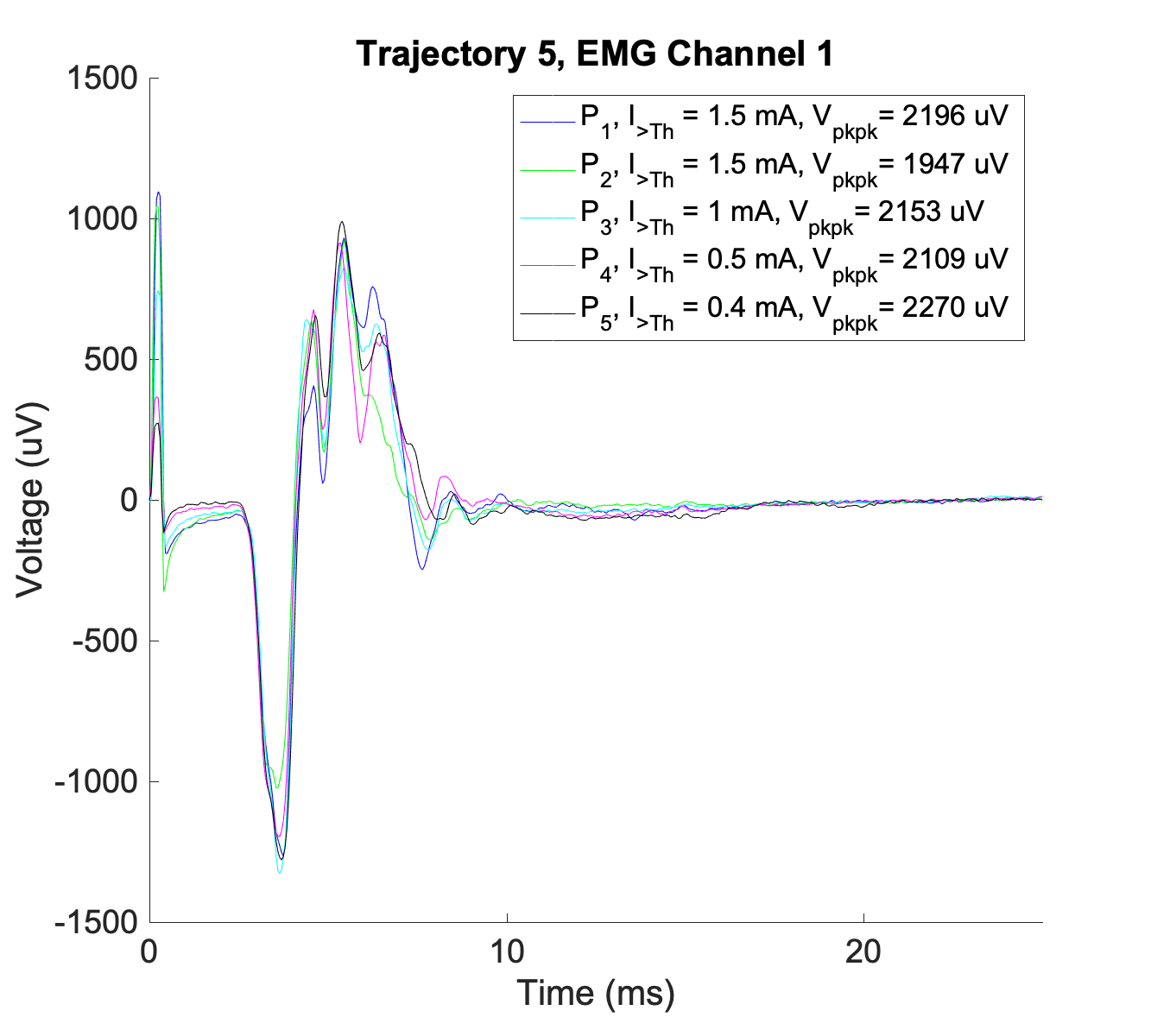

Supplement: Supplementary Data Sheet 1 — Overview of recorded electromyography data showing CMAP responses to the stimulation intensity ramp at each measurement point for the monopolar stimulation. A graph with maximum CMAP responses of monopolar stimulation for each trajectory is depicted. A Summary report (Subject 1, 2, 3.docx) of CMAP responses (for monopolar stimulation) in trajectories with potential FN damage are presented. Data sets of bipolar stimulation can be shared if the reader is interested (see Data Availability Statement). [file Data_Sheet_1.ZIP › Analysis_EMG_Amplitude_Changes/EMGAmp_OutputData/Subject2/Subject2_Traj5_AllPoints_EMG_CH1.png]

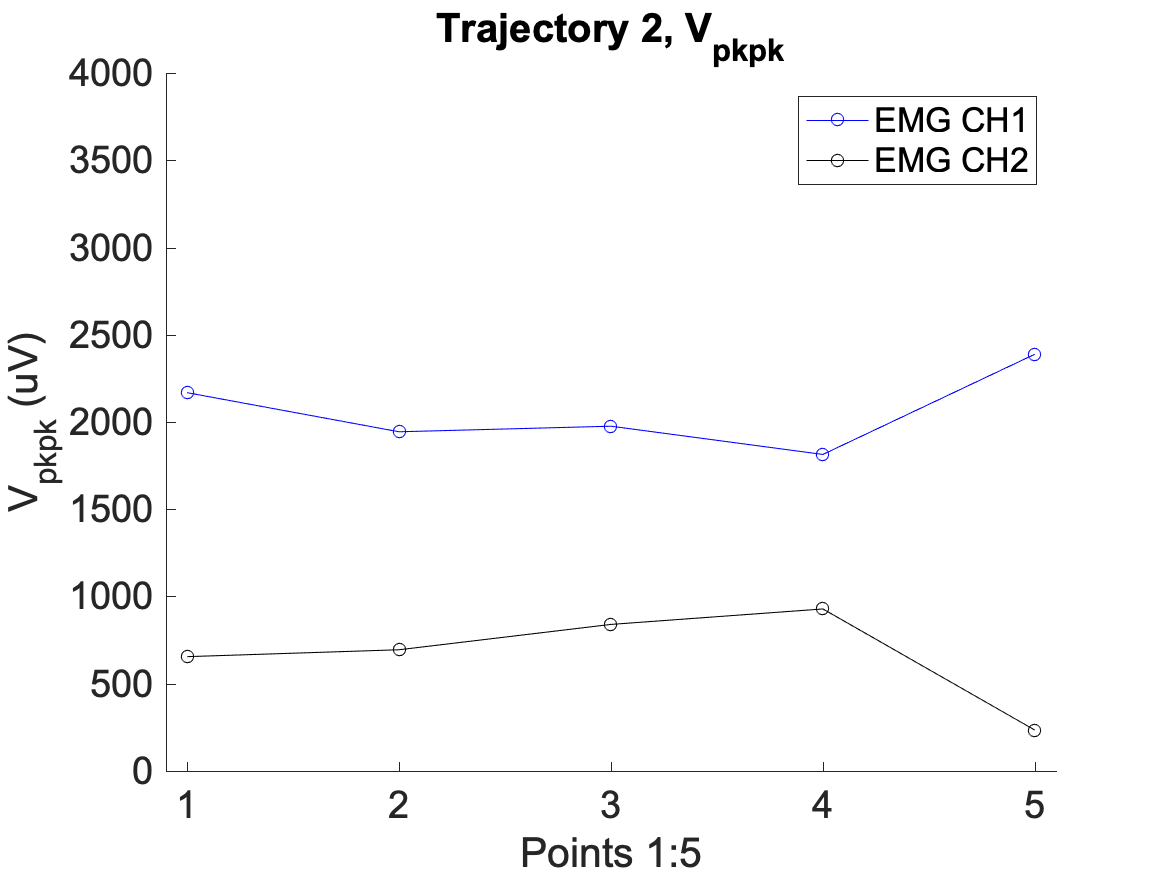

Supplement: Supplementary Data Sheet 1 — Overview of recorded electromyography data showing CMAP responses to the stimulation intensity ramp at each measurement point for the monopolar stimulation. A graph with maximum CMAP responses of monopolar stimulation for each trajectory is depicted. A Summary report (Subject 1, 2, 3.docx) of CMAP responses (for monopolar stimulation) in trajectories with potential FN damage are presented. Data sets of bipolar stimulation can be shared if the reader is interested (see Data Availability Statement). [file Data_Sheet_1.ZIP › Analysis_EMG_Amplitude_Changes/EMGAmp_OutputData/Subject2/Subject2_Traj2_Vpkk.png]

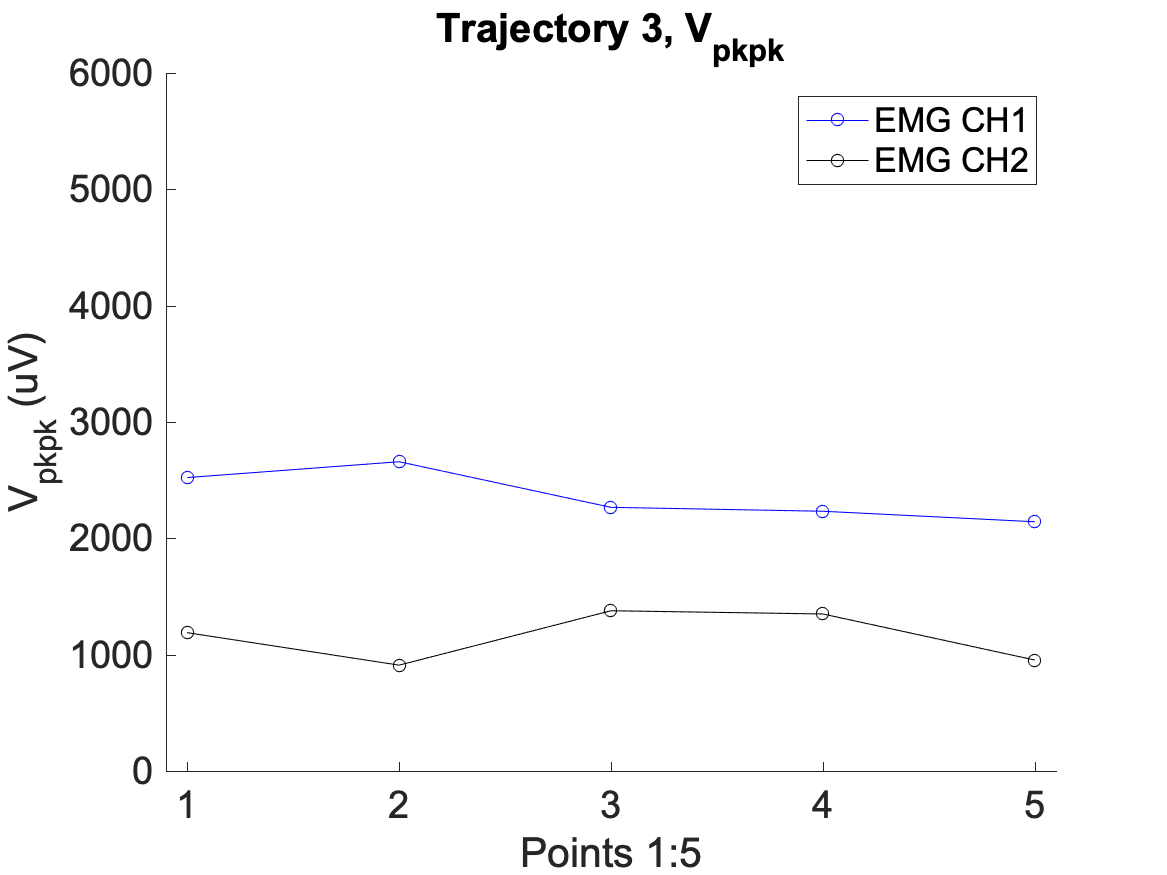

Supplement: Supplementary Data Sheet 1 — Overview of recorded electromyography data showing CMAP responses to the stimulation intensity ramp at each measurement point for the monopolar stimulation. A graph with maximum CMAP responses of monopolar stimulation for each trajectory is depicted. A Summary report (Subject 1, 2, 3.docx) of CMAP responses (for monopolar stimulation) in trajectories with potential FN damage are presented. Data sets of bipolar stimulation can be shared if the reader is interested (see Data Availability Statement). [file Data_Sheet_1.ZIP › Analysis_EMG_Amplitude_Changes/EMGAmp_OutputData/Subject2/Subject2_Traj3_Vpkk.png]

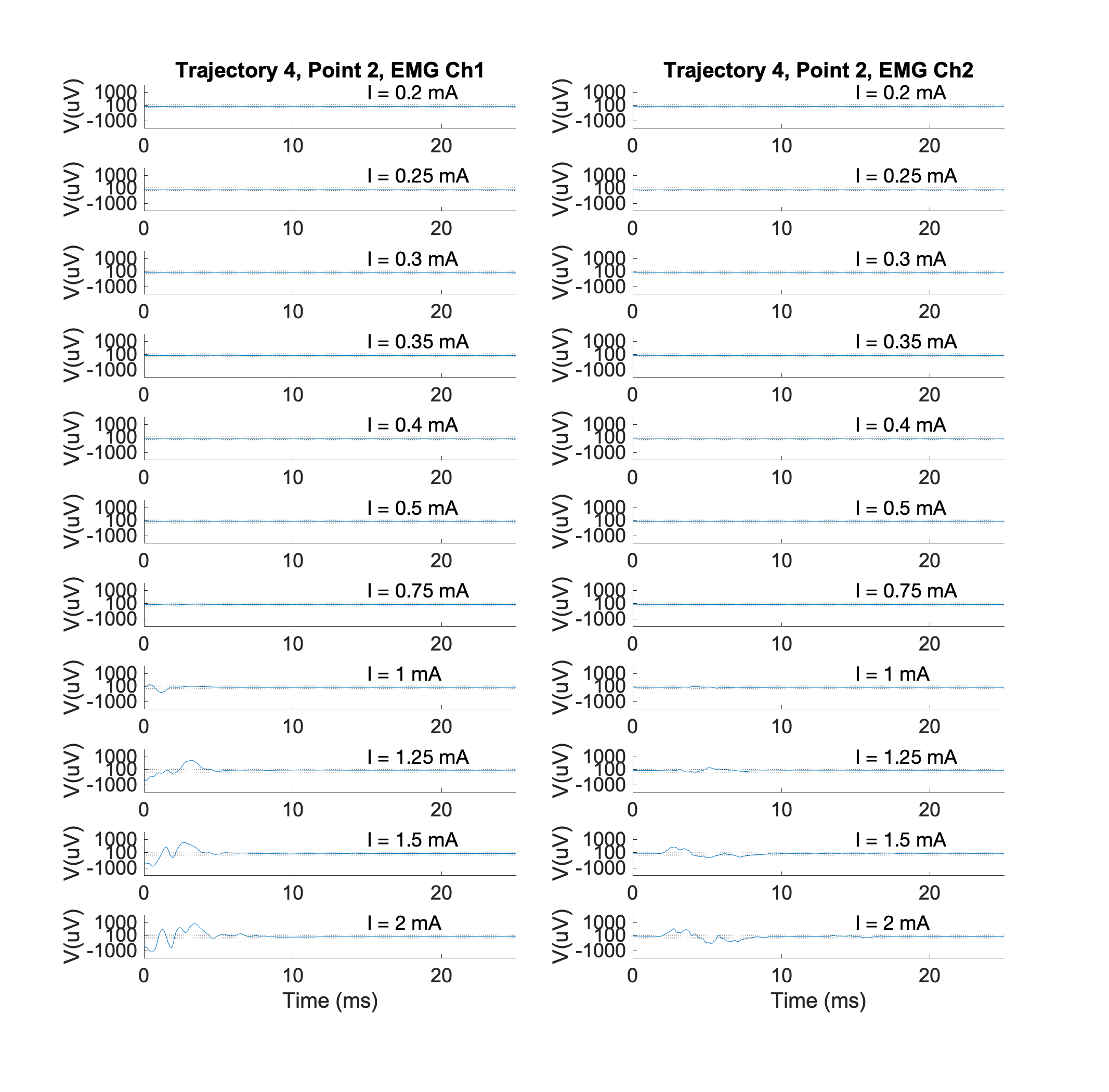

Supplement: Supplementary Data Sheet 1 — Overview of recorded electromyography data showing CMAP responses to the stimulation intensity ramp at each measurement point for the monopolar stimulation. A graph with maximum CMAP responses of monopolar stimulation for each trajectory is depicted. A Summary report (Subject 1, 2, 3.docx) of CMAP responses (for monopolar stimulation) in trajectories with potential FN damage are presented. Data sets of bipolar stimulation can be shared if the reader is interested (see Data Availability Statement). [file Data_Sheet_1.ZIP › Analysis_EMG_Amplitude_Changes/EMGAmp_OutputData/Subject2/Subject2_Traj4_Point2_EMGepochs.png]

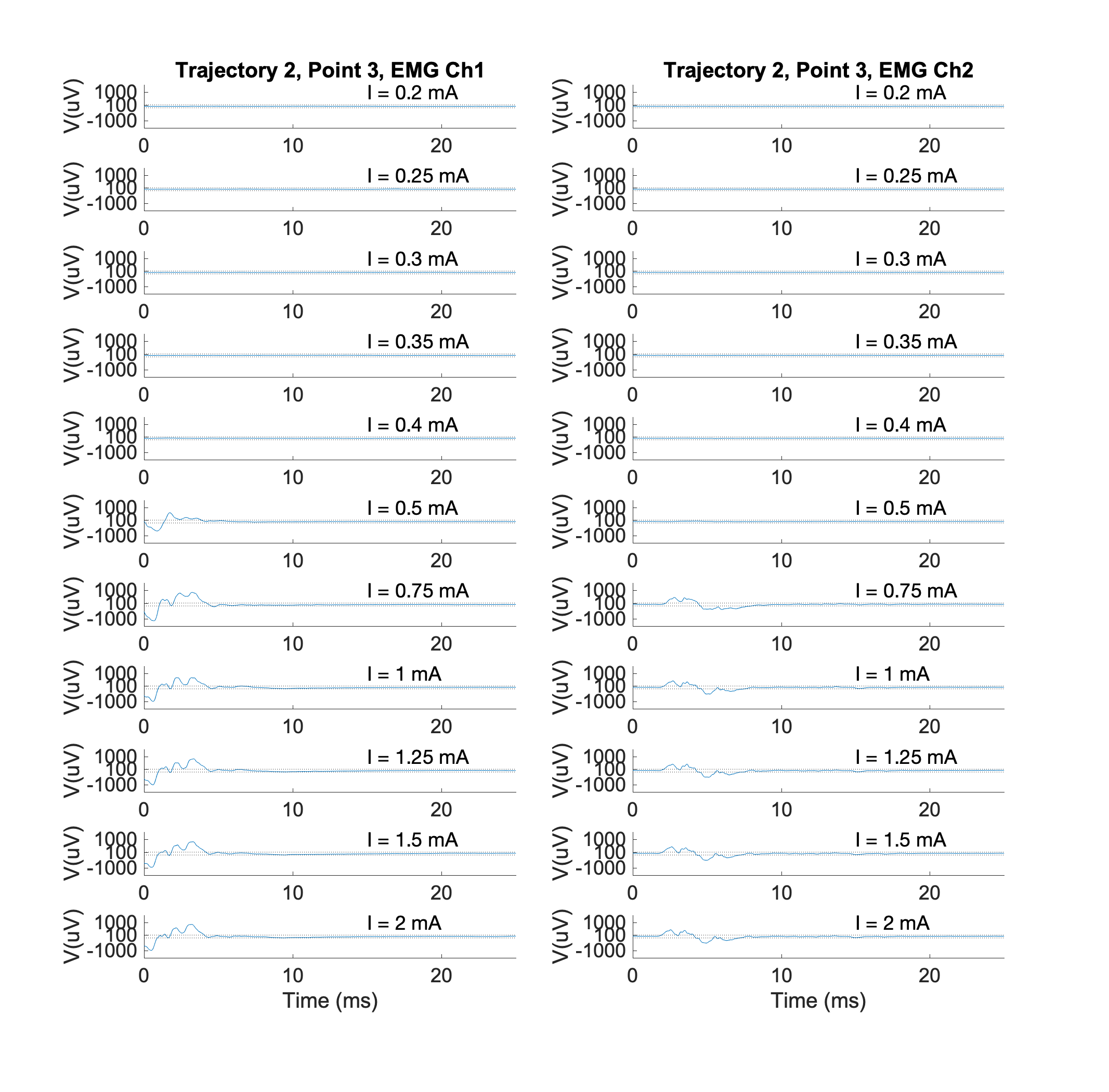

Supplement: Supplementary Data Sheet 1 — Overview of recorded electromyography data showing CMAP responses to the stimulation intensity ramp at each measurement point for the monopolar stimulation. A graph with maximum CMAP responses of monopolar stimulation for each trajectory is depicted. A Summary report (Subject 1, 2, 3.docx) of CMAP responses (for monopolar stimulation) in trajectories with potential FN damage are presented. Data sets of bipolar stimulation can be shared if the reader is interested (see Data Availability Statement). [file Data_Sheet_1.ZIP › Analysis_EMG_Amplitude_Changes/EMGAmp_OutputData/Subject2/Subject2_Traj2_Point3_EMGepochs.png]

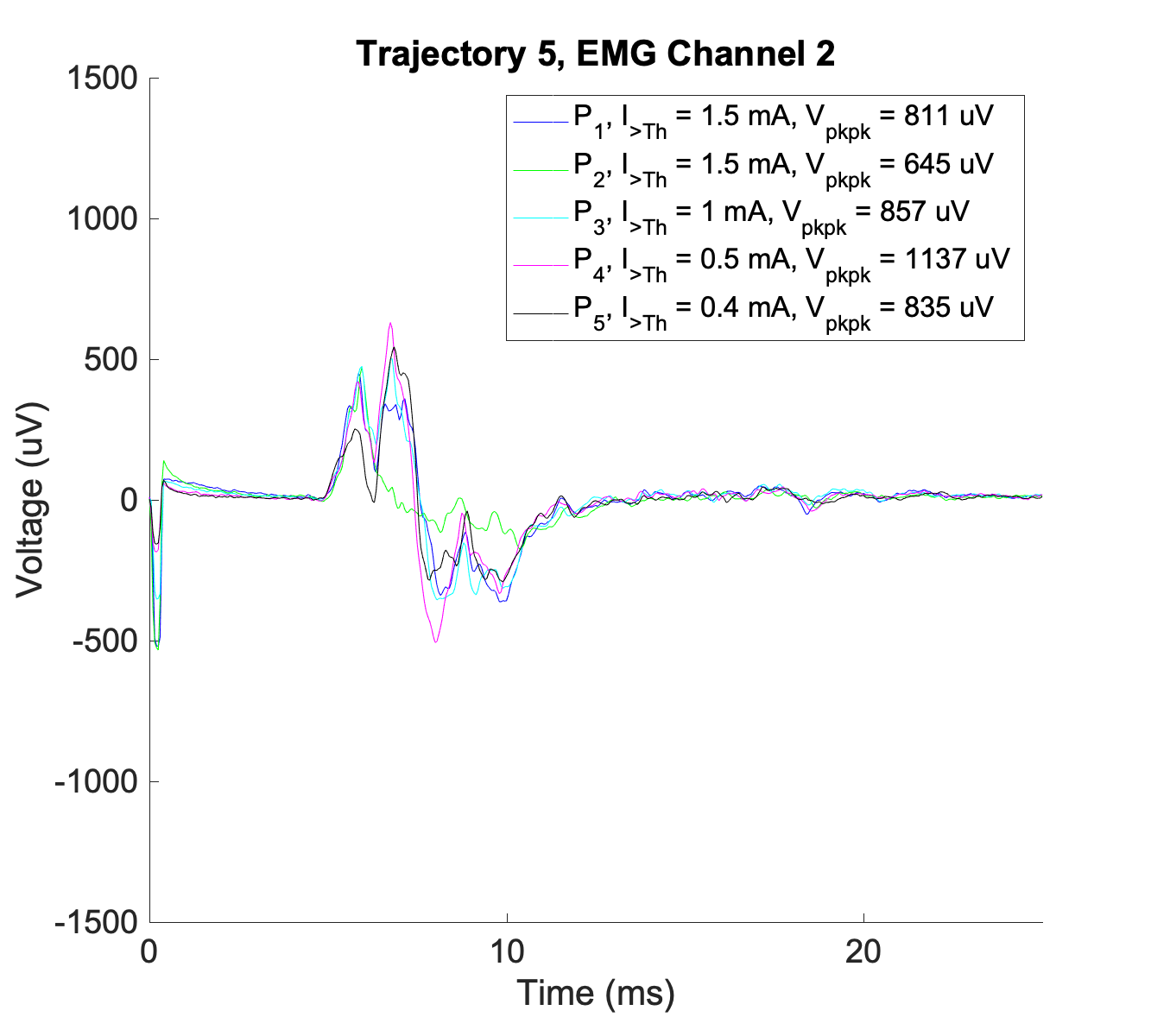

Supplement: Supplementary Data Sheet 1 — Overview of recorded electromyography data showing CMAP responses to the stimulation intensity ramp at each measurement point for the monopolar stimulation. A graph with maximum CMAP responses of monopolar stimulation for each trajectory is depicted. A Summary report (Subject 1, 2, 3.docx) of CMAP responses (for monopolar stimulation) in trajectories with potential FN damage are presented. Data sets of bipolar stimulation can be shared if the reader is interested (see Data Availability Statement). [file Data_Sheet_1.ZIP › Analysis_EMG_Amplitude_Changes/EMGAmp_OutputData/Subject2/Subject2_Traj5_AllPoints_EMG_CH2.png]

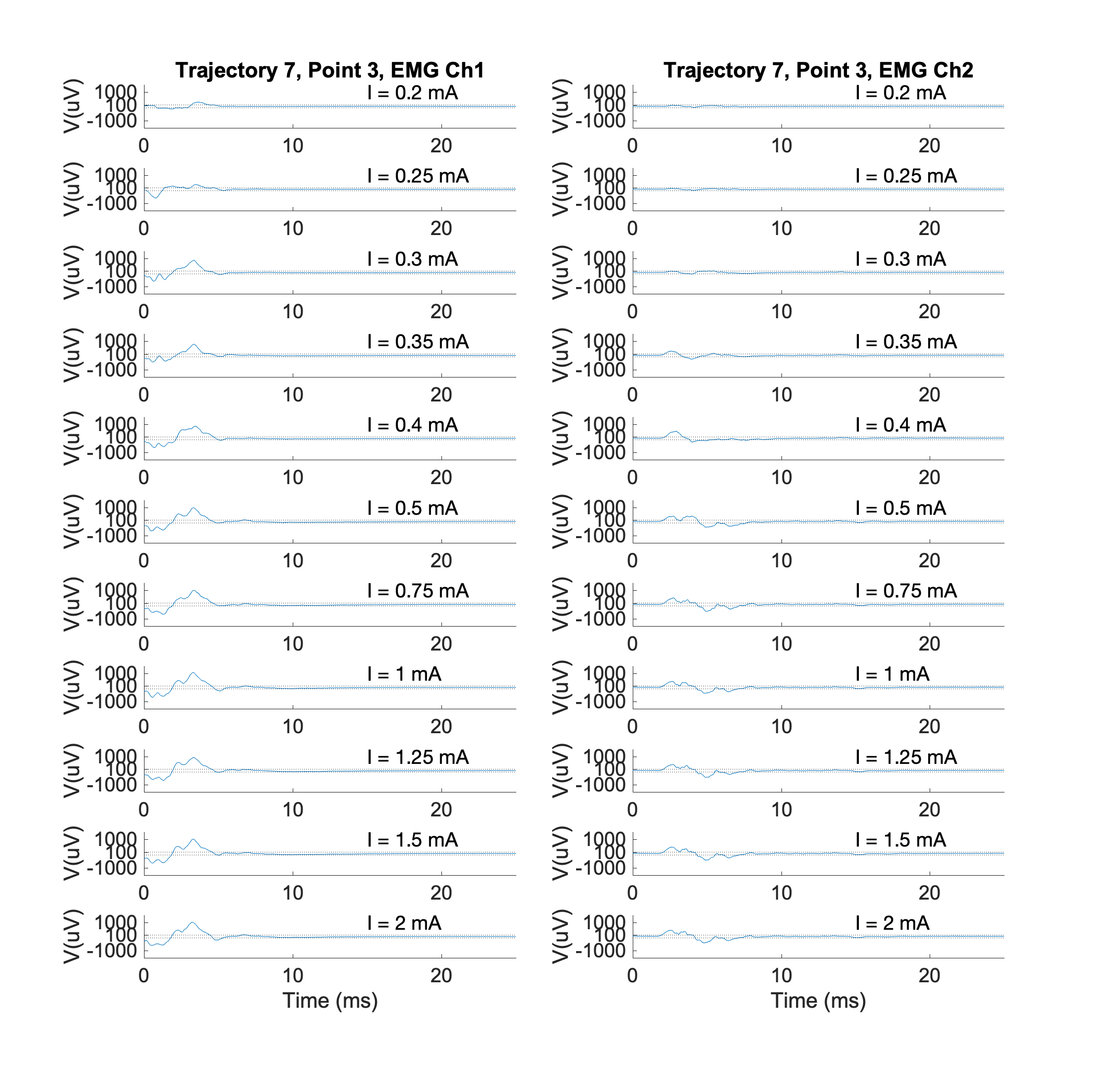

Supplement: Supplementary Data Sheet 1 — Overview of recorded electromyography data showing CMAP responses to the stimulation intensity ramp at each measurement point for the monopolar stimulation. A graph with maximum CMAP responses of monopolar stimulation for each trajectory is depicted. A Summary report (Subject 1, 2, 3.docx) of CMAP responses (for monopolar stimulation) in trajectories with potential FN damage are presented. Data sets of bipolar stimulation can be shared if the reader is interested (see Data Availability Statement). [file Data_Sheet_1.ZIP › Analysis_EMG_Amplitude_Changes/EMGAmp_OutputData/Subject2/Subject2_Traj7_Point3_EMGepochs.png]

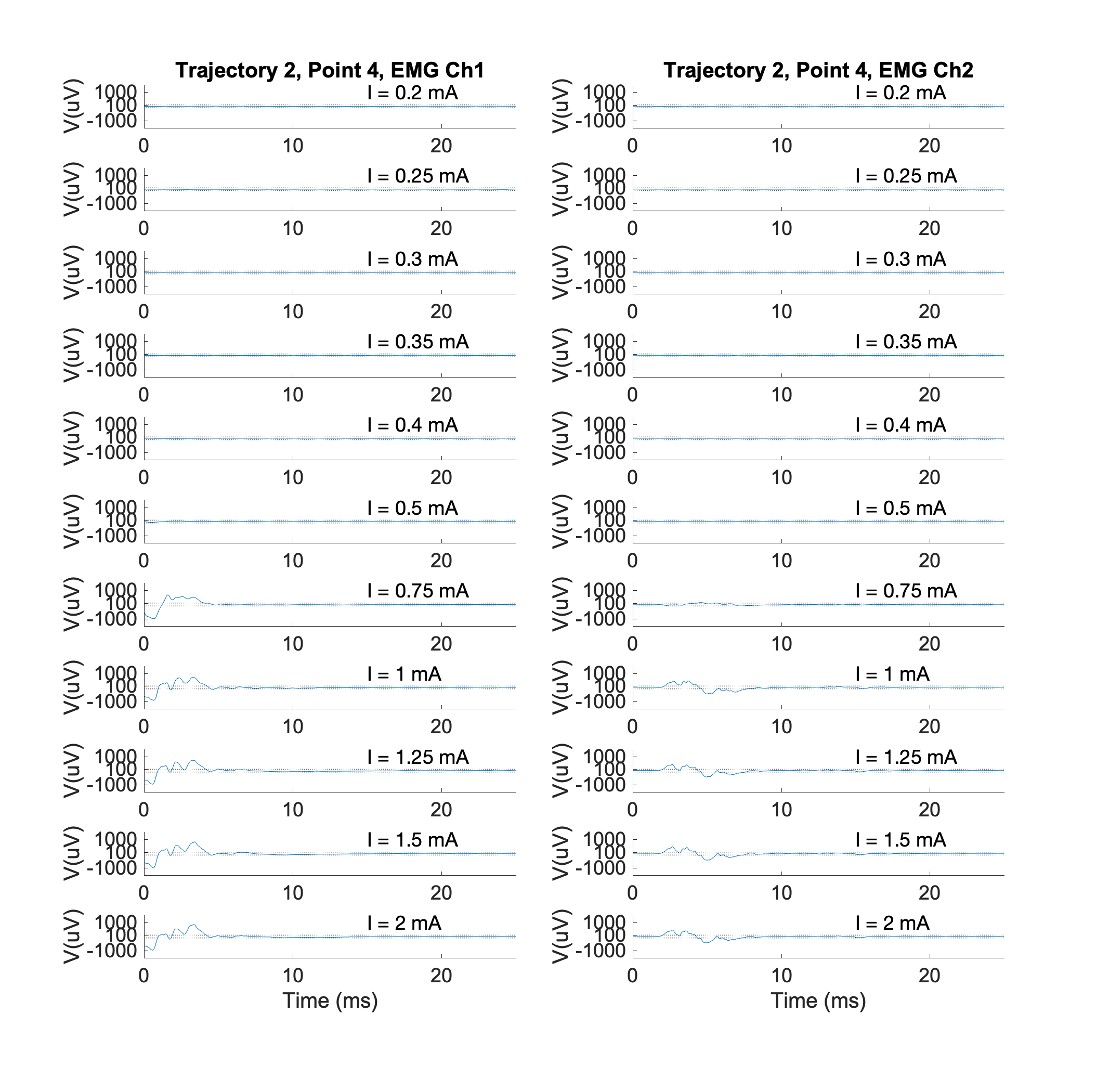

Supplement: Supplementary Data Sheet 1 — Overview of recorded electromyography data showing CMAP responses to the stimulation intensity ramp at each measurement point for the monopolar stimulation. A graph with maximum CMAP responses of monopolar stimulation for each trajectory is depicted. A Summary report (Subject 1, 2, 3.docx) of CMAP responses (for monopolar stimulation) in trajectories with potential FN damage are presented. Data sets of bipolar stimulation can be shared if the reader is interested (see Data Availability Statement). [file Data_Sheet_1.ZIP › Analysis_EMG_Amplitude_Changes/EMGAmp_OutputData/Subject2/Subject2_Traj2_Point4_EMGepochs.png]

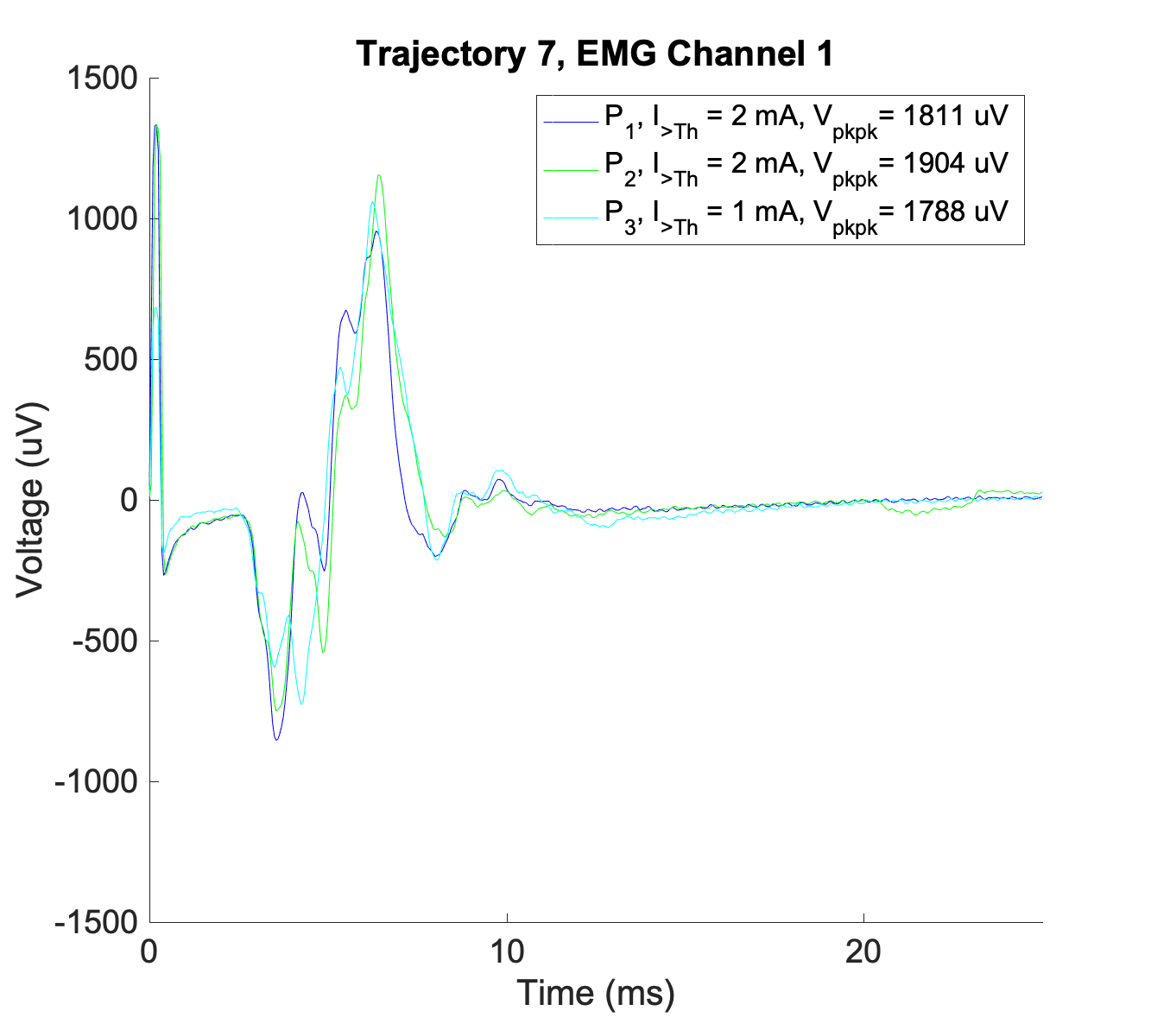

Supplement: Supplementary Data Sheet 1 — Overview of recorded electromyography data showing CMAP responses to the stimulation intensity ramp at each measurement point for the monopolar stimulation. A graph with maximum CMAP responses of monopolar stimulation for each trajectory is depicted. A Summary report (Subject 1, 2, 3.docx) of CMAP responses (for monopolar stimulation) in trajectories with potential FN damage are presented. Data sets of bipolar stimulation can be shared if the reader is interested (see Data Availability Statement). [file Data_Sheet_1.ZIP › Analysis_EMG_Amplitude_Changes/EMGAmp_OutputData/Subject2/Subject2_Traj7_AllPoints_EMG_CH1.png]

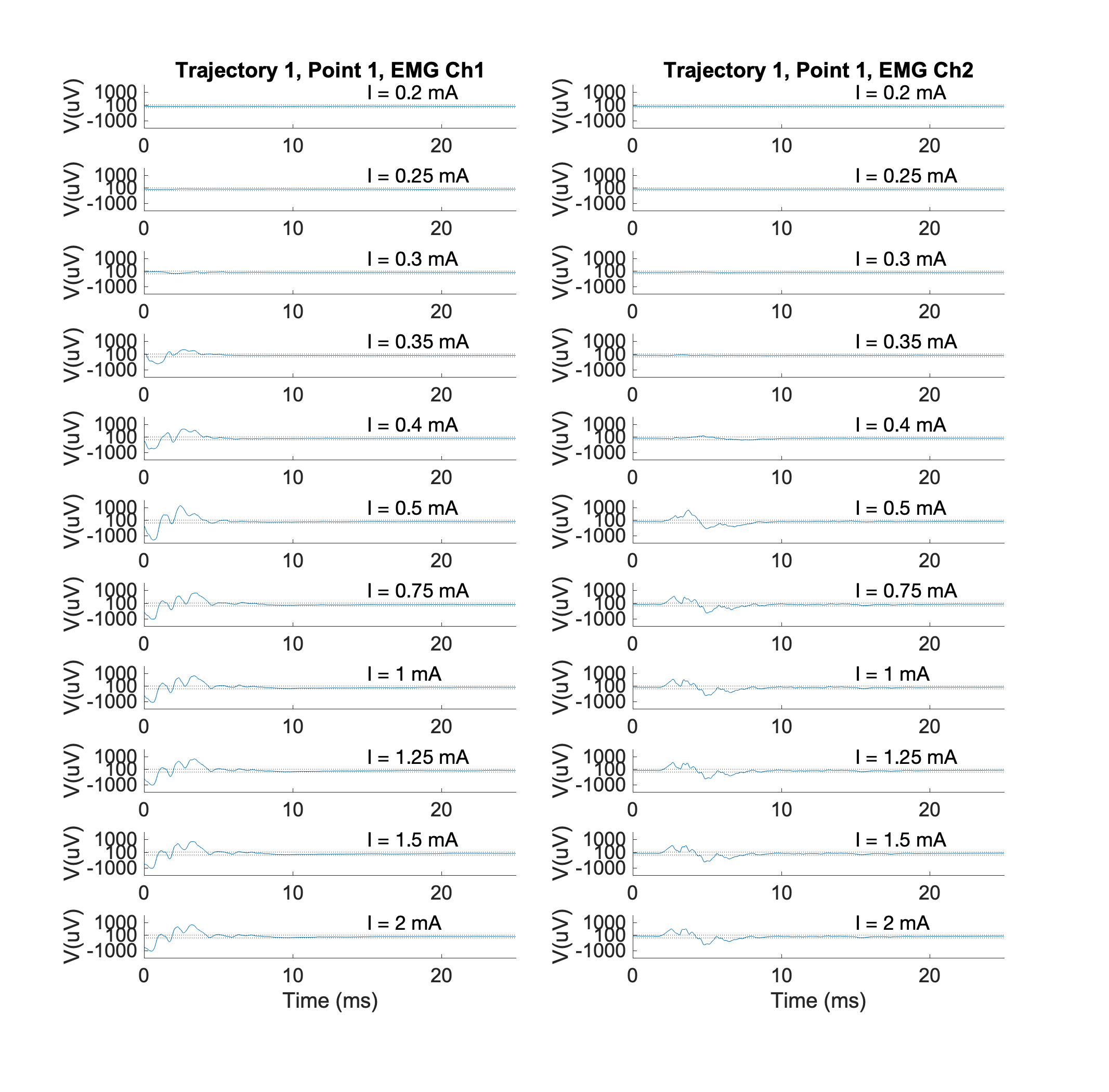

Supplement: Supplementary Data Sheet 1 — Overview of recorded electromyography data showing CMAP responses to the stimulation intensity ramp at each measurement point for the monopolar stimulation. A graph with maximum CMAP responses of monopolar stimulation for each trajectory is depicted. A Summary report (Subject 1, 2, 3.docx) of CMAP responses (for monopolar stimulation) in trajectories with potential FN damage are presented. Data sets of bipolar stimulation can be shared if the reader is interested (see Data Availability Statement). [file Data_Sheet_1.ZIP › Analysis_EMG_Amplitude_Changes/EMGAmp_OutputData/Subject2/Subject2_Traj1_Point1_EMGepochs.png]

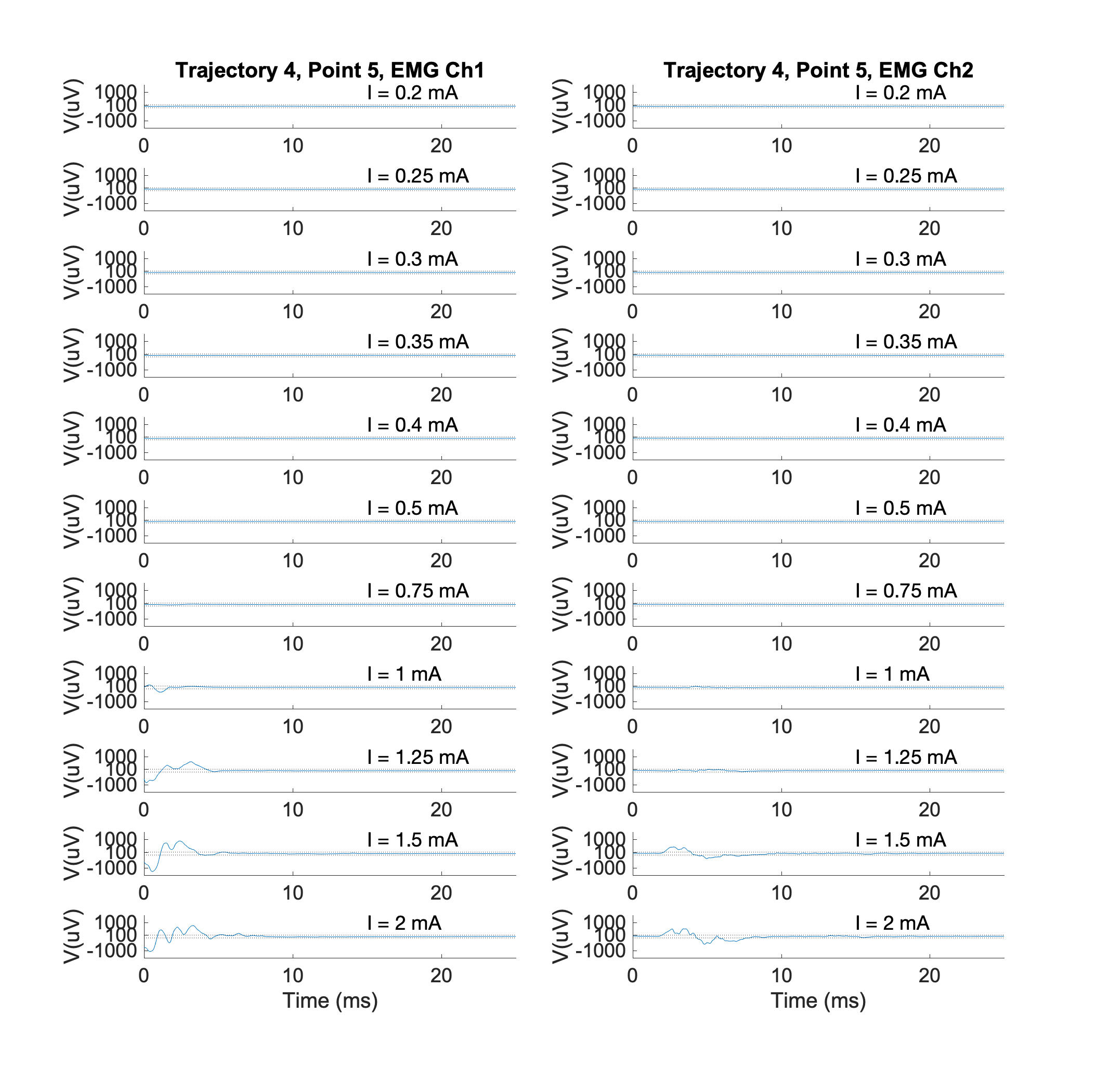

Supplement: Supplementary Data Sheet 1 — Overview of recorded electromyography data showing CMAP responses to the stimulation intensity ramp at each measurement point for the monopolar stimulation. A graph with maximum CMAP responses of monopolar stimulation for each trajectory is depicted. A Summary report (Subject 1, 2, 3.docx) of CMAP responses (for monopolar stimulation) in trajectories with potential FN damage are presented. Data sets of bipolar stimulation can be shared if the reader is interested (see Data Availability Statement). [file Data_Sheet_1.ZIP › Analysis_EMG_Amplitude_Changes/EMGAmp_OutputData/Subject2/Subject2_Traj4_Point5_EMGepochs.png]

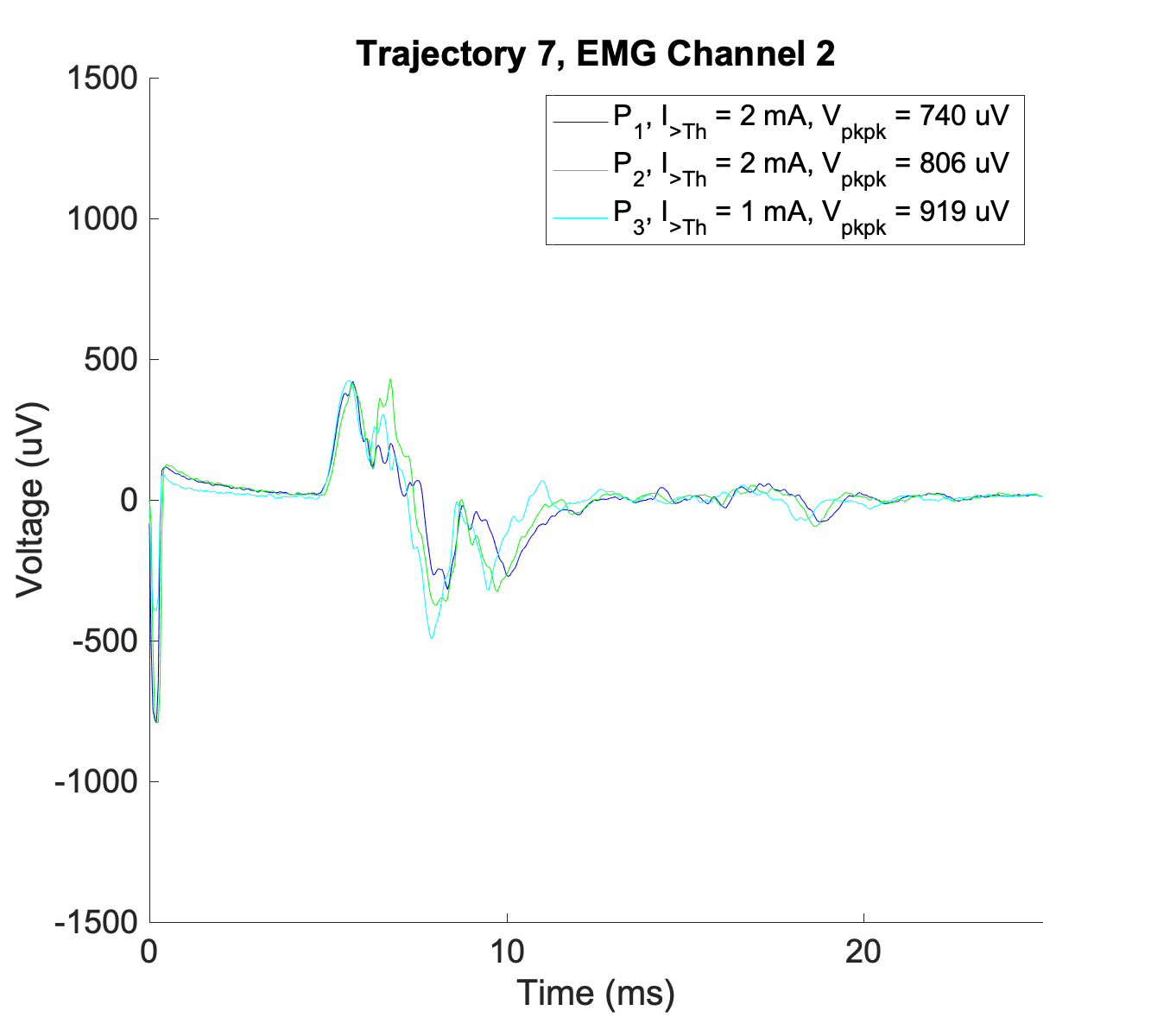

Supplement: Supplementary Data Sheet 1 — Overview of recorded electromyography data showing CMAP responses to the stimulation intensity ramp at each measurement point for the monopolar stimulation. A graph with maximum CMAP responses of monopolar stimulation for each trajectory is depicted. A Summary report (Subject 1, 2, 3.docx) of CMAP responses (for monopolar stimulation) in trajectories with potential FN damage are presented. Data sets of bipolar stimulation can be shared if the reader is interested (see Data Availability Statement). [file Data_Sheet_1.ZIP › Analysis_EMG_Amplitude_Changes/EMGAmp_OutputData/Subject2/Subject2_Traj7_AllPoints_EMG_CH2.png]

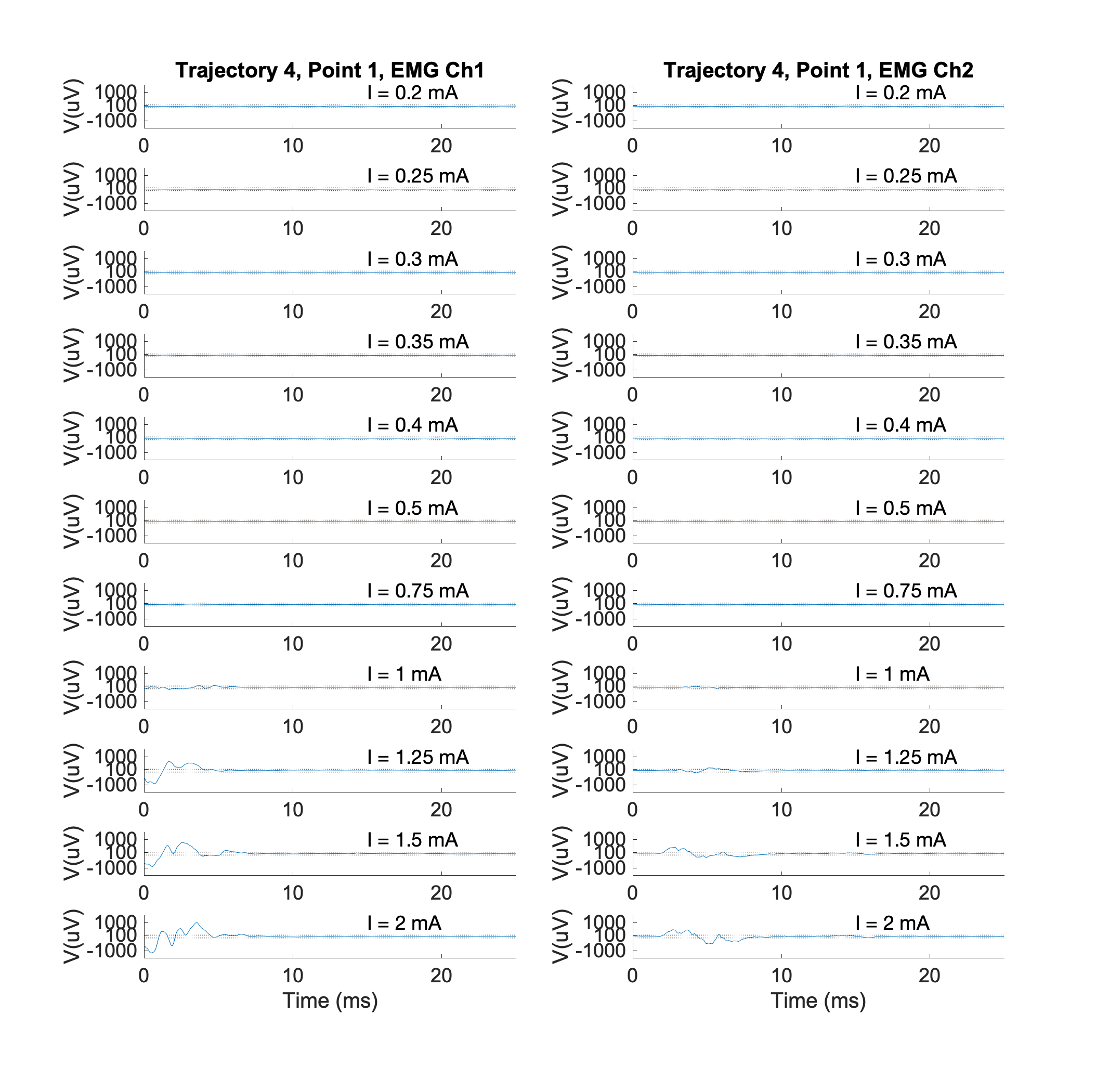

Supplement: Supplementary Data Sheet 1 — Overview of recorded electromyography data showing CMAP responses to the stimulation intensity ramp at each measurement point for the monopolar stimulation. A graph with maximum CMAP responses of monopolar stimulation for each trajectory is depicted. A Summary report (Subject 1, 2, 3.docx) of CMAP responses (for monopolar stimulation) in trajectories with potential FN damage are presented. Data sets of bipolar stimulation can be shared if the reader is interested (see Data Availability Statement). [file Data_Sheet_1.ZIP › Analysis_EMG_Amplitude_Changes/EMGAmp_OutputData/Subject2/Subject2_Traj4_Point1_EMGepochs.png]

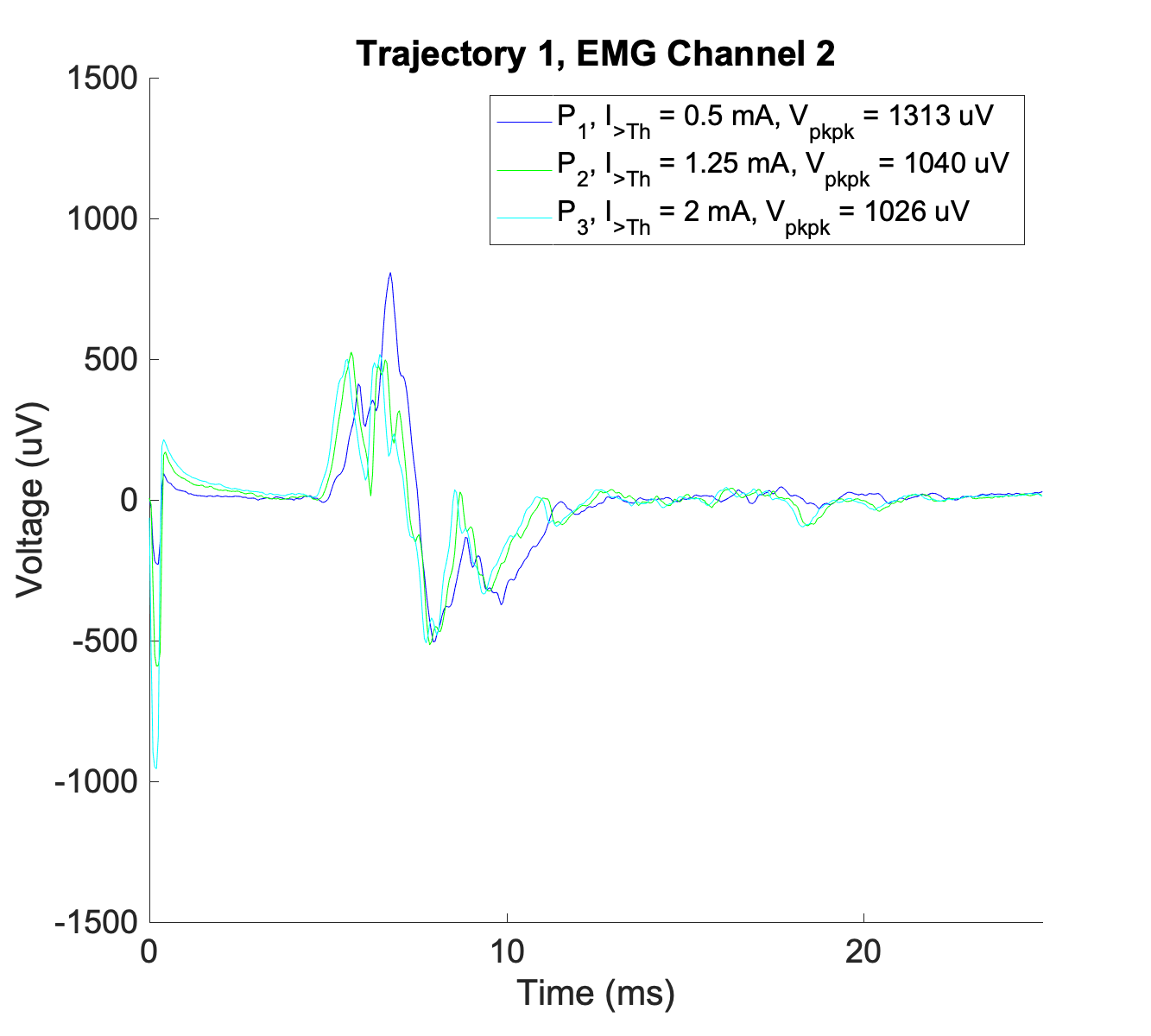

Supplement: Supplementary Data Sheet 1 — Overview of recorded electromyography data showing CMAP responses to the stimulation intensity ramp at each measurement point for the monopolar stimulation. A graph with maximum CMAP responses of monopolar stimulation for each trajectory is depicted. A Summary report (Subject 1, 2, 3.docx) of CMAP responses (for monopolar stimulation) in trajectories with potential FN damage are presented. Data sets of bipolar stimulation can be shared if the reader is interested (see Data Availability Statement). [file Data_Sheet_1.ZIP › Analysis_EMG_Amplitude_Changes/EMGAmp_OutputData/Subject2/Subject2_Traj1_AllPoints_EMG_CH2.png]

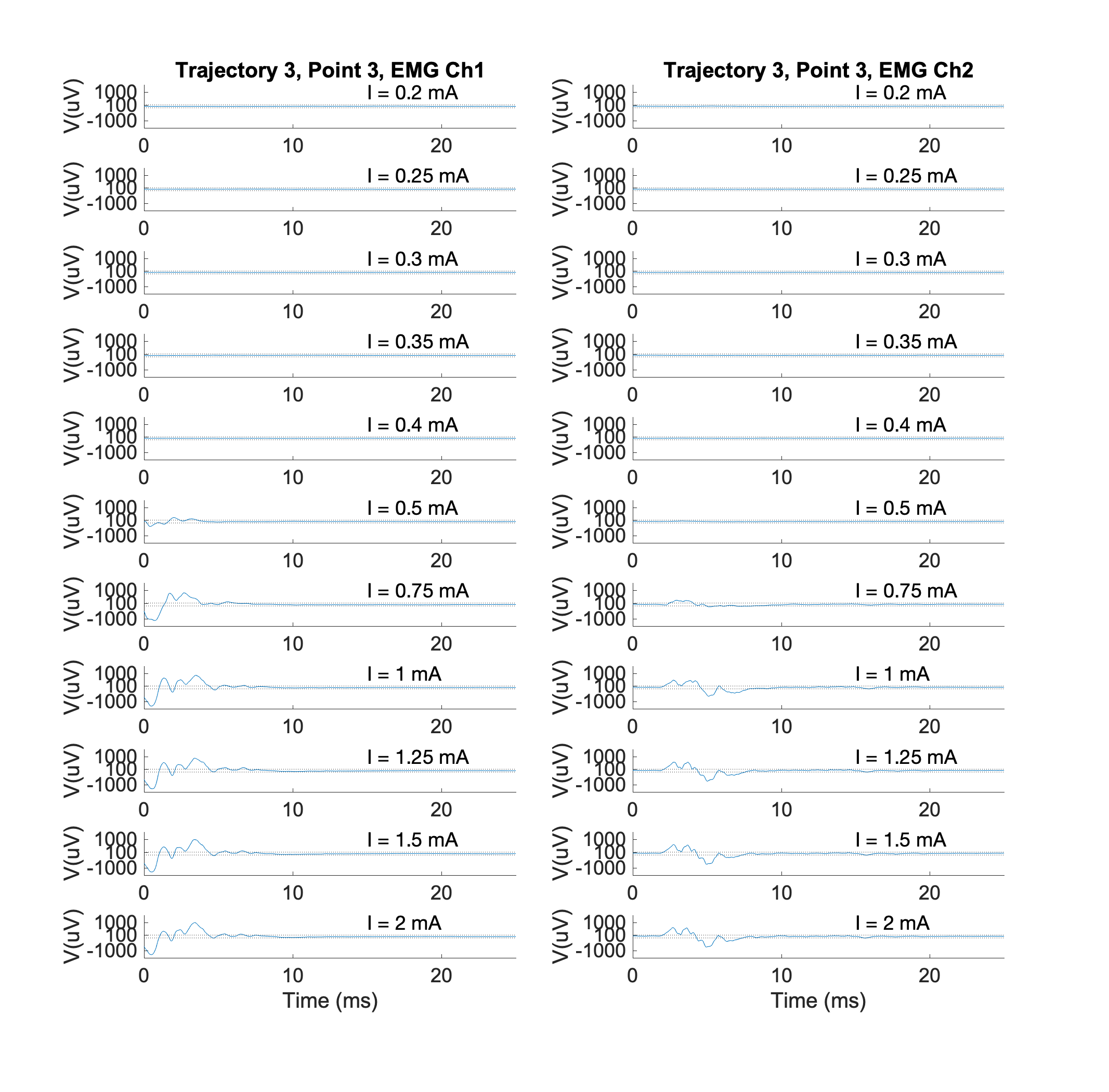

Supplement: Supplementary Data Sheet 1 — Overview of recorded electromyography data showing CMAP responses to the stimulation intensity ramp at each measurement point for the monopolar stimulation. A graph with maximum CMAP responses of monopolar stimulation for each trajectory is depicted. A Summary report (Subject 1, 2, 3.docx) of CMAP responses (for monopolar stimulation) in trajectories with potential FN damage are presented. Data sets of bipolar stimulation can be shared if the reader is interested (see Data Availability Statement). [file Data_Sheet_1.ZIP › Analysis_EMG_Amplitude_Changes/EMGAmp_OutputData/Subject2/Subject2_Traj3_Point3_EMGepochs.png]

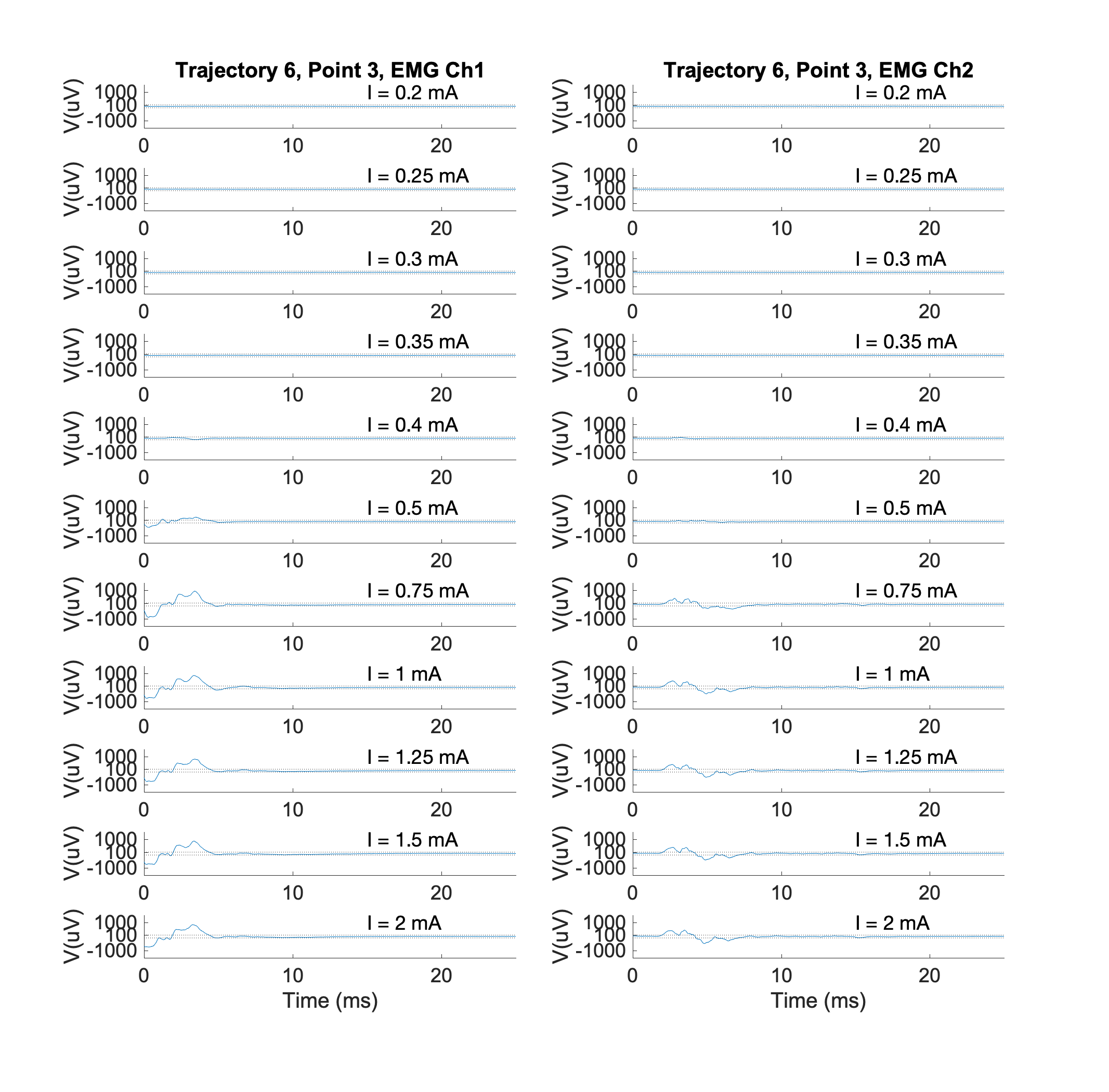

Supplement: Supplementary Data Sheet 1 — Overview of recorded electromyography data showing CMAP responses to the stimulation intensity ramp at each measurement point for the monopolar stimulation. A graph with maximum CMAP responses of monopolar stimulation for each trajectory is depicted. A Summary report (Subject 1, 2, 3.docx) of CMAP responses (for monopolar stimulation) in trajectories with potential FN damage are presented. Data sets of bipolar stimulation can be shared if the reader is interested (see Data Availability Statement). [file Data_Sheet_1.ZIP › Analysis_EMG_Amplitude_Changes/EMGAmp_OutputData/Subject2/Subject2_Traj6_Point3_EMGepochs.png]

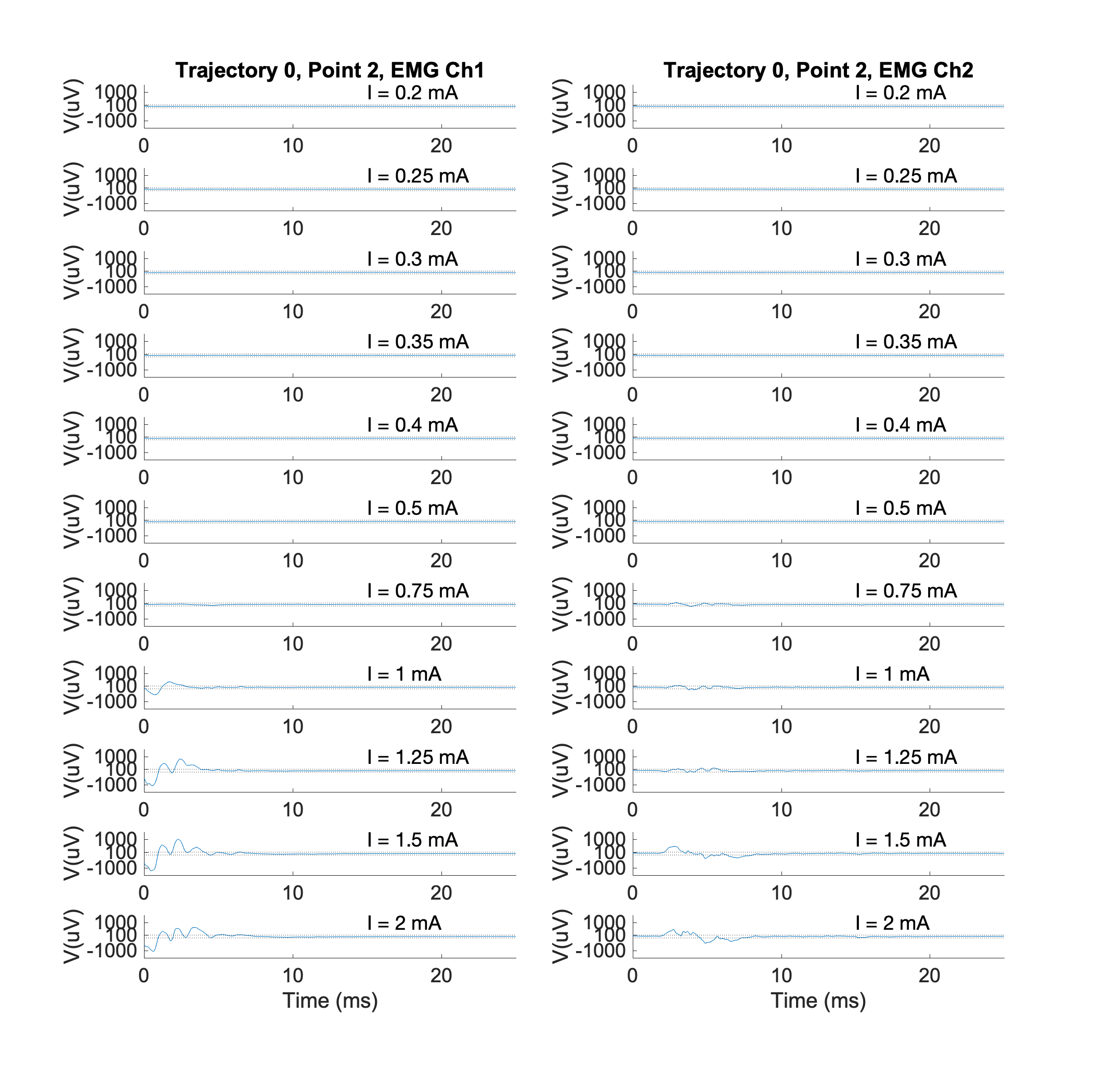

Supplement: Supplementary Data Sheet 1 — Overview of recorded electromyography data showing CMAP responses to the stimulation intensity ramp at each measurement point for the monopolar stimulation. A graph with maximum CMAP responses of monopolar stimulation for each trajectory is depicted. A Summary report (Subject 1, 2, 3.docx) of CMAP responses (for monopolar stimulation) in trajectories with potential FN damage are presented. Data sets of bipolar stimulation can be shared if the reader is interested (see Data Availability Statement). [file Data_Sheet_1.ZIP › Analysis_EMG_Amplitude_Changes/EMGAmp_OutputData/Subject2/Subject2_Traj0_Point2_EMGepochs.png]

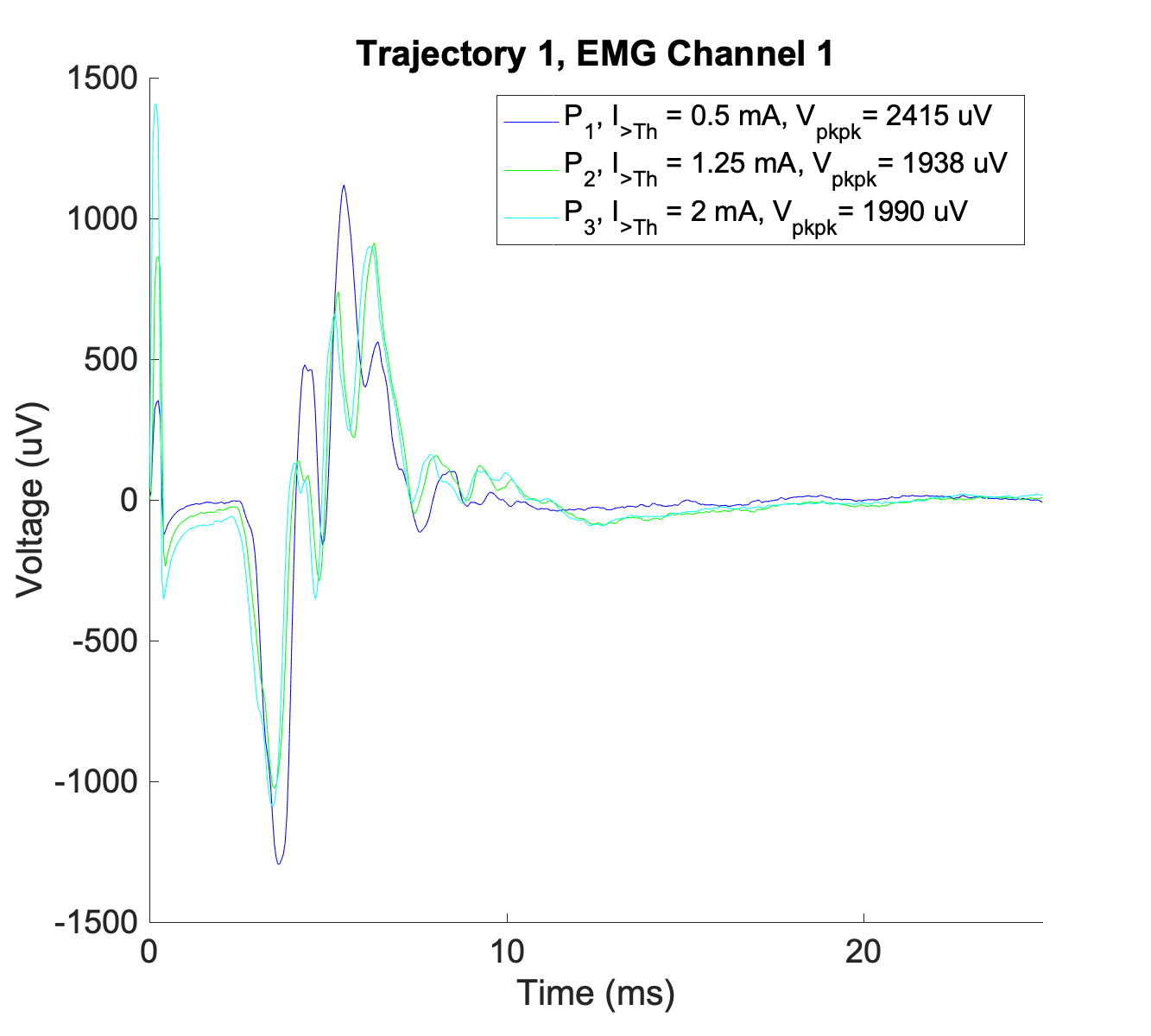

Supplement: Supplementary Data Sheet 1 — Overview of recorded electromyography data showing CMAP responses to the stimulation intensity ramp at each measurement point for the monopolar stimulation. A graph with maximum CMAP responses of monopolar stimulation for each trajectory is depicted. A Summary report (Subject 1, 2, 3.docx) of CMAP responses (for monopolar stimulation) in trajectories with potential FN damage are presented. Data sets of bipolar stimulation can be shared if the reader is interested (see Data Availability Statement). [file Data_Sheet_1.ZIP › Analysis_EMG_Amplitude_Changes/EMGAmp_OutputData/Subject2/Subject2_Traj1_AllPoints_EMG_CH1.png]

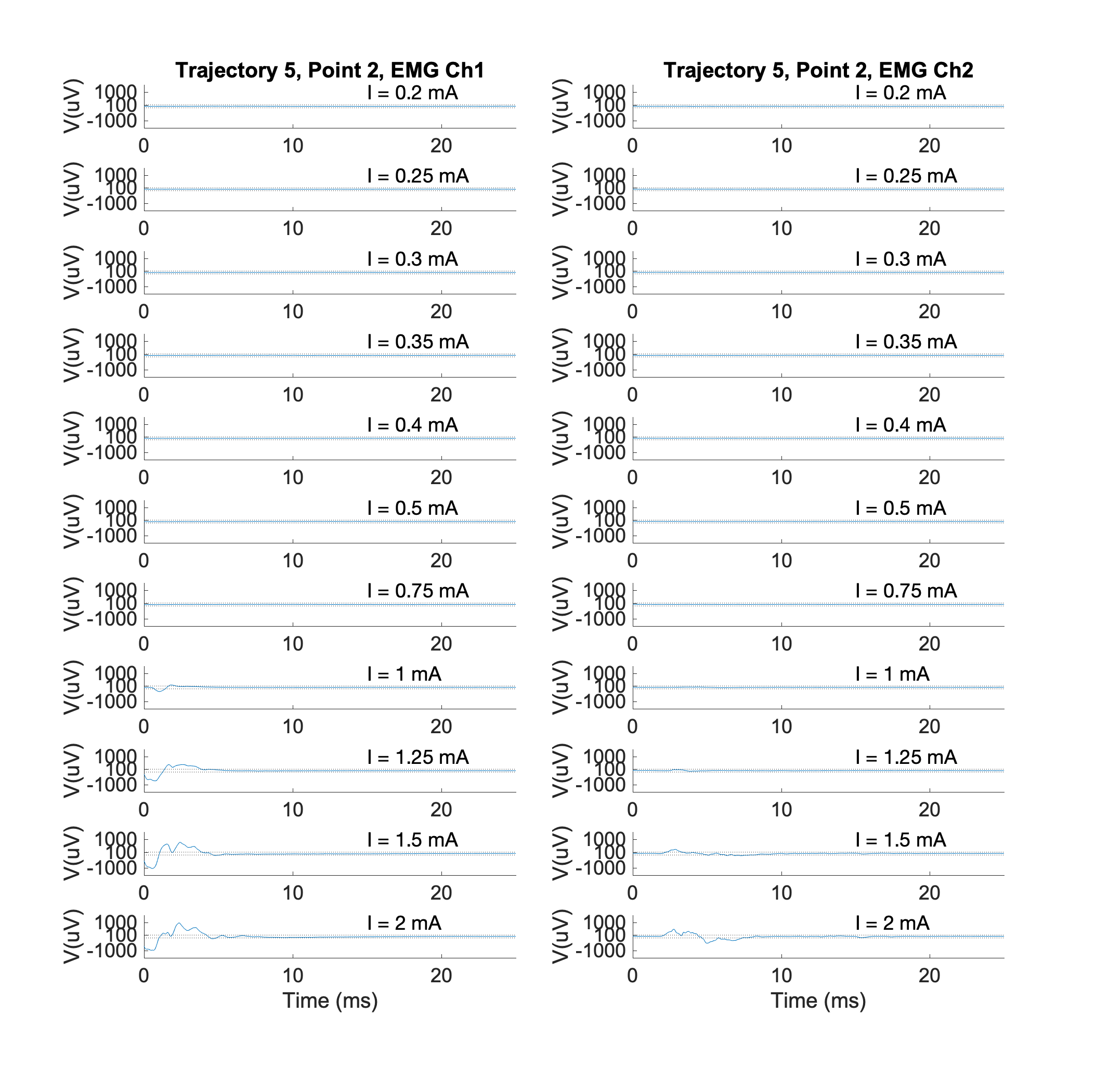

Supplement: Supplementary Data Sheet 1 — Overview of recorded electromyography data showing CMAP responses to the stimulation intensity ramp at each measurement point for the monopolar stimulation. A graph with maximum CMAP responses of monopolar stimulation for each trajectory is depicted. A Summary report (Subject 1, 2, 3.docx) of CMAP responses (for monopolar stimulation) in trajectories with potential FN damage are presented. Data sets of bipolar stimulation can be shared if the reader is interested (see Data Availability Statement). [file Data_Sheet_1.ZIP › Analysis_EMG_Amplitude_Changes/EMGAmp_OutputData/Subject2/Subject2_Traj5_Point2_EMGepochs.png]

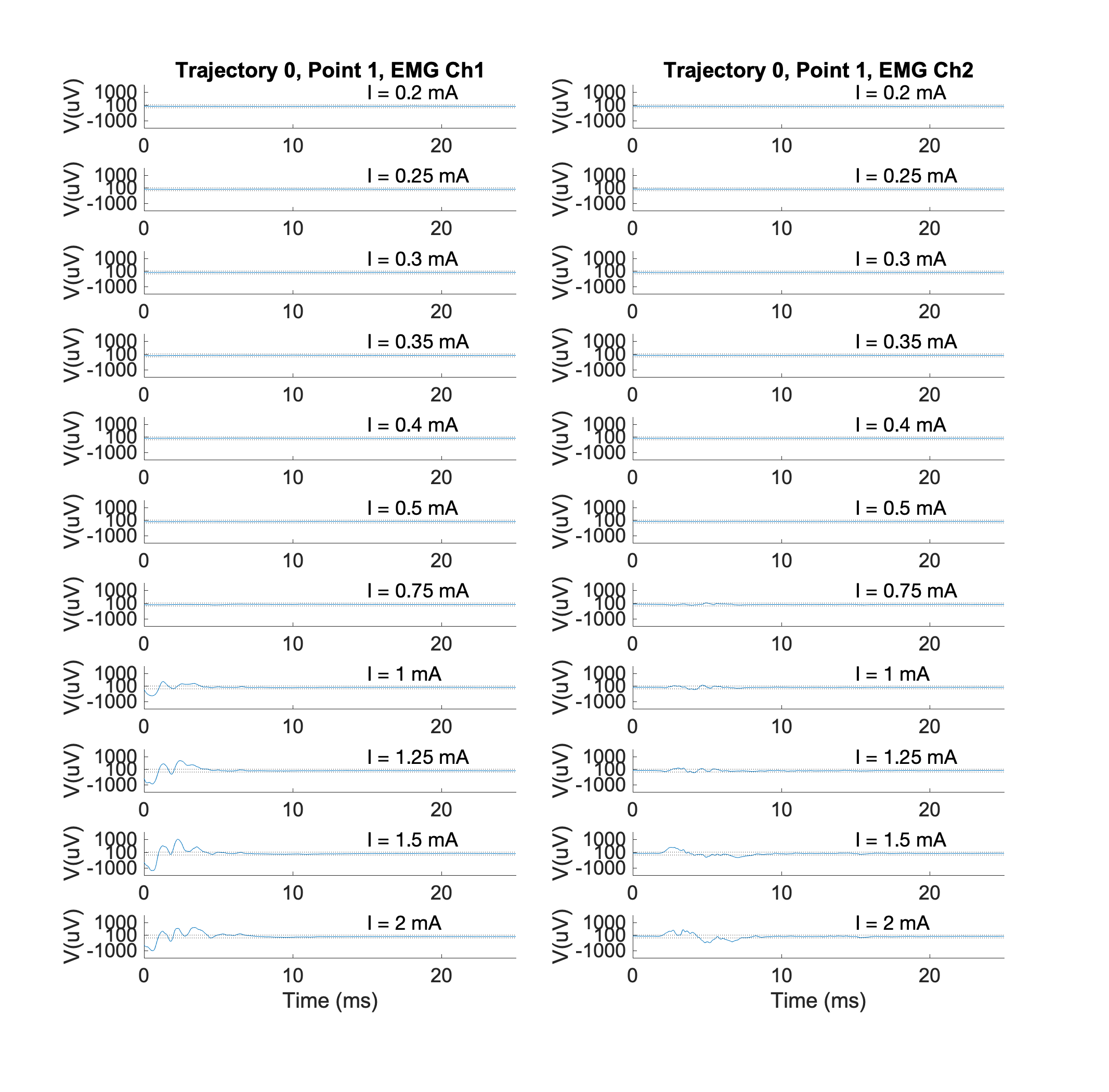

Supplement: Supplementary Data Sheet 1 — Overview of recorded electromyography data showing CMAP responses to the stimulation intensity ramp at each measurement point for the monopolar stimulation. A graph with maximum CMAP responses of monopolar stimulation for each trajectory is depicted. A Summary report (Subject 1, 2, 3.docx) of CMAP responses (for monopolar stimulation) in trajectories with potential FN damage are presented. Data sets of bipolar stimulation can be shared if the reader is interested (see Data Availability Statement). [file Data_Sheet_1.ZIP › Analysis_EMG_Amplitude_Changes/EMGAmp_OutputData/Subject2/Subject2_Traj0_Point1_EMGepochs.png]

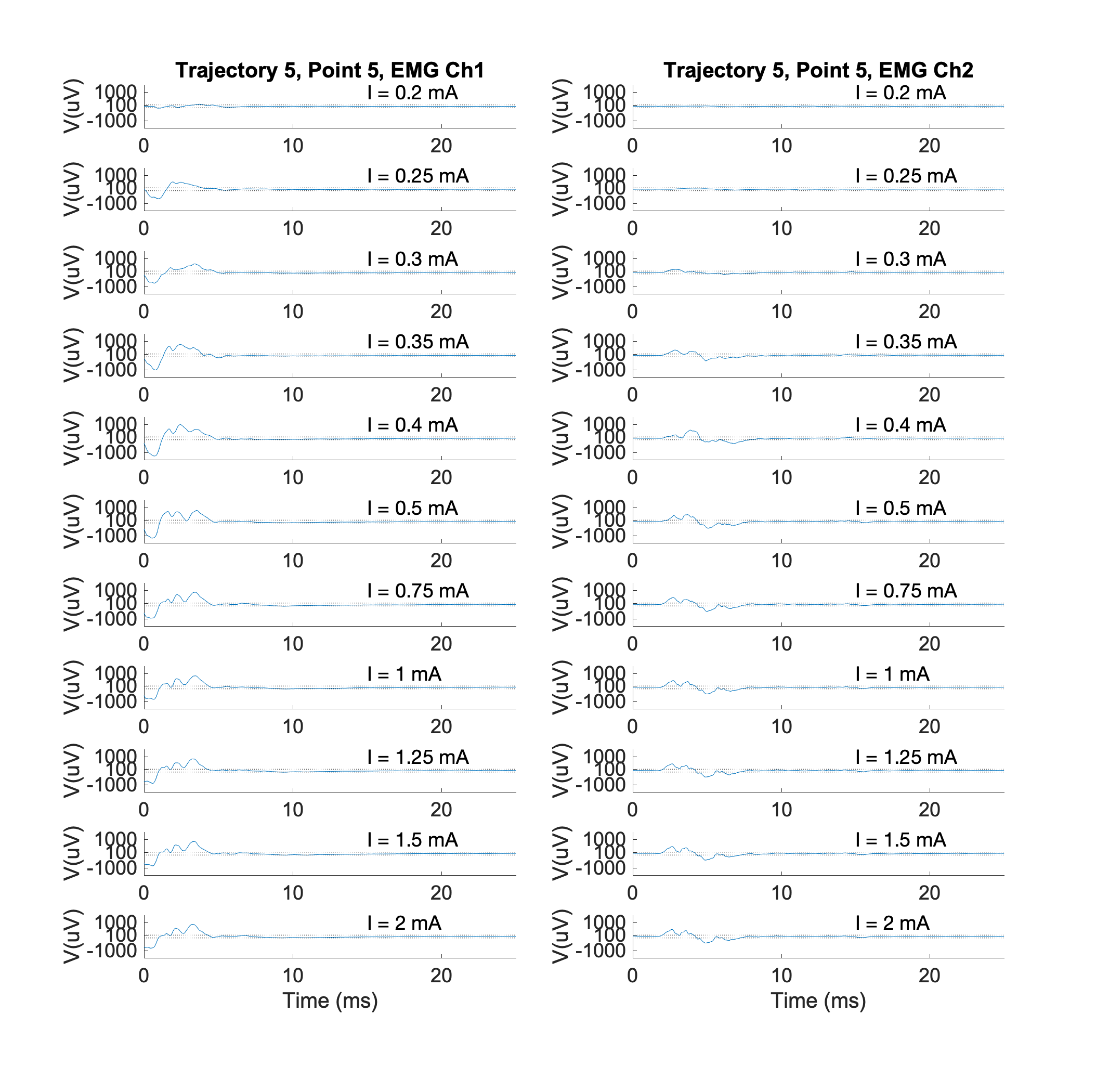

Supplement: Supplementary Data Sheet 1 — Overview of recorded electromyography data showing CMAP responses to the stimulation intensity ramp at each measurement point for the monopolar stimulation. A graph with maximum CMAP responses of monopolar stimulation for each trajectory is depicted. A Summary report (Subject 1, 2, 3.docx) of CMAP responses (for monopolar stimulation) in trajectories with potential FN damage are presented. Data sets of bipolar stimulation can be shared if the reader is interested (see Data Availability Statement). [file Data_Sheet_1.ZIP › Analysis_EMG_Amplitude_Changes/EMGAmp_OutputData/Subject2/Subject2_Traj5_Point5_EMGepochs.png]

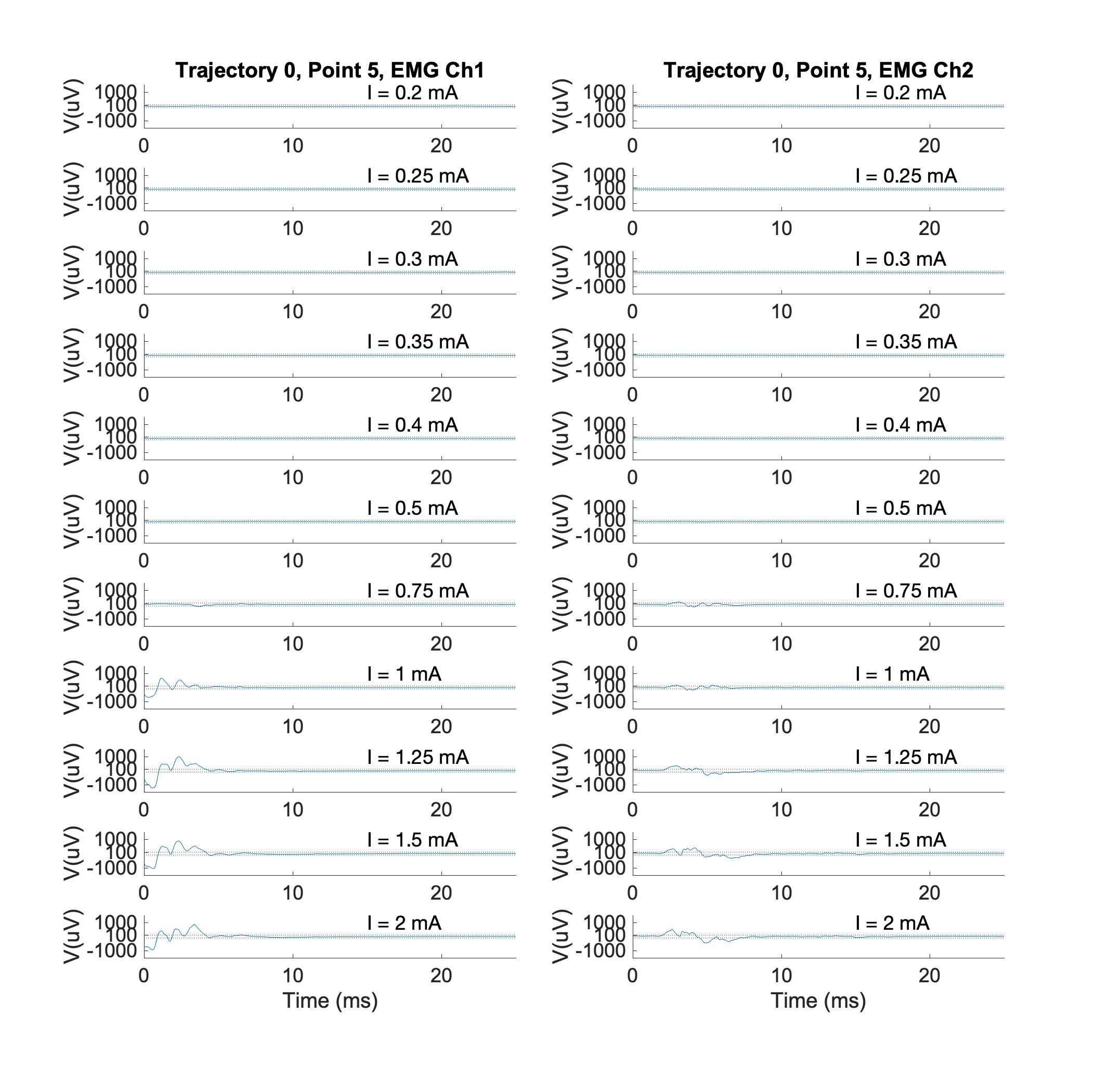

Supplement: Supplementary Data Sheet 1 — Overview of recorded electromyography data showing CMAP responses to the stimulation intensity ramp at each measurement point for the monopolar stimulation. A graph with maximum CMAP responses of monopolar stimulation for each trajectory is depicted. A Summary report (Subject 1, 2, 3.docx) of CMAP responses (for monopolar stimulation) in trajectories with potential FN damage are presented. Data sets of bipolar stimulation can be shared if the reader is interested (see Data Availability Statement). [file Data_Sheet_1.ZIP › Analysis_EMG_Amplitude_Changes/EMGAmp_OutputData/Subject2/Subject2_Traj0_Point5_EMGepochs.png]

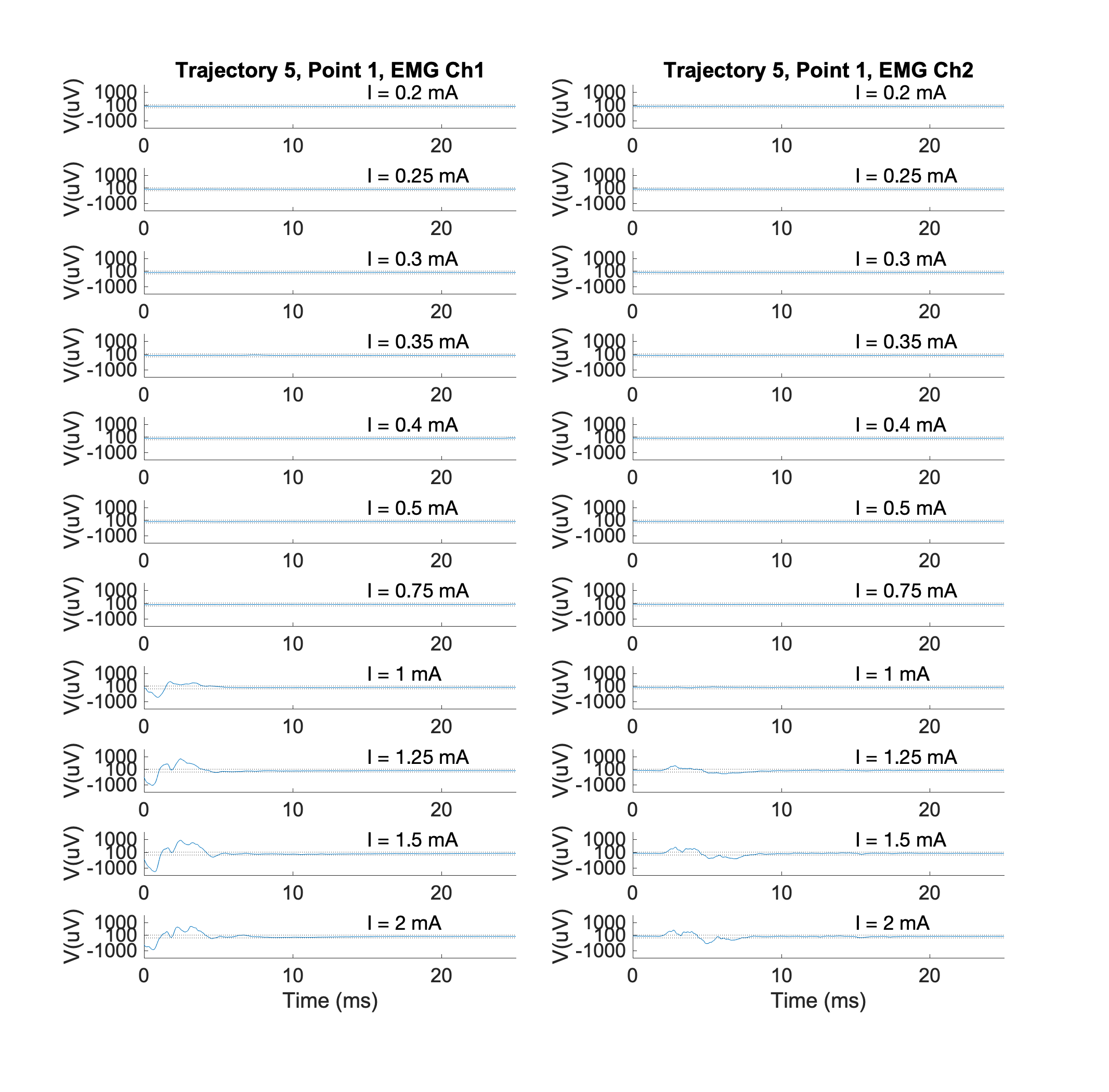

Supplement: Supplementary Data Sheet 1 — Overview of recorded electromyography data showing CMAP responses to the stimulation intensity ramp at each measurement point for the monopolar stimulation. A graph with maximum CMAP responses of monopolar stimulation for each trajectory is depicted. A Summary report (Subject 1, 2, 3.docx) of CMAP responses (for monopolar stimulation) in trajectories with potential FN damage are presented. Data sets of bipolar stimulation can be shared if the reader is interested (see Data Availability Statement). [file Data_Sheet_1.ZIP › Analysis_EMG_Amplitude_Changes/EMGAmp_OutputData/Subject2/Subject2_Traj5_Point1_EMGepochs.png]

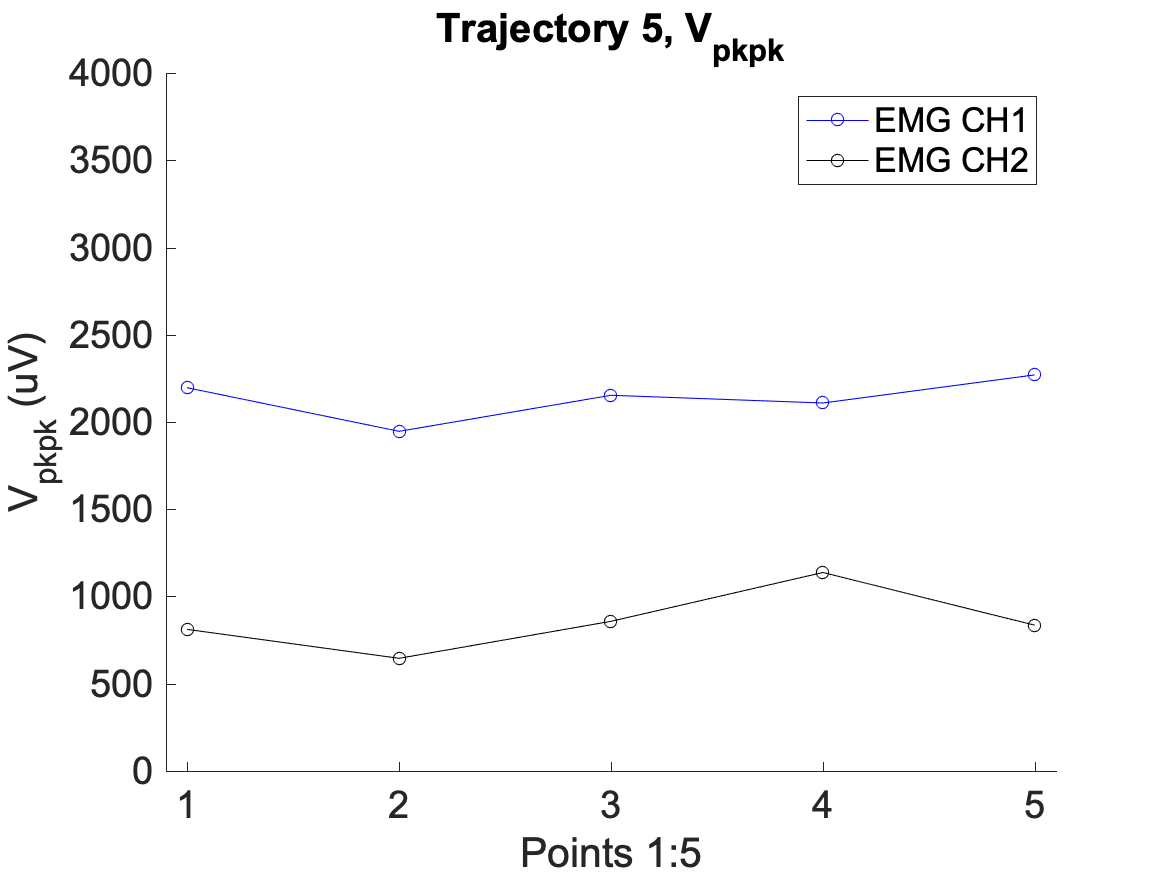

Supplement: Supplementary Data Sheet 1 — Overview of recorded electromyography data showing CMAP responses to the stimulation intensity ramp at each measurement point for the monopolar stimulation. A graph with maximum CMAP responses of monopolar stimulation for each trajectory is depicted. A Summary report (Subject 1, 2, 3.docx) of CMAP responses (for monopolar stimulation) in trajectories with potential FN damage are presented. Data sets of bipolar stimulation can be shared if the reader is interested (see Data Availability Statement). [file Data_Sheet_1.ZIP › Analysis_EMG_Amplitude_Changes/EMGAmp_OutputData/Subject2/Subject2_Traj5_Vpkk.png]

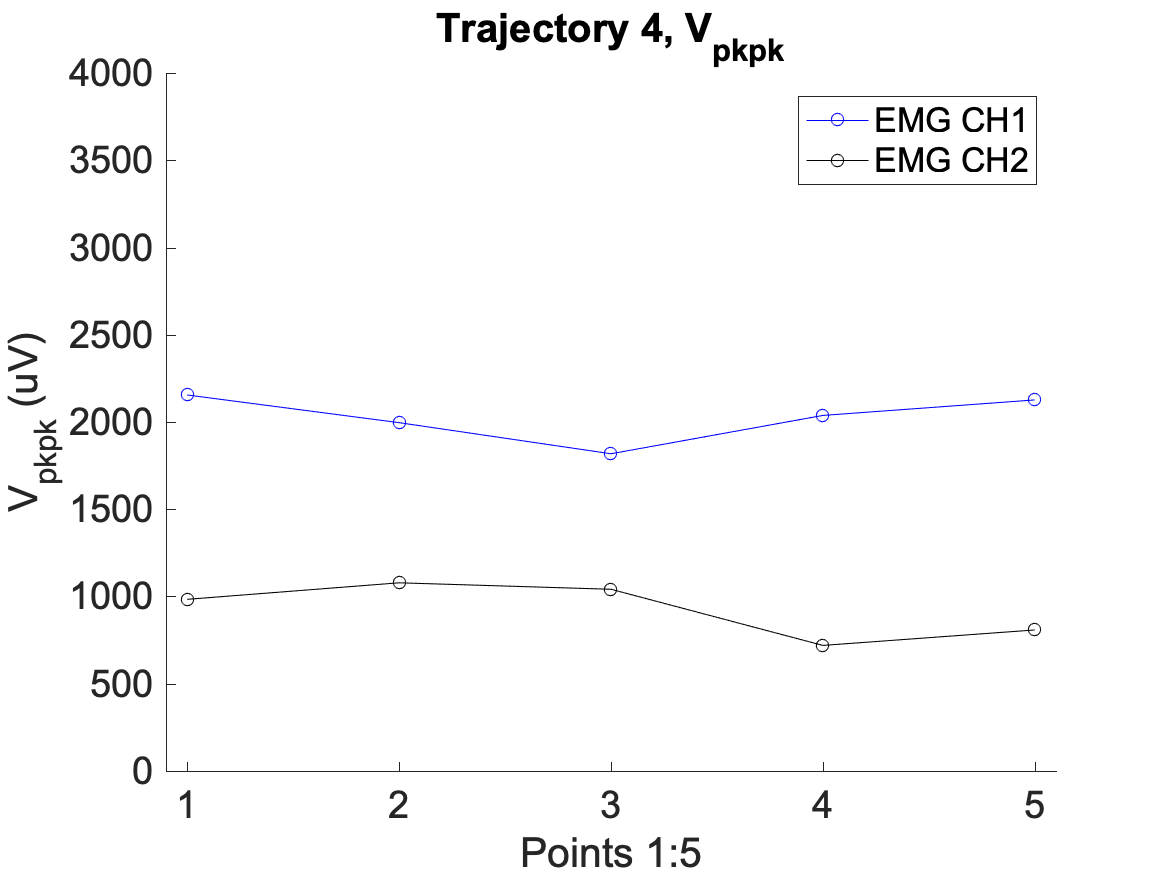

Supplement: Supplementary Data Sheet 1 — Overview of recorded electromyography data showing CMAP responses to the stimulation intensity ramp at each measurement point for the monopolar stimulation. A graph with maximum CMAP responses of monopolar stimulation for each trajectory is depicted. A Summary report (Subject 1, 2, 3.docx) of CMAP responses (for monopolar stimulation) in trajectories with potential FN damage are presented. Data sets of bipolar stimulation can be shared if the reader is interested (see Data Availability Statement). [file Data_Sheet_1.ZIP › Analysis_EMG_Amplitude_Changes/EMGAmp_OutputData/Subject2/Subject2_Traj4_Vpkk.png]

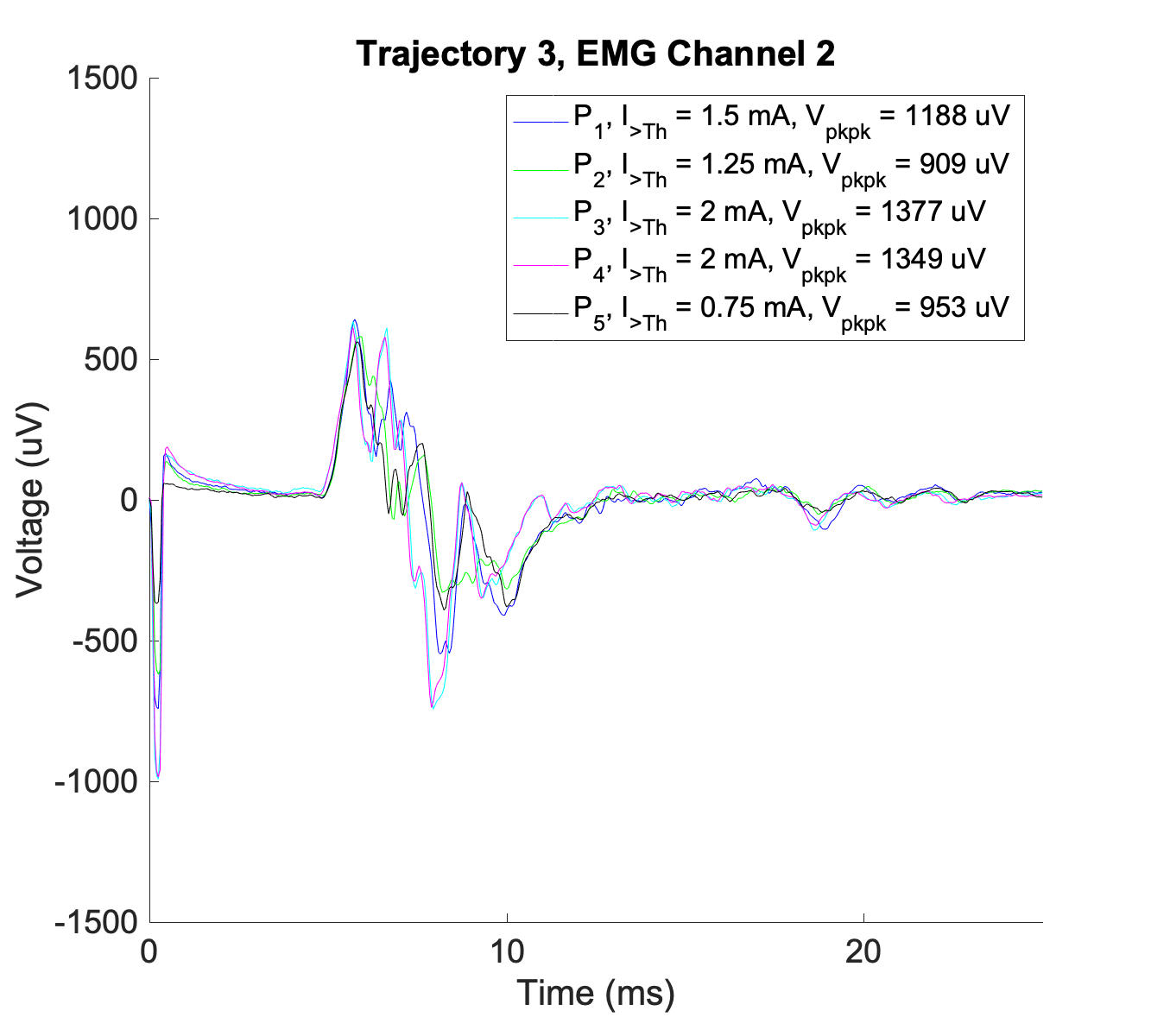

Supplement: Supplementary Data Sheet 1 — Overview of recorded electromyography data showing CMAP responses to the stimulation intensity ramp at each measurement point for the monopolar stimulation. A graph with maximum CMAP responses of monopolar stimulation for each trajectory is depicted. A Summary report (Subject 1, 2, 3.docx) of CMAP responses (for monopolar stimulation) in trajectories with potential FN damage are presented. Data sets of bipolar stimulation can be shared if the reader is interested (see Data Availability Statement). [file Data_Sheet_1.ZIP › Analysis_EMG_Amplitude_Changes/EMGAmp_OutputData/Subject2/Subject2_Traj3_AllPoints_EMG_CH2.png]

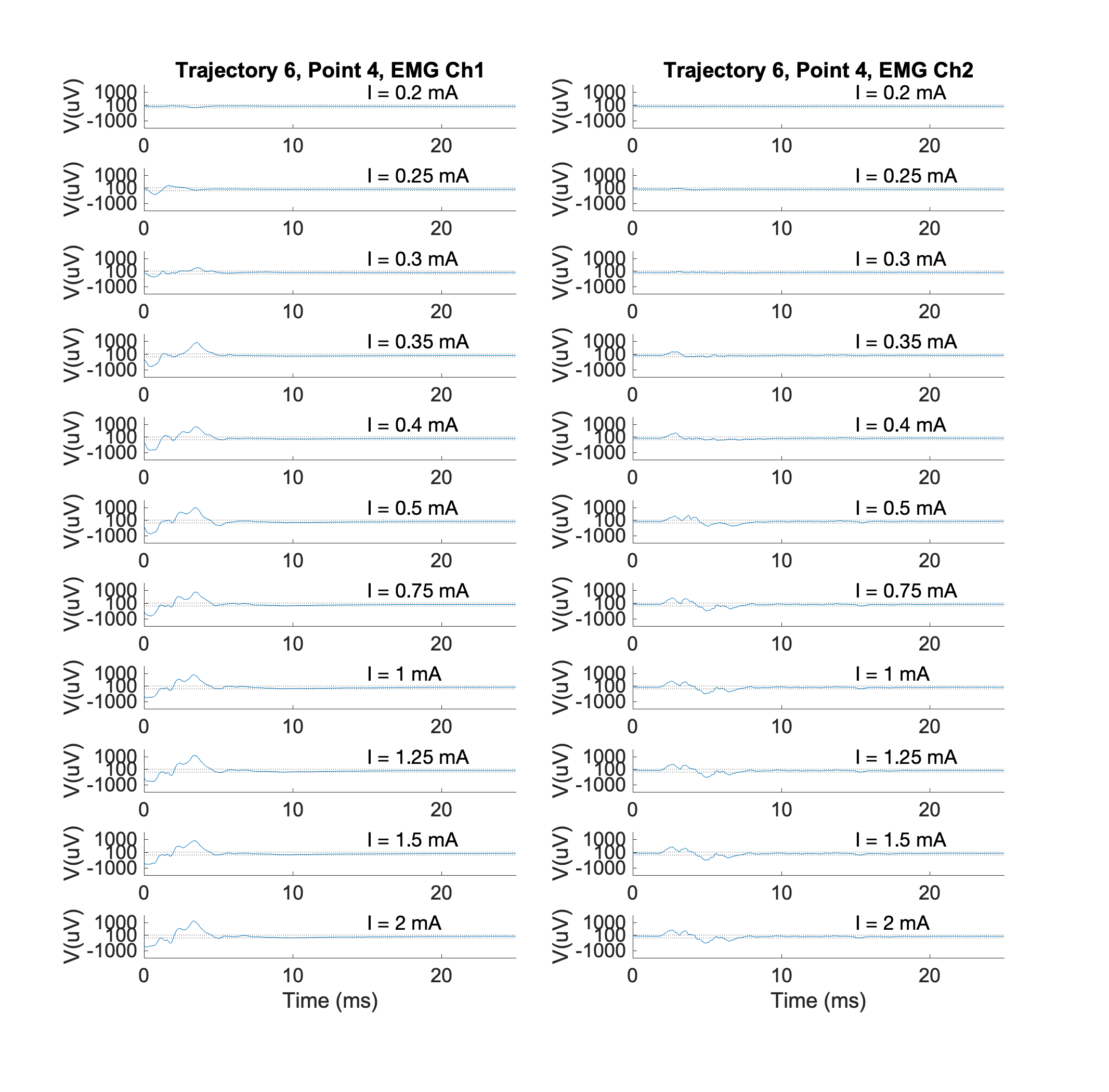

Supplement: Supplementary Data Sheet 1 — Overview of recorded electromyography data showing CMAP responses to the stimulation intensity ramp at each measurement point for the monopolar stimulation. A graph with maximum CMAP responses of monopolar stimulation for each trajectory is depicted. A Summary report (Subject 1, 2, 3.docx) of CMAP responses (for monopolar stimulation) in trajectories with potential FN damage are presented. Data sets of bipolar stimulation can be shared if the reader is interested (see Data Availability Statement). [file Data_Sheet_1.ZIP › Analysis_EMG_Amplitude_Changes/EMGAmp_OutputData/Subject2/Subject2_Traj6_Point4_EMGepochs.png]

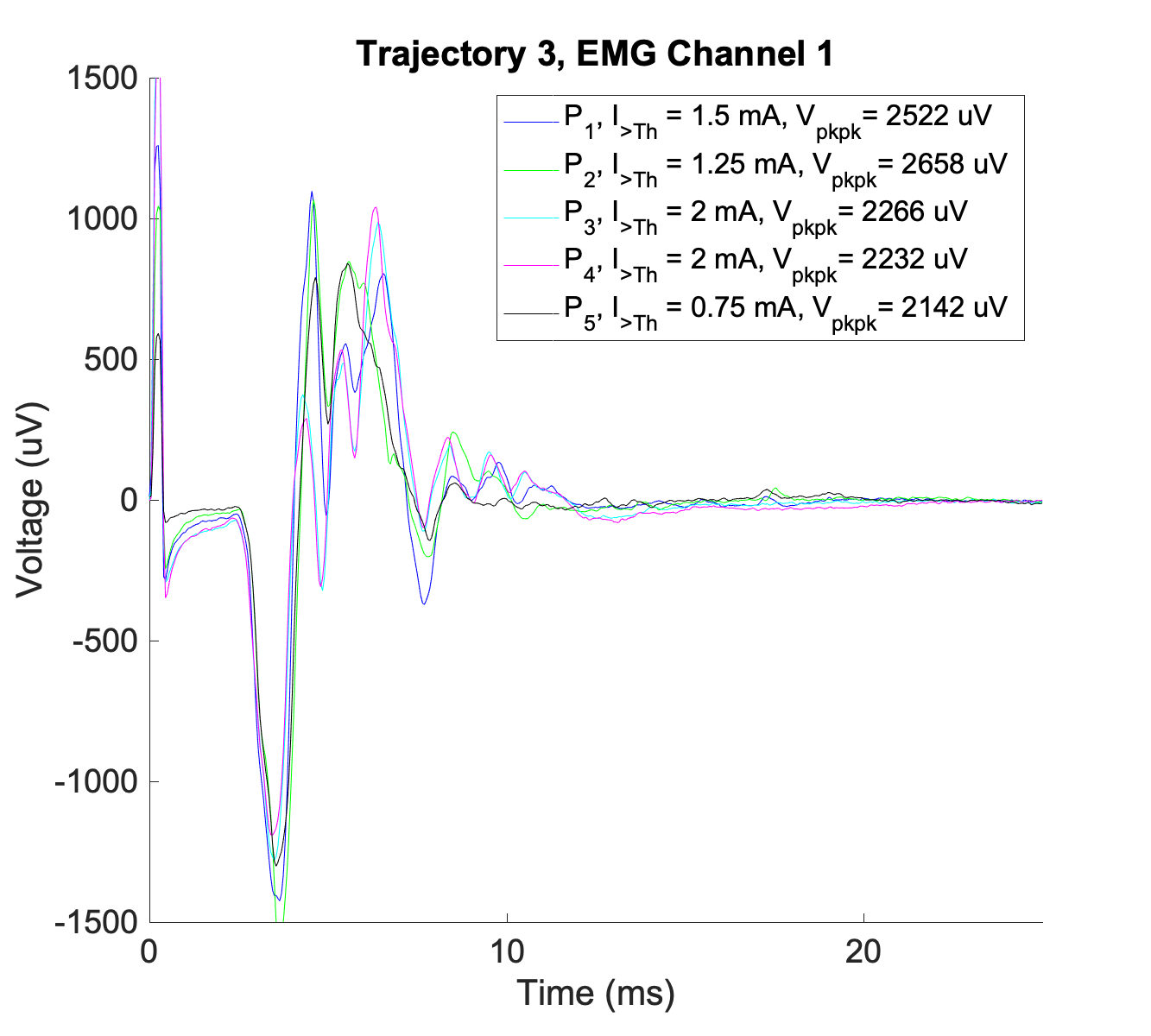

Supplement: Supplementary Data Sheet 1 — Overview of recorded electromyography data showing CMAP responses to the stimulation intensity ramp at each measurement point for the monopolar stimulation. A graph with maximum CMAP responses of monopolar stimulation for each trajectory is depicted. A Summary report (Subject 1, 2, 3.docx) of CMAP responses (for monopolar stimulation) in trajectories with potential FN damage are presented. Data sets of bipolar stimulation can be shared if the reader is interested (see Data Availability Statement). [file Data_Sheet_1.ZIP › Analysis_EMG_Amplitude_Changes/EMGAmp_OutputData/Subject2/Subject2_Traj3_AllPoints_EMG_CH1.png]

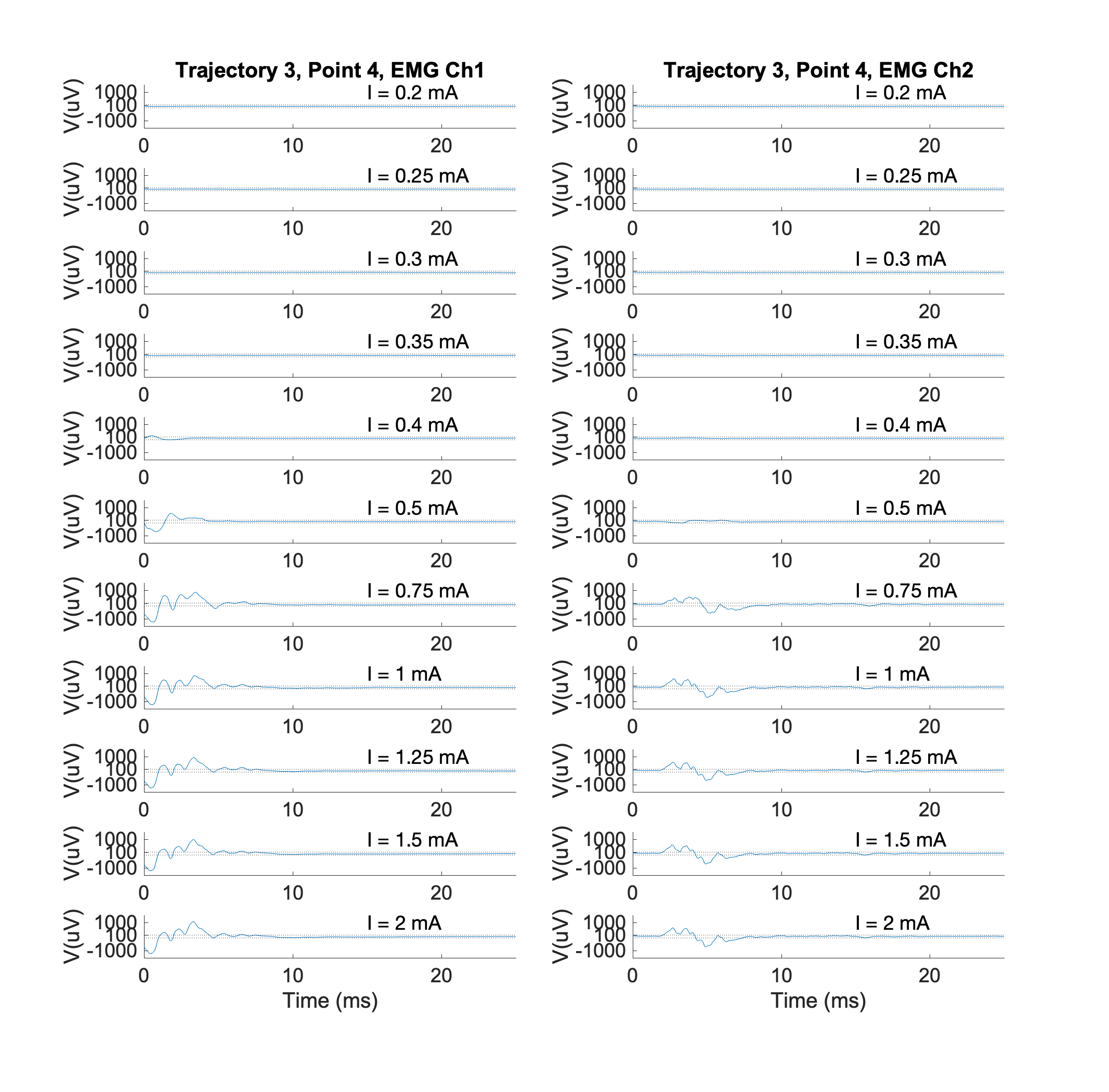

Supplement: Supplementary Data Sheet 1 — Overview of recorded electromyography data showing CMAP responses to the stimulation intensity ramp at each measurement point for the monopolar stimulation. A graph with maximum CMAP responses of monopolar stimulation for each trajectory is depicted. A Summary report (Subject 1, 2, 3.docx) of CMAP responses (for monopolar stimulation) in trajectories with potential FN damage are presented. Data sets of bipolar stimulation can be shared if the reader is interested (see Data Availability Statement). [file Data_Sheet_1.ZIP › Analysis_EMG_Amplitude_Changes/EMGAmp_OutputData/Subject2/Subject2_Traj3_Point4_EMGepochs.png]

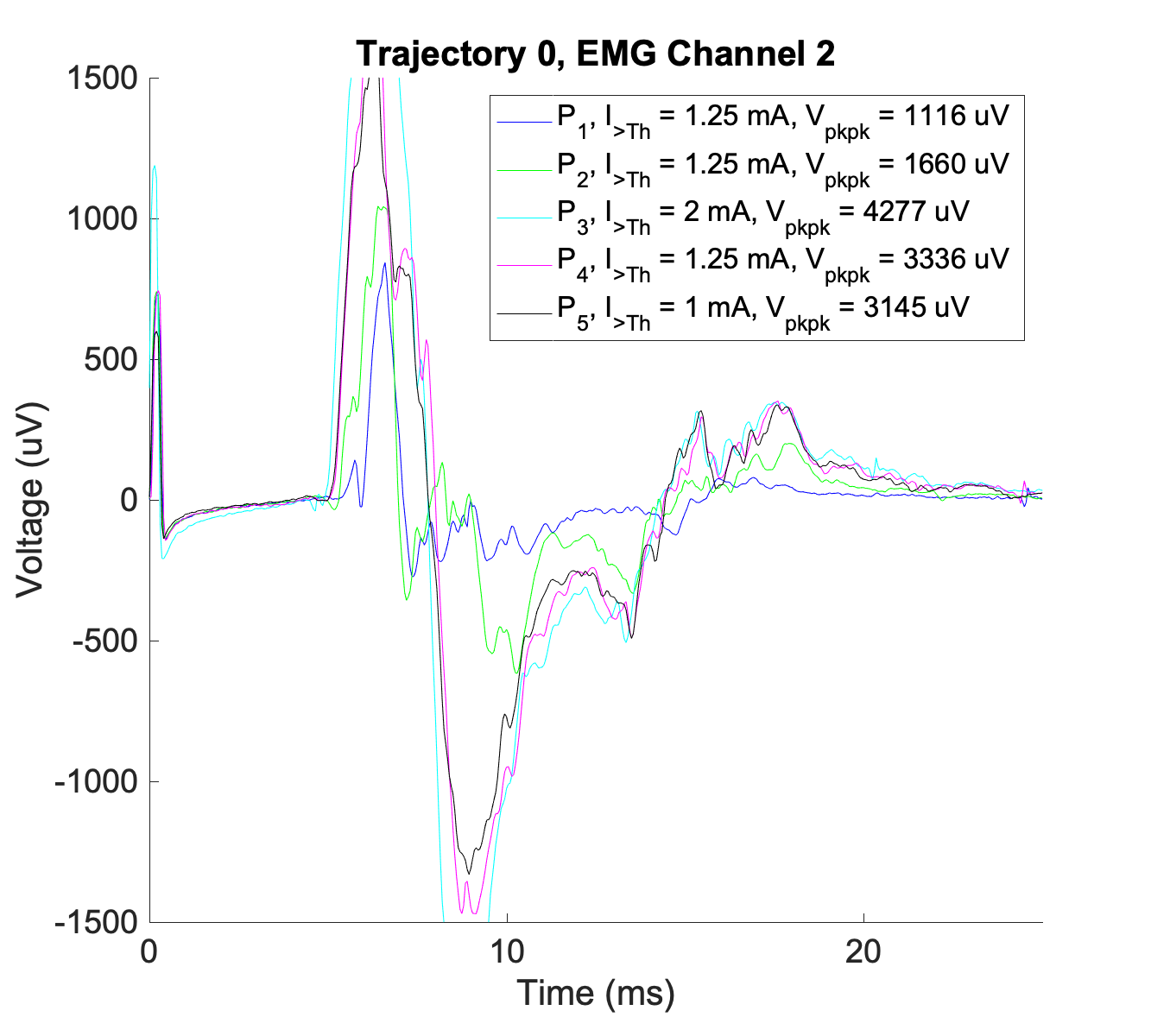

Supplement: Supplementary Data Sheet 1 — Overview of recorded electromyography data showing CMAP responses to the stimulation intensity ramp at each measurement point for the monopolar stimulation. A graph with maximum CMAP responses of monopolar stimulation for each trajectory is depicted. A Summary report (Subject 1, 2, 3.docx) of CMAP responses (for monopolar stimulation) in trajectories with potential FN damage are presented. Data sets of bipolar stimulation can be shared if the reader is interested (see Data Availability Statement). [file Data_Sheet_1.ZIP › Analysis_EMG_Amplitude_Changes/EMGAmp_OutputData/Subject3/Subject3_Traj0_AllPoints_EMG_CH2.png]

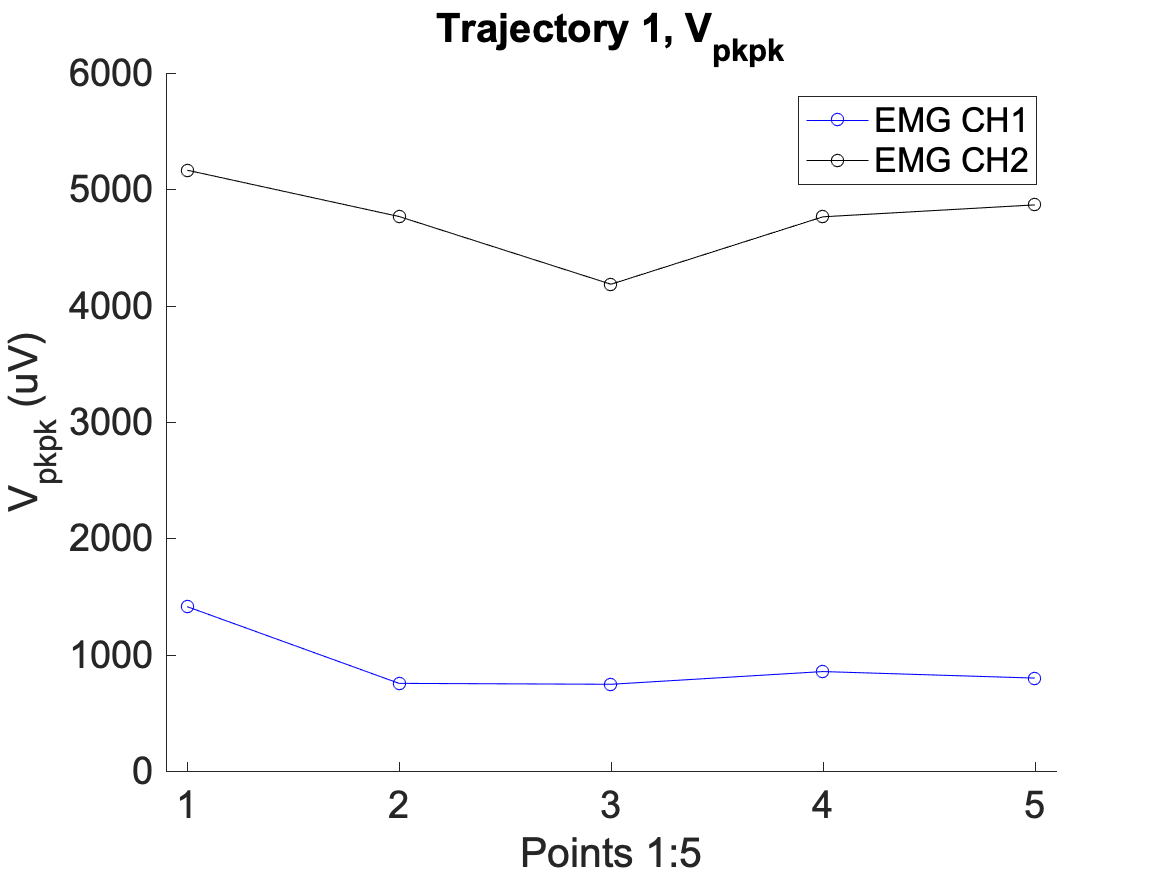

Supplement: Supplementary Data Sheet 1 — Overview of recorded electromyography data showing CMAP responses to the stimulation intensity ramp at each measurement point for the monopolar stimulation. A graph with maximum CMAP responses of monopolar stimulation for each trajectory is depicted. A Summary report (Subject 1, 2, 3.docx) of CMAP responses (for monopolar stimulation) in trajectories with potential FN damage are presented. Data sets of bipolar stimulation can be shared if the reader is interested (see Data Availability Statement). [file Data_Sheet_1.ZIP › Analysis_EMG_Amplitude_Changes/EMGAmp_OutputData/Subject3/Subject3_Traj1_Vpkk.png]

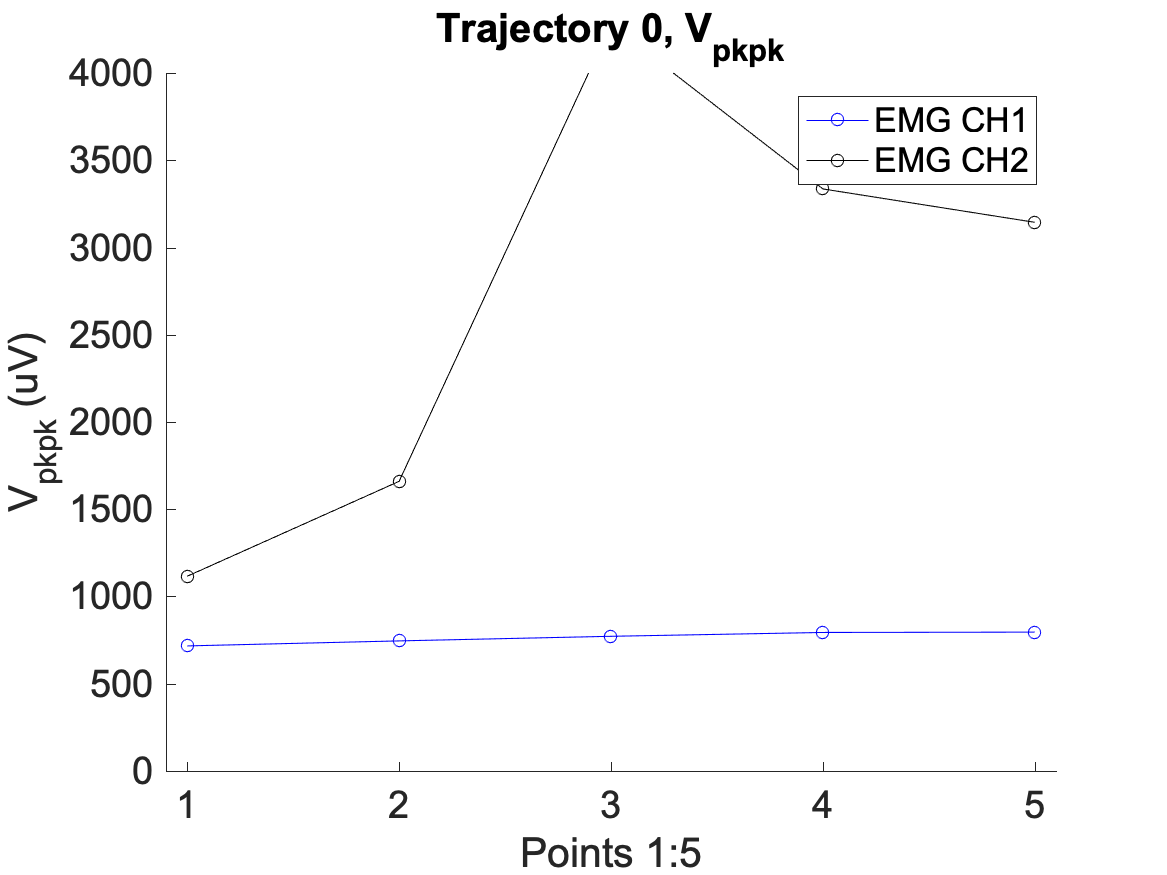

Supplement: Supplementary Data Sheet 1 — Overview of recorded electromyography data showing CMAP responses to the stimulation intensity ramp at each measurement point for the monopolar stimulation. A graph with maximum CMAP responses of monopolar stimulation for each trajectory is depicted. A Summary report (Subject 1, 2, 3.docx) of CMAP responses (for monopolar stimulation) in trajectories with potential FN damage are presented. Data sets of bipolar stimulation can be shared if the reader is interested (see Data Availability Statement). [file Data_Sheet_1.ZIP › Analysis_EMG_Amplitude_Changes/EMGAmp_OutputData/Subject3/Subject3_Traj0_Vpkk.png]

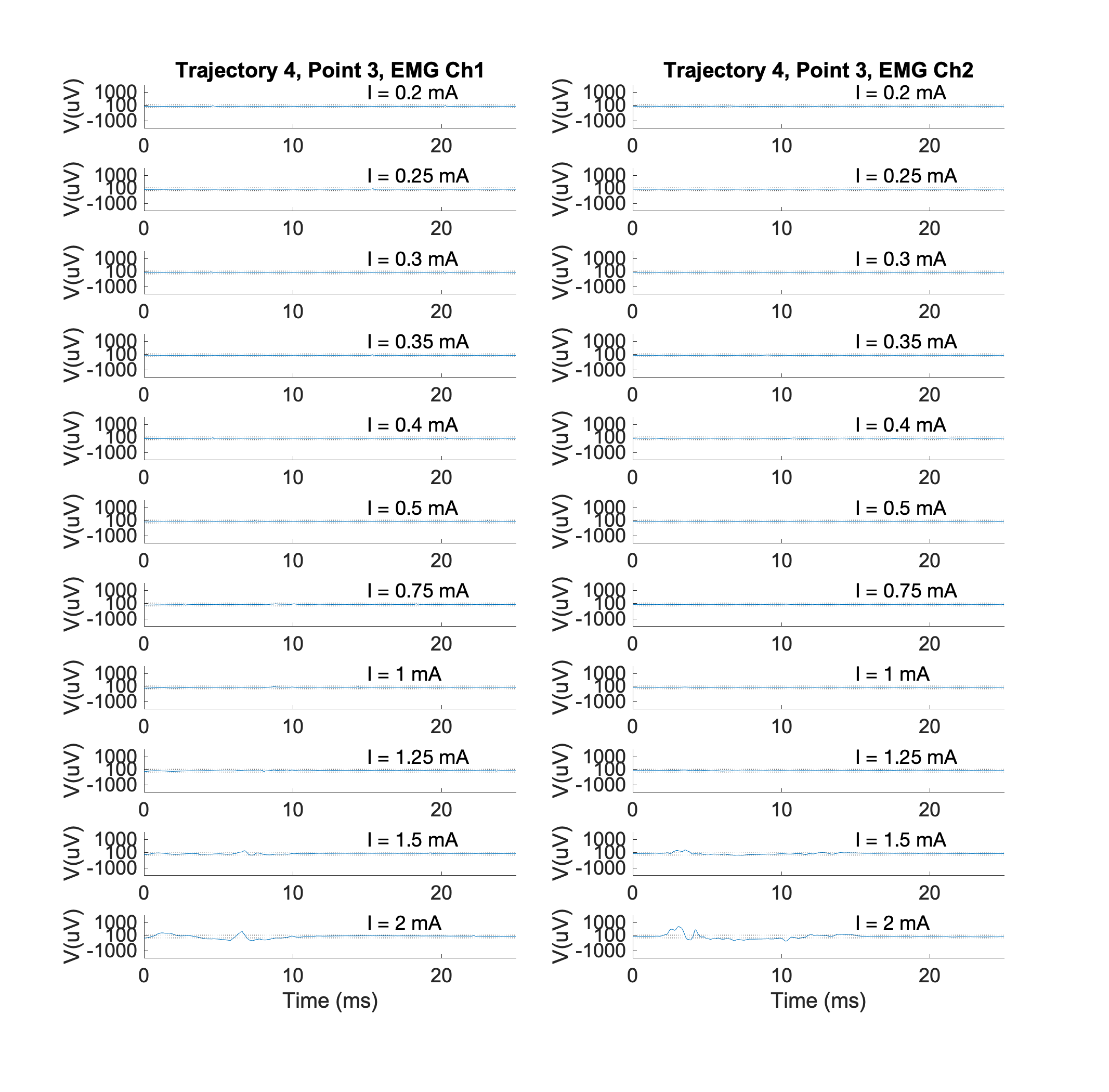

Supplement: Supplementary Data Sheet 1 — Overview of recorded electromyography data showing CMAP responses to the stimulation intensity ramp at each measurement point for the monopolar stimulation. A graph with maximum CMAP responses of monopolar stimulation for each trajectory is depicted. A Summary report (Subject 1, 2, 3.docx) of CMAP responses (for monopolar stimulation) in trajectories with potential FN damage are presented. Data sets of bipolar stimulation can be shared if the reader is interested (see Data Availability Statement). [file Data_Sheet_1.ZIP › Analysis_EMG_Amplitude_Changes/EMGAmp_OutputData/Subject3/Subject3_Traj4_Point3_EMGepochs.png]

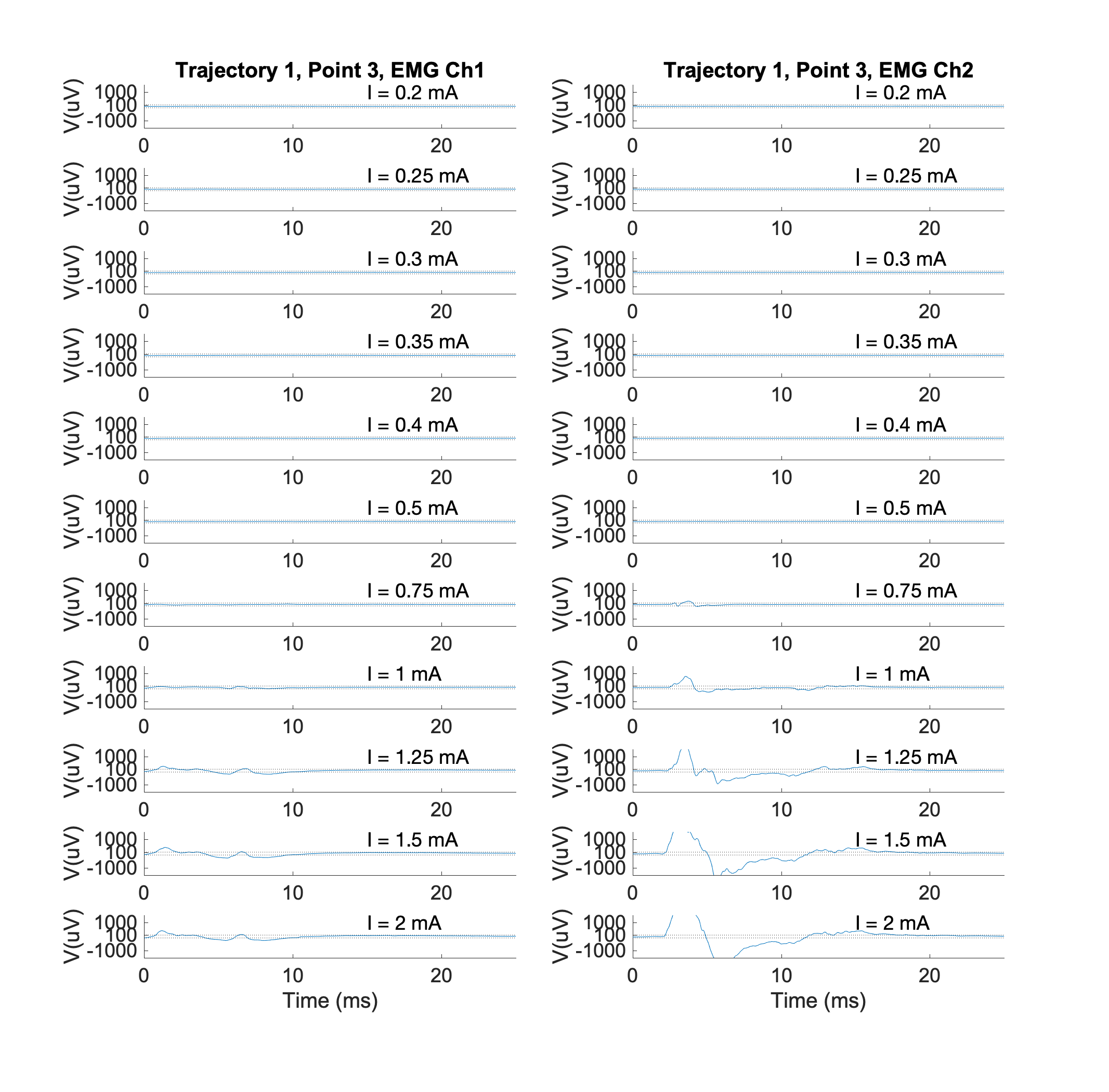

Supplement: Supplementary Data Sheet 1 — Overview of recorded electromyography data showing CMAP responses to the stimulation intensity ramp at each measurement point for the monopolar stimulation. A graph with maximum CMAP responses of monopolar stimulation for each trajectory is depicted. A Summary report (Subject 1, 2, 3.docx) of CMAP responses (for monopolar stimulation) in trajectories with potential FN damage are presented. Data sets of bipolar stimulation can be shared if the reader is interested (see Data Availability Statement). [file Data_Sheet_1.ZIP › Analysis_EMG_Amplitude_Changes/EMGAmp_OutputData/Subject3/Subject3_Traj1_Point3_EMGepochs.png]

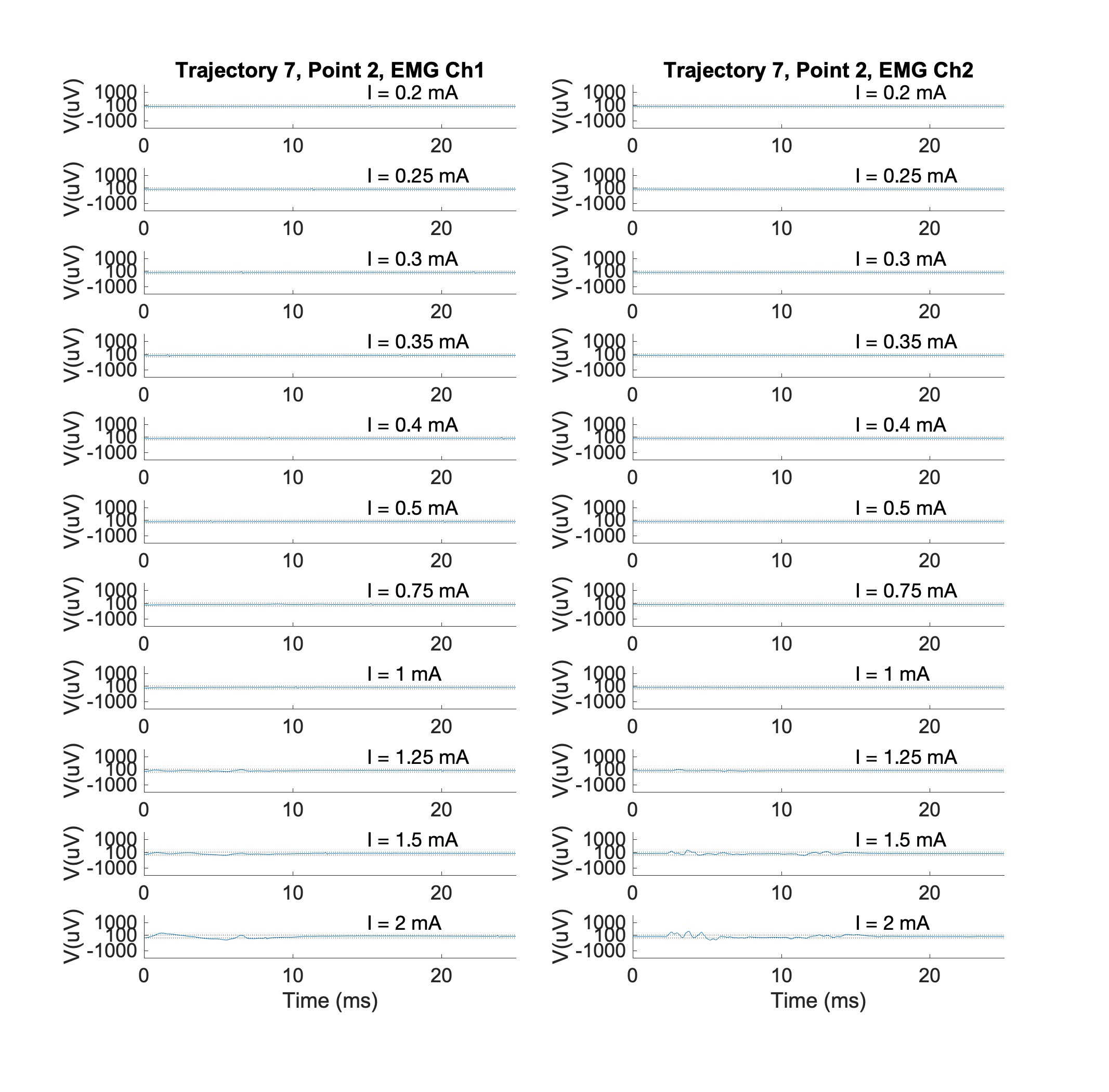

Supplement: Supplementary Data Sheet 1 — Overview of recorded electromyography data showing CMAP responses to the stimulation intensity ramp at each measurement point for the monopolar stimulation. A graph with maximum CMAP responses of monopolar stimulation for each trajectory is depicted. A Summary report (Subject 1, 2, 3.docx) of CMAP responses (for monopolar stimulation) in trajectories with potential FN damage are presented. Data sets of bipolar stimulation can be shared if the reader is interested (see Data Availability Statement). [file Data_Sheet_1.ZIP › Analysis_EMG_Amplitude_Changes/EMGAmp_OutputData/Subject3/Subject3_Traj7_Point2_EMGepochs.png]

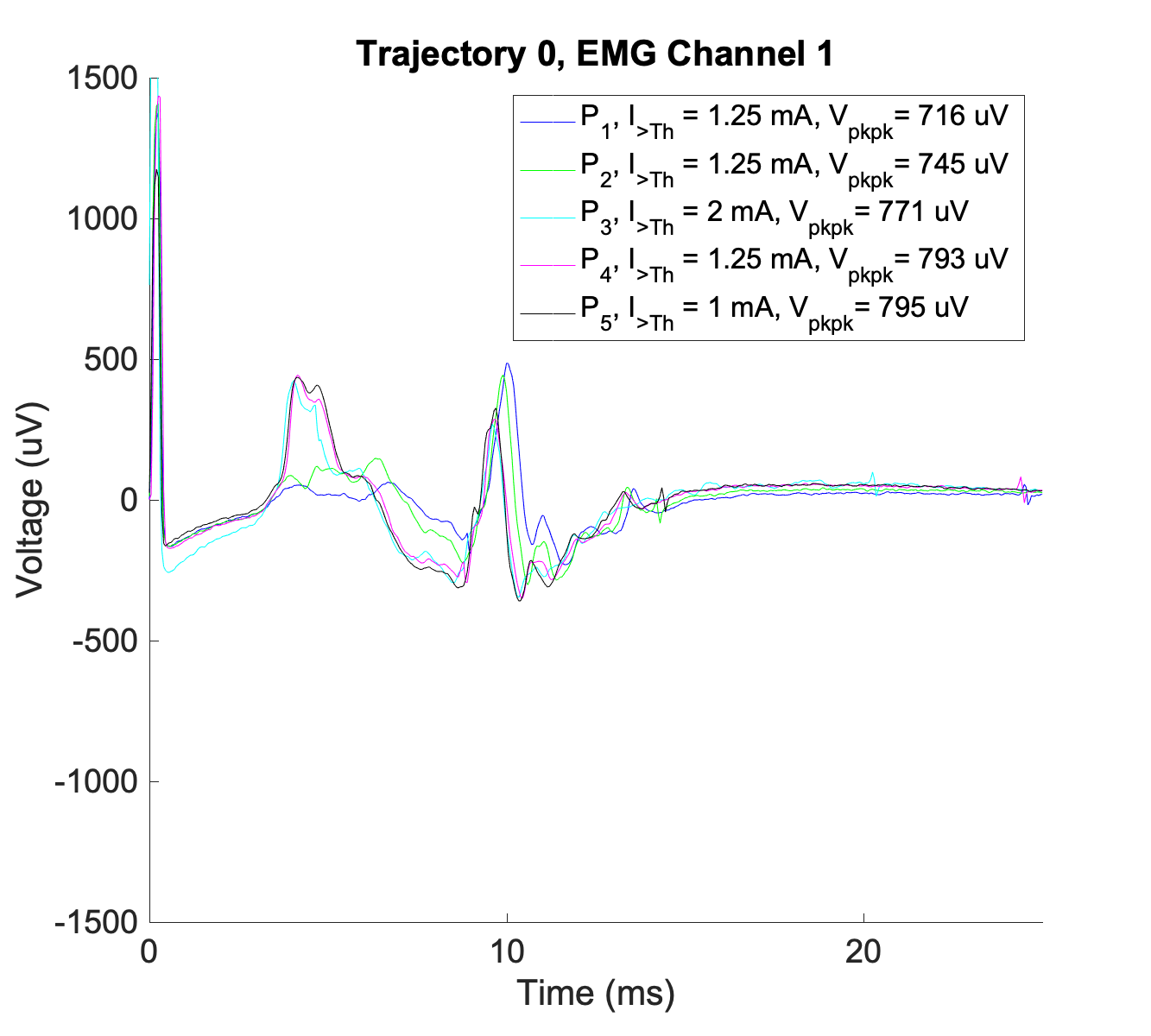

Supplement: Supplementary Data Sheet 1 — Overview of recorded electromyography data showing CMAP responses to the stimulation intensity ramp at each measurement point for the monopolar stimulation. A graph with maximum CMAP responses of monopolar stimulation for each trajectory is depicted. A Summary report (Subject 1, 2, 3.docx) of CMAP responses (for monopolar stimulation) in trajectories with potential FN damage are presented. Data sets of bipolar stimulation can be shared if the reader is interested (see Data Availability Statement). [file Data_Sheet_1.ZIP › Analysis_EMG_Amplitude_Changes/EMGAmp_OutputData/Subject3/Subject3_Traj0_AllPoints_EMG_CH1.png]

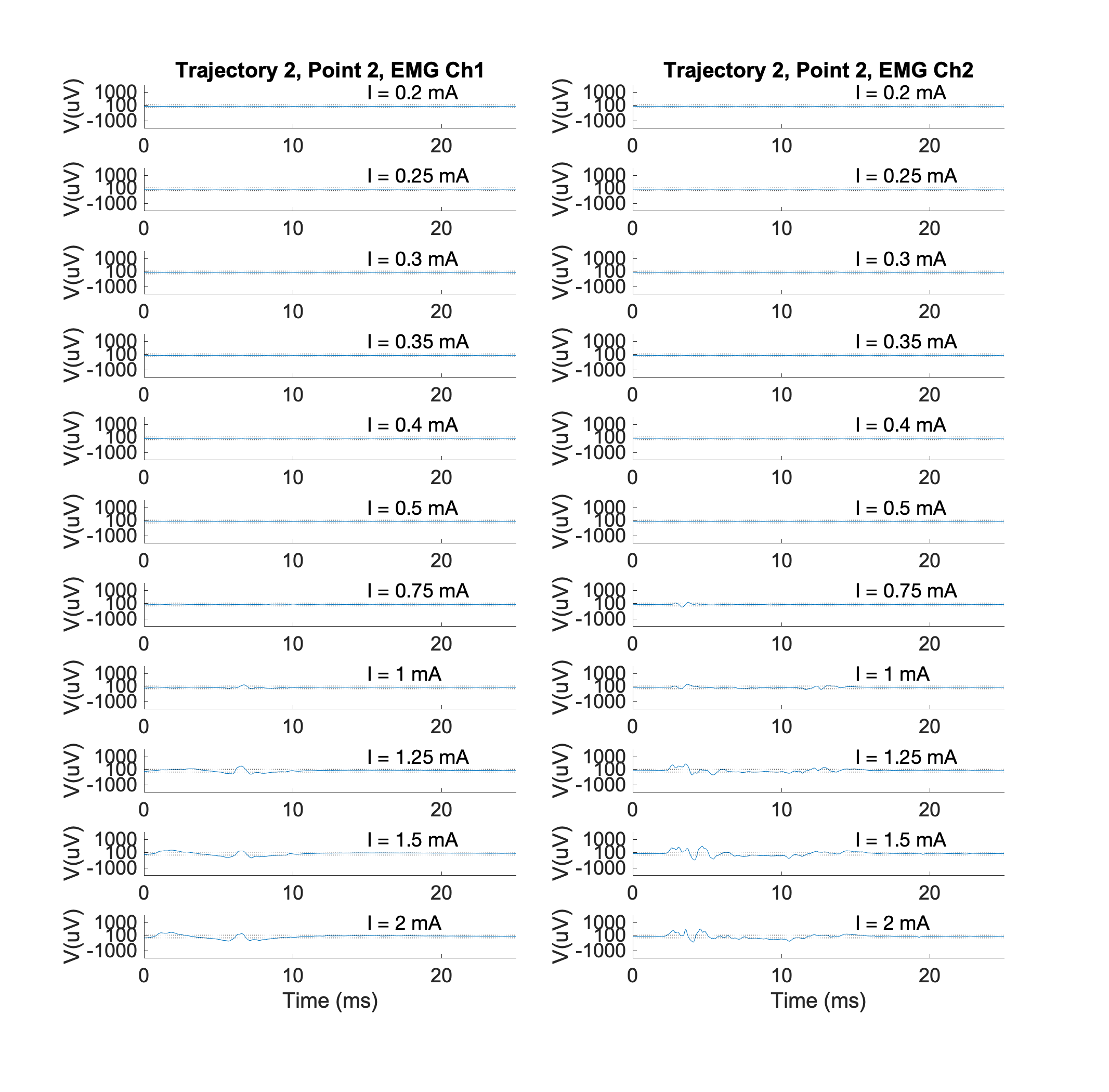

Supplement: Supplementary Data Sheet 1 — Overview of recorded electromyography data showing CMAP responses to the stimulation intensity ramp at each measurement point for the monopolar stimulation. A graph with maximum CMAP responses of monopolar stimulation for each trajectory is depicted. A Summary report (Subject 1, 2, 3.docx) of CMAP responses (for monopolar stimulation) in trajectories with potential FN damage are presented. Data sets of bipolar stimulation can be shared if the reader is interested (see Data Availability Statement). [file Data_Sheet_1.ZIP › Analysis_EMG_Amplitude_Changes/EMGAmp_OutputData/Subject3/Subject3_Traj2_Point2_EMGepochs.png]

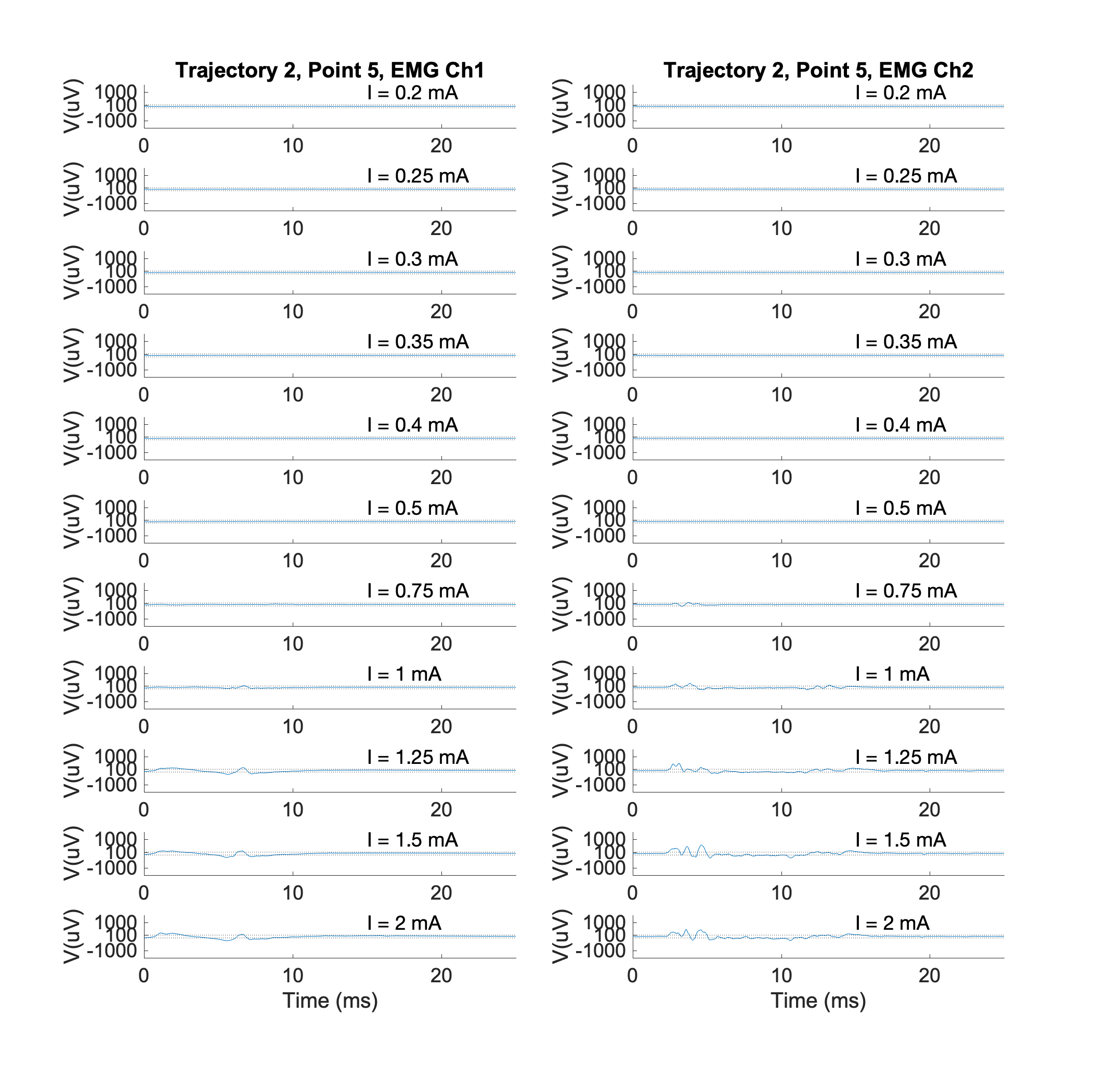

Supplement: Supplementary Data Sheet 1 — Overview of recorded electromyography data showing CMAP responses to the stimulation intensity ramp at each measurement point for the monopolar stimulation. A graph with maximum CMAP responses of monopolar stimulation for each trajectory is depicted. A Summary report (Subject 1, 2, 3.docx) of CMAP responses (for monopolar stimulation) in trajectories with potential FN damage are presented. Data sets of bipolar stimulation can be shared if the reader is interested (see Data Availability Statement). [file Data_Sheet_1.ZIP › Analysis_EMG_Amplitude_Changes/EMGAmp_OutputData/Subject3/Subject3_Traj2_Point5_EMGepochs.png]

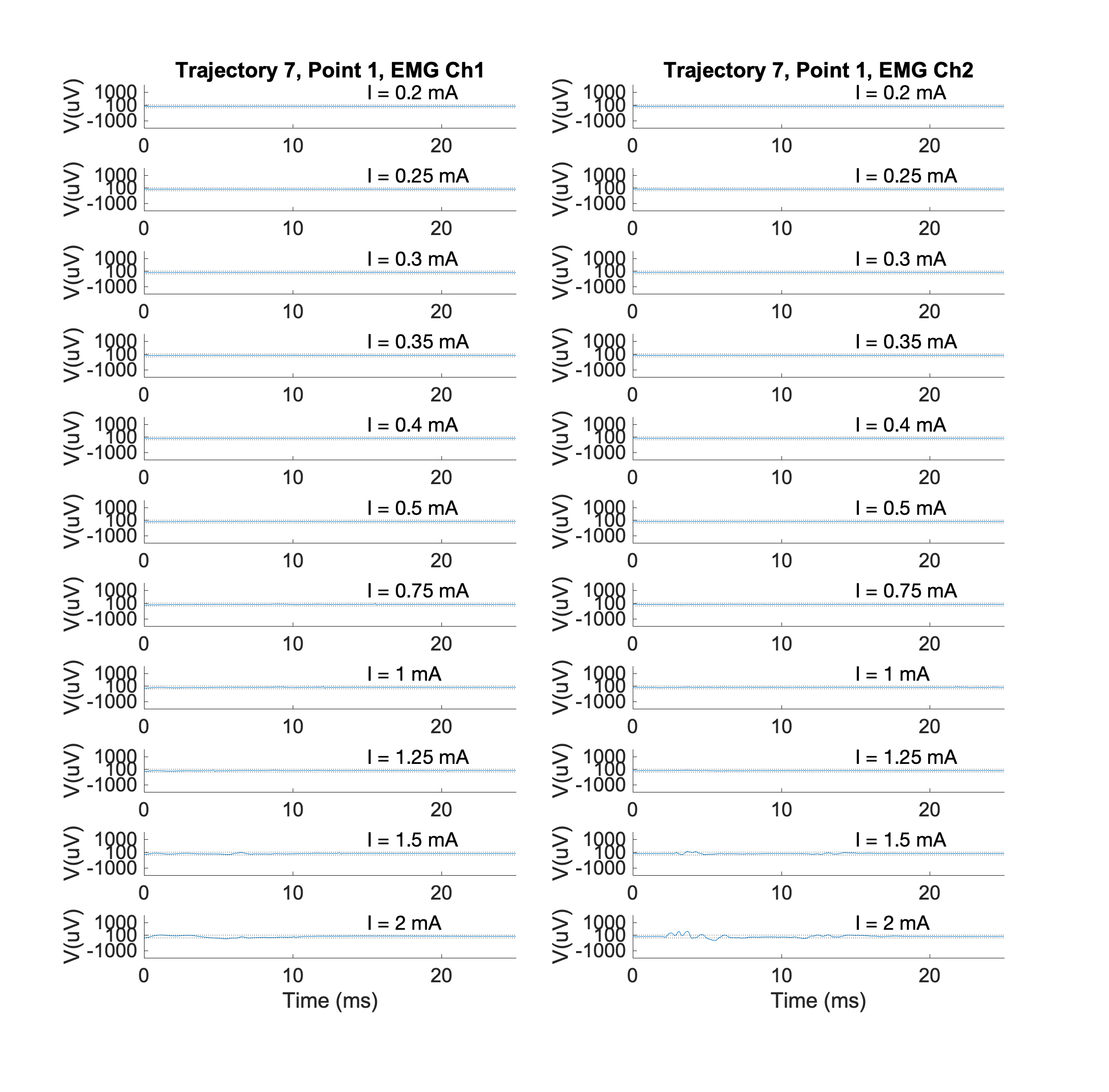

Supplement: Supplementary Data Sheet 1 — Overview of recorded electromyography data showing CMAP responses to the stimulation intensity ramp at each measurement point for the monopolar stimulation. A graph with maximum CMAP responses of monopolar stimulation for each trajectory is depicted. A Summary report (Subject 1, 2, 3.docx) of CMAP responses (for monopolar stimulation) in trajectories with potential FN damage are presented. Data sets of bipolar stimulation can be shared if the reader is interested (see Data Availability Statement). [file Data_Sheet_1.ZIP › Analysis_EMG_Amplitude_Changes/EMGAmp_OutputData/Subject3/Subject3_Traj7_Point1_EMGepochs.png]

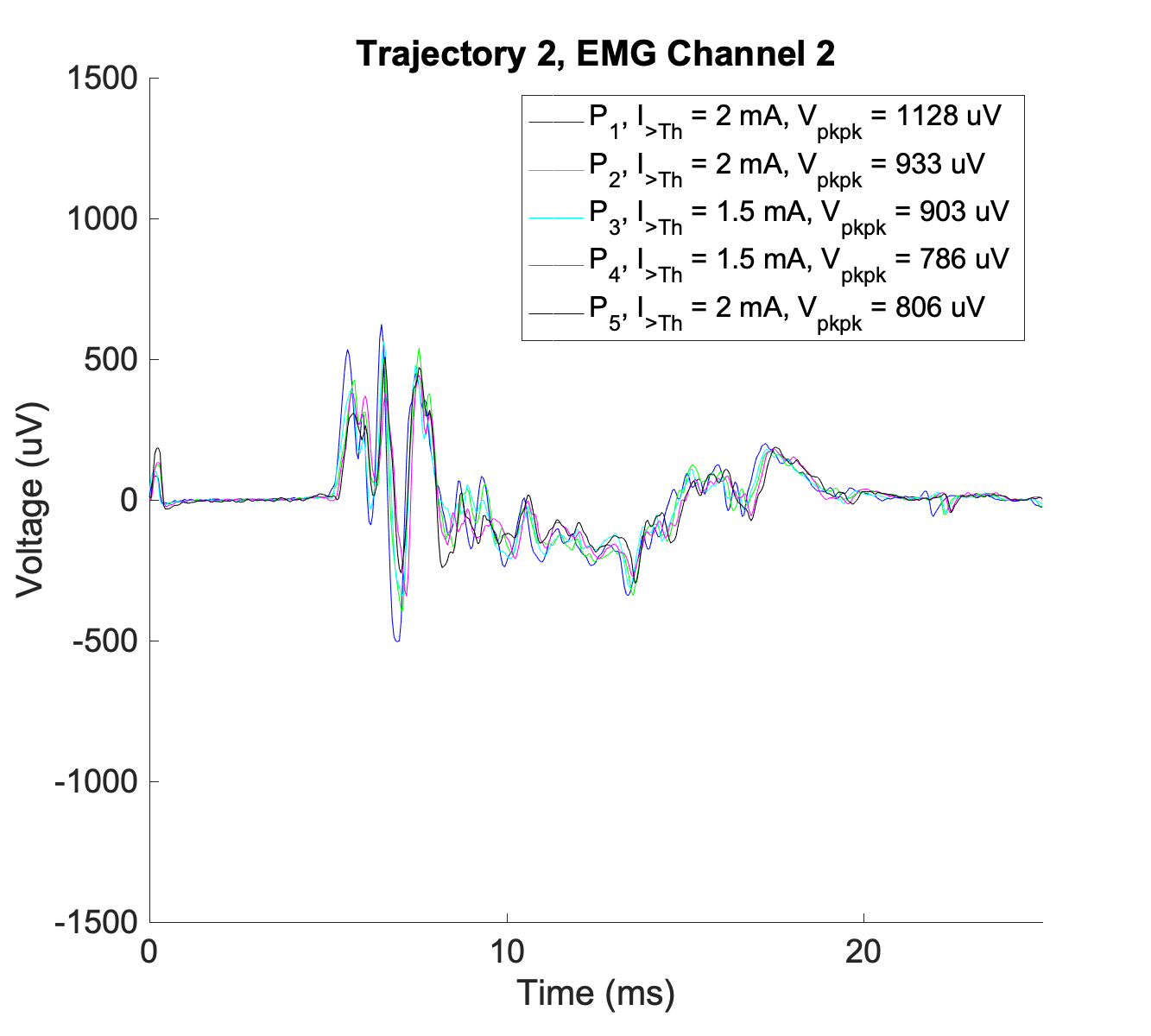

Supplement: Supplementary Data Sheet 1 — Overview of recorded electromyography data showing CMAP responses to the stimulation intensity ramp at each measurement point for the monopolar stimulation. A graph with maximum CMAP responses of monopolar stimulation for each trajectory is depicted. A Summary report (Subject 1, 2, 3.docx) of CMAP responses (for monopolar stimulation) in trajectories with potential FN damage are presented. Data sets of bipolar stimulation can be shared if the reader is interested (see Data Availability Statement). [file Data_Sheet_1.ZIP › Analysis_EMG_Amplitude_Changes/EMGAmp_OutputData/Subject3/Subject3_Traj2_AllPoints_EMG_CH2.png]

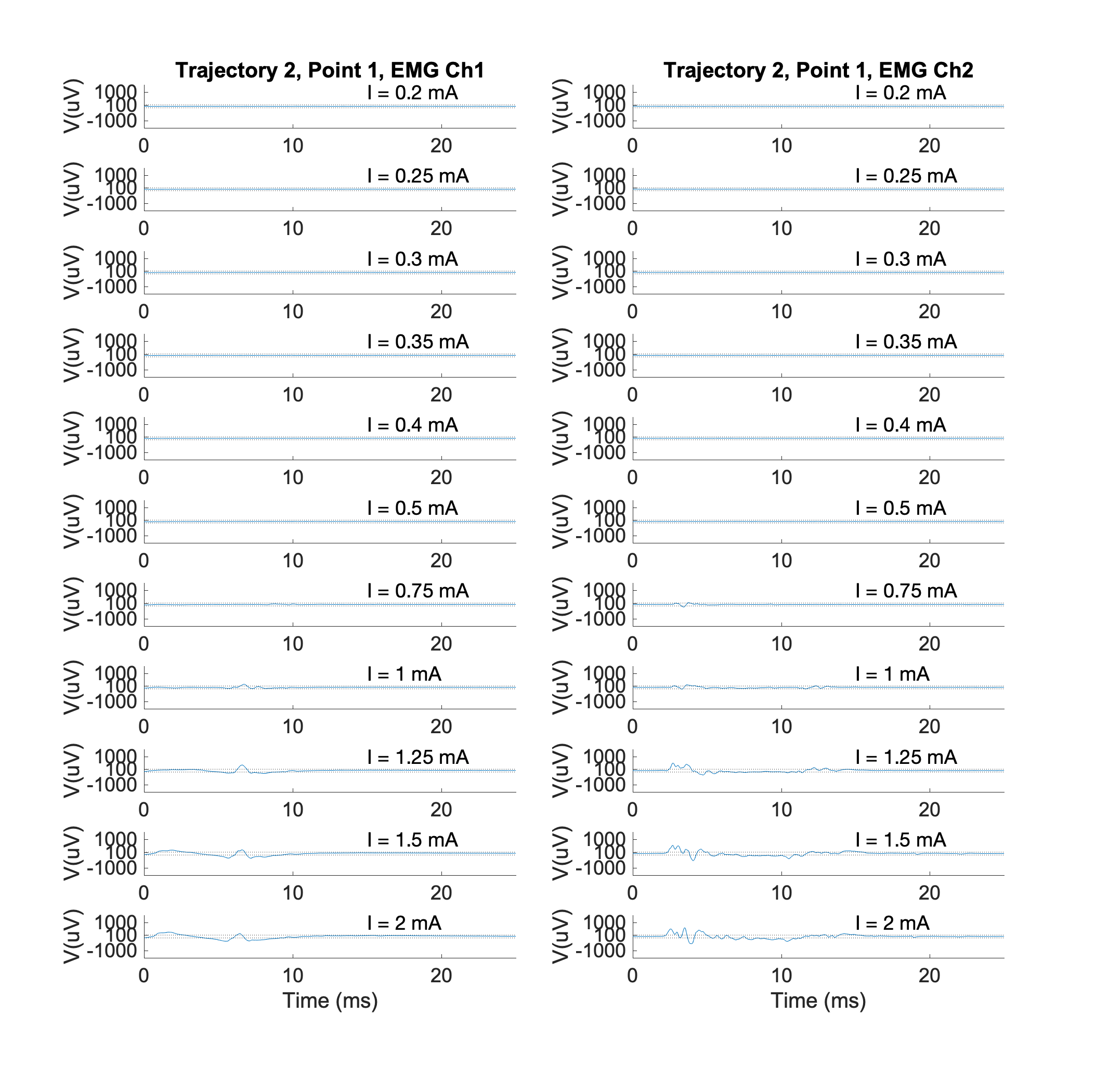

Supplement: Supplementary Data Sheet 1 — Overview of recorded electromyography data showing CMAP responses to the stimulation intensity ramp at each measurement point for the monopolar stimulation. A graph with maximum CMAP responses of monopolar stimulation for each trajectory is depicted. A Summary report (Subject 1, 2, 3.docx) of CMAP responses (for monopolar stimulation) in trajectories with potential FN damage are presented. Data sets of bipolar stimulation can be shared if the reader is interested (see Data Availability Statement). [file Data_Sheet_1.ZIP › Analysis_EMG_Amplitude_Changes/EMGAmp_OutputData/Subject3/Subject3_Traj2_Point1_EMGepochs.png]

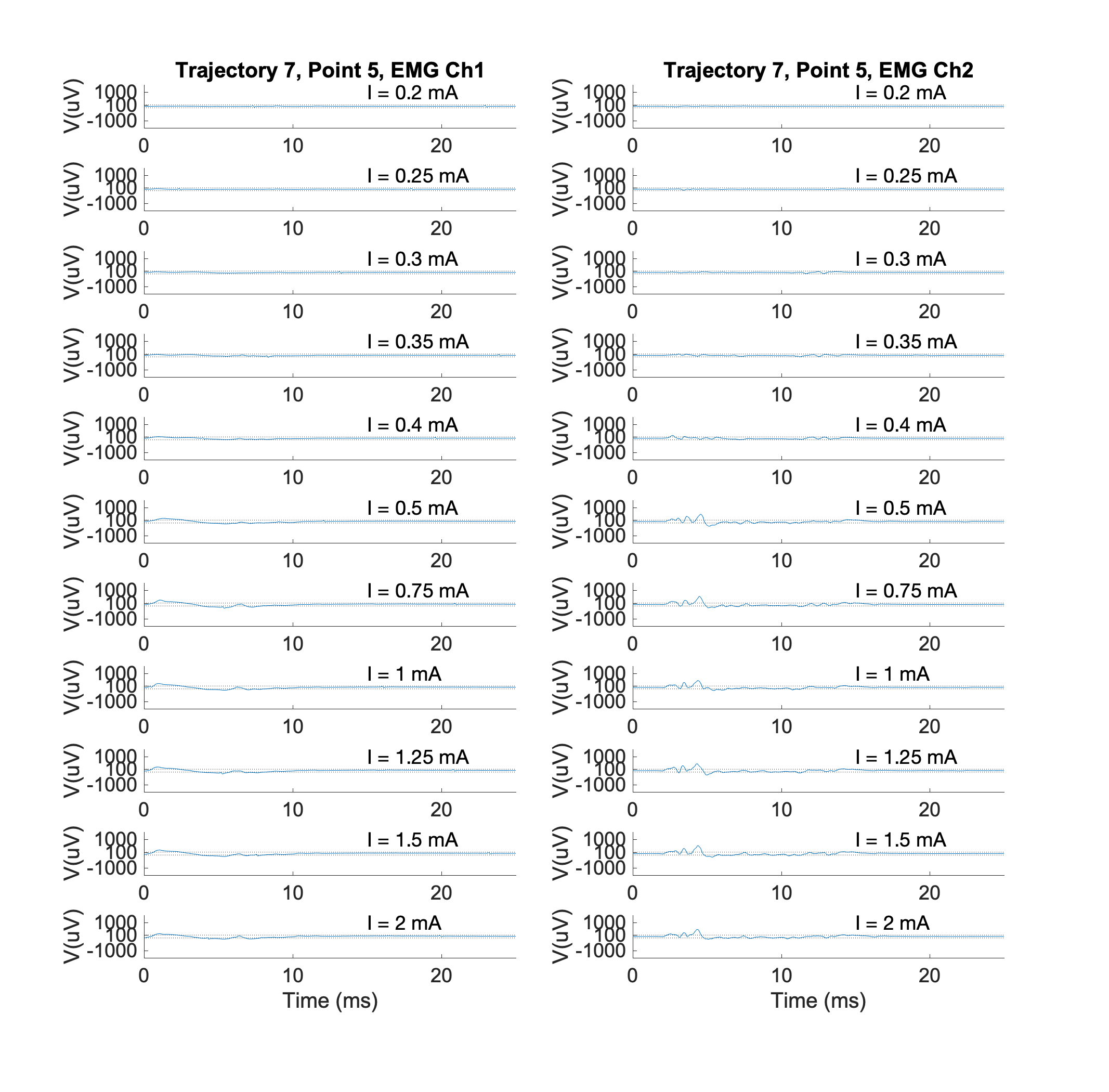

Supplement: Supplementary Data Sheet 1 — Overview of recorded electromyography data showing CMAP responses to the stimulation intensity ramp at each measurement point for the monopolar stimulation. A graph with maximum CMAP responses of monopolar stimulation for each trajectory is depicted. A Summary report (Subject 1, 2, 3.docx) of CMAP responses (for monopolar stimulation) in trajectories with potential FN damage are presented. Data sets of bipolar stimulation can be shared if the reader is interested (see Data Availability Statement). [file Data_Sheet_1.ZIP › Analysis_EMG_Amplitude_Changes/EMGAmp_OutputData/Subject3/Subject3_Traj7_Point5_EMGepochs.png]

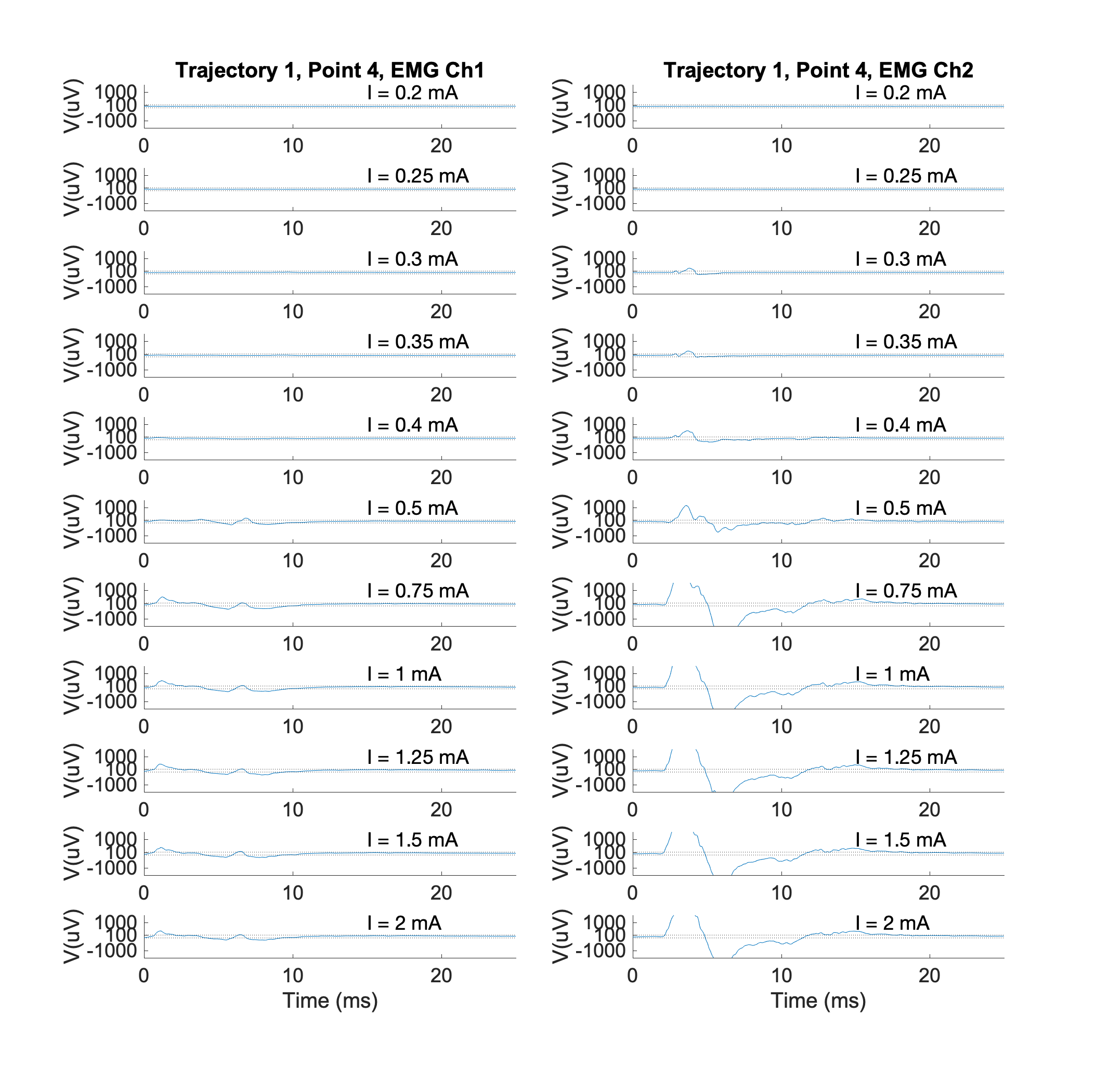

Supplement: Supplementary Data Sheet 1 — Overview of recorded electromyography data showing CMAP responses to the stimulation intensity ramp at each measurement point for the monopolar stimulation. A graph with maximum CMAP responses of monopolar stimulation for each trajectory is depicted. A Summary report (Subject 1, 2, 3.docx) of CMAP responses (for monopolar stimulation) in trajectories with potential FN damage are presented. Data sets of bipolar stimulation can be shared if the reader is interested (see Data Availability Statement). [file Data_Sheet_1.ZIP › Analysis_EMG_Amplitude_Changes/EMGAmp_OutputData/Subject3/Subject3_Traj1_Point4_EMGepochs.png]

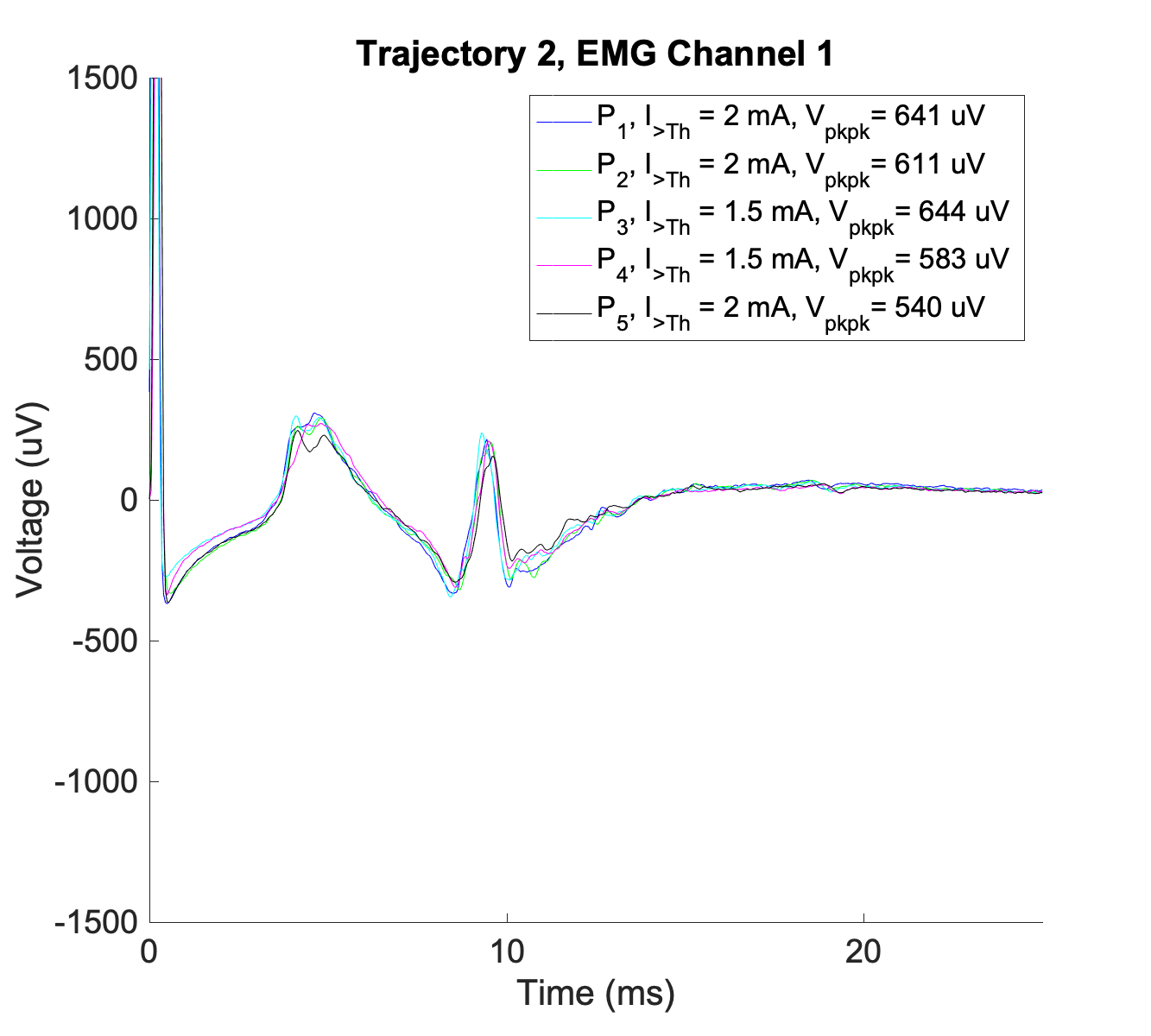

Supplement: Supplementary Data Sheet 1 — Overview of recorded electromyography data showing CMAP responses to the stimulation intensity ramp at each measurement point for the monopolar stimulation. A graph with maximum CMAP responses of monopolar stimulation for each trajectory is depicted. A Summary report (Subject 1, 2, 3.docx) of CMAP responses (for monopolar stimulation) in trajectories with potential FN damage are presented. Data sets of bipolar stimulation can be shared if the reader is interested (see Data Availability Statement). [file Data_Sheet_1.ZIP › Analysis_EMG_Amplitude_Changes/EMGAmp_OutputData/Subject3/Subject3_Traj2_AllPoints_EMG_CH1.png]

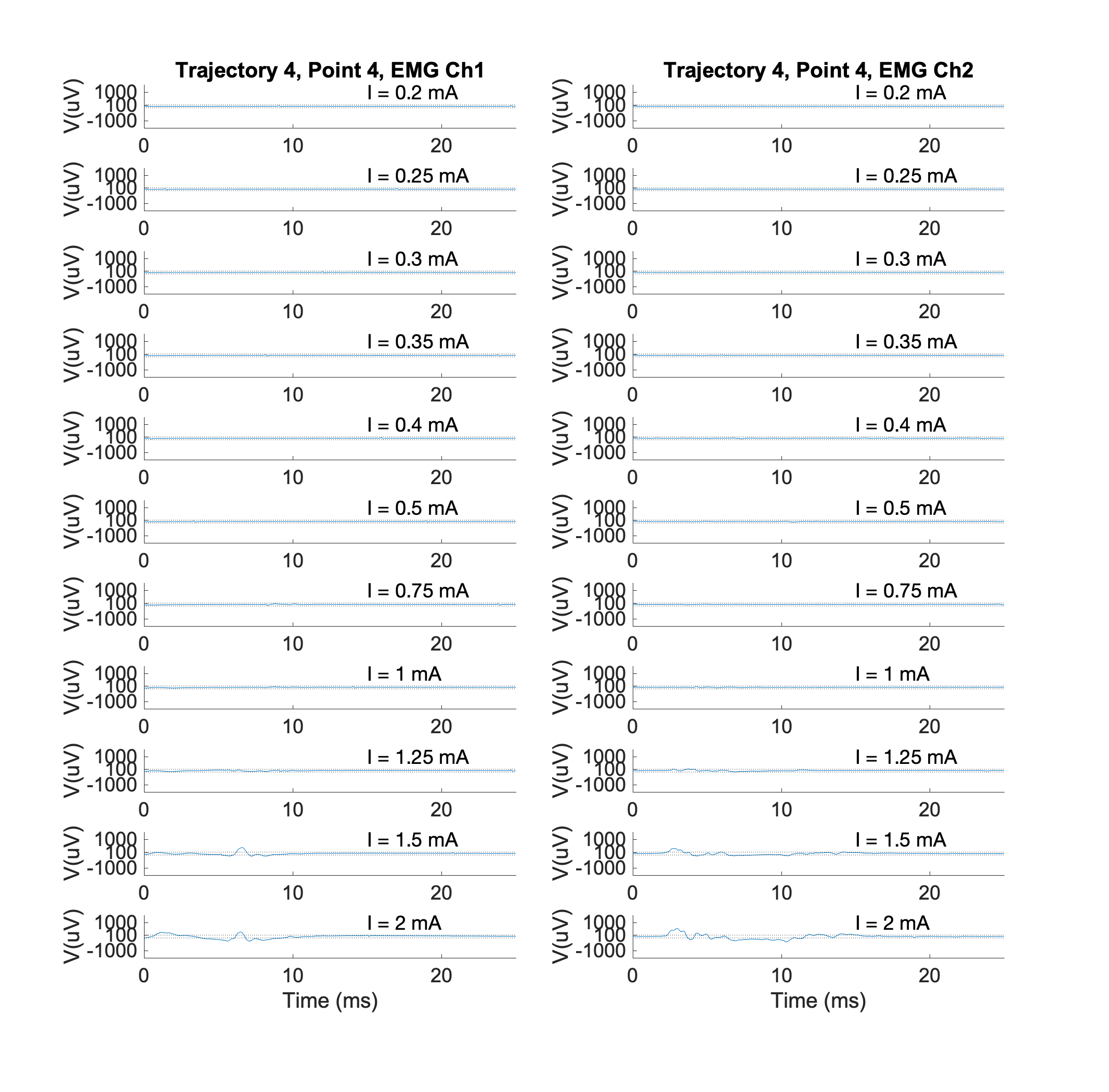

Supplement: Supplementary Data Sheet 1 — Overview of recorded electromyography data showing CMAP responses to the stimulation intensity ramp at each measurement point for the monopolar stimulation. A graph with maximum CMAP responses of monopolar stimulation for each trajectory is depicted. A Summary report (Subject 1, 2, 3.docx) of CMAP responses (for monopolar stimulation) in trajectories with potential FN damage are presented. Data sets of bipolar stimulation can be shared if the reader is interested (see Data Availability Statement). [file Data_Sheet_1.ZIP › Analysis_EMG_Amplitude_Changes/EMGAmp_OutputData/Subject3/Subject3_Traj4_Point4_EMGepochs.png]

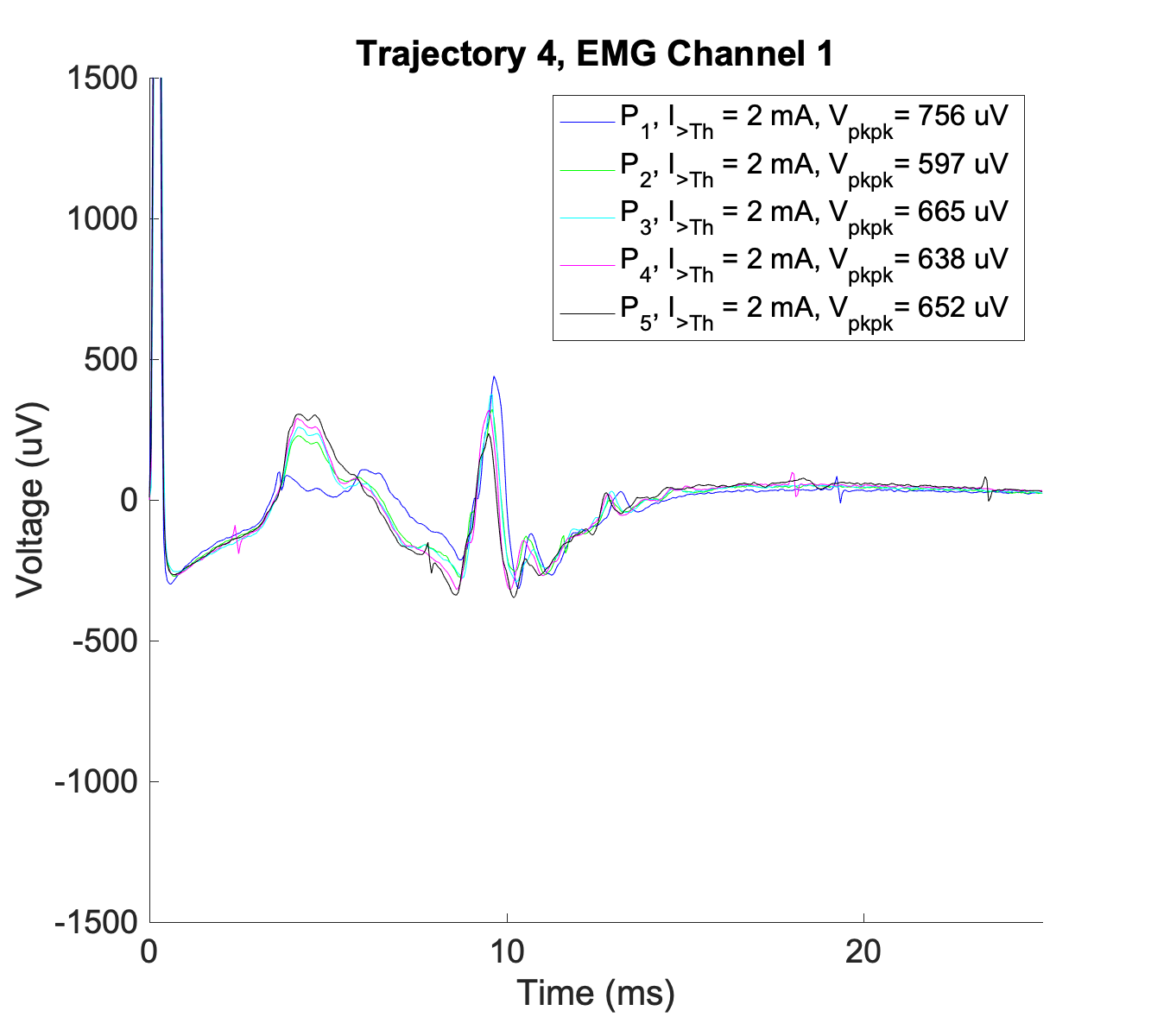

Supplement: Supplementary Data Sheet 1 — Overview of recorded electromyography data showing CMAP responses to the stimulation intensity ramp at each measurement point for the monopolar stimulation. A graph with maximum CMAP responses of monopolar stimulation for each trajectory is depicted. A Summary report (Subject 1, 2, 3.docx) of CMAP responses (for monopolar stimulation) in trajectories with potential FN damage are presented. Data sets of bipolar stimulation can be shared if the reader is interested (see Data Availability Statement). [file Data_Sheet_1.ZIP › Analysis_EMG_Amplitude_Changes/EMGAmp_OutputData/Subject3/Subject3_Traj4_AllPoints_EMG_CH1.png]

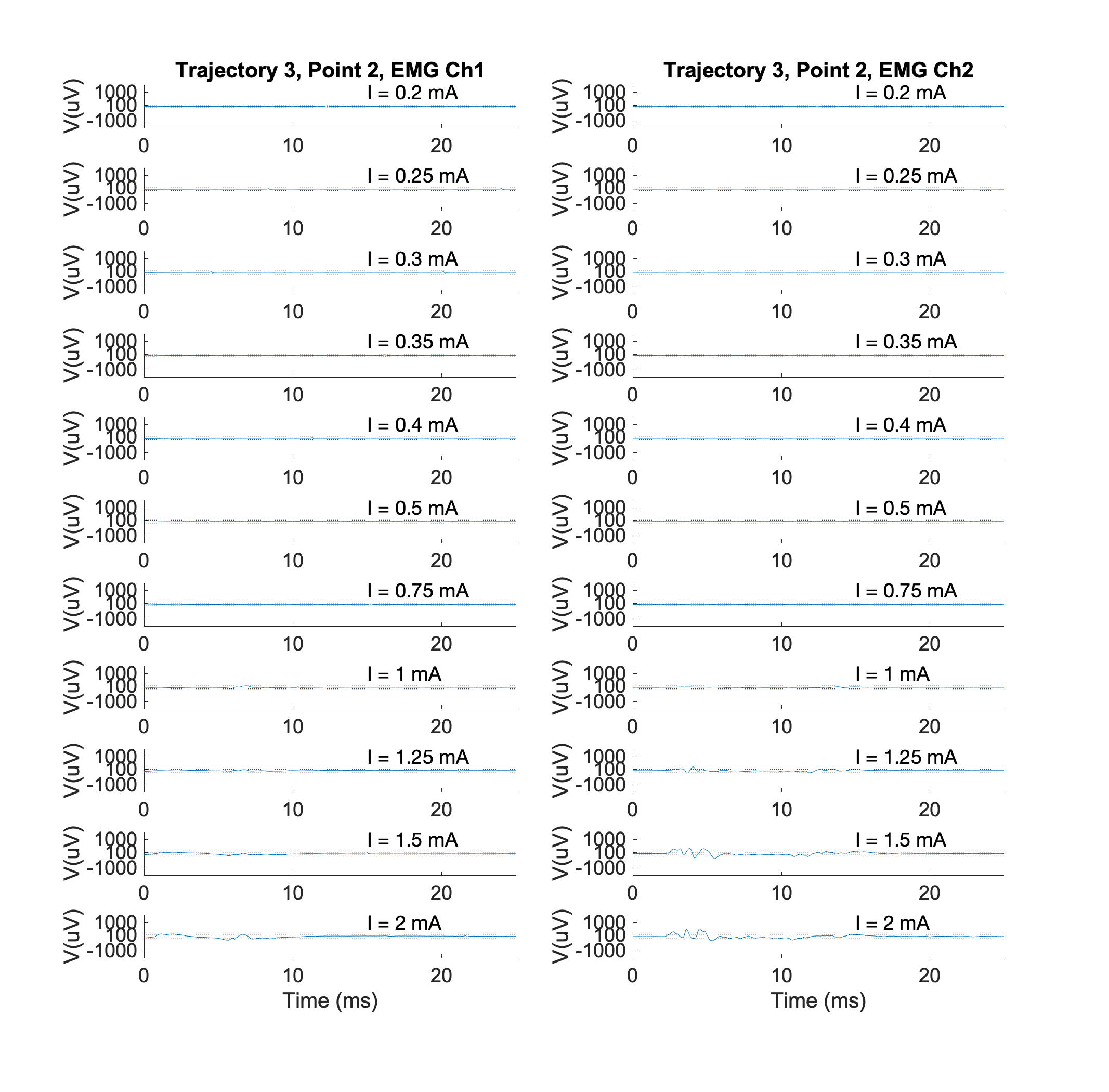

Supplement: Supplementary Data Sheet 1 — Overview of recorded electromyography data showing CMAP responses to the stimulation intensity ramp at each measurement point for the monopolar stimulation. A graph with maximum CMAP responses of monopolar stimulation for each trajectory is depicted. A Summary report (Subject 1, 2, 3.docx) of CMAP responses (for monopolar stimulation) in trajectories with potential FN damage are presented. Data sets of bipolar stimulation can be shared if the reader is interested (see Data Availability Statement). [file Data_Sheet_1.ZIP › Analysis_EMG_Amplitude_Changes/EMGAmp_OutputData/Subject3/Subject3_Traj3_Point2_EMGepochs.png]

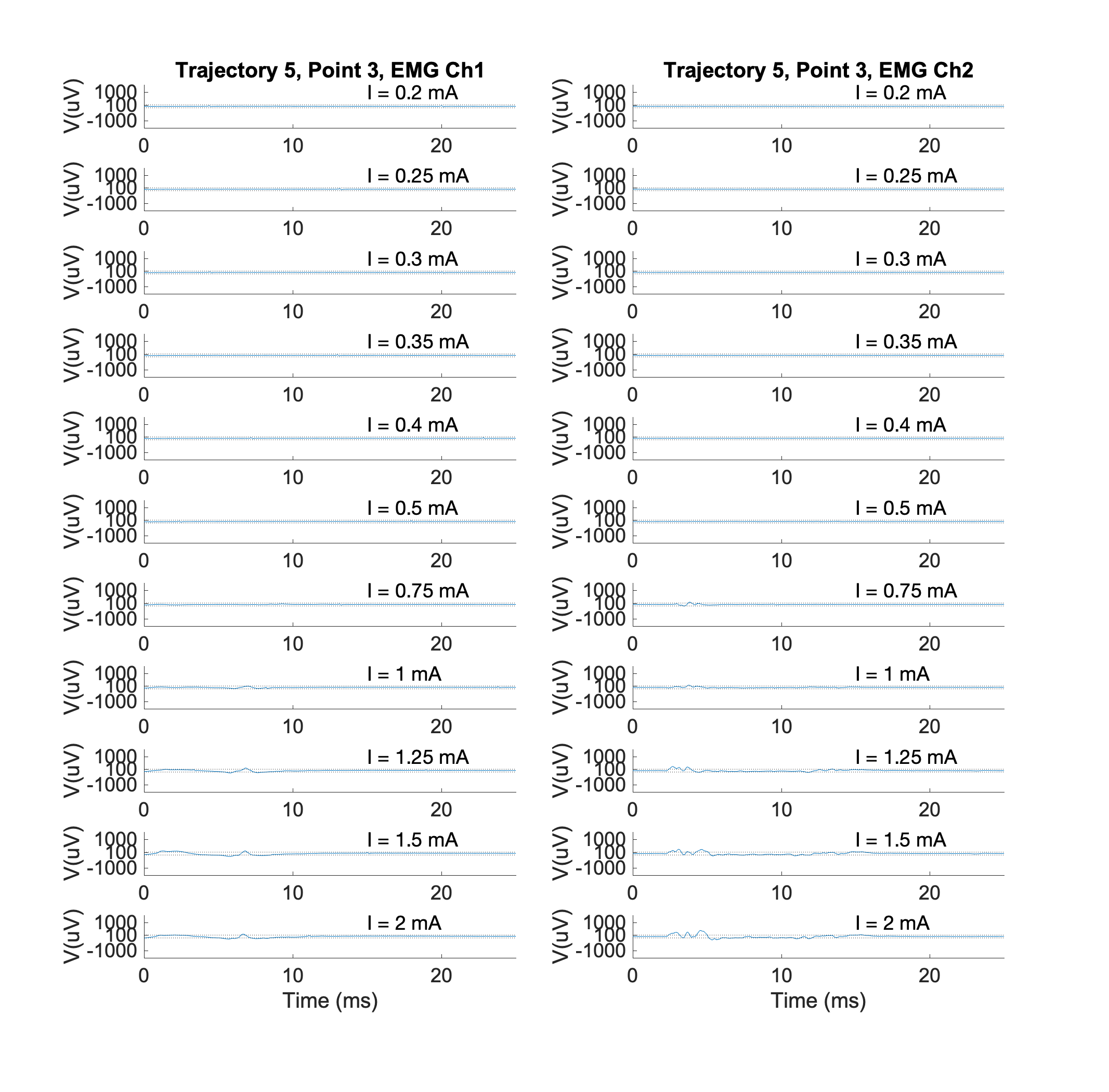

Supplement: Supplementary Data Sheet 1 — Overview of recorded electromyography data showing CMAP responses to the stimulation intensity ramp at each measurement point for the monopolar stimulation. A graph with maximum CMAP responses of monopolar stimulation for each trajectory is depicted. A Summary report (Subject 1, 2, 3.docx) of CMAP responses (for monopolar stimulation) in trajectories with potential FN damage are presented. Data sets of bipolar stimulation can be shared if the reader is interested (see Data Availability Statement). [file Data_Sheet_1.ZIP › Analysis_EMG_Amplitude_Changes/EMGAmp_OutputData/Subject3/Subject3_Traj5_Point3_EMGepochs.png]

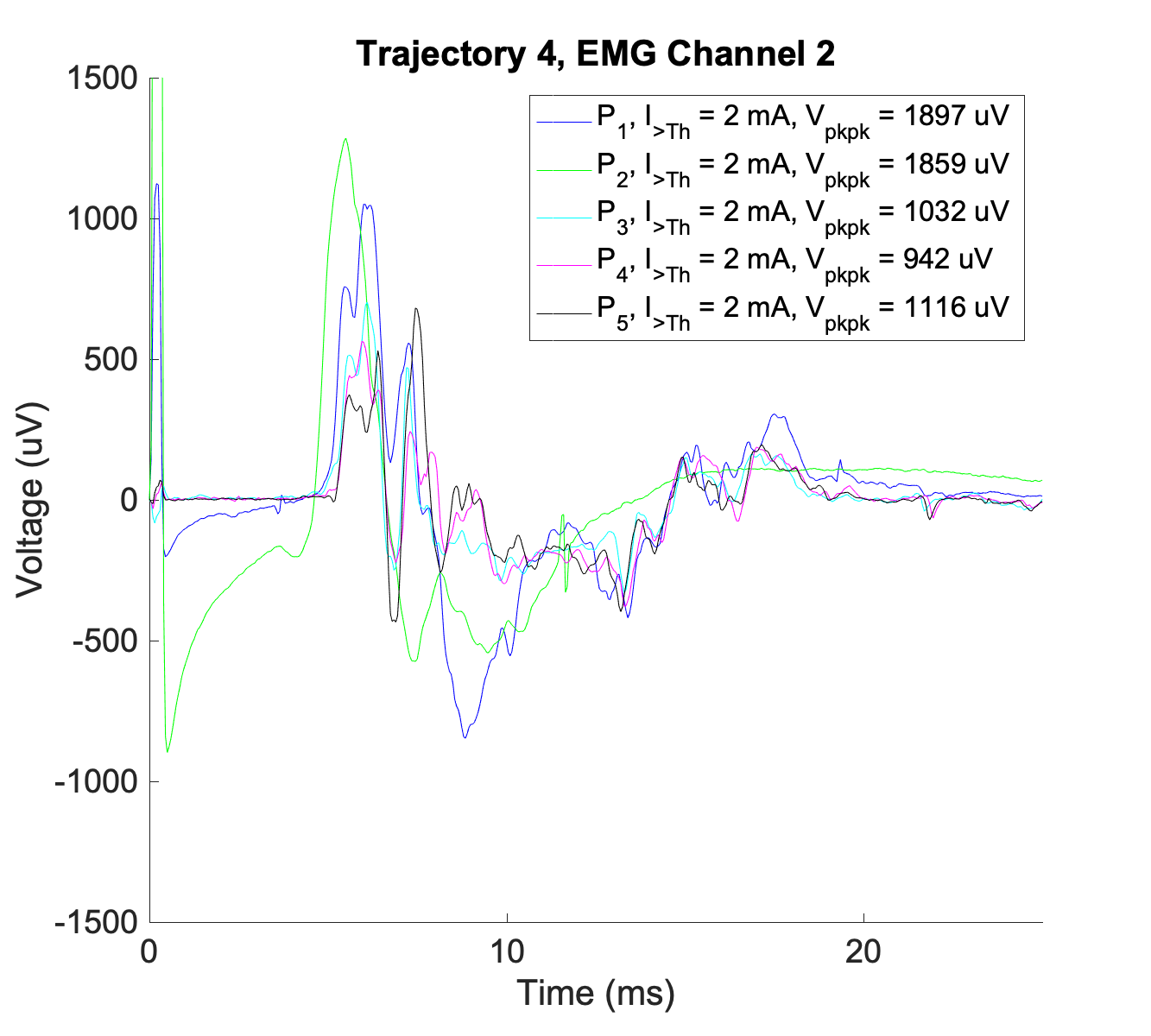

Supplement: Supplementary Data Sheet 1 — Overview of recorded electromyography data showing CMAP responses to the stimulation intensity ramp at each measurement point for the monopolar stimulation. A graph with maximum CMAP responses of monopolar stimulation for each trajectory is depicted. A Summary report (Subject 1, 2, 3.docx) of CMAP responses (for monopolar stimulation) in trajectories with potential FN damage are presented. Data sets of bipolar stimulation can be shared if the reader is interested (see Data Availability Statement). [file Data_Sheet_1.ZIP › Analysis_EMG_Amplitude_Changes/EMGAmp_OutputData/Subject3/Subject3_Traj4_AllPoints_EMG_CH2.png]

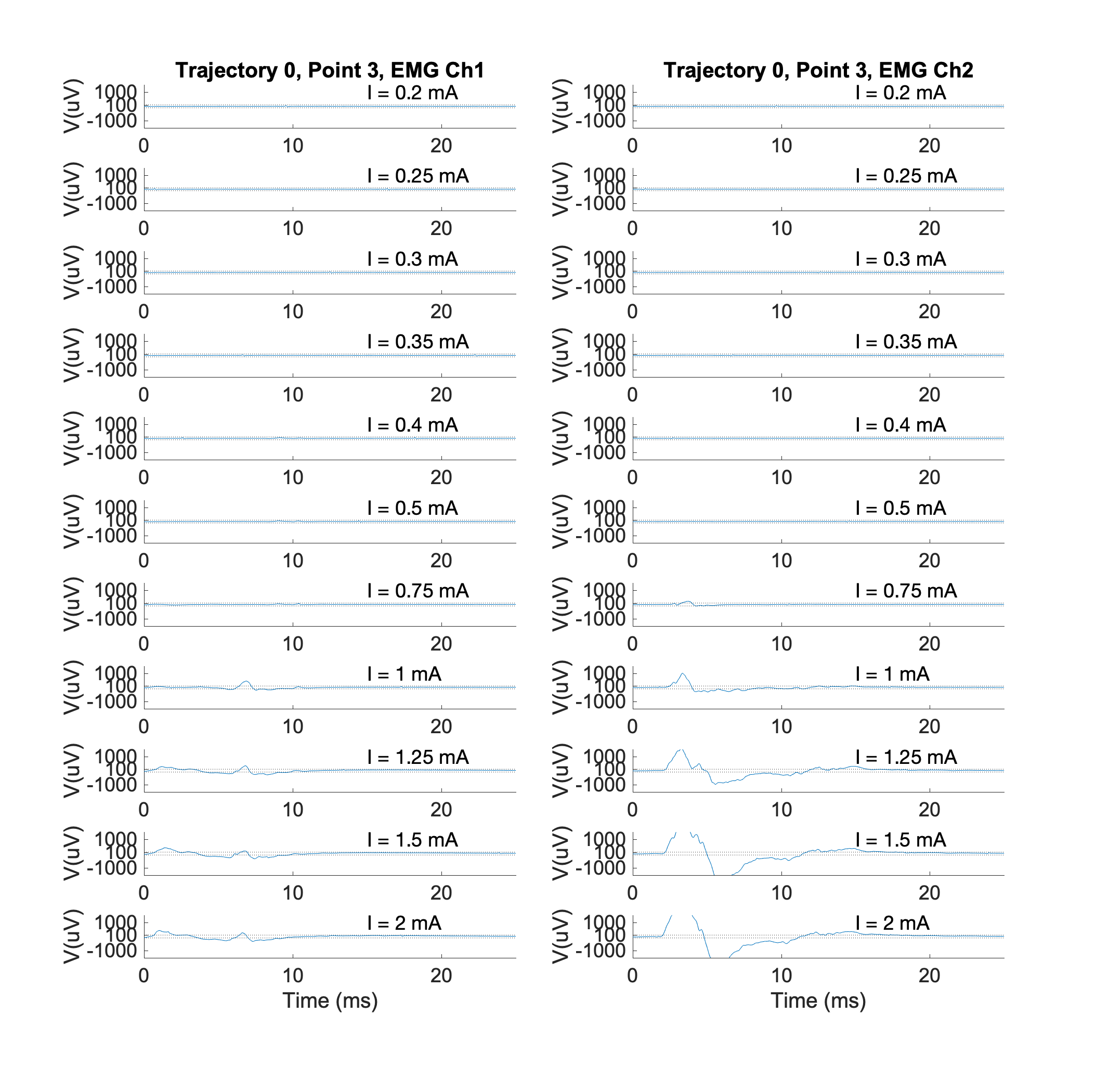

Supplement: Supplementary Data Sheet 1 — Overview of recorded electromyography data showing CMAP responses to the stimulation intensity ramp at each measurement point for the monopolar stimulation. A graph with maximum CMAP responses of monopolar stimulation for each trajectory is depicted. A Summary report (Subject 1, 2, 3.docx) of CMAP responses (for monopolar stimulation) in trajectories with potential FN damage are presented. Data sets of bipolar stimulation can be shared if the reader is interested (see Data Availability Statement). [file Data_Sheet_1.ZIP › Analysis_EMG_Amplitude_Changes/EMGAmp_OutputData/Subject3/Subject3_Traj0_Point3_EMGepochs.png]

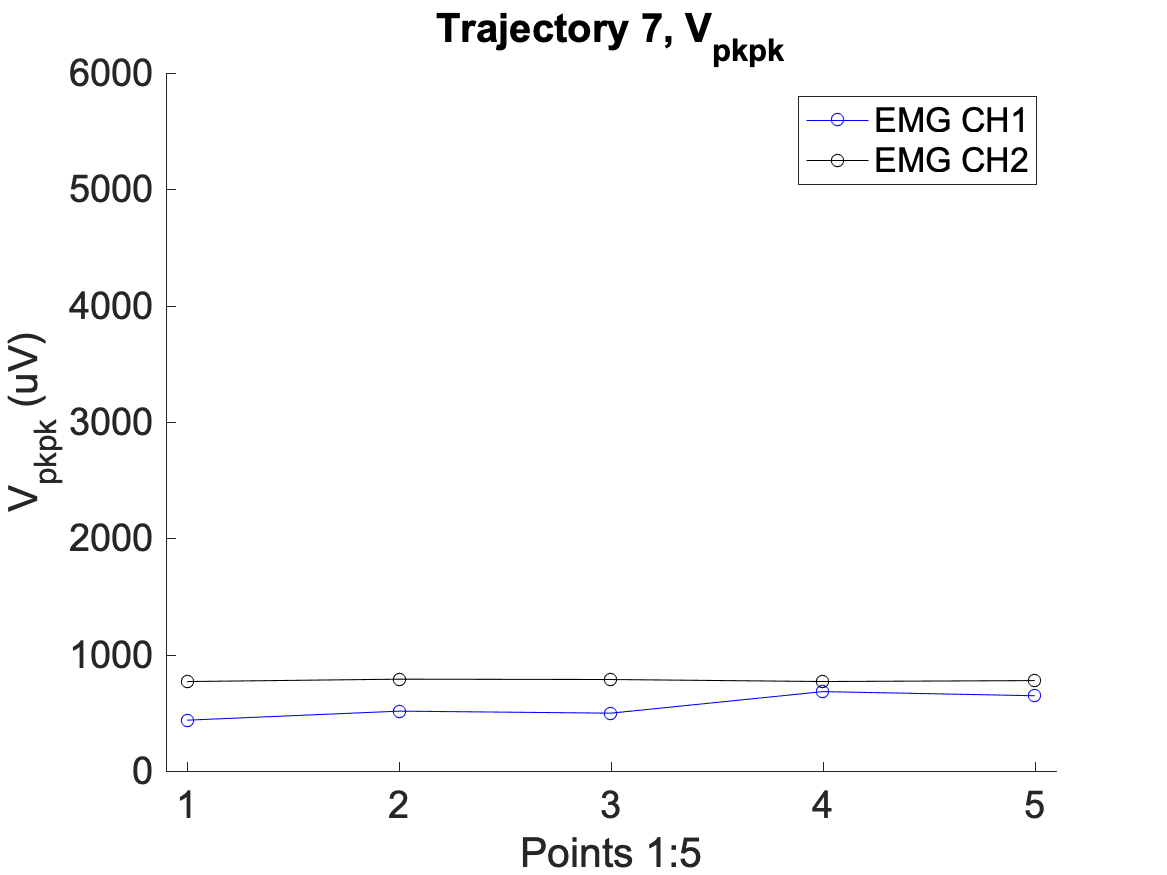

Supplement: Supplementary Data Sheet 1 — Overview of recorded electromyography data showing CMAP responses to the stimulation intensity ramp at each measurement point for the monopolar stimulation. A graph with maximum CMAP responses of monopolar stimulation for each trajectory is depicted. A Summary report (Subject 1, 2, 3.docx) of CMAP responses (for monopolar stimulation) in trajectories with potential FN damage are presented. Data sets of bipolar stimulation can be shared if the reader is interested (see Data Availability Statement). [file Data_Sheet_1.ZIP › Analysis_EMG_Amplitude_Changes/EMGAmp_OutputData/Subject3/Subject3_Traj7_Vpkk.png]

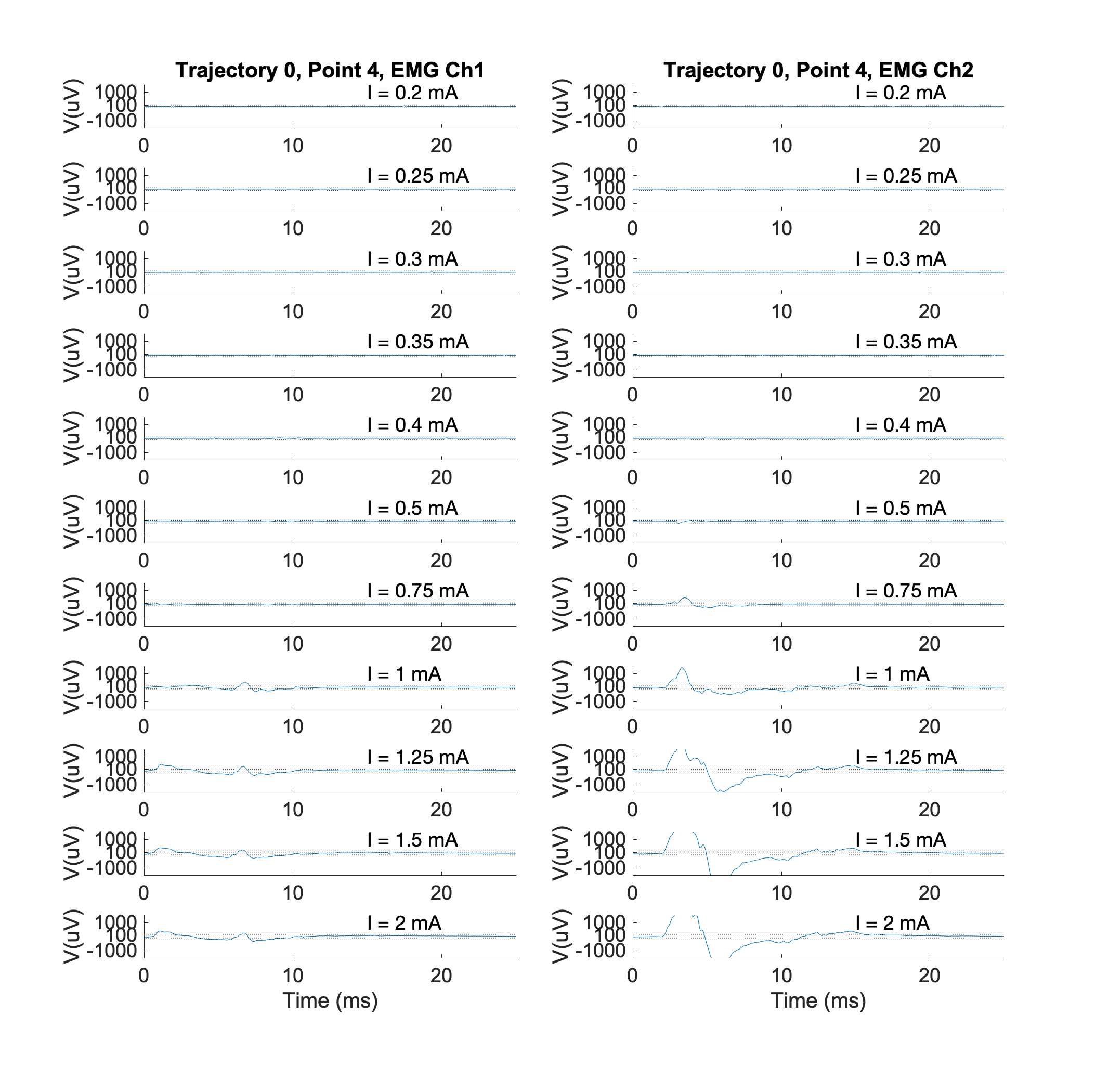

Supplement: Supplementary Data Sheet 1 — Overview of recorded electromyography data showing CMAP responses to the stimulation intensity ramp at each measurement point for the monopolar stimulation. A graph with maximum CMAP responses of monopolar stimulation for each trajectory is depicted. A Summary report (Subject 1, 2, 3.docx) of CMAP responses (for monopolar stimulation) in trajectories with potential FN damage are presented. Data sets of bipolar stimulation can be shared if the reader is interested (see Data Availability Statement). [file Data_Sheet_1.ZIP › Analysis_EMG_Amplitude_Changes/EMGAmp_OutputData/Subject3/Subject3_Traj0_Point4_EMGepochs.png]

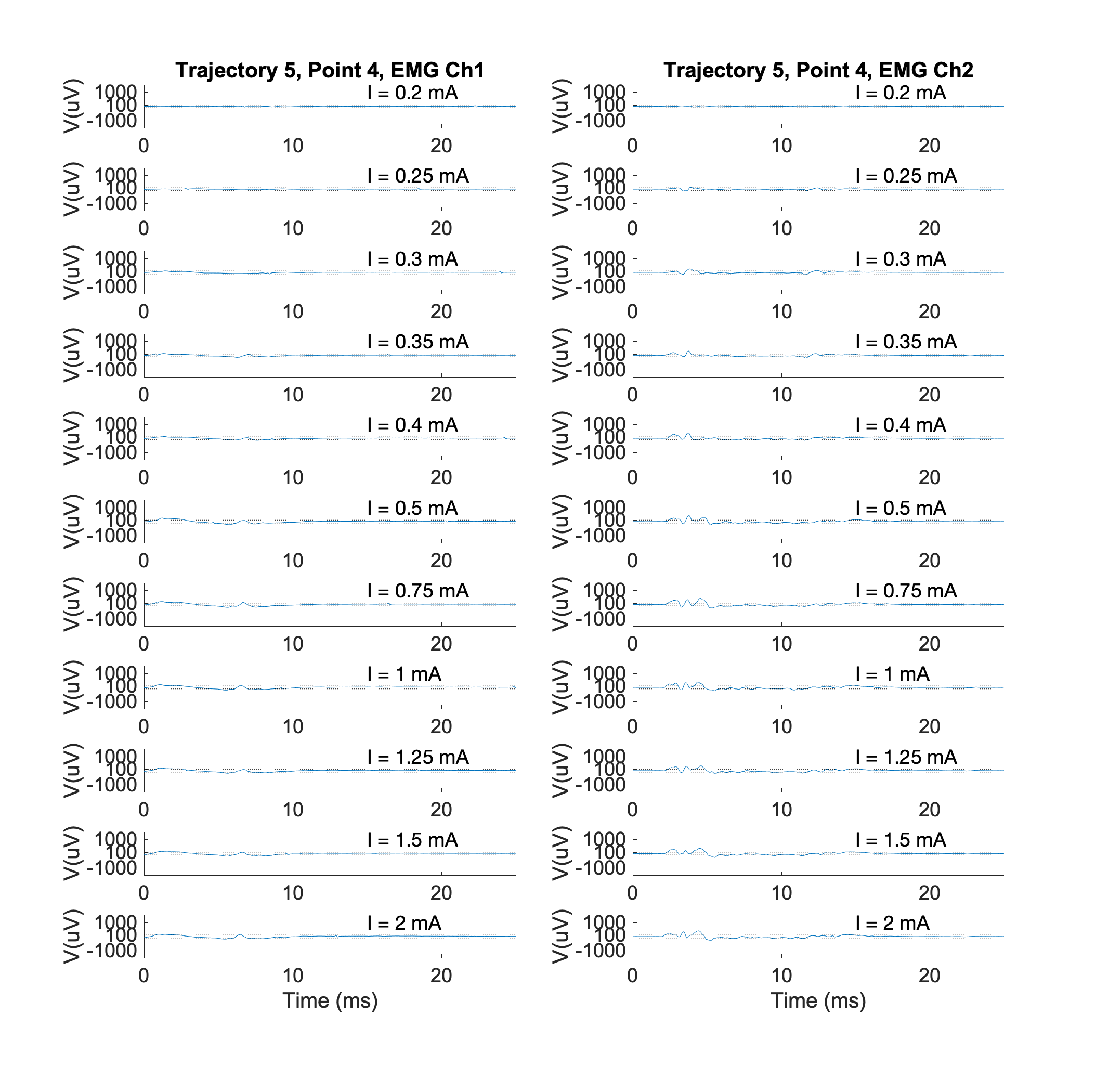

Supplement: Supplementary Data Sheet 1 — Overview of recorded electromyography data showing CMAP responses to the stimulation intensity ramp at each measurement point for the monopolar stimulation. A graph with maximum CMAP responses of monopolar stimulation for each trajectory is depicted. A Summary report (Subject 1, 2, 3.docx) of CMAP responses (for monopolar stimulation) in trajectories with potential FN damage are presented. Data sets of bipolar stimulation can be shared if the reader is interested (see Data Availability Statement). [file Data_Sheet_1.ZIP › Analysis_EMG_Amplitude_Changes/EMGAmp_OutputData/Subject3/Subject3_Traj5_Point4_EMGepochs.png]

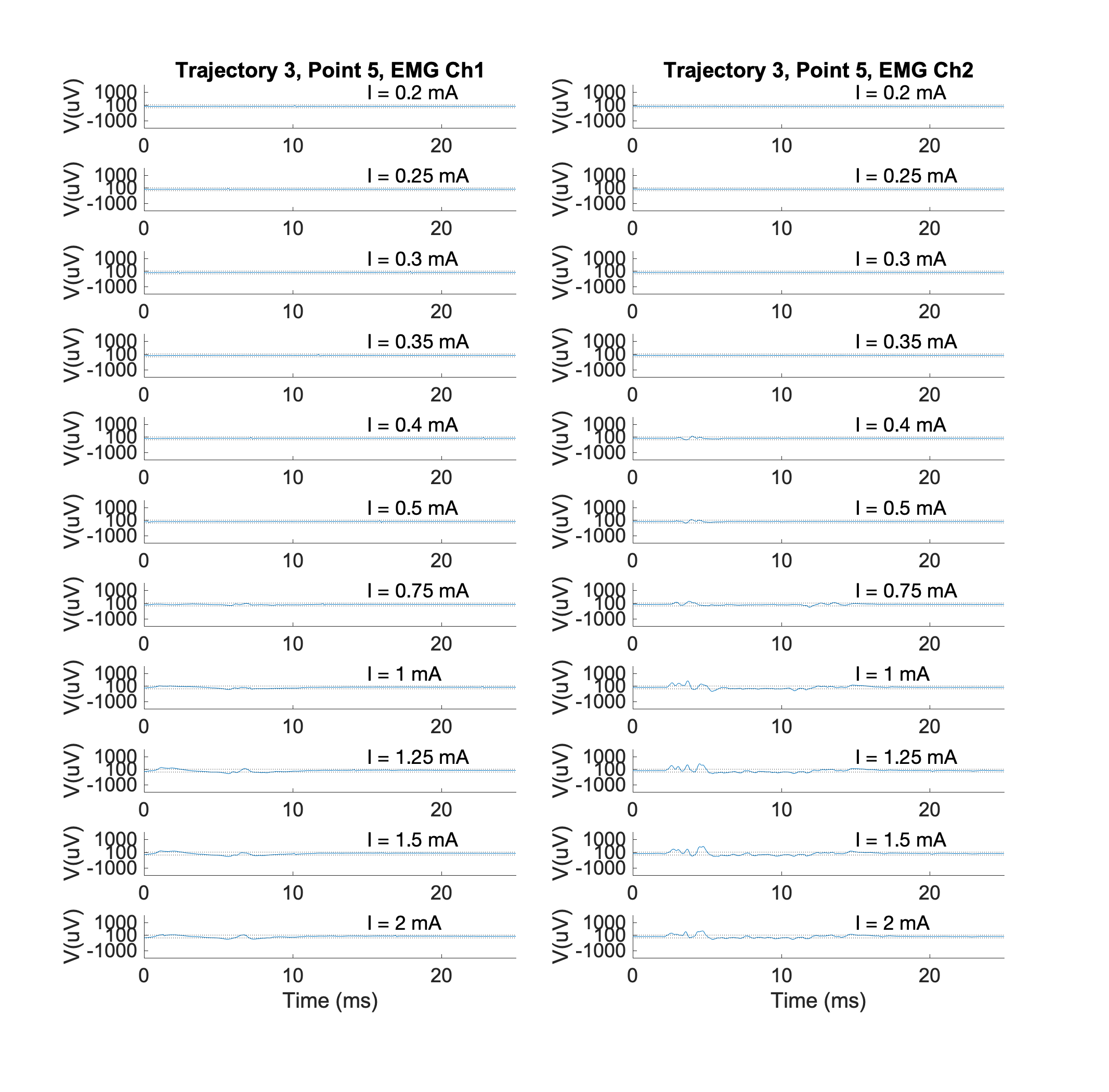

Supplement: Supplementary Data Sheet 1 — Overview of recorded electromyography data showing CMAP responses to the stimulation intensity ramp at each measurement point for the monopolar stimulation. A graph with maximum CMAP responses of monopolar stimulation for each trajectory is depicted. A Summary report (Subject 1, 2, 3.docx) of CMAP responses (for monopolar stimulation) in trajectories with potential FN damage are presented. Data sets of bipolar stimulation can be shared if the reader is interested (see Data Availability Statement). [file Data_Sheet_1.ZIP › Analysis_EMG_Amplitude_Changes/EMGAmp_OutputData/Subject3/Subject3_Traj3_Point5_EMGepochs.png]

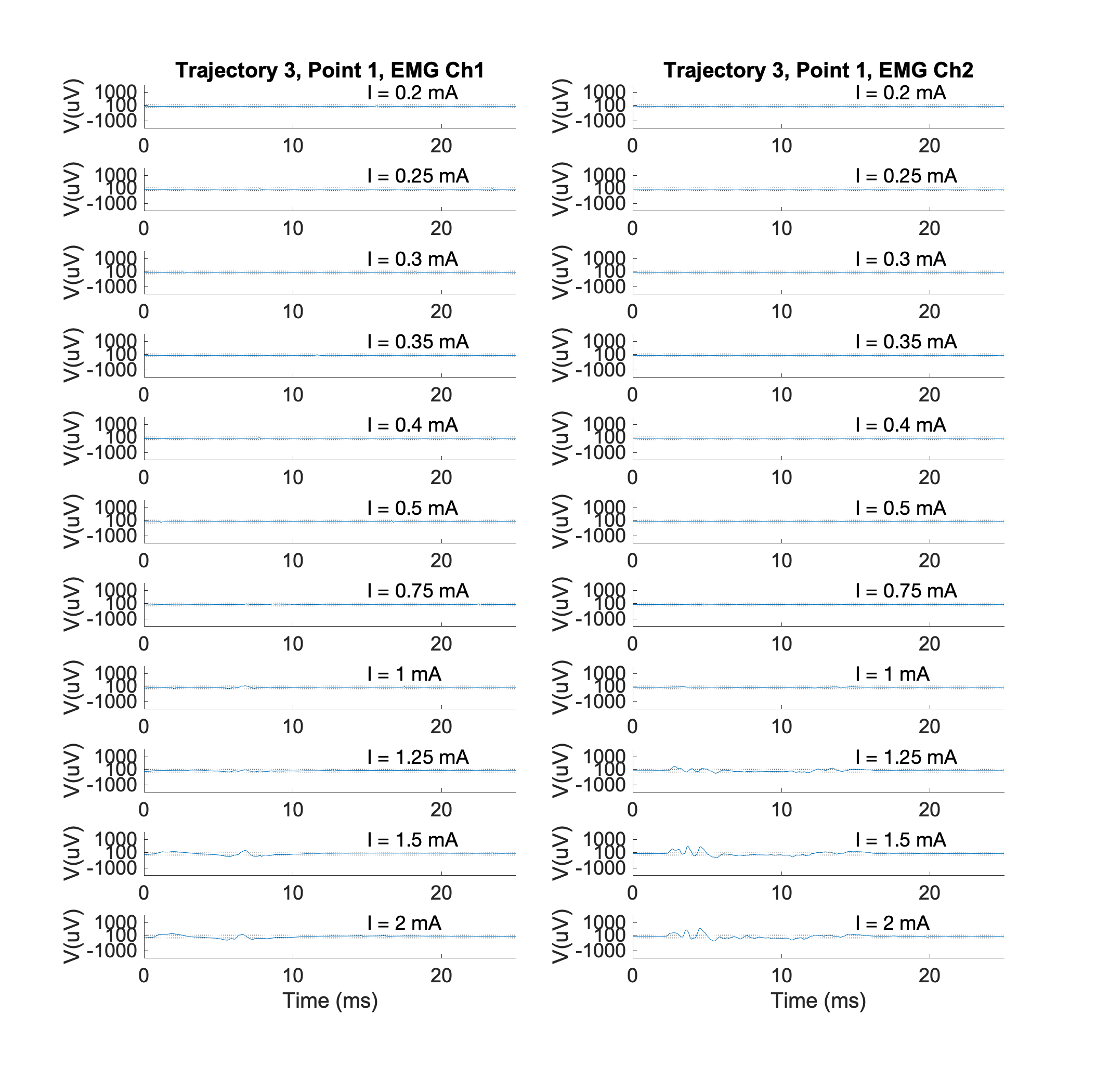

Supplement: Supplementary Data Sheet 1 — Overview of recorded electromyography data showing CMAP responses to the stimulation intensity ramp at each measurement point for the monopolar stimulation. A graph with maximum CMAP responses of monopolar stimulation for each trajectory is depicted. A Summary report (Subject 1, 2, 3.docx) of CMAP responses (for monopolar stimulation) in trajectories with potential FN damage are presented. Data sets of bipolar stimulation can be shared if the reader is interested (see Data Availability Statement). [file Data_Sheet_1.ZIP › Analysis_EMG_Amplitude_Changes/EMGAmp_OutputData/Subject3/Subject3_Traj3_Point1_EMGepochs.png]

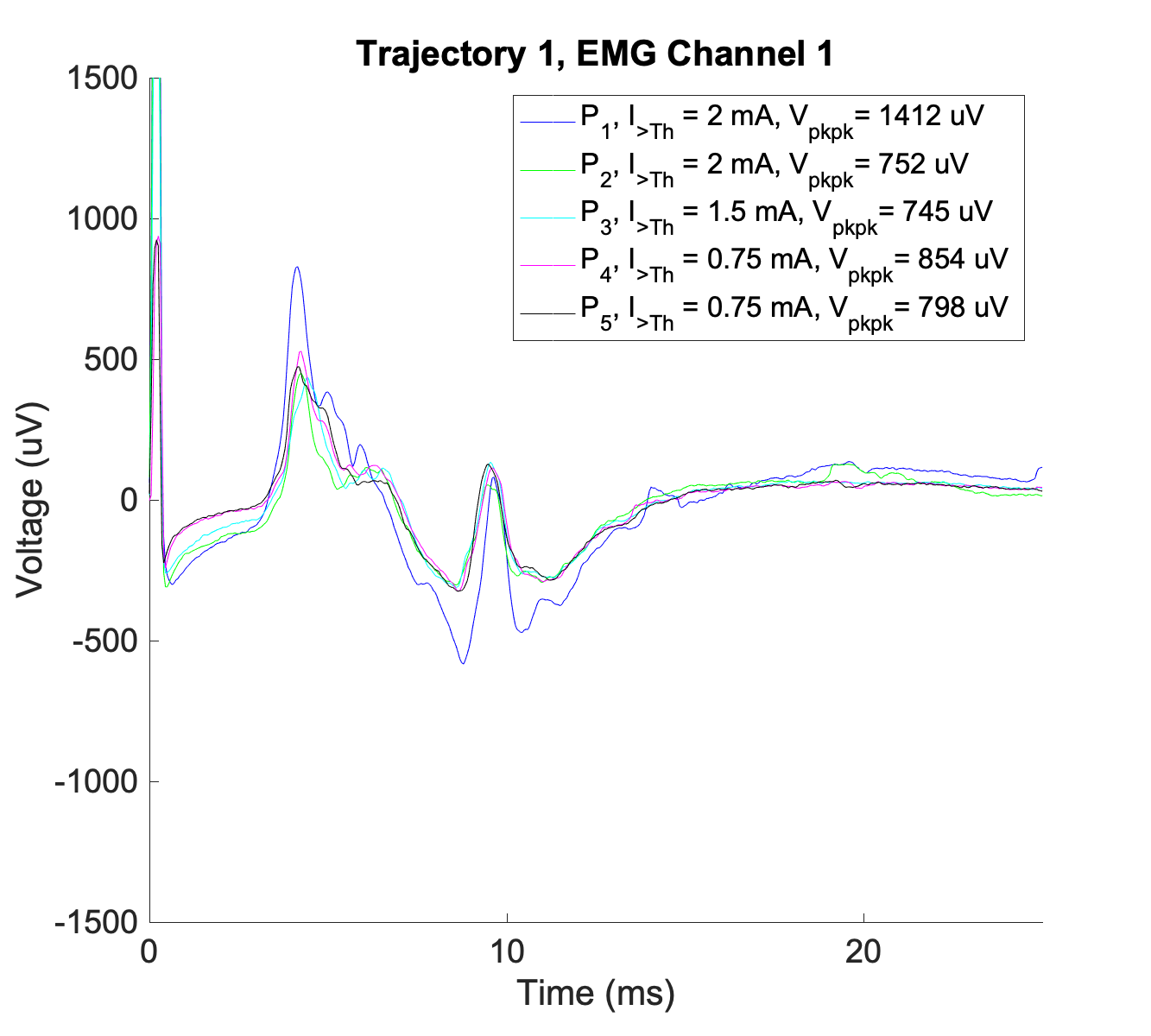

Supplement: Supplementary Data Sheet 1 — Overview of recorded electromyography data showing CMAP responses to the stimulation intensity ramp at each measurement point for the monopolar stimulation. A graph with maximum CMAP responses of monopolar stimulation for each trajectory is depicted. A Summary report (Subject 1, 2, 3.docx) of CMAP responses (for monopolar stimulation) in trajectories with potential FN damage are presented. Data sets of bipolar stimulation can be shared if the reader is interested (see Data Availability Statement). [file Data_Sheet_1.ZIP › Analysis_EMG_Amplitude_Changes/EMGAmp_OutputData/Subject3/Subject3_Traj1_AllPoints_EMG_CH1.png]

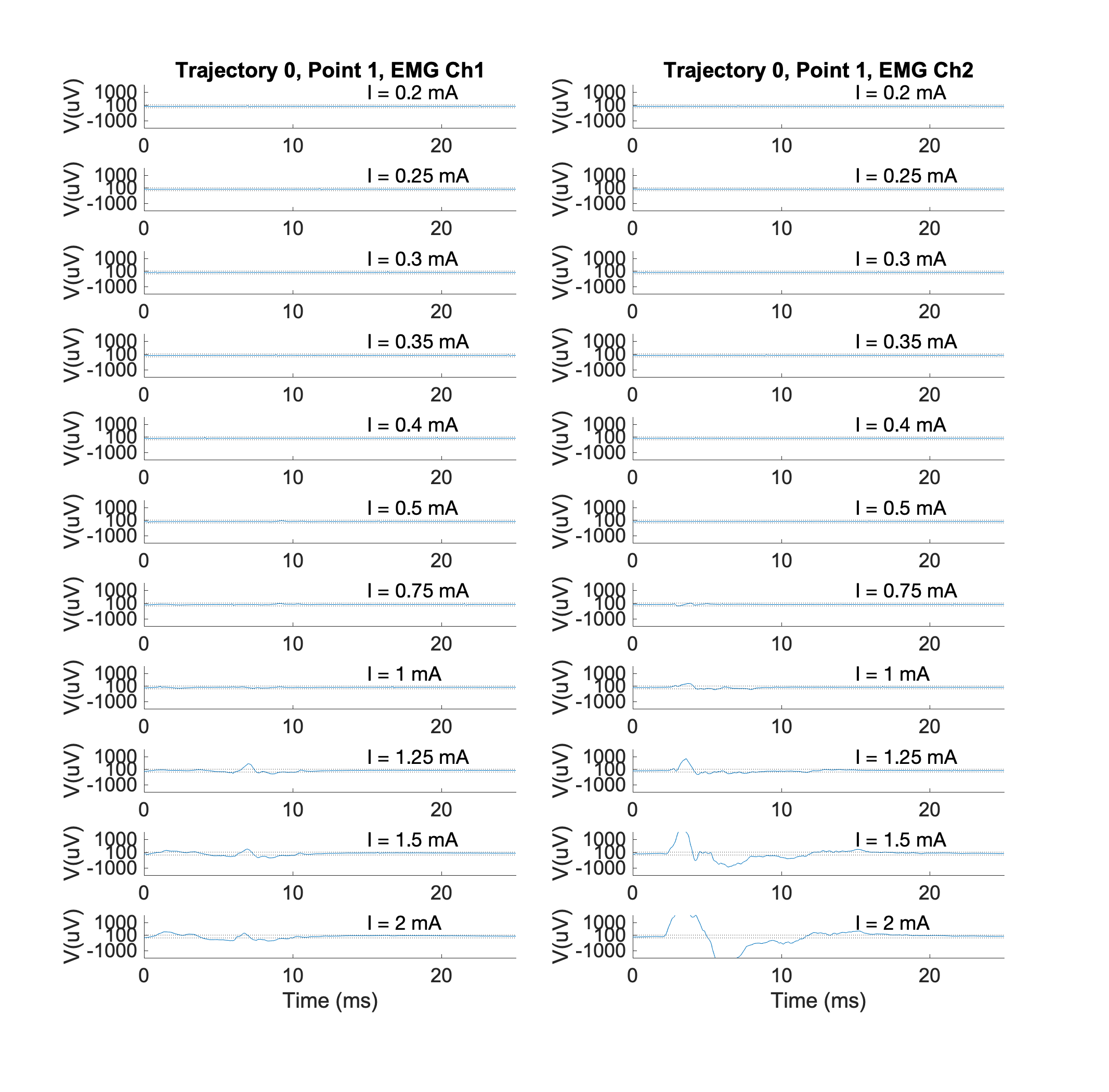

Supplement: Supplementary Data Sheet 1 — Overview of recorded electromyography data showing CMAP responses to the stimulation intensity ramp at each measurement point for the monopolar stimulation. A graph with maximum CMAP responses of monopolar stimulation for each trajectory is depicted. A Summary report (Subject 1, 2, 3.docx) of CMAP responses (for monopolar stimulation) in trajectories with potential FN damage are presented. Data sets of bipolar stimulation can be shared if the reader is interested (see Data Availability Statement). [file Data_Sheet_1.ZIP › Analysis_EMG_Amplitude_Changes/EMGAmp_OutputData/Subject3/Subject3_Traj0_Point1_EMGepochs.png]

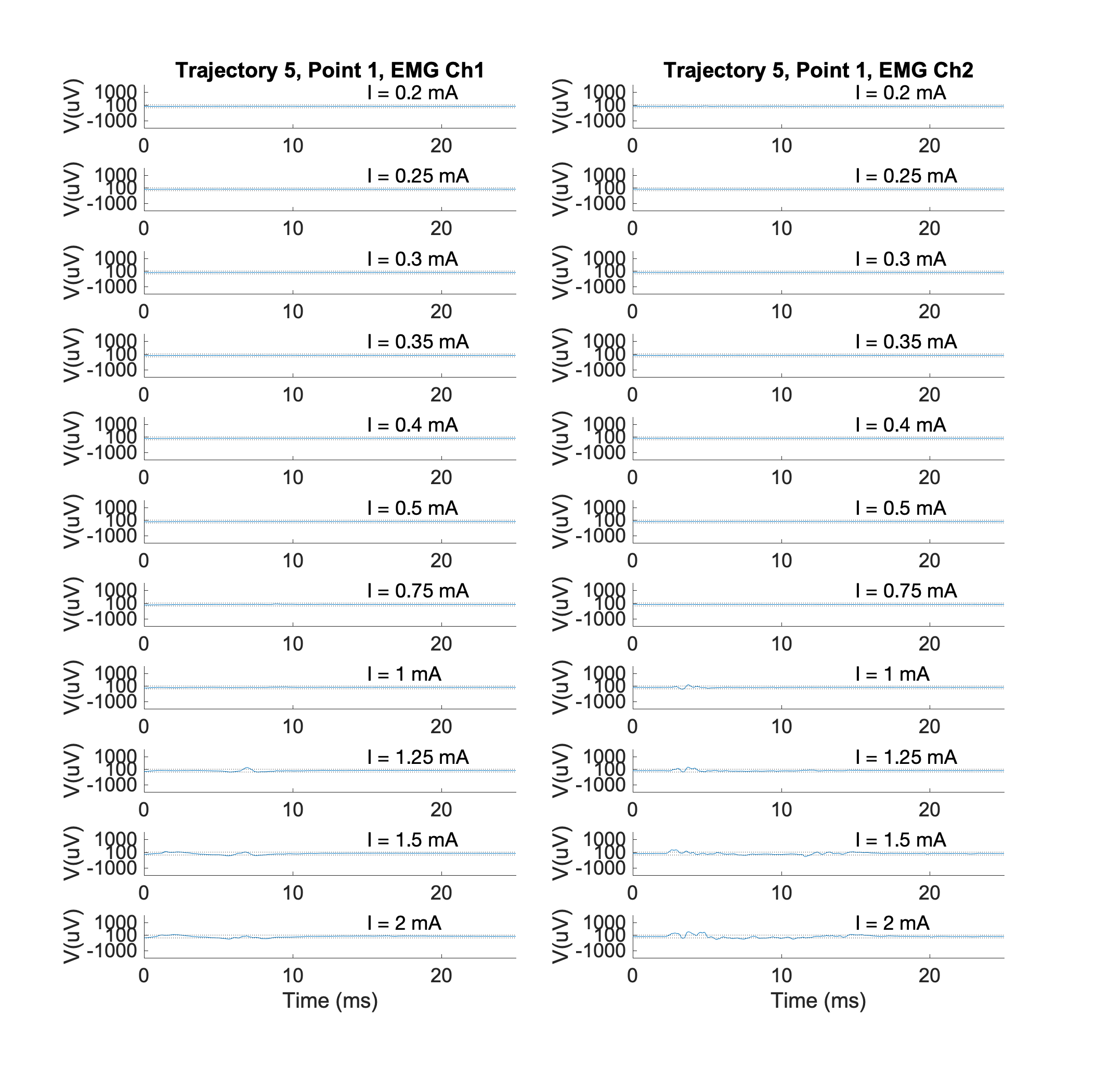

Supplement: Supplementary Data Sheet 1 — Overview of recorded electromyography data showing CMAP responses to the stimulation intensity ramp at each measurement point for the monopolar stimulation. A graph with maximum CMAP responses of monopolar stimulation for each trajectory is depicted. A Summary report (Subject 1, 2, 3.docx) of CMAP responses (for monopolar stimulation) in trajectories with potential FN damage are presented. Data sets of bipolar stimulation can be shared if the reader is interested (see Data Availability Statement). [file Data_Sheet_1.ZIP › Analysis_EMG_Amplitude_Changes/EMGAmp_OutputData/Subject3/Subject3_Traj5_Point1_EMGepochs.png]

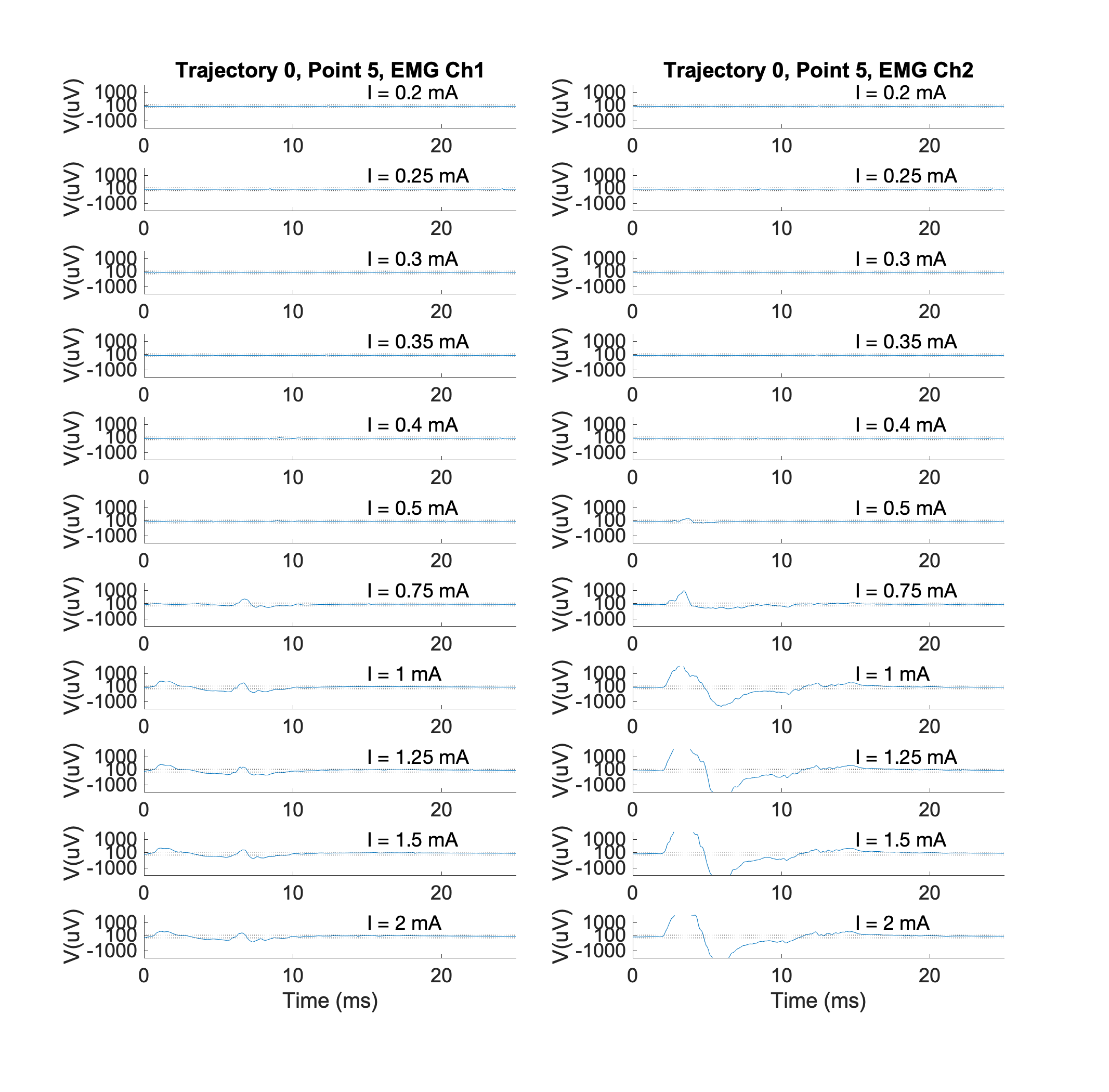

Supplement: Supplementary Data Sheet 1 — Overview of recorded electromyography data showing CMAP responses to the stimulation intensity ramp at each measurement point for the monopolar stimulation. A graph with maximum CMAP responses of monopolar stimulation for each trajectory is depicted. A Summary report (Subject 1, 2, 3.docx) of CMAP responses (for monopolar stimulation) in trajectories with potential FN damage are presented. Data sets of bipolar stimulation can be shared if the reader is interested (see Data Availability Statement). [file Data_Sheet_1.ZIP › Analysis_EMG_Amplitude_Changes/EMGAmp_OutputData/Subject3/Subject3_Traj0_Point5_EMGepochs.png]

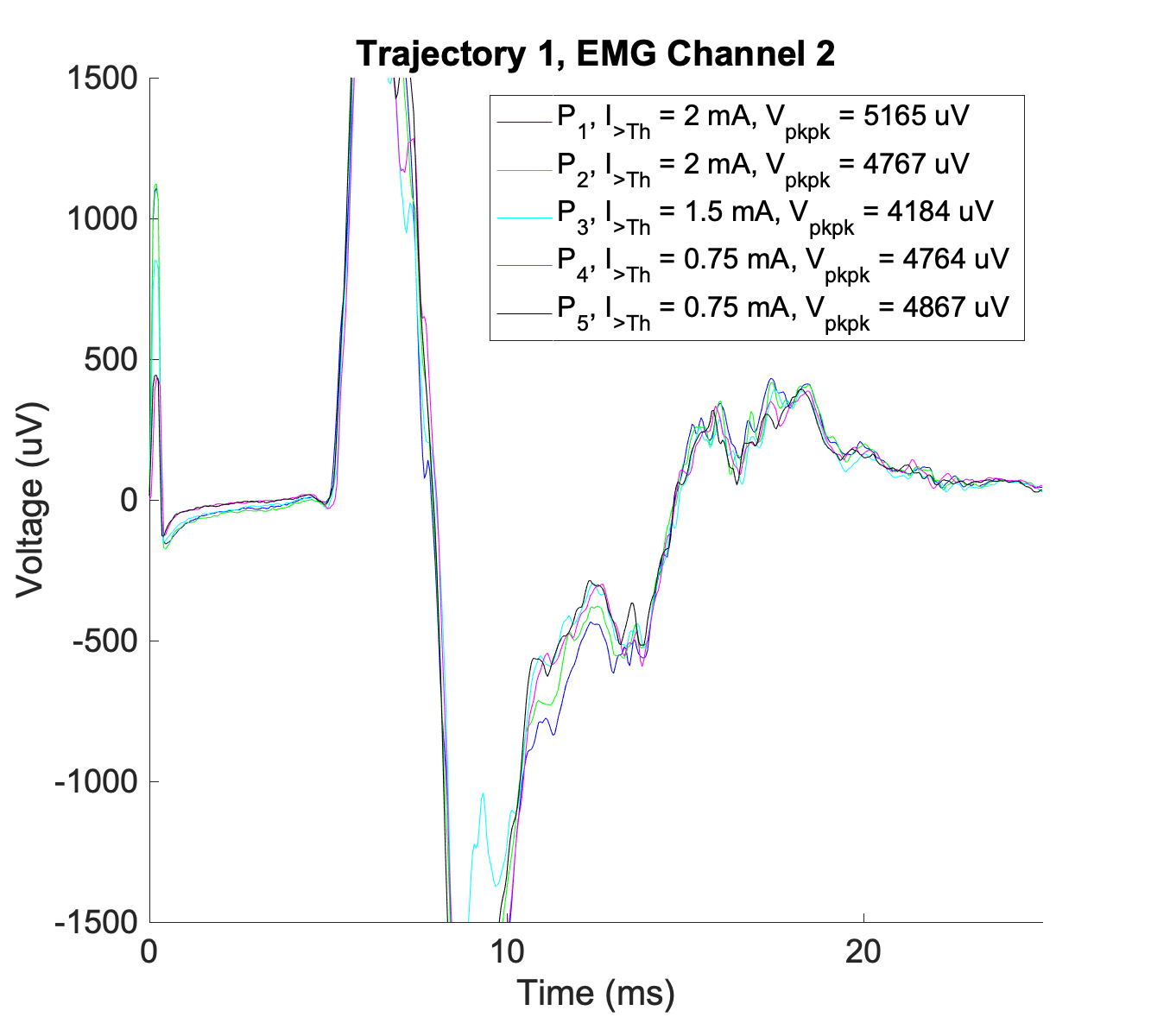

Supplement: Supplementary Data Sheet 1 — Overview of recorded electromyography data showing CMAP responses to the stimulation intensity ramp at each measurement point for the monopolar stimulation. A graph with maximum CMAP responses of monopolar stimulation for each trajectory is depicted. A Summary report (Subject 1, 2, 3.docx) of CMAP responses (for monopolar stimulation) in trajectories with potential FN damage are presented. Data sets of bipolar stimulation can be shared if the reader is interested (see Data Availability Statement). [file Data_Sheet_1.ZIP › Analysis_EMG_Amplitude_Changes/EMGAmp_OutputData/Subject3/Subject3_Traj1_AllPoints_EMG_CH2.png]

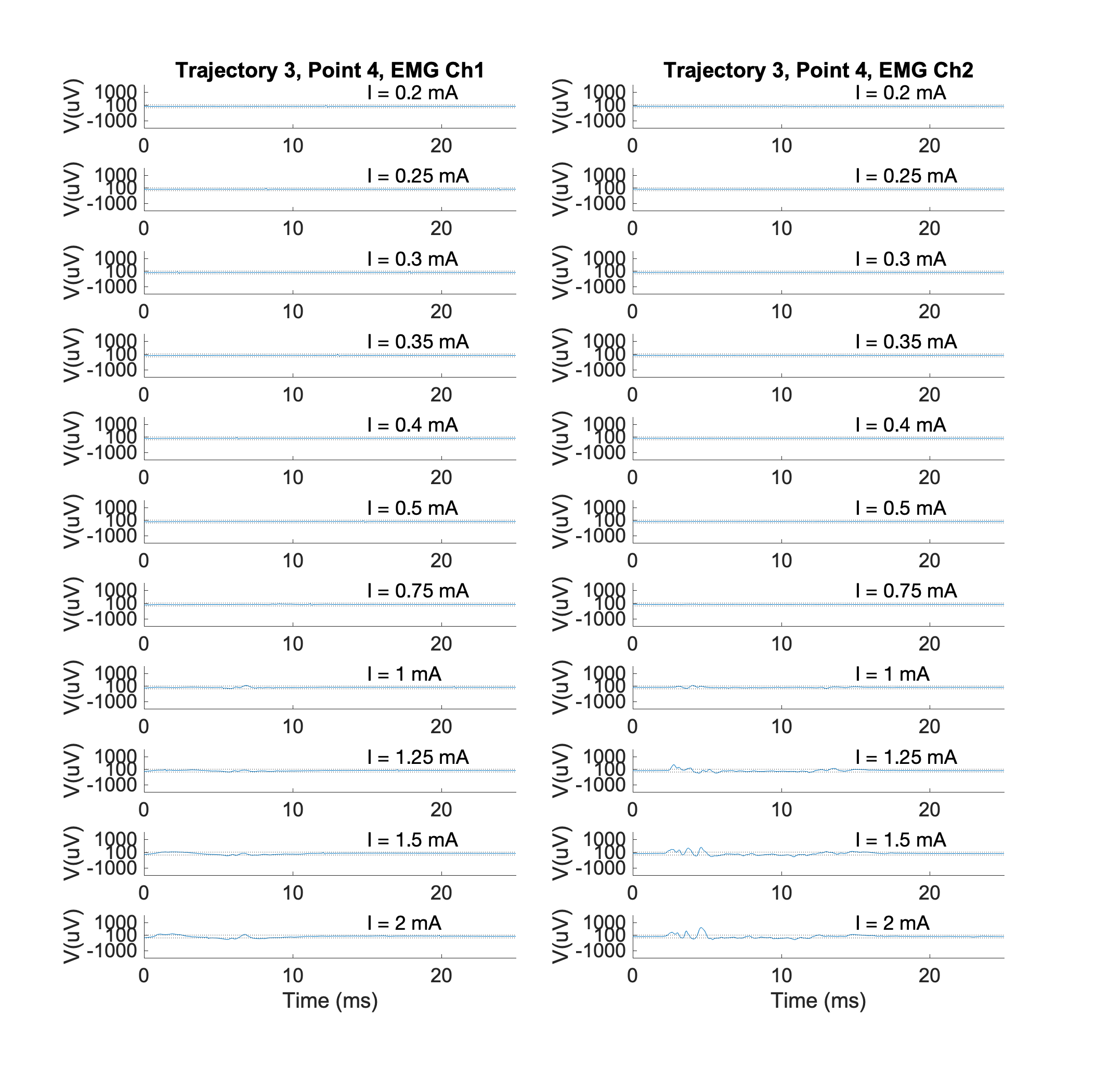

Supplement: Supplementary Data Sheet 1 — Overview of recorded electromyography data showing CMAP responses to the stimulation intensity ramp at each measurement point for the monopolar stimulation. A graph with maximum CMAP responses of monopolar stimulation for each trajectory is depicted. A Summary report (Subject 1, 2, 3.docx) of CMAP responses (for monopolar stimulation) in trajectories with potential FN damage are presented. Data sets of bipolar stimulation can be shared if the reader is interested (see Data Availability Statement). [file Data_Sheet_1.ZIP › Analysis_EMG_Amplitude_Changes/EMGAmp_OutputData/Subject3/Subject3_Traj3_Point4_EMGepochs.png]

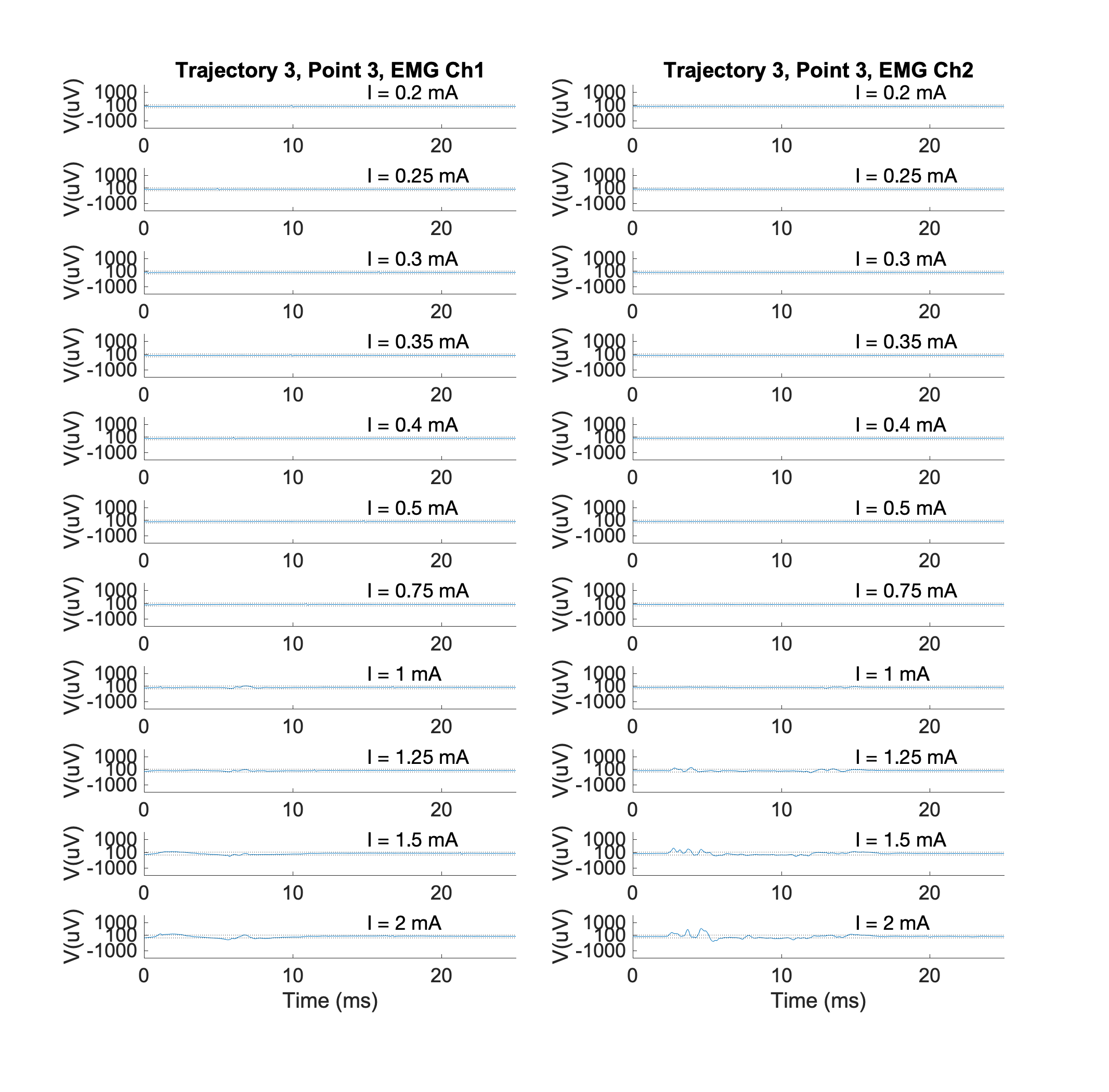

Supplement: Supplementary Data Sheet 1 — Overview of recorded electromyography data showing CMAP responses to the stimulation intensity ramp at each measurement point for the monopolar stimulation. A graph with maximum CMAP responses of monopolar stimulation for each trajectory is depicted. A Summary report (Subject 1, 2, 3.docx) of CMAP responses (for monopolar stimulation) in trajectories with potential FN damage are presented. Data sets of bipolar stimulation can be shared if the reader is interested (see Data Availability Statement). [file Data_Sheet_1.ZIP › Analysis_EMG_Amplitude_Changes/EMGAmp_OutputData/Subject3/Subject3_Traj3_Point3_EMGepochs.png]

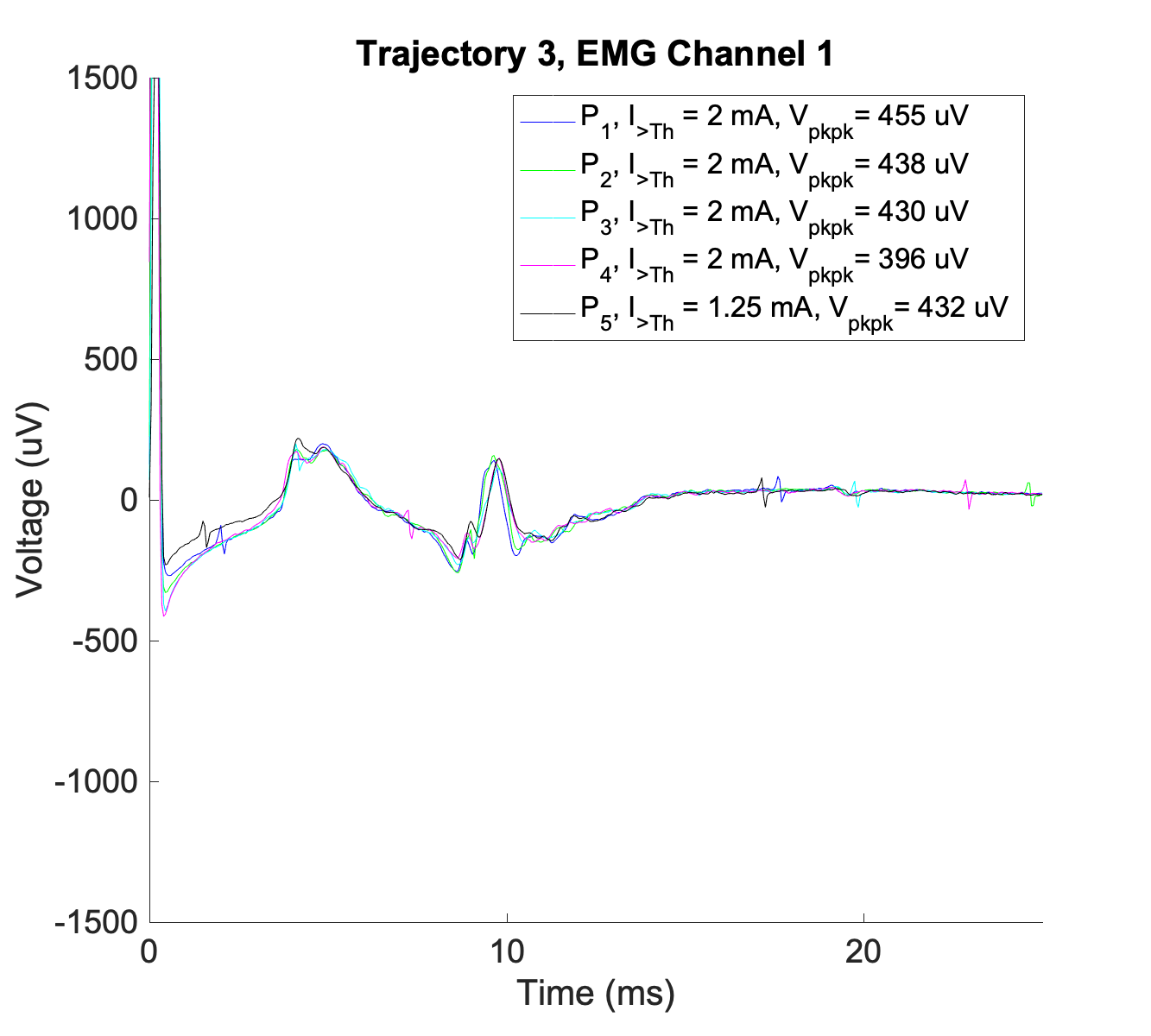

Supplement: Supplementary Data Sheet 1 — Overview of recorded electromyography data showing CMAP responses to the stimulation intensity ramp at each measurement point for the monopolar stimulation. A graph with maximum CMAP responses of monopolar stimulation for each trajectory is depicted. A Summary report (Subject 1, 2, 3.docx) of CMAP responses (for monopolar stimulation) in trajectories with potential FN damage are presented. Data sets of bipolar stimulation can be shared if the reader is interested (see Data Availability Statement). [file Data_Sheet_1.ZIP › Analysis_EMG_Amplitude_Changes/EMGAmp_OutputData/Subject3/Subject3_Traj3_AllPoints_EMG_CH1.png]

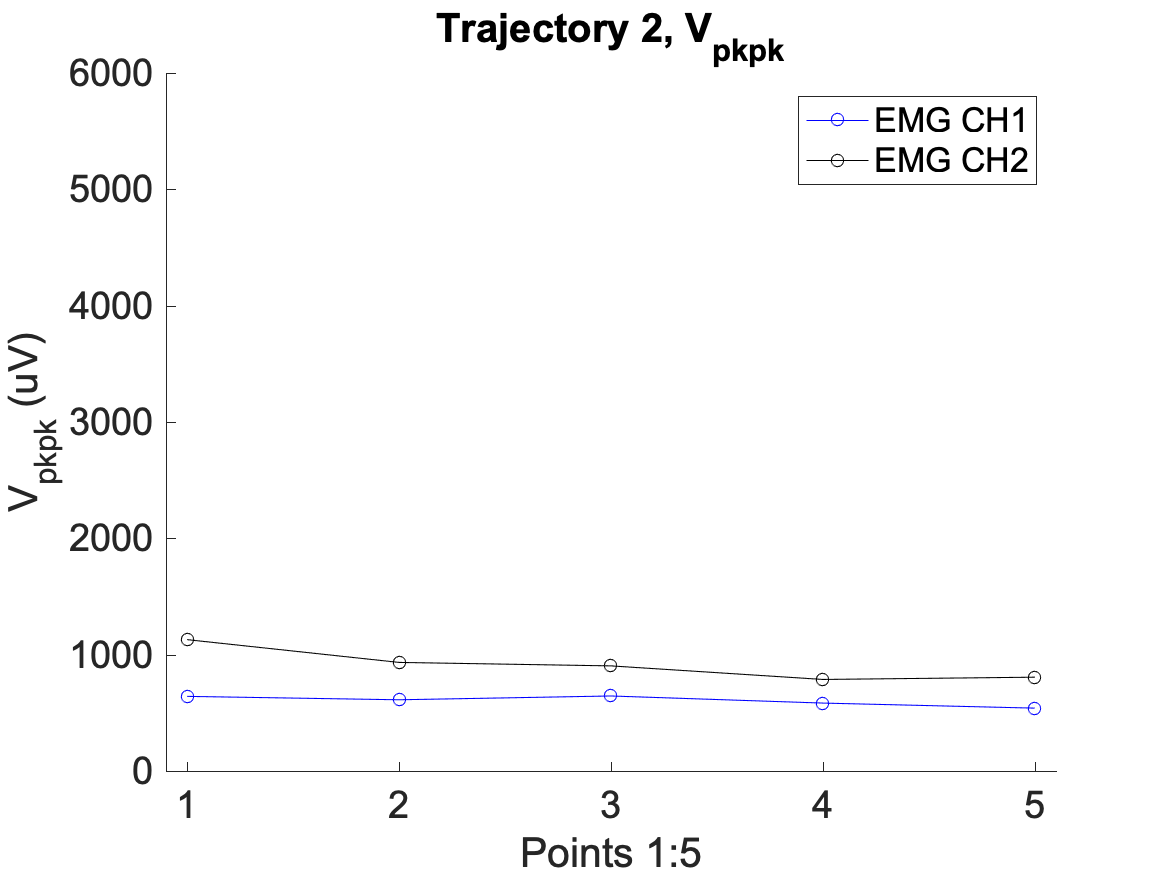

Supplement: Supplementary Data Sheet 1 — Overview of recorded electromyography data showing CMAP responses to the stimulation intensity ramp at each measurement point for the monopolar stimulation. A graph with maximum CMAP responses of monopolar stimulation for each trajectory is depicted. A Summary report (Subject 1, 2, 3.docx) of CMAP responses (for monopolar stimulation) in trajectories with potential FN damage are presented. Data sets of bipolar stimulation can be shared if the reader is interested (see Data Availability Statement). [file Data_Sheet_1.ZIP › Analysis_EMG_Amplitude_Changes/EMGAmp_OutputData/Subject3/Subject3_Traj2_Vpkk.png]

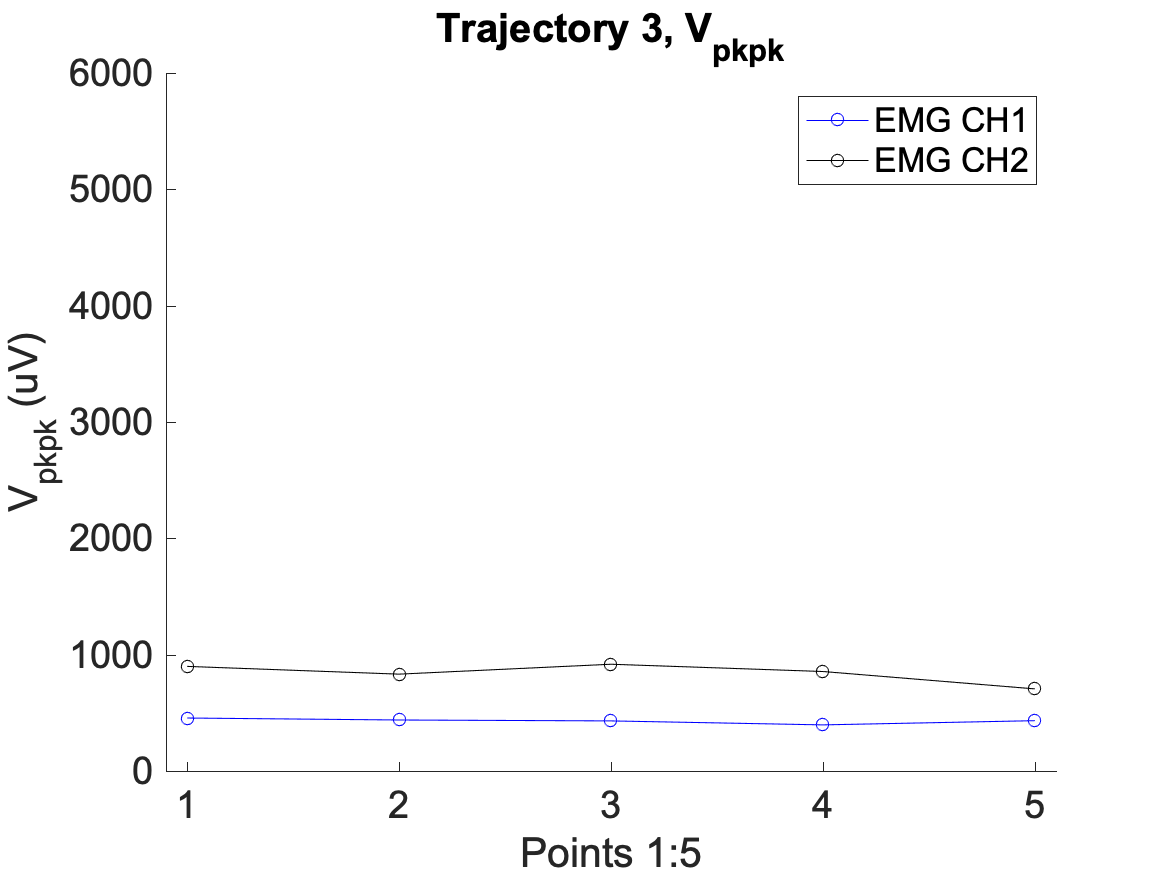

Supplement: Supplementary Data Sheet 1 — Overview of recorded electromyography data showing CMAP responses to the stimulation intensity ramp at each measurement point for the monopolar stimulation. A graph with maximum CMAP responses of monopolar stimulation for each trajectory is depicted. A Summary report (Subject 1, 2, 3.docx) of CMAP responses (for monopolar stimulation) in trajectories with potential FN damage are presented. Data sets of bipolar stimulation can be shared if the reader is interested (see Data Availability Statement). [file Data_Sheet_1.ZIP › Analysis_EMG_Amplitude_Changes/EMGAmp_OutputData/Subject3/Subject3_Traj3_Vpkk.png]

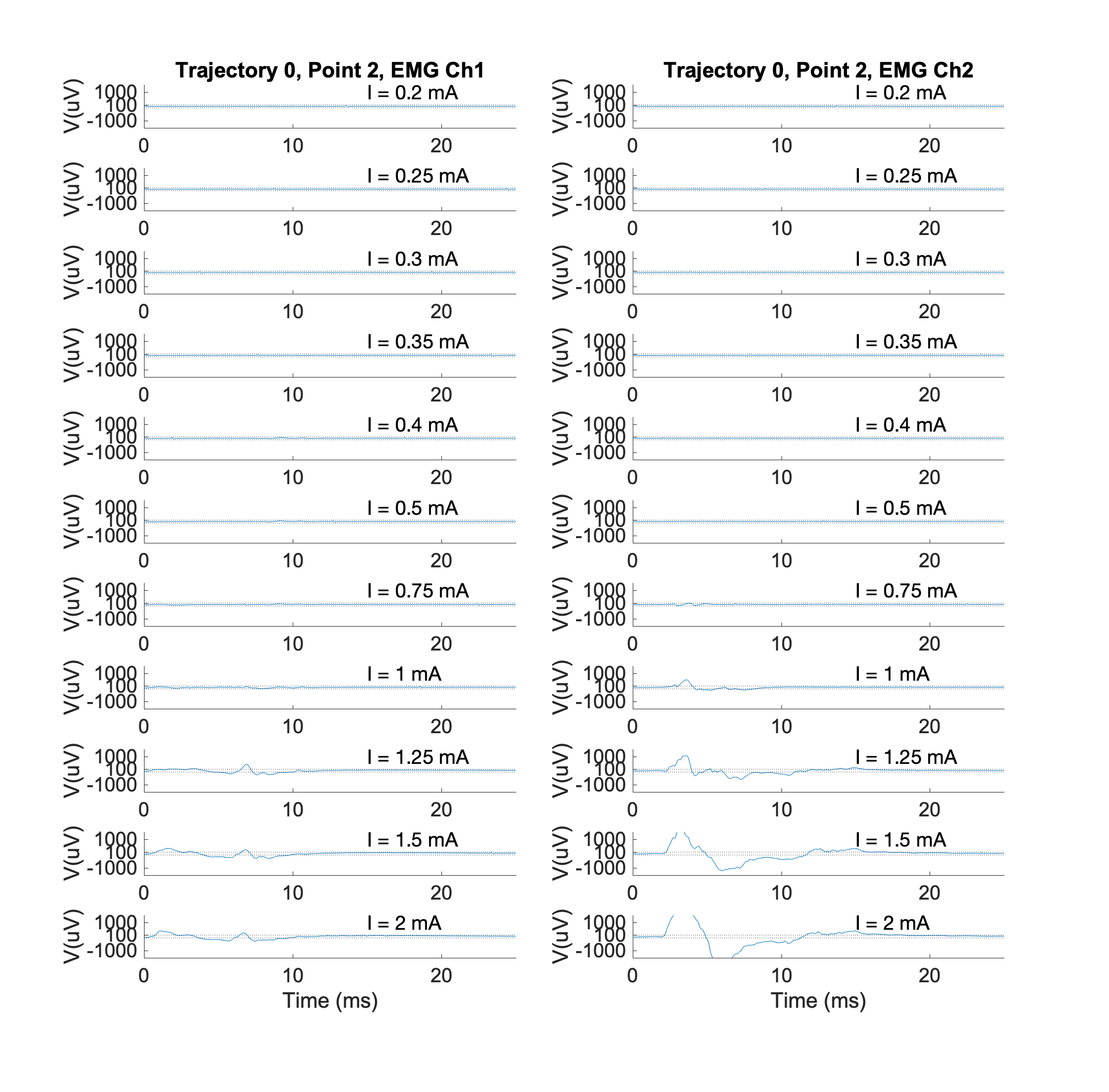

Supplement: Supplementary Data Sheet 1 — Overview of recorded electromyography data showing CMAP responses to the stimulation intensity ramp at each measurement point for the monopolar stimulation. A graph with maximum CMAP responses of monopolar stimulation for each trajectory is depicted. A Summary report (Subject 1, 2, 3.docx) of CMAP responses (for monopolar stimulation) in trajectories with potential FN damage are presented. Data sets of bipolar stimulation can be shared if the reader is interested (see Data Availability Statement). [file Data_Sheet_1.ZIP › Analysis_EMG_Amplitude_Changes/EMGAmp_OutputData/Subject3/Subject3_Traj0_Point2_EMGepochs.png]

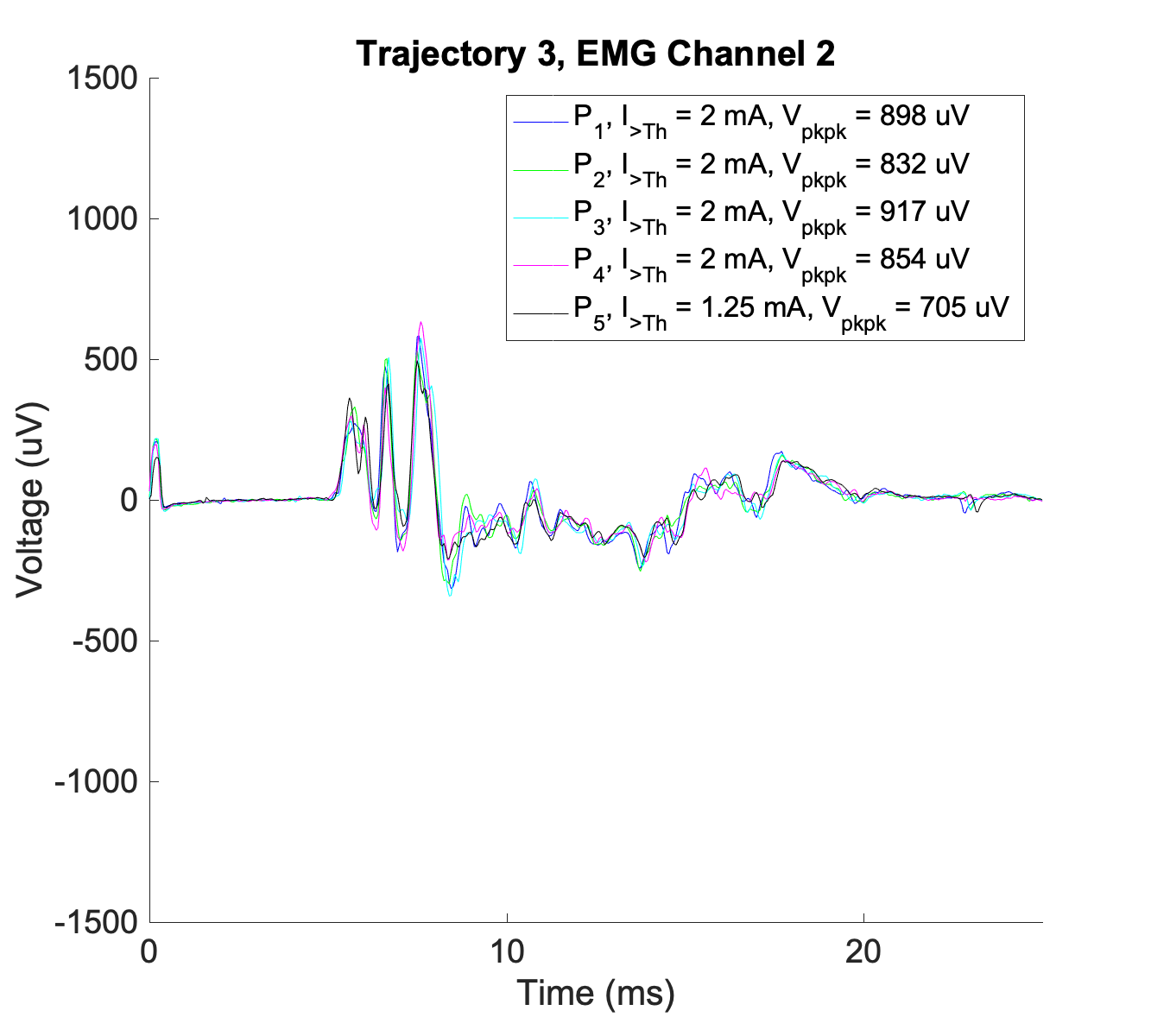

Supplement: Supplementary Data Sheet 1 — Overview of recorded electromyography data showing CMAP responses to the stimulation intensity ramp at each measurement point for the monopolar stimulation. A graph with maximum CMAP responses of monopolar stimulation for each trajectory is depicted. A Summary report (Subject 1, 2, 3.docx) of CMAP responses (for monopolar stimulation) in trajectories with potential FN damage are presented. Data sets of bipolar stimulation can be shared if the reader is interested (see Data Availability Statement). [file Data_Sheet_1.ZIP › Analysis_EMG_Amplitude_Changes/EMGAmp_OutputData/Subject3/Subject3_Traj3_AllPoints_EMG_CH2.png]

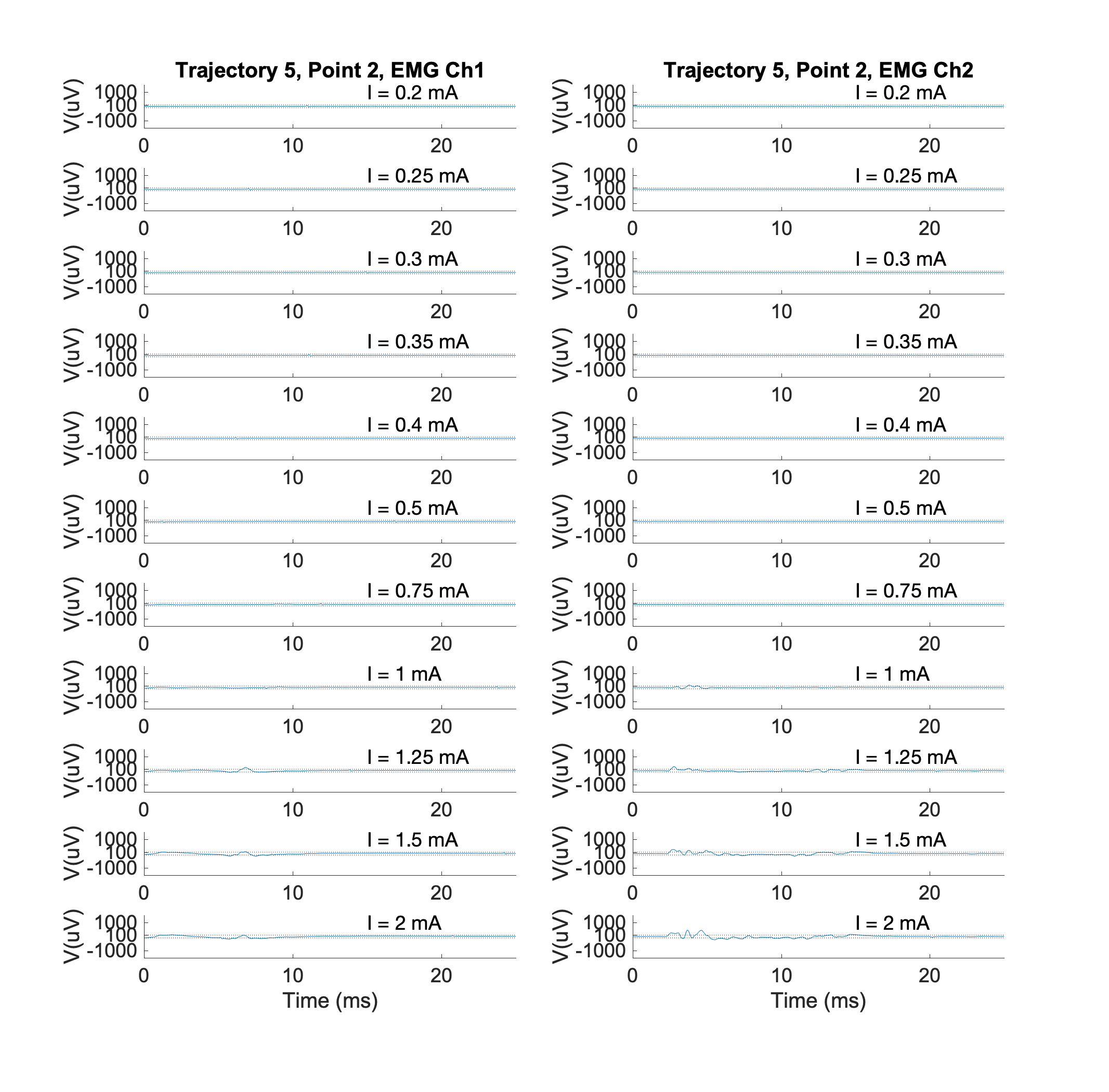

Supplement: Supplementary Data Sheet 1 — Overview of recorded electromyography data showing CMAP responses to the stimulation intensity ramp at each measurement point for the monopolar stimulation. A graph with maximum CMAP responses of monopolar stimulation for each trajectory is depicted. A Summary report (Subject 1, 2, 3.docx) of CMAP responses (for monopolar stimulation) in trajectories with potential FN damage are presented. Data sets of bipolar stimulation can be shared if the reader is interested (see Data Availability Statement). [file Data_Sheet_1.ZIP › Analysis_EMG_Amplitude_Changes/EMGAmp_OutputData/Subject3/Subject3_Traj5_Point2_EMGepochs.png]

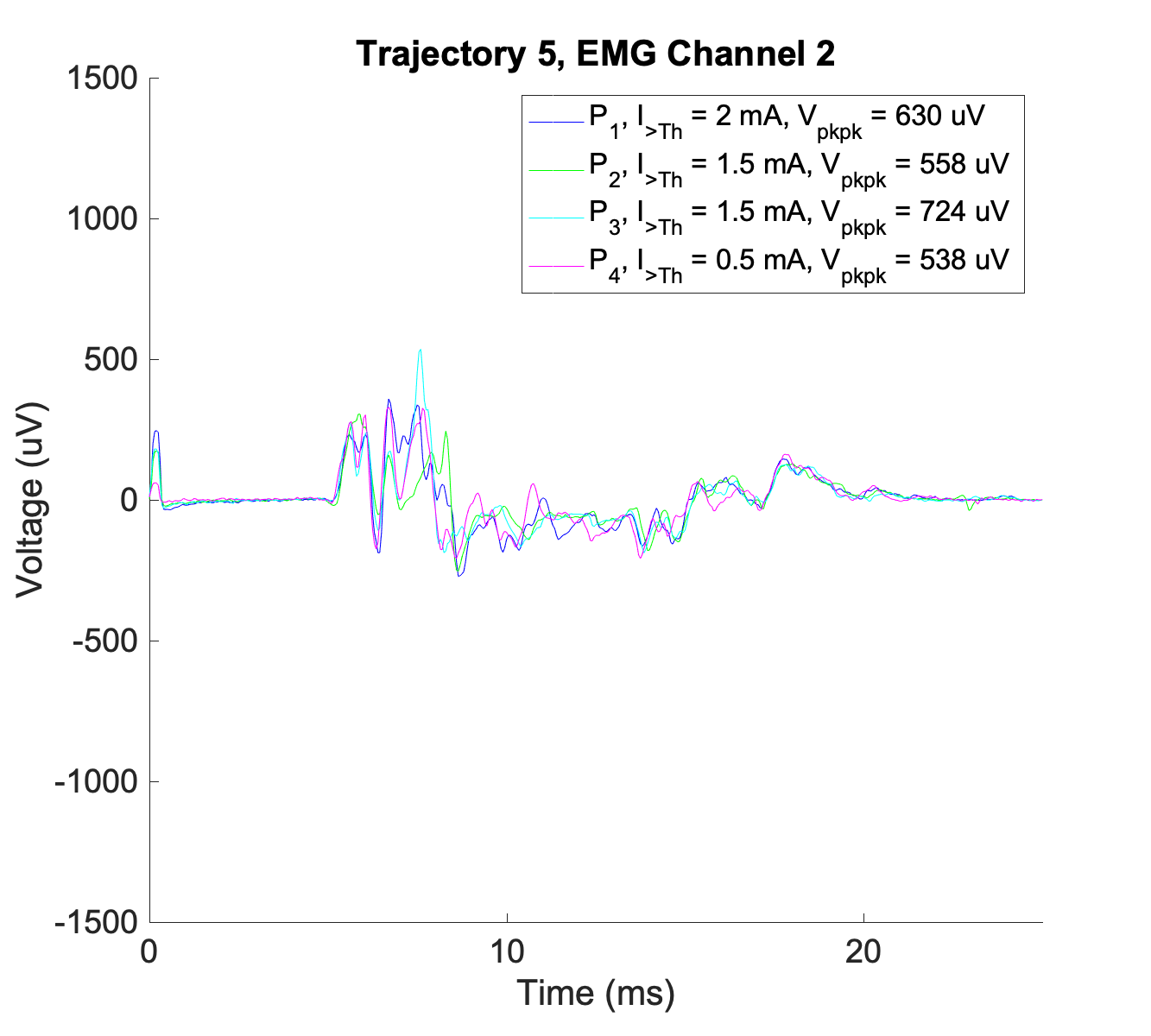

Supplement: Supplementary Data Sheet 1 — Overview of recorded electromyography data showing CMAP responses to the stimulation intensity ramp at each measurement point for the monopolar stimulation. A graph with maximum CMAP responses of monopolar stimulation for each trajectory is depicted. A Summary report (Subject 1, 2, 3.docx) of CMAP responses (for monopolar stimulation) in trajectories with potential FN damage are presented. Data sets of bipolar stimulation can be shared if the reader is interested (see Data Availability Statement). [file Data_Sheet_1.ZIP › Analysis_EMG_Amplitude_Changes/EMGAmp_OutputData/Subject3/Subject3_Traj5_AllPoints_EMG_CH2.png]

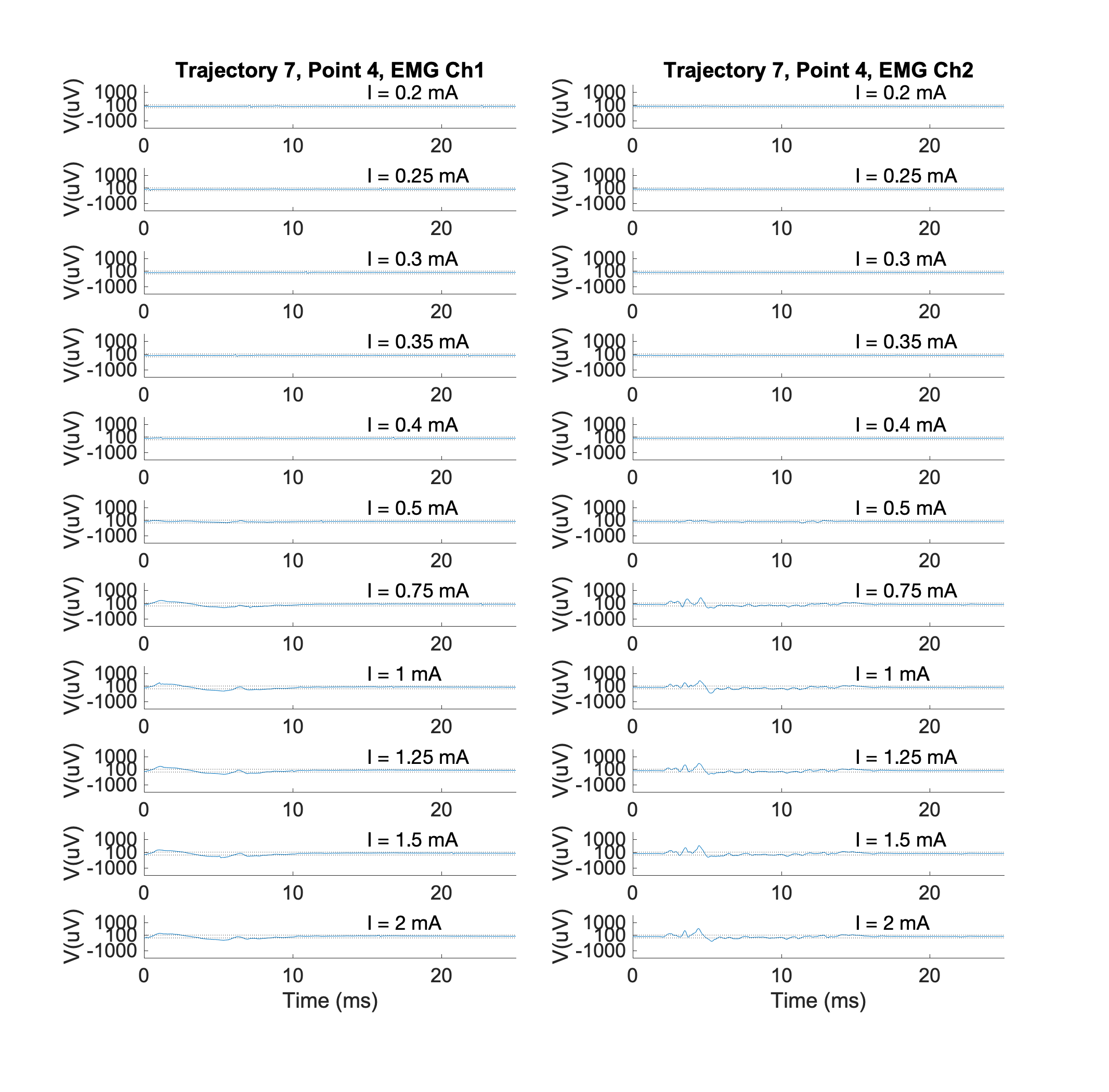

Supplement: Supplementary Data Sheet 1 — Overview of recorded electromyography data showing CMAP responses to the stimulation intensity ramp at each measurement point for the monopolar stimulation. A graph with maximum CMAP responses of monopolar stimulation for each trajectory is depicted. A Summary report (Subject 1, 2, 3.docx) of CMAP responses (for monopolar stimulation) in trajectories with potential FN damage are presented. Data sets of bipolar stimulation can be shared if the reader is interested (see Data Availability Statement). [file Data_Sheet_1.ZIP › Analysis_EMG_Amplitude_Changes/EMGAmp_OutputData/Subject3/Subject3_Traj7_Point4_EMGepochs.png]
